# Suppressing Glycerol‐3‐phosphate Phosphatase and Enhancing Glycerol‐3‐Phosphate Shuttle Flux Crucial for High‐Efficiency Fatty Acid Production in the Fast‐Growing Oleaginous *Schizochytrium*

**DOI:** 10.1002/advs.202510021

**Published:** 2025-09-24

**Authors:** Fangzhong Wang, Weijia Jin, Tiantian Wang, Junkai Ji, Kun Cao, Jianqiang Li, Lei Chen, Weiwen Zhang

**Affiliations:** ^1^ State Key Laboratory of Synthetic Biology, School of Synthetic Biology and Biomanufacturing Tianjin University Tianjin 300350 P. R. China; ^2^ Center for Biosafety Research and Strategy, School of Synthetic Biology and Biomanufacturing Tianjin University Tianjin 300350 P. R. China; ^3^ National Engineering Laboratory for Big Data System Computing Technology Shenzhen University Shenzhen 518060 P. R. China; ^4^ College of Computer Science and Software Engineering Shenzhen University Shenzhen 518060 P. R. China

**Keywords:** fatty acid, glycerol‐3‐phosphate phosphatase, productivity, *Schizochytrium*, titer

## Abstract

The industrial microbial production of fatty acids is limited by low titers and productivities, highlighting the need for chassis engineering and mechanistic insights into efficient metabolic pathways. The heterotrophic microalga *Schizochytrium* offers distinct advantages, and *Schizochytrium* ZW1 is engineered into a high‐efficiency fatty acid production chassis. A fluorescence‐activated cell sorting strategy is developed to overcome cellular aggregation, enabling the rapid isolation of *Schizochytrium* ZW2, which exhibited enhanced growth and fatty acid accumulation. Multiomics analysis of ZW2 revealed that mutations suppressing glycerol‐3‐phosphate phosphatase activity promoted triacylglycerol synthesis, and mutations in the polyunsaturated fatty acid synthase C subunit increased polyunsaturated fatty acid production. Furthermore, increased glycerol‐3‐phosphate shuttle flux in ZW2 enhanced both biomass and fatty acid accumulation. The crucial roles of these mutations are confirmed by introducing modifications to enhance glycerol‐3‐phosphate shuttle and suppress glycerol‐3‐phosphate phosphatase activity in ZW1, which yielded ≈80% of the fatty acid production of ZW2. Further engineering generated *Schizochytrium* ZW2‐D‐L2, which achieved total fatty acid and triacylglycerol titers of 94.65 and 88.51 g L^−1^, and productivities of 1.39 and 1.30 g L^−1^ h^−1^, respectively. This performance doubles that of ZW1 and surpasses microbial benchmarks. This work elucidates regulatory mechanisms and establishes *Schizochytrium* as a scalable platform for industrial biotechnology.

## Graphical abstract

This study establishes an efficient oleaginous heterotrophic microalga, *Schizochytrium*, through chassis optimization and metabolic engineering, significantly enhancing fatty acid titers and productivities. It also identifies a regulatory mechanism that coordinates biomass and lipid synthesis, providing a rational target for metabolic pathway rewiring.

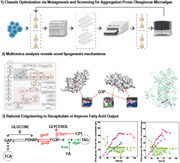

## 1 Introduction

Fatty acids have multiple valuable applications, for example, as sustainable biofuels that address energy security and fossil fuel pollution and as ω‐3 polyunsaturated variant supplements to offer health benefits such as anti‐inflammatory activity and neonatal development support.^[^ ^1^, ^2^ ^]^ In recent decades, considerable advances have been made in microbial fatty acid production using various chassis, and key performance metrics from earlier studies with different microbial strains are summarized in **Table** **1**.^[^ ^3^, ^4^, ^5^, ^6^, ^7^, ^8^, ^9^, ^10^, ^11^ ^]^ However, achieving high titers and productivities remains a major challenge. Titer and productivity serve as critical metrics for evaluating the efficiency of a bioprocess. Suboptimal levels of either parameter directly lead to increased substrate consumption, elevated capital expenditure, and higher operational costs, thereby significantly elevating the unit production cost.^[^ ^12^, ^13^, ^14^ ^]^ To address the issue of low titers and productivities, several strategies, such as engineering diauxic shifts into monoauxic biomass‒lipid coproduction or constructing new acetyl‐CoA synthesis pathways to decouple lipid biosynthesis from biomass production, have been developed and evaluated.^[^ ^14^, ^15^ ^]^ Despite some progress, enhancing the titer and productivity of microbially derived fatty acids to achieve cost competitiveness against other alternatives, such as plant‐ or animal‐based production, remains a major challenge.^[^ ^13^ ^]^

**Table 1:** Fermentation performance comparison among fatty acid‐producing chassis.

Product	Chassis	Time [h]	Biomass		TFA/FAME/lipid/TAG				Source
			Titer	Productivity	Titer	Yield	Productivity	Content	
			[g L^−1^]	[g L^−1^ h^−1^]	[g L^−1^]	[g g^−1^]	[g L^−1^ h^−1^]	[%]	
TFA	*S*. *cerevisiae*	48	12.22	0.25	2.20	0.12	0.05	≈18.00^a)^	11
TFA	*C*. *glutamicum*	45	16.85	0.37	3.02	0.08	0.07	17.80	10
TFA	*E*. *coli*	60	8.97	0.15	7.00	0.28	0.12	78.00	9
Lipid	*R*. *toruloides*	134	107.45	0.80	72.53	0.23	0.54	67.50	8
Lipid	*C*. *oleaginosus*	50	118.00	2.36	29.50	n/a	0.59	25.00	7
TAG	*Rhodococcus opacus* PD630	96	109.80	1.14	82.90	0.29	0.86	75.50	6
Lipid	*R. glutinis*	≈83^a)^	180.00	2.17	72.00	n/a	0.87	40.00	5
FAME	*Schizochytrium* ATCC20888	87	171.50	1.57	84.89	n/a	0.98	49.50	4
FAME	*Y*. *lipolytica*	84	148.00	1.76	98.90	0.27	1.30	66.80	3
FAME	*Schizochytrium* ZW2‐D‐L2	68	122.64	1.80	99.40	0.28	1.46	81.13	This study
TAG	*Schizochytrium* ZW2‐D‐L2	68	122.64	1.80	88.51	0.25	1.30	72.22	This study

n/a, not available; TFA: Total Fatty Acid; TAG: Triacylglycerol; FAME: Fatty Acid Methyl Ester.

^a)^ The parameters were derived from the graphical data presented in the original publication.

One promising approach to increase titer and productivity is the utilization of fast‐growing chassis with high carbon assimilation efficiency.^[^ ^16^ ^]^ Among these, marine thraustochytrids have garnered significant attention due to their rapid growth rates, efficient carbon source uptake capabilities, high lipid droplet accumulation capacity, and adaptability to seawater‐based cultivation systems. For example, the oleaginous marine microalga *Schizochytrium* sp. SH103, when cultivated in a medium with approximately half‐strength seawater salinity for 48 h, achieved a fatty acid content of 48.31%, a titer of 48.6 g L^−1^, and a productivity of 1.0 g L^−1^ h^−1^.^[^ ^17^ ^]^ However, the limited genetic tractability and poorly characterized metabolic pathways of *Schizochytrium* sp. hinder genome‐scale metabolic engineering efforts – challenges shared by other fast‐growing, high‐carbon‐utilization chassis, such as *Chlorella*, *Cutaneotrichosporon oleaginosus*, and *Rhodotorula glutinis*.^[^ ^5^, ^7^, ^16^, ^18^, ^19^ ^]^ This underscores the urgent need to develop alternative strategies for achieving desired phenotypes.

Another promising strategy for enhancing fatty acid titer and productivity involves the identification and utilization of novel regulatory mechanisms.^[^ ^20^ ^]^ For instance, the alleviation of the allosteric inhibition of fatty acid biosynthesis by saturated acyl‐CoA in high‐lipid‐producing *Y. lipolytica* strains conferred a threefold growth advantage, leading to 3.5–5.0 times greater productivity than that previously reported.^[^ ^21^ ^]^ Optimizing intracellular redox metabolism to maximize electron flux toward fatty acid precursors has enabled the highest reported titer and productivity to date.^[^ ^3^ ^]^ In addition, emerging evidence suggests potential critical roles in fatty acid synthesis for the glycerol‐3‐phosphate (G3P) shuttle system, transcriptional regulation, and lipid droplet biogenesis.^[^ ^22^, ^23^, ^24^ ^]^ Although their effects on titer and productivity have not been systematically investigated in microbial systems, elucidating these mechanisms could accelerate the engineering of microbial platforms for fatty acid synthesis and production of fatty acid‐derived products.

**Figure 1:**
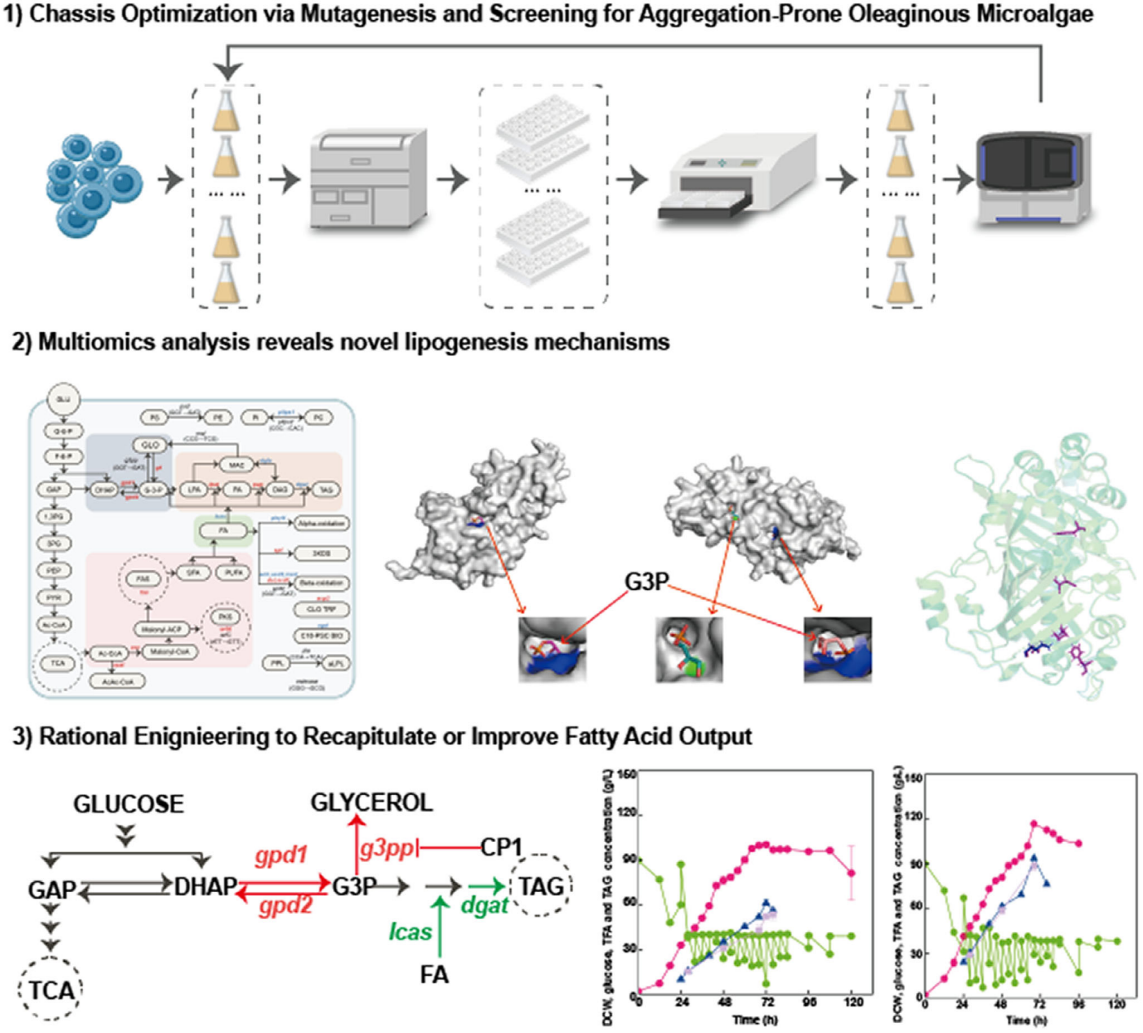
Part I: Chassis optimization. The initial stage involved ARTP mutagenesis followed by screening via FACS to isolate aggregated microalgal mutants. Part II: Multi‐omics analysis. Integrated multi‐omics approaches were employed to investigate the molecular mechanisms responsible for enhanced fatty acid accumulation in the selected mutants. The left schematic illustrates central carbon metabolism and fatty acid biosynthesis pathways. The two central protein structures depict the parent (left) and mutant (right) forms of G3PP, highlighting changes in docking interactions with the substrate G3P. The rightmost model represents the mutated ORFC protein. Part III: Validation and metabolic engineering. Based on mechanistic insights from Part II, the beneficial mutations were heterologously reconstructed in the parental strain, and novel genetic targets were engineered to further increase fatty acid production. The left diagram outlines the engineering strategies: reconstruction of mutations in the parent strain (red) and additional modifications in the mutant background (green). The middle and right panels present fermentation performance data for the reconstructed and final engineered strains, respectively.

In this study, we developed an efficient heterotrophic algal chassis for fatty acid production and identified novel regulatory mechanisms governing fatty acid synthesis (**Figure** **1**). We first established a rapid evolution strategy to rapidly evolve fast‐growing *Schizochytrium* to obtain a ZW2 strain with high fatty acid titer and productivity. Then, omics‐based mechanism analysis revealed that enhanced G3P shuttle flux and inhibition of G3P degradation were responsible for the observed high fatty acid titer and productivity. Subsequent engineering efforts of ZW2 generated a strain with total fatty acid (TFA) and triacylglycerol (TAG) titers of 94.65 and 88.51 g L^−1^ and productivities of 1.39 and 1.30 g L^−1^ h^−1^, respectively. This work provides insights into the mechanisms underlying high fatty acid synthesis efficiency and establishes a novel industrial platform for scalable production using engineered *Schizochytrium*.

## 2 Results

### 2.1 *Schizochytrium* sp. ZW1 with the Potential for Increased Fatty Acid Titer and Productivity

**Figure 2:**
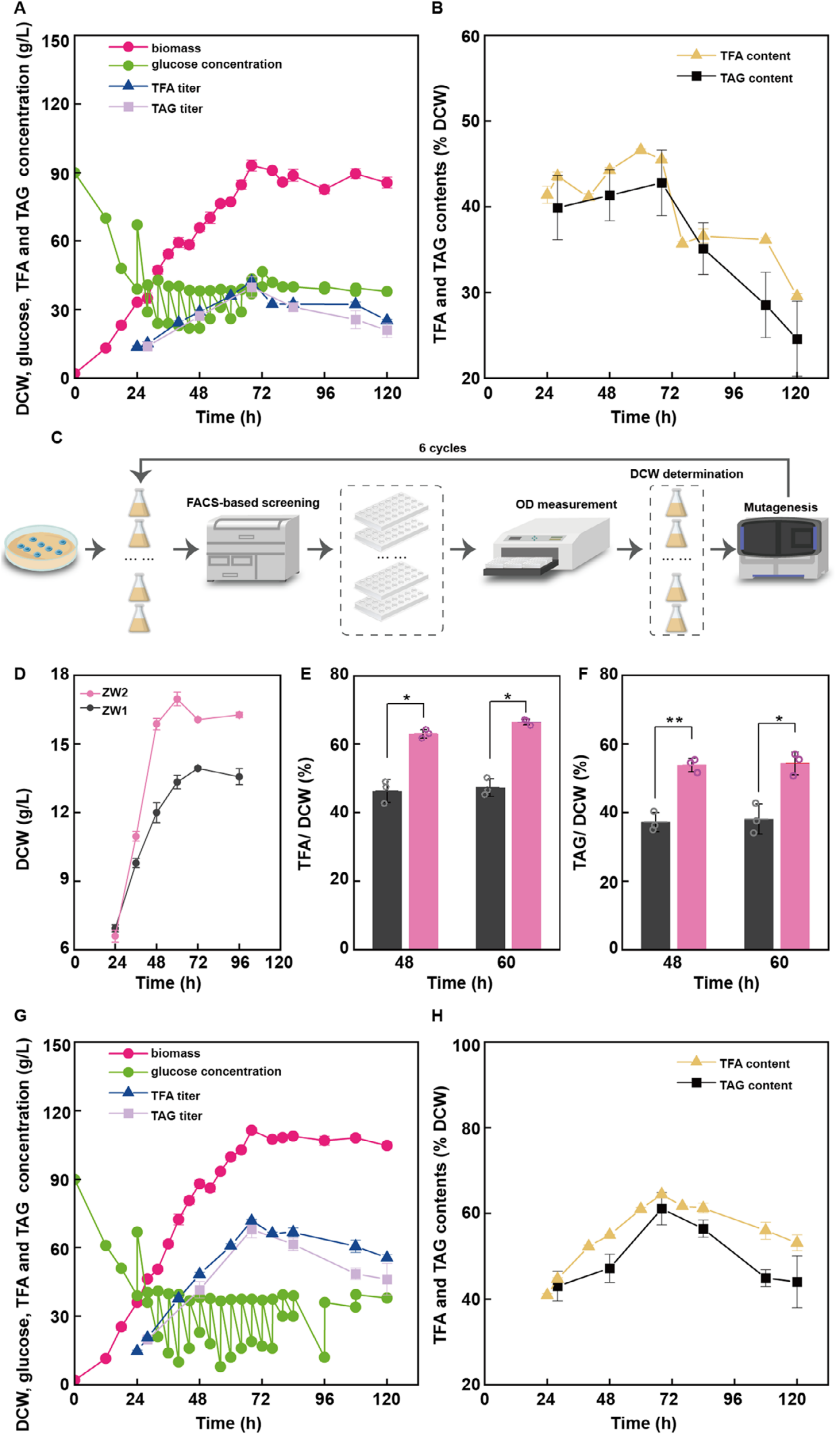
Development of the high‐productivity fatty acid‐producing *Schizochytrium* sp. strain ZW2 through novel screening. A) Growth (pink), residual glucose titer (lime green), TFA titer (dark blue), and TAG titer (lavender) profiles of ZW1 in a 5‐L fermentation system. B) Intracellular TFA (yellow) and TAG content (black) profiles of ZW1 in a 5‐L fermentation system. C) FACS‐based screening strategy for isolating ZW2. D–F) Comparative analysis of ZW1 (dark grey) versus ZW2 (light rose): Growth (D), TFA (E), and TAG (F). G) Growth (pink), residual glucose titer (lime green), TFA titer (dark blue), and TAG titer (lavender) profiles of ZW2 in a 5‐L fermentation system. H) Intracellular TFA (yellow) and TAG content (black) profiles of ZW2 in a 5‐L fermentation system. Error bars represent mean ± SD. For panels A, B, G, and H, data represent *n* = 3 technical replicates. For panels D–F, statistical analysis was performed using a two‐tailed Student's *t*‐test in Microsoft Excel, with *n* = 3 biologically independent samples. Statistical significance was defined as *p* < 0.05. * *p* < 0.05, ** *p* < 0.01.

We first evaluated the fatty acid levels of the *Schizochytrium* sp. ZW1 strain grown under continuous fed‐batch cultivation using a 5‐L fermenter. The feed medium consisted of glucose and yeast extract, with glucose concentrations maintained at 20–40 g L^−1^. Yeast extract supplementation was adjusted to achieve the appropriate carbon/nitrogen (C/N) ratio in the feed medium. The C/N ratio was set to 4:1 during the initial 40 h of fermentation, after which it was adjusted to 7:1 until the end of fermentation. *Schizochytrium* sp. ZW1 entered the logarithmic growth phase at 12 h. Between 12 and 40 h, the strain presented a growth rate of 1.69 ± 0.05 g L^−1^ h^−1^. From 40 to 68 h, the growth rate was decreased to 1.20 ± 0.00 g L^−1^ h^−1^, possibly due to the reduced nitrogen content in the feed medium. The overall exponential growth rate was 1.44 ± 0.03 g L^−1^ h^−1^ for the first 68 h. At 68 h, the culture reached the stationary phase, with a maximum biomass of 92.18 ± 2.01 g L^−1^. At ≈80 h, the culture entered the decline phase, with a slight decrease in biomass, possibly due to cell lysis (**Figure** **2**A; Figure S1A, Supporting Information).

We conducted TFA and TAG measurements across various stages of cellular growth (Figure 2B; Figure S1B, Supporting Information). Notably, after only a short fermentation (24 h), *Schizochytrium* sp. ZW1 accumulated a TFA content of 39.45 ± 2.65%. As nitrogen availability decreased, the TFA content increased slightly, reaching 44.64 ± 0.98% at 68 h, and then decreased to 37.55 ± 2.02% at 76 h. The TAG content also increased continuously at 28, 48, and 68 h, reaching 35.59 ± 5.70%, 37.91 ± 4.60%, and 41.61 ± 3.60%, respectively. Ultimately, the highest TFA concentration (41.15 ± 1.58 g L^−1^) was reached at 68 h, with a fatty acid yield of 0.18 ± 0.01 g g^−1^ and a productivity of 0.61 ± 0.02 g L^−1^ h^−1^. The highest TAG concentration was 38.40 ± 3.92 g L^−1^, with a yield of 0.17 ± 0.02 g g^−1^ and a productivity of 0.56 ± 0.06 g L^−1^ h^−1^, at 68 h. Finally, the total glucose consumed reached 224.96 ± 5.80 g L^−1^ at 68 h. These results demonstrate that *Schizochytrium* sp. ZW1 presents a fast‐growth phenotype coupled with high glucose uptake capacity and high‐flux fatty acid biosynthesis, making it a promising platform for high‐efficiency fatty acid production.

### 2.2 Chassis Optimization via Mutagenesis and Customized Fluorescence‐Activated Cell Sorting for Aggregation‐Prone Oleaginous Microalgae

Compared with those of *Y. lipolytica* and *R. glutinis*, the fatty acid titer and productivity of *Schizochytrium* sp. ZW1 are currently still relatively low and require further improvement.^[^ ^3^, ^5^ ^]^ However, the limited availability of genetic tools hinders genome reprogramming efforts in *Schizochytrium* sp. ZW1.^[^ ^19^ ^]^ To address these challenges, we applied a chassis optimization strategy that combined random mutagenesis with high‐throughput screening. We optimized atmospheric and room temperature plasma (ARTP) conditions and adopted an 80‐s treatment (>90% lethality) for subsequent mutagenesis (Figure S2A, Supporting Information). For fatty acid screening, fluorescence‐activated cell sorting (FACS) technology has proven to be an effective technique in microalgal breeding, but its application to *Schizochytrium* posed specific challenges: *i*) cellular aggregation during oleaginous cultivation (Figure S2B, Supporting Information), *ii*) variable staining efficiency, and *iii*) maintenance of post‐sorting viability.^[^ ^25^, ^26^ ^]^ To mitigate aggregation, we installed a miniature motor at the sample inlet to promote cell dissociation via continuous agitation. Additionally, we designed a multi‐step gating strategy (Figure S2C–E, Supporting Information): P1: Initial whole population; P2: Intact single‐cell fraction; P3: Non‐aggregated subpopulation; P4: Top 1% fluorescent cells (high‐lipid producers), and then fermentation samples were collected at 72 h, the time point when both biomass and lipid content peaked (Figure S2F,G, Supporting Information), thereby minimizing cell cycle‐related variability in intracellular lipid content. We optimized BODIPY 505/515 concentration, and find a concentration of 1.6–2.0 µg mL^−1^ was sufficient for uniform cell staining, and the addition of DMSO did not increase the staining efficiency (Figure S2H,I, Supporting Information). Flow cytometry analysis confirmed that 2.0 µg mL^−1^ BODIPY 505/515 resulted in complete cell staining (Figure S2J, Supporting Information). Approximately 1000 cells were collected in each sorting session, and most cells resumed growth within 48 h, confirming their post‐sorting viability. For biomass screening, cells collected from each round of FACS sorting were inoculated into two 24‐well plates containing liquid medium. Strains exhibiting relatively higher cell density (OD values) were subsequently transferred into shake flasks for further rescreening. The mutant showing the highest OD and dry cell weight (DCW) was selected to proceed to the next round. To improve screening throughput, ten individual colonies were chosen and subjected to parallel mutagenesis and screening following the same procedure. Although certain mutants displayed inferior fermentation performance compared with the parental strain—likely attributable to the stochastic nature of mutagenesis—we consistently maintained a pool of ten colonies for each subsequent round of mutagenesis. Detailed procedures are described in the Experimental Section, and a schematic illustration of the screening workflow is provided in Figure 2C.

After six rounds of screening, ten mutants originating from the initial ten single colonies were selected and individually assessed for DCW and TFA production in the fermentation medium (Figure S2K,L, Supporting Information). All the mutants presented increased fatty acid contents, with the E5 and E6 strains showing the most significant increases (1.10‐ and 1.23‐fold, respectively), reaching 52.29 ± 1.30% and 58.54 ± 4.29% TFA content in terms of dry weight, respectively. Biomass analysis also revealed that E2, E6, E7, and E8 exhibited notable growth enhancements. On the basis of these results, strain E6, renamed ZW2, was selected as the optimal candidate for the following experiments.

We evaluated the fatty acid production potential of the *Schizochytrium* sp. ZW1 and ZW2 strains in parallel in shake flask cultures. Starting at 36 h, the biomass of ZW2 consistently surpassed that of *Schizochytrium* sp. ZW1, with increases of 1.32‐fold at 48 h and 1.27‐fold at 60 h (Figure 2D). At 48 h, the TFA and TAG contents of the ZW2 mutant reached 62.87% and 53.83%, representing 1.36‐ and 1.45‐fold increases, respectively, relative to the levels in *Schizochytrium* sp. ZW1 (Figure 2E,F). At 60 h, the TFA and TAG contents of ZW2 were 66.37% (1.40‐fold increase) and 54.38% (1.43‐fold increase), respectively (Figure 2E,F). In addition, glucose consumption analysis revealed that ZW2 exhibited a faster uptake rate than ZW1 (Figure S1C, Supporting Information). Serial passaging of the ZW2 mutant for 20 generations did not produce any significant changes in growth or fatty acid synthesis, confirming the genetic stability of the strain (Figure S1D,E, Supporting Information). Finally, in a 5‐L fermenter, ZW2 presented a log‐phase growth rate of 1.81 ± 0.03 g L^−1^ h^−1^, which was 1.26‐fold greater than that of *Schizochytrium* sp. ZW1, and reached a maximum biomass of 111.78 ± 1.46 g L^−1^, which was 1.21‐fold greater than that of *Schizochytrium* sp. ZW1 at 68 h (Figure 2G; Figure S1F, Supporting Information). TFA and TAG analysis revealed that the ZW2 mutant presented higher levels of TFA and TAG than those in the ZW1 parental strain as early as the initial logarithmic phase (28 h). By 68 h, the ZW2 mutant reached peak production, with TFA and TAG contents of 64.54 ± 0.55% and 61.14 ± 2.99%, respectively (Figure 2H; Figure S1G, Supporting Information). At 68 h, the ZW2 mutant presented 1.75‐, 1.29‐, 1.75‐, and 1.45‐fold increases in the TFA titer, yield, productivity, and content, respectively, compared to ZW1. Likewise, the TAG concentration, yield, productivity, and content in ZW2 were 1.78‐, 1.31‐, 1.78‐, and 1.47‐fold greater, respectively, than those observed in ZW1. Compared with those of other rapidly growing oleaginous chassis, the TFA titer of *Schizochytrium* ZW2 reached the same level but achieved 1.22‐, 1.96‐, and 1.13‐fold enhancements in productivity relative to those of *R*. *glutinis, Rhodosporidium toruloides*, and *Schizochytrium* ATCC20888, respectively.^[^ ^4^, ^5^, ^8^ ^]^ Collectively, these findings demonstrate that ZW2 represents a metabolically stable and high‐performance *Schizochytrium* chassis with superior fatty acid production capabilities.

### 2.3 Multiomics Analysis Reveals Novel Lipogenesis Mechanisms in *Schizochytrium* ZW2 Mutant

**Figure 3:**
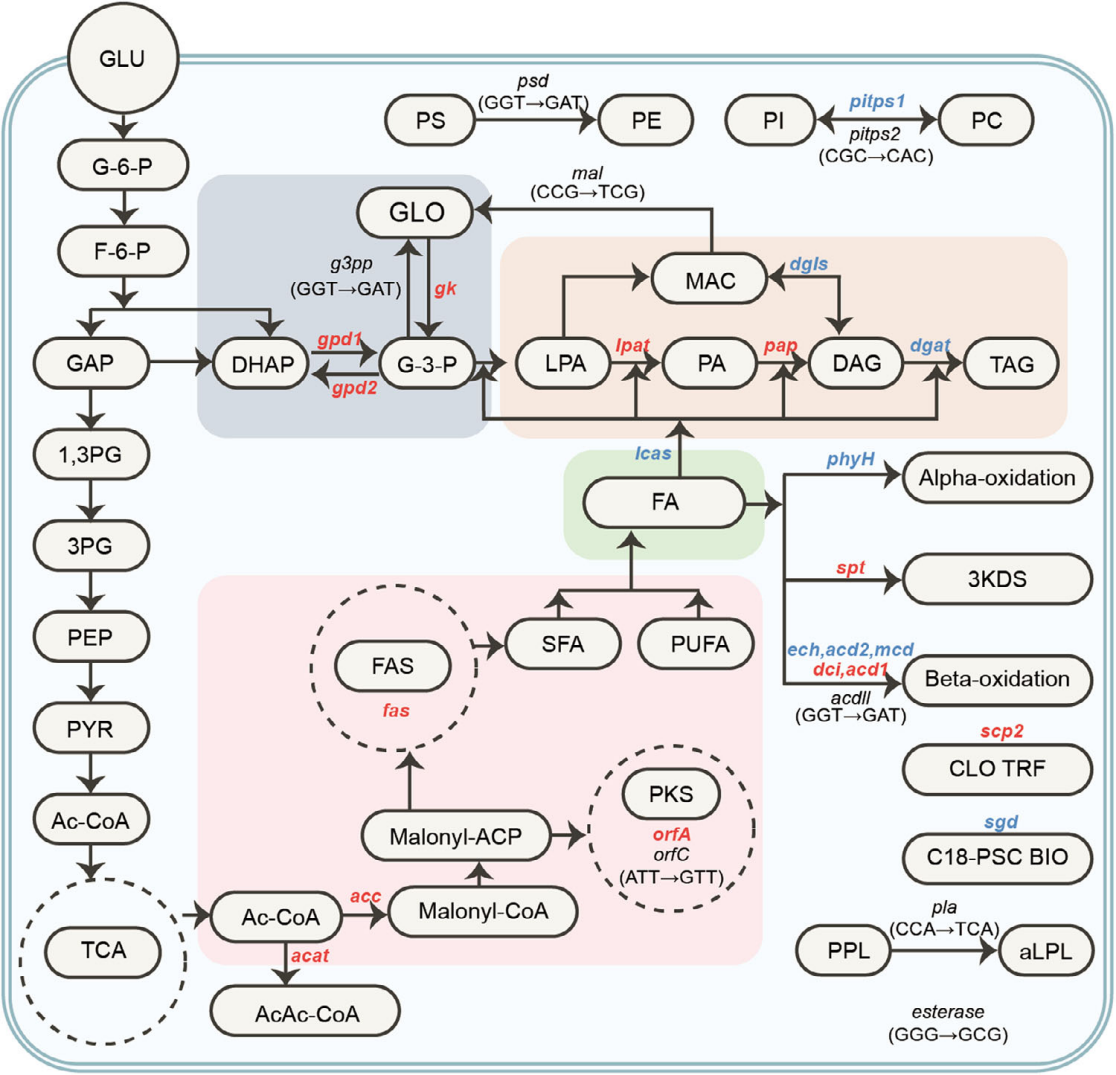
Integrated multiomics analysis to uncover the mechanisms driving enhanced fatty acid production in *Schizochytrium* sp. strain ZW2. Red, upregulated; blue, downregulated.

Given the superior fatty acid accumulation and production efficiency demonstrated by *Schizochytrium* ZW2, we further employed a comprehensive multiomics approach integrating genome resequencing, transcriptomics, and metabolomics to systematically elucidate the possible mechanisms governing its enhanced lipid biosynthesis (**Figure** **3**). The genome resequencing analysis achieved an average sequencing coverage of 78×, with the reads mapped to the reference genome of *Schizochytrium* ATCC20888, the wild‐type of the ZW1 strain. Indel analysis revealed 2 variations in coding regions and 13 variations in intergenic regions between the two strains (Tables S5‐1 and S5‐2, Supporting Information). SNP analysis revealed 559 mutations in intergenic regions, as well as 234 beneficial mutations and 140 non‐beneficial mutations in coding regions (Tables S5‐3 and S5‐4, Supporting Information). Our initial focus was on indels and beneficial SNPs in coding regions. Gene annotation and functional classification led to the identification of genes involved in lipid metabolism (Table S5‐4, Supporting Information and Figure 3). We then predicted the functional domains of these enzymes and mapped the mutation sites within their respective protein structures (Note S1, Supporting Information). We prioritized genes that are involved in both fatty acid metabolism and contain mutations within their core structural domains. Specifically, these genes include ORFC (scaffold1624.t2.cds1), monoacylglycerol lipase (MAL, scaffold18.t23.cds1), glycerol‐3‐phosphate phosphatase (G3PP, scaffold483.t2.cds1), and phosphatidylserine decarboxylase (PSD, scaffold602.t8.cds1), with each mutation independently validated by PCR and DNA sequencing.

**Figure 4:**
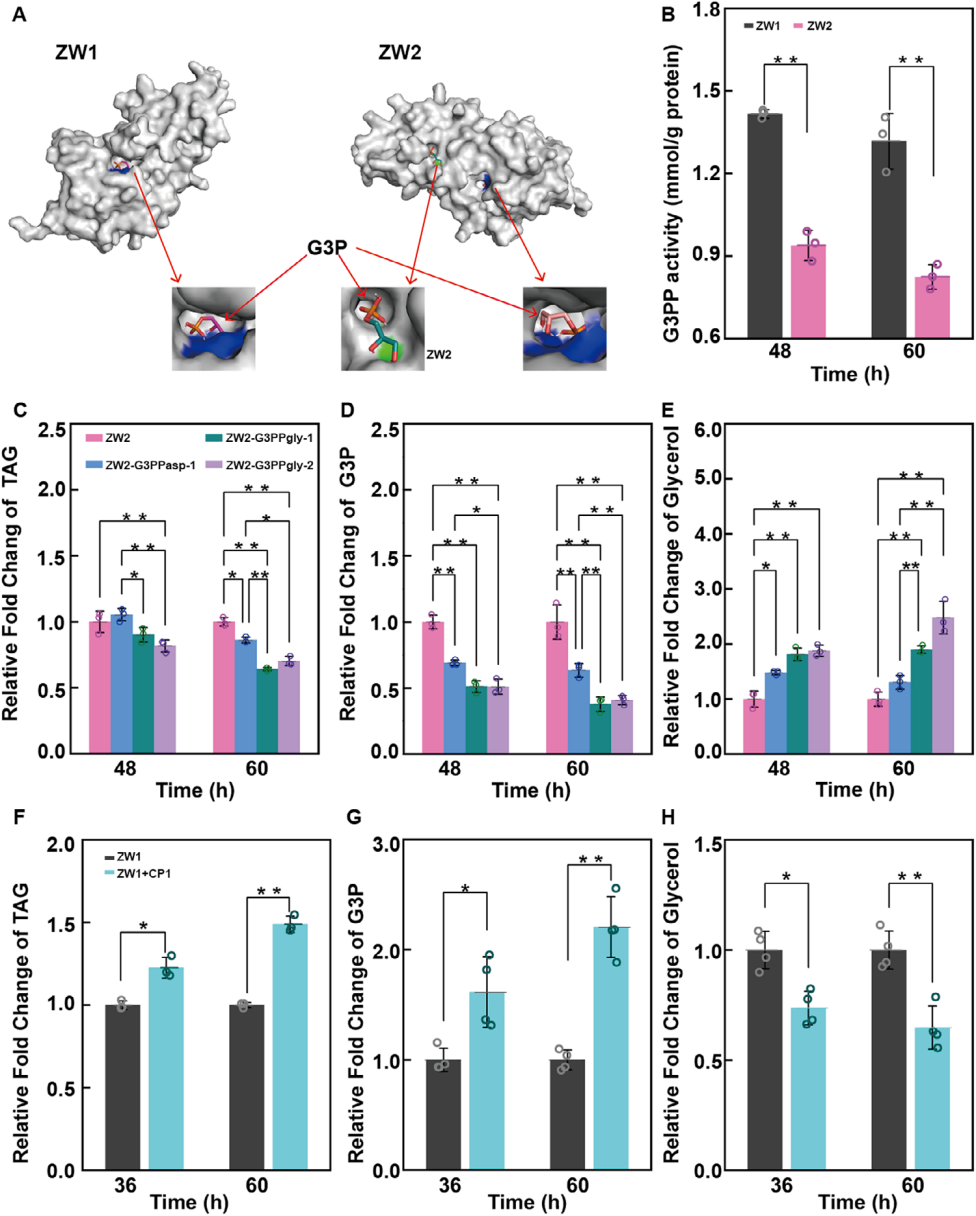
G3PP mutation enhances fatty acid biosynthesis in *Schizochytrium* sp. strain ZW2. A) Predicted G3PP structures: ZW1 (premutation, confidence 95.94; residue 147: 93.79) versus ZW2 (postmutation, confidence 96.12; residue 147: 97.96). B) G3PP activity: ZW1 (dark grey) versus ZW2 mutant (light rose). C–E) TAG (C), G3P (D), or glycerol (E) content in ZW2 (light rose) versus transformants (G3PP_asp_: blue; G3PP_gly_: green/purple). F–H) Triacylglycerol (F), G3P (G), or glycerol (H) content between *Schizochytrium* ZW1 without (dark grey) and with the CP1 inhibitor (cyan blue). Error bars represent mean ± SD. For panels B, F, G, and H, statistical analysis was performed using a two‐tailed Student's *t*‐test in Microsoft Excel, with *n* = 3–4 biologically independent samples. * *p* < 0.05, ** *p* < 0.01. For panels C, D, and E, values were first normalized to the mean of the control group, followed by one‐way ANOVA with Tukey's multiple comparison test using the Paired Comparison Plot tool in OriginPro 2021 (*n* = 3 biologically independent samples, statistical significance was defined as *p* < 0.05, * *p* < 0.05, ** *p* < 0.01).

Using AlphaFold2, we predicted the protein structures before and after mutation (**Figure** **4**A; Figure S3A,B, Supporting Information). The predicted structures and individual amino acid confidence scores fell within the confidence range, with G3PP predictions achieving high confidence. Since MAL, G3PP, and PSD have well‐defined catalytic substrates, we used AutoDock Vina to predict substrate binding modules and calculate the binding free energy and intermolecular energy for the parent and mutant proteins (**Table** **2**). These analyses showed that the binding energy and intermolecular energy decreased for both MAL and G3PP after mutation, with G3PP exhibiting a significant reduction of 1.37 kcal mol^−1^. In contrast, PSD increased the binding energy and intermolecular energy. As lower energy values indicate tighter binding, these results suggest that the mutations in MAL and G3PP may have created new substrate binding sites. Subsequent enzyme activity assays revealed that the G3PP activity of the ZW2 mutant was significantly lower than that of the ZW1 strain at 48 and 60 h (Figure 4B), whereas MAL activity was not significantly different between the two strains (Figure S3C, Supporting Information). Therefore, we focused on G3PP in our subsequent analyses. Detailed examination of the interaction bonds revealed no differences in the salt bridges between the ZW2 and ZW1 proteins, but significant differences in hydrogen bonding were observed (Table S6, Supporting Information). The mutation at position 147 in the G3PP protein of ZW2 introduced two new hydrogen bonds with the substrate that were absent in the substrate interaction with the parent protein of ZW1. Additionally, new hydrogen bonds were also formed at residues 116, 117, and 149 in the G3PP protein of ZW2.

**Table 2:** Calculation of binding and intermolecular energies between the enzyme and substrate at the selected site.

Receptor	Binding energy [kcal mol^−1^]	Intermolecular energy [kcal mol^−1^]	Residue
ZW1‐G3PP	−3.758	−6.144	147 Gly
ZW2‐G3PP	−5.125	−7.511	147 Asp
ZW1‐MAL	−8.422	−14.388	249 Pro
ZW2‐MAL	−8.843	−14.809	249 Ser
ZW1‐PSD	−12.973	−26.993	58 Gly
ZW2‐PSD	−8.045	−22.065	58 Asp

Together, these hydrogen bonds contributed to a reduced intermolecular energy in the mutant protein, indicating possible tighter substrate binding. To further validate these findings, we performed molecular dynamics simulations to track the movement of the substrate at position 147 (Videos S1 and S2, Supporting Information). The simulation analysis demonstrated that glycerol‐3‐phosphate remained tightly bound to the G3PP protein in the ZW2 mutant throughout the simulation process, whereas it dissociated from the ZW1 G3PP protein in the later stages. On the basis of these analyses, we speculate that the mutation in the G3PP protein of ZW2 has created a novel substrate‐binding site (Figure 4A), leading to reduced enzyme activity.

We obtained proteins homologous to G3PP from the NCBI database and constructed a phylogenetic tree (Figure S4A, Supporting Information). It was discovered that G3PP homologs are ubiquitously present in fast‐growing oleaginous microorganisms (*C*. *oleaginosus*, *R*. *glutini*, and *Lipomyces starkeyi*) or microalgae (*Phaeodactylum tricomutum*, *Chlamydomonas reinhardtii*, *C*. *variabilis*). Although homologs were identified in *Saccharomyces cerevisiae* and *Arabidopsis thaliana*, the evolutionary relationship between them is distant. In contrast, we detected highly homologous proteins in *Caenorhabditis elegans* and *Mus musculus*. We then performed multiple sequence alignment between the PGPH‐2 of *C. elegans*, *M. musculus* G3PP (named Mmg3pp), and the G3PP protein from *Schizochytrium* ZW2 (named Spg3pp). We found that Spg3pp presented high sequence similarity with PGPH‐2 and Mmg3pp (expected < 4 × 10^−40^, identity > 30%) and that the active motifs of each protein were fully conserved (Figure S4B, Supporting Information). PGPH‐2 and Mmg3pp hydrolyze glucose‐derived glycerol‐3‐phosphate to glycerol.^[^ ^27^, ^28^, ^29^ ^]^ To confirm that G3PP in *Schizochytrium* has similar biochemical functions, as we were unable to achieve homologous recombination in *Schizochytrium* ZW1 and ZW2 despite multiple attempts, we transformed the high‐activity G3PP_gly_ from *Schizochytrium* ZW1 into ZW2 and overexpressed a low‐activity G3PP_asp_ from strain ZW2 into ZW2 as a control. To minimize false positives potentially arising from random genomic integration, two independent biological replicate clones were isolated and validated for their genetic construct. PCR and sequencing verification confirmed successful transformation, resulting in four distinct validated transformants. These were designated as ZW2‐G3PP_asp_‐1, ZW2‐G3PP_asp_‐2, ZW2‐G3PP_gly_‐1, and ZW2‐G3PP_gly_‐2. The qRT‒PCR analysis revealed that the expression levels of the *g3pp* gene were upregulated at least 2‐fold in all four transformants compared with those in the original ZW2 mutant, with comparable expression levels observed across the four transformants (Figure S4C, Supporting Information). This ensured that subsequent phenotypic comparisons were not confounded by variations in *g3pp* expression. Then, we measured the G3PP enzyme activity in the different transformants and found that all four transformants presented greater enzyme activity than the untransformed ZW2 strain did; notably, G3PP enzyme activity was significantly greater in ZW2‐G3PP_gly_‐1 and ZW2‐G3PP_gly_‐2 than in ZW2‐G3PP_asp_‐1 and ZW2‐G3PP_asp_‐2 (Figure S4D,E, Supporting Information). We then compared the TAG, G3P, and glycerol contents in these strains. The TAG contents in ZW2‐G3PP_gly_‐1, ZW2‐G3PP_gly_‐2, ZW2‐G3PP_asp_‐1, and ZW2‐G3PP_asp_‐2 were lower than those in the ZW2 mutant. Specifically, the TAG contents of ZW2‐G3PP_gly_‐1 and ZW2‐G3PP_gly_‐2 were significantly lower than those of ZW2‐G3PP_asp_‐1 and ZW2‐G3PP_asp_‐2 (Figure 4C; Figure S4F, Supporting Information). The G3P contents in the four transformants were also significantly lower than that in the untransformed ZW2 strain, with ZW2‐G3PP_gly_‐1 and ZW2‐G3PP_gly_‐2 showing a further significant decrease compared with ZW2‐G3PP_asp_‐1 or ZW2‐G3PP_asp_‐2 (Figure 4D; Figure S4G, Supporting Information). In contrast, the glycerol content showed the opposite trend, with all four transformants exhibiting significantly higher glycerol levels than the ZW1 strain. Notably, ZW2‐G3PP_gly_‐1 and ZW2‐G3PP_gly_‐2 had significantly higher glycerol contents than ZW2‐G3PP_asp_‐1 or ZW2‐G3PP_asp_‐2 (Figure 4E; Figure S4H, Supporting Information). Further, to observe the effects of G3PP activity on triglyceride synthesis, we reduced G3PP enzyme activity in the ZW1 strain by treatment with a compound, CP1, that inhibits Mmg3pp with high selectivity and submicromolar potency.^[^ ^30^ ^]^ As expected, G3PP enzyme activity was significantly reduced at 36 and 60 h in the CP1‐treated group (Figure S4I, Supporting Information). We subsequently measured the triglyceride, G3P, and glycerol contents of the strains and found that at 36 and 60 h, the CP1‐treated strains presented significantly greater relative amounts of TAG and G3P than did the parent strain, whereas the glycerol content was significantly lower in the CP1‐treated strains (Figure 4F–H). Specifically, at 60 h, the TAG content in the CP1‐treated strains was ≈1.5 times greater than that in the untreated strains. Considering the results from both experiments, in which we detected increased G3PP enzyme activity in high‐yield strains and decreased G3PP enzyme activity in low‐yield strains, we conclude that G3PP in *Schizochytrium* functions to hydrolyze glycerol‐3‐phosphate to glycerol and that the identified point mutations are crucial for decreasing the activity of G3PP and thereby enhancing the fatty acid content in the ZW2 mutant.

**Figure 5:**
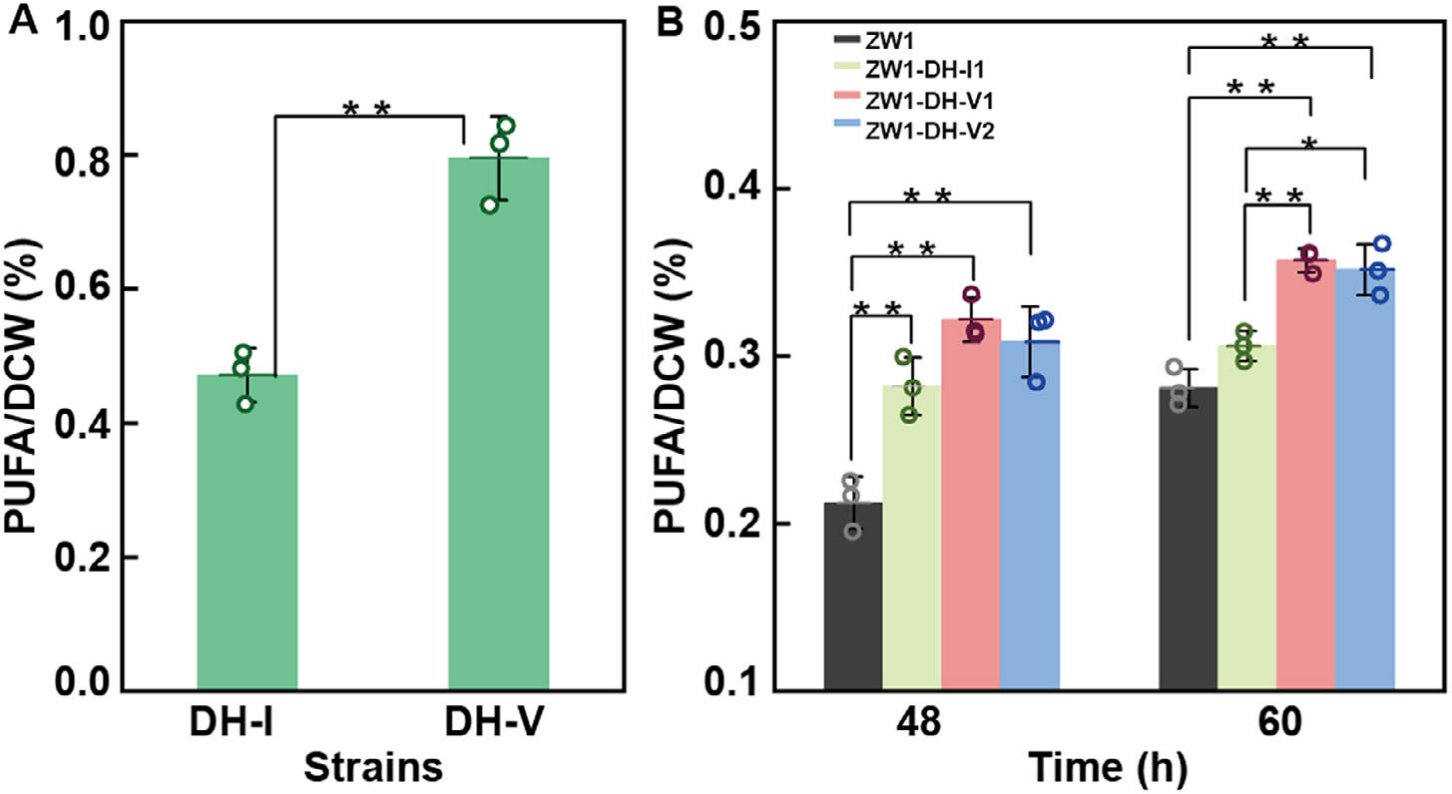
ORFC site mutation enhances PUFA accumulation in *Schizochytrium* sp. strain ZW1. A) Heterologous expression of *orfA*, *orfB*, and *orfC* in *E*. *coli*. B) PUFA content comparison: ZW1: dark grey; ZW1‐DH‐I1: Pale yellowish green; ZW1‐DH‐V1: lavender; ZW1‐DH‐V2: light blue. Error bars represent mean ± SD. For panels A, statistical analysis was performed using a two‐tailed Student's *t*‐test in Microsoft Excel, with *n* = 3 biologically independent samples. * *p* < 0.05, ** *p* < 0.01. For panels B, statistical analysis was conducted using one‐way ANOVA followed by Tukey's multiple comparison test with the Paired Comparison Plot tool in OriginPro 2021 (*n* = 3 biologically independent samples, statistical significance was defined as *p* < 0.05, * *p* < 0.05, ** *p* < 0.01).

Since the substrate of polyketide synthase subunit OrfC has not yet been clearly defined, we were unable to perform substrate‐enzyme docking or calculate the binding free energy and intermolecular interactions between the substrate and the enzyme. Consequently, we proceeded to express *Schizochytrium orfA*, *orfB*, and *orfC* directly in *E*. *coli* to investigate their catalytic potential for the production of docosahexaenoic acid (DHA) and docosapentaenoic acid (DPA). Following transformation into *E*. *coli*, we compared the contents of DHA and DPA in the presence and absence of ORFC mutation. Remarkably, the mutant strain exhibited a 1.77‐fold increase in both DHA and DPA contents (**Figure** **5**A). To further corroborate these findings, we conducted overexpression experiments in *Schizochytrium*. Given that the mutation site resides within the DH domain of ORFC, we expressed the DH‐I and DH‐V variants in *Schizochytrium* ZW1. Successful overexpression of the DH‐I and DH‐V strains was confirmed through qRT‒PCR analyses (Figure S4J, Supporting Information). These strains were subsequently cultivated in fermentation medium, and the polyunsaturated fatty acid (PUFA) content was measured at 48 and 60 h. At 48 h, the PUFA content of the starting strain was 21.25%, whereas that of the DH‐I‐overexpressing strains reached 27.96–28.19%, and that of the DH‐V strains reached 30.87–32.17%. At 60 h, the starting strain presented a PUFA content of 28.11%, whereas the PUFA contents of the DH‐I‐overexpressing strains reached 30.59–31.04%, and those of the DH‐V strains reached 35.15–35.71%. Notably, the DH‐V‐overexpressing strain presented significantly greater PUFA content than both the starting strain and the DH‐I‐overexpressing strain, especially at 60 h (Figure 5B; Figure S4K, Supporting Information). These results collectively demonstrate that the substitution of isoleucine with valine at residue 273 of ORFC enhances PUFA synthesis in *Schizochytrium*, underscoring the functional importance of this mutation in promoting PUFA production.

We then conducted RNA‐seq transcriptome analysis on log‐phase cells (36 h) of the ZW1 strain and the ZW2 mutant to identify differentially expressed transcripts related to lipid metabolism (Table S7, Supporting Information, Figure 3). The significantly upregulated transcripts included genes involved in *i*) fatty acid biosynthesis (e.g., acetyl‐CoA carboxylase, polyunsaturated fatty acid synthase subunit A, fatty acid synthase), *ii*) G3P shuttling (e.g., glycerol‐3‐phosphate dehydrogenase 1, GPD1, glycerol‐3‐phosphate dehydrogenase 2, GPD2), *iii*) glycerol degradation (e.g., glycerol kinase), *iv*) TAG biosynthesis (e.g., lysophosphatidate acyltransferase, phosphatidate phosphatase), *v*) lipid degradation (e.g., delta3,5‐delta2,4‐dienoyl‐CoA isomerase, acyl‐CoA dehydrogenase), and *vi*) other pathways (e.g., sterol carrier protein 2, serine palmitoyltransferase, acetyl‐CoA acetyltransferase). The downregulated transcripts included genes associated with *i*) triacylglycerol biosynthesis (e.g., 2‐acylglycerol O‐acyltransferase 2, *dgat*, long‐chain acyl‐CoA synthetase, *lcas*), *ii*) lipid degradation (e.g., enoyl‐CoA hydratase, phytanoyl‐CoA hydroxylase, acyl‐CoA dehydrogenase, sn1‐specific diacylglycerol lipase, short‐chain 2‐methylacyl‐CoA dehydrogenase), and *iii*) other functions (e.g., phosphatidylinositol transfer protein, sphingolipid 8‐(E)‐desaturase). We also conducted LC‒MS analysis and biochemical determination of central carbon metabolism metabolites, which revealed the upregulation of glycolytic intermediates (F6P, PEP, GAP), G3P shuttle components (DHAP, G3P), pentose phosphate pathway intermediates (R5P, E4P), cofactors (NADP, NAD, AMP), energy metabolites (ATP, ADP), TCA cycle intermediates (AKG, OXA, SUC, CIT) and fatty acid biosynthetic metabolites (Ac‐CoA), along with the downregulation of glycerol, FUM, MAL, 3‐PG and 1,3‐PG (Figure S3D–F and Table S8, Supporting Information). Taken together, these findings suggest that central carbon metabolism and fatty acid biosynthesis are enhanced in the ZW2 mutant, potentially enabling more efficient uptake and conversion of glucose into biomass and fatty acids.

**Figure 6:**
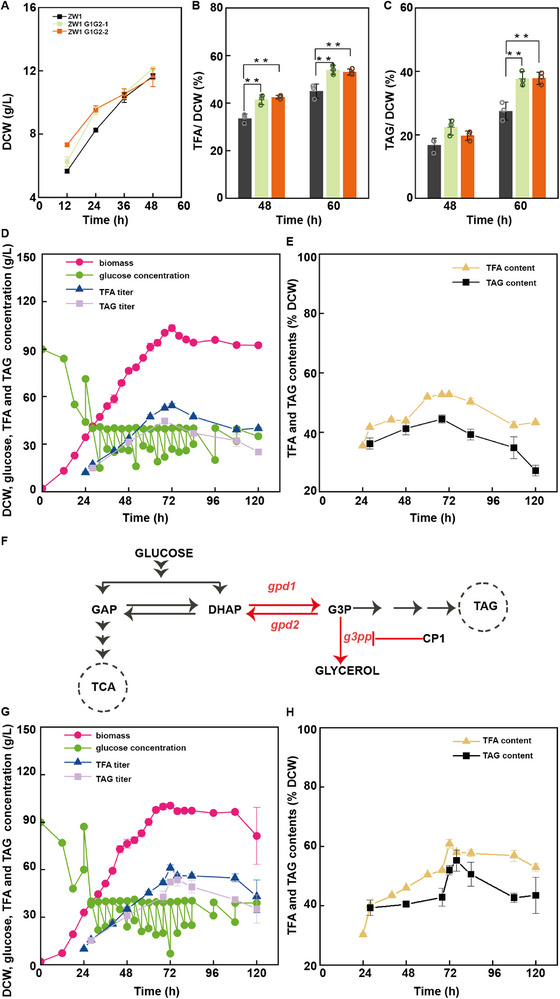
Engineering G3P flux drives biomass and fatty acid production in *Schizochytrium* sp. strain ZW1. A–C) Biomass (A), TFA (B), and TAG (C) in ZW1 (dark grey) versus *gpd1*/*gpd2* coexpressing transformants (ZW1 G1G2‐1: light green; ZW1 G1G2‐2: orange). D) Growth (pink), residual glucose titer (lime green), TFA titer (dark blue), and TAG titer (lavender) profiles of ZW1 G1G2‐1 in a 5‐L fermentation system. E) Intracellular TFA (yellow) and TAG content (black) profiles of ZW1 G1G2‐1 in a 5‐L fermentation system. F) Schematic representation of the engineering strategy used to reproduce the fermentation performance of ZW2. G) Fermentation profile of ZW1 G1G2‐1 with the CP1 inhibitor added at 56 h, showing growth (pink), residual glucose titer (lime green), TFA titer (dark blue), and triacylglycerol titer (lavender). H) TFA (yellow) and TAG (black) content profiles of ZW1 G1G2‐1 under CP1 inhibitor treatment at 56 h. Error bars represent mean ± SD. For panels A–C, statistical analysis was conducted using one‐way ANOVA followed by Tukey's multiple comparison test with the Paired Comparison Plot tool in OriginPro 2021 (*n* = 3 biologically independent samples, statistical significance was defined as *p* < 0.05, * *p* < 0.05, ** *p* < 0.01). For panels D, E, G, H, the data represent *n* = 3 technical replicates.

The upregulation of fatty acid and triacylglycerol biosynthesis genes is well known to contribute to increased fatty acid accumulation,^[^ ^19^, ^31^ ^]^ and the role of lipid degradation genes remains unclear because of their nonuniform expression patterns. We thus focused our study on the G3P shuttle genes. Transcriptomic analysis revealed that two GPD1 transcripts (CL731. Contig2_All, unchanged; CL731. Contig1_All, 2.04‐fold increase) and two GPD2 transcripts (Unigene5193_All, 2.49‐fold increase; Unigene4055_All, 1.14‐fold increase). Enzyme activity assays confirmed significantly greater GPD1 and GPD2 activity in the ZW2 mutant at 36 and 60 h than in ZW1 (Figure S5A,B, Supporting Information), indicating a possible increase in G3PP shuttle flux in cells. We also detected increased levels of G3P and DHAP metabolites in the ZW2 mutant (Figure S3D,E, Supporting Information). These results prompted us to investigate the role of increased G3P shuttle flux in improving biomass and fatty acid production in the ZW2 mutant. We first overexpressed the *gpd1* and *gpd2* genes in *Schizochytrium* ZW1 and observed that single overexpression of *gpd1* led to a significant decrease in biomass, accompanied by a significant increase in TFA content, whereas single overexpression of *gpd2* did not significantly affect biomass or TFA levels (Figure S5C–E, Supporting Information). We then simultaneously overexpressed *gpd1* and *gpd2* in *Schizochytrium* ZW1 and obtained two transformants named ZW1 G1G2‐1 and ZW1 G1G2‐2. The results revealed that both the ZW1 G1G2‐1 and ZW1 G1G2‐2 transformants presented significantly greater biomass during the early growth phase of 24–36 h, and by 60 h, the biomass of the double‐overexpressing transformants slightly exceeded that of the parental strain (**Figure** **6**A). The TFA content in the transformants was 1.18–1.26 times greater than that in the parental strain at 36 and 60 h, whereas the TAG content was 1.18–1.37 times greater (Figure 6B,C). Metabolomic analysis at 36 h revealed elevated levels of glycolytic intermediates (F6P, 3‐PG, 1,3‐PG, PEP, GAP), G3PP shuttle metabolites (DHAP, G3P), pentose phosphate pathway metabolites (R5P, E4P) and fatty acid biosynthetic metabolites (CoA, acetyl‐CoA and NADPH) (Figure S5F,G, Supporting Information), suggesting the upregulation of central carbon metabolism and fatty acid biosynthesis pathways in the double‐overexpressing transformants ZW1 G1G2‐1 and ZW1 G1G2‐2.

To further evaluate the growth and lipid production capabilities of these transformants, *Schizochytrium* ZW1 G1G2‐1 was evaluated in a 5‐L fermenter (Figure 6D,E; Figure S5H,I, Supporting Information). The logarithmic growth rate of ZW1 G1G2‐1 was 1.55 ± 0.01 g L^−1^ h^−1^, which was 1.08 times greater than that of ZW1, with a biomass of 100.22 ± 0.82 g L^−1^ at 68 h, which was 1.09 times greater than that of ZW1. The TFA content in ZW1 G1G2‐1 significantly exceeded that in ZW1 from 60 h onward, reaching 51.89 ± 0.92% at 68 h, which was 1.16 times greater than the TFA content of ZW1. Similarly, the TAG content at 68 h was 44.12 ± 1.64%, which was 1.06 times greater than that of ZW1. These results demonstrate that co‐overexpression of *gpd1* and *gpd2* enhances biomass and fatty acid accumulation in *Schizochytrium*.

### 2.4 Rational Engineering to Recapitulate the Productivity of *Schizochytrium* ZW2

As two of the three newly identified mechanisms were demonstrated to be related to the G3P shuttle cycle, we therefore combined these two mechanisms in engineered cells to determine whether this combination could achieve a fermentation profile similar to that of the ZW2 mutant (Figure 6F). *Schizochytrium* ZW1 G1G2‐1 was cultivated in a 5‐L fermenter for 56 h, followed by the addition of 100 µm CP1 inhibitor (Figure 6G,H; Figure S5J,K, Supporting Information). While the growth curve remained unchanged, the TFA content increased sharply after 72 h, reaching 60.60 ± 0.94% at 72 h and 58.48 ± 0.60% at 76 h, respectively. Similarly, the TAG content also increased significantly, reaching 51.57 ± 1.30% at 72 h and 55.32 ± 3.52% at 76 h. The highest TFA titer, yield, productivity and content of these fermentations were 85.03%, 91.67%, 80.19%, and 93.90% of the values observed in the ZW2 strain, respectively; additionally, the highest TAG concentration, yield, productivity and content were 76.38%, 86.36%, 72.00%, and 84.35% of those in the ZW2 mutant, respectively (**Table** **3**). These results suggest that the combined increase in G3P shuttle flux and decrease in G3P degradation are indeed critical factors contributing to the increased fatty acid production and biomass observed in the ZW2 mutant.

**Table 3:** Fermentation performance comparisons among *Schizochytrium* strains used in this study.

Strains	Biomass		TFA				TAG			
	Titer [g L^−1^]	Productivity [g L^−1^ h^−1^]	Titer [g L^−1^]	Yield [g g^−1^]	Productivity [g L^−1^ h^−1^]	Content [%]	Titer [g L^−1^]	Yield [g g^−1^]	Productivity [g L^−1^ h^−1^]	Content [%]
ZW1	92.18 ± 2.01	1.36 ± 0.03	41.15 ± 1.58	0.18 ± 0.01	0.61 ± 0.02	44.64 ± 0.98	38.40 ± 3.92	0.17 ± 0.02	0.56 ± 0.06	41.61 ± 3.60
ZW1 G1G2‐1	100.22 ± 0.82	1.47 ± 0.01	52.01 ± 1.11	0.21 ± 0.01	0.76 ± 0.02	51.89 ± 0.92	44.22 ± 1.72	0.18 ± 0.01	0.65 ± 0.03	44.12 ± 1.64
ZW1 G1G2‐1‐CP1	101.21 ± 1.32	1.47 ± 0.03	61.34 ± 1.22	0.22 ± 0.01	0.85 ± 0.02	60.60 ± 0.94	52.19 ± 1.15	0.19 ± 0.01	0.72 ± 0.02	51.57 ± 1.30
ZW2	111.78 ± 1.46	1.64 ± 0.02	72.14 ± 1.38	0.24 ± 0.01	1.06 ± 0.02	64.54 ± 0.55	68.33 ± 3.14	0.22 ± 0.01	1.00 ± 0.05	61.14 ± 2.99
ZW2‐D‐L2	122.64 ± 5.05	1.80 ± 0.07	94.65 ± 1.29	0.26 ± 0.02	1.39 ± 0.02	77.26 ± 2.39	88.51 ± 5.38	0.25 ± 0.02	1.30 ± 0.08	72.22 ± 4.28

### 2.5 Engineering ZW2 to Achieve Higher Fatty Acid Productivity

**Figure 7:**
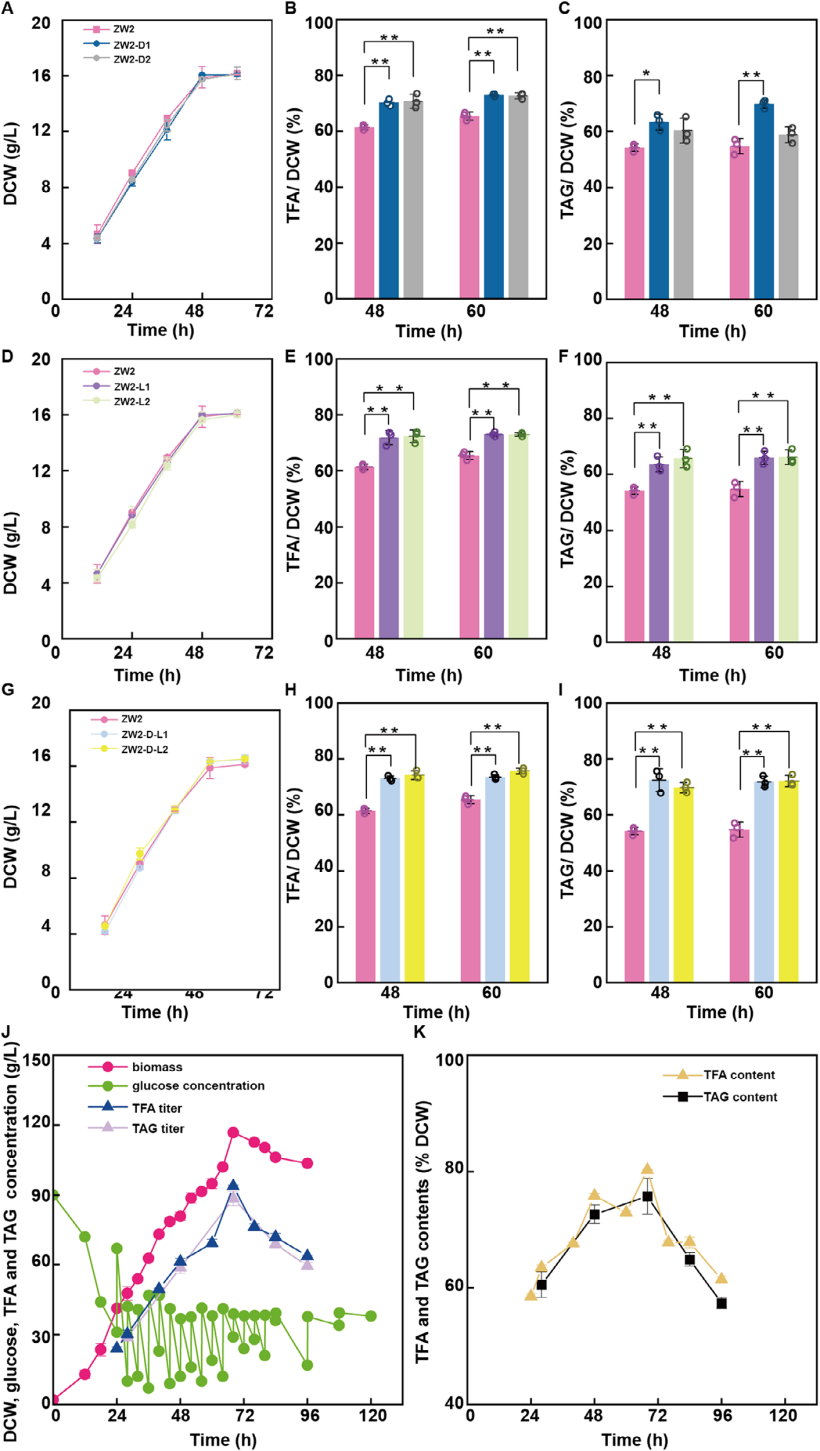
Increased fatty acid production in *Schizochytrium* ZW2. A–C) Biomass (A), TFA (B), and TAG (C) in ZW2 (light rose) versus ZW2‐D1 (blue) and ZW2‐D2 (gray). D–F) Biomass (D), TFA (E), and TAG (F) in ZW2 (light rose) versus ZW2‐L1 (violet) and ZW2‐L2 (mint green). G–I) Biomass (G), TFA (H), and TAG (I) in ZW2 (light rose) versus ZW2‐D‐L1 (silver) and ZW2‐D‐L2 (yellow). J) 5‐L fermentation of *Schizochytrium* ZW2‐D‐L2. Growth curve (pink), residual glucose titer (lime green), TFA titer (dark blue), and triacylglycerol titer (lavender). K) TFA (yellow) and TAG (black) content profiles of *Schizochytrium* ZW2‐D‐L2 in a 5‐L fermentation system. Error bars represent mean ± SD. For panels A–I, statistical analysis was conducted using one‐way ANOVA followed by Tukey's multiple comparison test with the Paired Comparison Plot tool in OriginPro 2021 (*n* = 3 biologically independent samples, statistical significance was defined as *p* < 0.05, * *p* < 0.05, ** *p* < 0.01). For panels J,K, the data represent *n* = 3 technical replicates.

Transcriptomic analysis revealed significant downregulation of two TAG biosynthetic genes, *dgat* and *lcas*, in the ZW2 mutant. To increase fatty acid titer and productivity, we overexpressed these genes in the ZW2 mutant individually and in combination, and these transformants were designated ZW2‐D‐1, ZW2‐D‐2, ZW2‐L‐1, ZW2‐L‐2, ZW2‐D‐L‐1, and ZW2‐D‐L‐2 (Figure S6A,B, Supporting Information). Growth curve analysis revealed no significant differences in biomass between the transformants and the parental strain (**Figure** **7**A,D,G). Fatty acid analysis revealed that at 48 h, the TFA content in ZW2 was 61.46%, whereas the TFA contents in the single‐overexpression strains ZW2‐L1 and ZW2‐L2 were 71.80% and 72.30%, respectively, and those in ZW2‐D1 and ZW2‐D2 were 70.25% and 70.78%, respectively. The double‐overexpression strains ZW2‐D‐L1 and ZW2‐D‐L2 presented even higher TFA contents, of 73.04% and 74.20%, respectively. At 60 h, the TFA content in ZW2 was 65.43%, while single‐overexpression strains maintained TFA contents of ≈73%. The double‐overexpression strains showed a slight increase in TFA content, with ZW2‐D‐L1 and ZW2‐D‐L2 reaching 73.55% and 75.72%, respectively (Figure 7B,E,H). Similarly, analysis at 48 h revealed TAG content of 54.20% in the ZW2 mutant, whereas the single‐overexpression strains presented TAG contents ranging from 60.32% to 65.68%; the double‐overexpressing strains presented significantly higher TAG levels, with ZW2‐D‐L1 and ZW2‐D‐L2 reaching 72.45% and 69.75%, respectively. At 60 h, the TAG content was 54.74% in ZW2, 58.82–69.83% in the single‐overexpression strains, and reached 71.83% and 72.15% in the double‐overexpression strains ZW2‐D‐L1 and ZW2‐D‐L2, respectively (Figure 7C,F,I). Taken together, these results demonstrate that the overexpression of *dgat* and *lcas* significantly increased TFA and TAG accumulation in the ZW2 mutant without any negative effects on growth, suggesting a synergistic effect of these genes in the TAG biosynthesis pathway.

As ZW2‐D‐L1 and ZW2‐D‐L2 exhibited similar phenotypes in the flask fermentation, ZW2‐D‐L2 was selected for further evaluation in a 5‐L fermenter (Figure 7J,K; Figure S6E–H, Supporting Information). The transformant rapidly accumulated biomass after 12 h of logarithmic growth, reaching a peak biomass of 122.64 ± 5.05 g L^−1^ at 68 h, ≈1.10 times greater than that of ZW2. The logarithmic growth rate was 1.96 ± 0.09 g L^−1^ h^−1^, which was 1.08 times greater than that of ZW2. The fatty acid content was measured. At 24 h, the TFA content in ZW2‐D‐L2 was 54.55 ± 3.14%, which was significantly (1.36 times) greater than that in ZW2. TFA accumulation in ZW2‐D‐L2 peaked at 68 h, reaching 77.26 ± 2.39%, which was 1.20 times greater than that in ZW2, before decreasing at 76 h, likely due to cell lysis. The TAG content in ZW2‐D‐L2 peaked at 68 h, reaching 72.22 ± 4.28% and representing a value 1.18 times greater than that in the control strain ZW2. These findings demonstrated that further genetic modifications of the identified target genes could significantly increase lipid production in the ZW2 mutant.

To evaluate the genetic and phenotypic stability of the engineered strain ZW2‐D‐L2, the strain was subjected to the consecutive passages under non‐selective conditions. Throughout this process, the expression levels of the two target genes—*dgat* and *lcas*—remained consistently upregulated by more than twofold, and the resistance marker gene was persistently detected (Figure S7A,B, Supporting Information). Furthermore, after ten passages, no significant differences were observed in cell growth, TFA content, or TAG accumulation compared to the pre‐passaged culture (Figure S7C,D, Supporting Information). These findings demonstrate that ZW2‐D‐L2 exhibits robust genetic and phenotypic stability, highlighting its potential suitability for large‐scale industrial applications.

We next compared the fermentation performance of the ZW1 and ZW2‐D‐L2 strains. The TFA concentration, yield, and productivity of ZW2‐D‐L2 were enhanced 2.30‐, 1.44‐, 2.28‐, and 1.73‐fold, respectively, compared to those of the ZW1 strain (Table 3). Similarly, the TAG concentration, yield, and productivity in ZW2‐D‐L2 were 2.30, 1.47, 2.32, and 1.74 times greater than those in the ZW1 strain (Table 3). To visualize TAG accumulation, we performed BODIPY 505/515 staining on the ZW1 and ZW2‐D‐L2 strains at 68 h. In the ZW1 strain, TAG occupied only a small fraction of the cell volume (Figure S6C, Supporting Information), whereas in the ZW2‐D‐L2 strain, TAG filled nearly the entire cell space, accompanied by a significant increase in cell size compared to ZW1 (Figure S6D, Supporting Information).

Further, we compared the fermentation performance of ZW2‐D‐L2 strains to that of current widely used oil‐producing chassis strains under optimal fermentation conditions (Table 1). Since previous studies have reported data for fatty acid methyl ester (FAME),^[^ ^3^, ^4^ ^]^ we calculated the FAME content from the fatty acids produced by the ZW2‐D‐L2 strain at 68 h as 99.40 ± 1.36 g L^−1^, corresponding to a yield of 0.27 ± 0.01 g g^−1^ and a productivity of 1.46 ± 0.02 g L^−1^ h^−1^. Compared with the previously reported optimal fermentation of *Schizochytrium*, the FAME concentration in our ZW2‐D‐L2 strain was 1.17 times greater, and the productivity was 1.49 times greater.^[^ ^4^ ^]^ Compared with the highest reported FAME production in *Y. lipolytica*,^[^ ^3^ ^]^ the concentration and yield in ZW2‐D‐L2 were similar, but the productivity was 1.12 times greater. Compared with the highest TAG production reported in *R. opacus* PD630,^[^ ^6^ ^]^ the ZW2‐D‐L2 strain presented a 1.07‐fold greater TAG concentration and 1.51‐fold greater productivity. Finally, our fermentation process was completed in 68 h, which was 19, 28, and 16 h faster than the optimal production times required for *Schizochytrium* ATCC20888, *R*. *opacus* PD630, and *Y. lipolytica*, respectively.^[^ ^3^, ^4^, ^6^ ^]^

## 3 Discussion

Despite increasing interest in microbial fatty acid biosynthesis, low titer and productivity remain a critical barrier to its industrial scalability. To address this issue, we employed the heterotrophic microalga *Schizochytrium* ZW1, exhibiting rapid growth, high glucose assimilation, and efficient lipid biosynthesis, as a new chassis. Through an innovative screening strategy, we generated and isolated the mutant ZW2. Integrated multi‐omics analyses uncovered the mechanisms underlying the increased fatty acid titer and productivity in ZW2, enabling the recapitulation of ≈80% of its fermentation phenotype via rational engineering on the basis of the identified mechanisms. Finally, targeted metabolic engineering was applied to *Schizochytrium* ZW2, resulting in the titers and productivities of TFA and TAG that exceed all previously reported values in microbial systems.

A high‐density fermentation system is a critical prerequisite for this work. Therefore, we benchmarked our fermentation parameters against those of other high‐performance chassis at the laboratory scale. A notable distinction between our fermentation strategy and that established for *Y*. *lipolytica* lies in the rational design of nutrient supply.^[^ ^3^ ^]^ Specifically, while the *Y*. *lipolytica* process depends on a high inorganic nitrogen concentration (8.8 g L^−1^ (NH_4_)_2_ SO_4_ versus only 2.5 g L^−1^ yeast extract), we formulated our medium with yeast extract as the main nitrogen source. Furthermore, a critical operational divergence is evident in the feeding strategy: where *Y*. *lipolytica* employs continuous, fixed‐rate glucose feeding,^[^ ^3^ ^]^ our system uses a pulse‐feeding strategy simultaneously supplies glucose and yeast extract. By dynamically adjusting nitrogen supplementation based on consumed glucose, our strategy aims to regulate the carbon‐to‐nitrogen balance, avoiding both nitrogen starvation—which would arrest growth—and nitrogen excess—which represses lipid accumulation. Similarly, compared to the *R*. *opacus* PD630 protocol,^[^ ^6^ ^]^ which relies on a minimal defined medium with (NH_4_)_2_SO_4_ as the sole nitrogen source. Moreover, the most striking operational contrast lies in pH management: *R*. *opacus* PD630 utilizes a two‐stage pH‐shift strategy—starting low to shorten lag phase and then elevating to promote biomass.^[^ ^6^ ^]^ In contrast, our results indicate that sustained pH stability at 6.5 ± 0.1 proved optimal, underscoring that a constant neutral pH better supports concurrent biomass and lipid production in our system. Together, these comparisons highlight that optimal high‐density fermentation conditions are organism‐specific and depend on finely tuned nutrient and operational strategies to balance growth and product synthesis.

Uncovering novel regulatory mechanisms to reconstruct complex metabolic pathways remains a central goal in advancing molecular engineering strategies for microalgae.^[^ ^20^ ^]^ G3P is recognized as a critical metabolic node that links energy metabolism, TAG synthesis, and central carbon metabolism,^[^ ^22^ ^]^ however, G3PP enzyme has largely been overlooked in early studies with oleaginous microbe. possibly due to the absence of identifiable homologs. It was reported that no homologous gene has been detected in *Y*. *lipolytica*.^[^ ^32^ ^]^ Nonetheless, our multiple sequence alignment revealed that G3PP proteins share high similarity with homologs in *R. graminis* WP1, *Lipomyces starkeyi* NRRL Y‐11557, and *C. oleaginosus* IBC0246 (Expect < 1e^−30^; Identities > 30%). This suggests that comparable mechanisms might also be present in these species. Although homologs exist in oleaginous microalgae such as *Chlamydomonas* and *Dunaliella*, studies indicate that the NAD⁺‐dependent glycerol‐3‐phosphate dehydrogenase is often associated with a phosphoserine phosphatase domain, which executes a G3PP‐like hydrolytic function.^[^ ^33^, ^34^, ^35^ ^]^ This suggests that the strategy of enhancing TAG synthesis by suppressing G3PP activity might not be applicable to these species. Beyond microbes, G3PP has gained increasing attention in *M. musculus* and *C. elegans* and is now considered a potential therapeutic target for lipogenesis‐related diseases.^[^ ^27^, ^28^, ^36^, ^37^ ^]^ *Schizochytrium* GP3PP exhibits significant sequence homology, conserved catalytic sites, and functional parallels with *M*. *musculus* Mm G3PP and *C*. *elegans* PGPH‐2. Based on comparative analysis of corresponding amino acid residues among these homologous proteins,^[^ ^30^ ^]^ we speculate that this mutation enhances the hydrogen bonding between glycerol‐3‐phosphate and aspartate, along with the surrounding amino acids. These factors impede substrate entry into the active site, leading to significantly reduced enzymatic activity. These discoveries also offer new insights into the modulation of G3PP for therapeutic applications in type 2 diabetes, obesity, and age‐related metabolic disorders.

Previous studies have focused predominantly on increasing G3P levels through GPD1 overexpression to increase lipid accumulation;^[^ ^32^, ^38^, ^39^ ^]^ however, our investigation revealed an intrinsic physiological conflict: although GPD1 overexpression effectively stimulated fatty acid biosynthesis, it simultaneously triggered growth retardation, which is detrimental. These compensatory effects ultimately negated the potential improvements in lipid titer and production efficiency. In contrast to prior approaches, our dual‐modulation strategy achieved carbon flux optimization through *i*) GDP2 coexpression to redirect partial G3P into glycolysis pathways, thereby restoring cell growth, and *ii*) the targeted inhibition of G3P hydrolysis to channel metabolic flux toward TAG synthesis. This study represents the first systematic exploration of the G3P shuttle as a molecular target for metabolic engineering, addressing the challenge of balancing cellular growth and fatty acid biosynthesis through carbon flux redistribution.^[^ ^40^ ^]^ Importantly, the combined role of the G3P shuttle cycle and the identified glycerol‐3‐phosphate phosphatase mutation was sufficient to reproduce ≈80% of the mutant phenotype, suggesting that we have uncovered a central mutational mechanism, meanwhile, we also acknowledge that certain proteins with mutations outside their core domains may play a critical role in fatty acid synthesis, and we have a plan to examine their functional relevance in future work.

The DH domain was previously reported to play a critical role in determining the specificity and titer of PUFA profiles.^[^ ^41^ ^]^ Sequence comparison and structural modeling of the *Schizochytrium* DH domain revealed that the mutation site is located relatively far from the catalytic center but close to the substrate‐binding site.^[^ ^42^ ^]^ We hypothesize that the mutation of isoleucine to valine at position 71 may reduce steric hindrance, thus facilitating increased substrate binding and consequently increased PUFA synthesis. A similar mechanism has also been observed in human carbonic anhydrase and in beta‐galactosidase from Antarctic extremophiles.^[^ ^43^, ^44^ ^]^ Future studies should be conducted to clarify the acyl‐ACP substrates of *Schizochytrium* DH and identify its catalytic and substrate‐interacting residues to enable further mechanistic analysis and rational design.

Our engineering strategy also differs substantially from those applied in other chassis organisms. In *Y*. *lipolytica*, redox engineering was leveraged to maximize electron capture, resulting in the highest reported titer of FAME;^[^ ^3^ ^]^ in *R. opacus*, optimizing fermentation parameters such as pH control, led to the highest TAG production.^[^ ^6^ ^]^ By contrast, our study adopted a comprehensive strategy that integrates fermentation optimization, random mutagenesis with high‐throughput screening, multi‐omics analysis, and target‐based genetic engineering. Beyond achieving high titer and productivity, this multi‐faceted approach offers several distinct advantages. First, it provides a systematic framework that incorporates not only classical fermentation optimization and evolutionary techniques but also enables the elucidation of previously unknown regulatory mechanisms and functional targets. Second, it introduced critical technical modifications to conventional FACS‐based screening to challenges associated with cellular viscosity, thereby significantly enhancing screening efficiency and mutant reliability. Third, it provides novel mechanistic insights into the conventional G3PP shuttle cycle and discovered physiological function of G3PP in TAG biosynthesis and novel mutational sites regulating its enzymatic activity. These insights not only advance lipid production capabilities but also expand the functional understanding of G3PP in the context of lipogenesis‐related diseases. Altogether, this strategy may also provide a promising solution for engineering other microalgae with glucose as a substrate, such as *C*. *variabilis* and *Scenedesmus acuminatus* ^[^ ^18^, ^45^ ^]^ and in other fast‐growing chassis systems, such as *C*. *oleaginosus* and *R*. *glutinis*.^[^ ^5^, ^7^ ^]^

The *Schizochytrium* ZW2‐D‐L2 engineered in this study offers unique advantages for advancing the industrialiazation of microbial fatty acid production: *i*) it significantly reduces the unit cost of manufacturing. Based on the techno‐economic model established in literature,^[^ ^13^ ^]^ microbial oil production costs were calculated for our engineered strain, *Y*. *lipolytica*, and *R*. *opacus* PD630.^[^ ^3^, ^6^ ^]^ The analysis demonstrated that producing 10,000 tons of FAME requires M$77.05 with *Y*. *lipolytica* versus M$68.74 million for our engineered strain, representing a 10.79% cost reduction. Similarly, TAG production costs total M$ 90.04 million for *R*. *opacus* PD630 compared to M$74.78 for our strain, achieving a 16.95% reduction (Note S2, Supporting Information). *ii*) it enhances overall economic viability. The strain engineered in this study produces high‐value PUFAs as ≈50% of its TFA content. These high‐value metabolites (e.g., DHA and EPA) compensate for the relatively low market value of biodiesel‐grade fatty acids. *iii*) it increases production efficiency. The strain engineered in this study increases overall fatty acid production by 23–41% through cycle time compression, improving equipment utilization efficiency and production line throughput.^[^ ^46^ ^]^ This efficiency operational advantage significantly boosts potential return on investment, particularly for commercial projects with a projected payback period. Finally, the research paradigm proposed here—leveraging native traits, tool development, regulatory elucidation, and metabolic rewiring—can be applied to diverse microalgal species to expand chassis options and accelerate industrial deployment.

## 4 Conclusion

In this study, a high‐performance fatty acid‐producing strain was developed through random mutagenesis coupled with FACS‐based screening. Subsequent multi‐omics analyses elucidated key mechanisms underlying enhanced lipid biosynthesis and identified novel engineering targets. The resulting engineered strain achieved TFA and TAG titers of 94.65 and 88.51 g L^−1^, with productivities of 1.39 and 1.30 g L^−1^ h^−1^, respectively—representing a twofold increase over the parent strain and surpassing the performance of all reported microbial systems to date. This work not only provides a breakthrough solution for microbial fatty acid production but also establishes a “strain optimization‐mechanistic elucidation‐genetic engineering” paradigm that advances microalgae‐based industrial biotechnology. While representing a promising starting point, future efforts should focus on expanding the genetic toolkit, utilizing low‐cost carbon sources (e.g., lignocellulosic hydrolysates), and ensuring long‐term industrial stability to further enable sustainable biomanufacturing.

## 5 Experimental Section

### Strains, Plasmids, and Cultivation Conditions

All strains were derived from *Schizochytrium* ATCC20888. *Schizochytrium* transformation was performed using the electroporation method, and 200 µg mL^−1^ G418 was added to the media for transformant screening.^[^ ^47^ ^]^ The G3PPasp denotes the G3PP protein with Aspartic acid at position 147, while G3PPgly refers to the variant with Glycine at the same site. Similarly, DH‐I indicates the DH protein with Isoleucine at residue 273, and DH‐V designates the Valine variant at this position. The engineering strategies for generating these recombinant strains, along with the corresponding parental strains, are summarized in Table S1 (Supporting Information). The plasmids used for gene expression were constructed using a NovoRec Plus One‐step PCR Cloning Kit. For *SsorfA*, *SsorfB*, and *SsorfC* expression, three genes were amplified from the *Schizochytrium* genome. The 4′‐phosphopantetheinyl transferase (*hetI*, GenBank accession number L22883) gene from *Nostoc* sp. PCC 7120 was synthesized by GENEWIZ. These genes were subsequently cloned and inserted into plasmids and introduced into *E*. *coli* BL21(DE3) via electroporation. The plasmids and primers used in this study are listed in Tables S2 and S3 (Supporting Information), respectively.

### Shake‐Flask Cultivation

The *Schizochytrium* strains were initially cultured in minimal medium (5 g L^−1^ glucose, 1 g L^−1^ peptone, 1 g L^−1^ yeast extract, and 20 g L^−1^ sea salt) for 48 h at 28 °C with agitation at 200 rpm and then transferred to fermentation medium for an additional 48 h to serve as seed cultures. The composition of the fermentation medium had been described previously.^[^ ^48^ ^]^ ≈1 OD_660_ of these seed cultures was subsequently inoculated into fermentation media for further analysis.

For PUFA expression, the plasmid‐harboring *E*. *coli* was cultured in 765 medium (KH_2_PO_4_ 4.49 g L^−1^, K_2_HPO_4_·3H_2_O 15.29 g L^−1^, (NH_4_)_2_SO_4_ 1.98 g L^−1^, FeSO_4_·7H_2_O 0.5 mg L^−1^, MgSO_4_·7H_2_O 0.25 g L^−1^, casamino acids 11 g L^−1^, glycerol 100 g L^−1^) with the appropriate antibiotics (100 µg mL^−1^ ampicillin, 50 µg mL^−1^ kanamycin, or 50 µg mL^−1^ chloramphenicol) to an OD_600_ of 0.5–0.8, after which 0.5 mM IPTG was added. The cultures were incubated for 60 h at 20 °C and 200 rpm, after which the cells were harvested and freeze‐dried, and ≈100 mg of dry cell weight was subjected to fatty acid analysis.

### Fed‐Batch Fermentation

Fed‐batch cultures were carried out in a 5‐L fermenter containing 2.5 L of medium (glucose 100 g L^−1^, yeast extract 25 g L^−1^, K_2_SO_4_ 7 g L^−1^, MgSO_4_·7H_2_O 10 g L^−1^, KCl 2 g L^−1^, K_2_HPO_4_ 3.93 g L^−1^, (NH_4_)_2_SO_4_ 1 g L^−1^, Na_2_SO_4_ 12 g L^−1^, 0.58 mg L^−1^ FeSO_4_·7H_2_O, 5.2 mg L^−1^ MnCl_2_·4H_2_O, 5.2 mg L^−1^ ZnSO_4_·7H_2_O, 0.8 mg L^−1^ CuSO_4_·5H_2_O, 0.8 mg L^−1^ NiSO_4_·6H_2_O, 0.066 mg L^−1^ CoCl_2_·6H_2_O, 0.152 mg L^−1^ vitamin B1, 0.024 mg L^−1^ vitamin B12, 0.512 mg L^−1^ C_18_H_32_CaN_2_O_10_, 0.016 g L^−1^ NaMoO_4_·2H_2_O). Seed cultures were grown in 250 mL Erlenmeyer flasks for 48 h. ≈2 g L^−1^ of dry cell weight was used to inoculate the 5‐L fermenter. The fermentation temperature was maintained at 28 °C. The pH was maintained at 6.5 ± 0.1. Antifoam SE‐15 was added as needed to control foam formation. The agitation speed was set to 200 rpm for 0–12 h, 500 rpm for 12–32 h, 550 rpm for 32–52 h, and 650 rpm from 55 h until the end of fermentation, with a constant air flow rate of 1 *vvm*. A fed‐batch strategy was implemented to maintain high cell density. Glucose supplementation (600 g L^−1^ solution) was triggered when the residual concentration fell below 20 g L^−1^, raising it to 40 g L^−1^. Yeast extract (50%, *w*/*v*) was then added proportionally based on the consumed glucose to maintain a C/N ratio of 4:1 (0–40 h) or 7:1 (after 40 h) in the feeding solutions. A CP1 inhibitor was added to the cultures at 56 h when necessary.

### Analytical Procedure

Cell growth was monitored by measuring the DCW. To determine the DCW, 2 mL of culture broth was centrifuged at 13 000 rpm for 15 min, washed twice with deionized water, freeze‐dried, and then weighed. The glucose concentration was measured using a glucose oxidase assay kit.

The fatty acid content was determined using a previously reported method.^[^ ^49^ ^]^ namely, calculated using the standard curve method with nonadecanoic acid as an internal standard (Table S4, Supporting Information). For TAG determination, the lipid content (M_lipid_) was determined via the gravimetric method,^[^ ^50^ ^]^ and lipids dissolved in chloroform (20 µg µL^−1^, 100 µL) were separated on silica gel 60 F254 TLC plates (Merck) using hexane:diethyl ether:acetic acid (70:30:1). Bands visualized via 10% (*w*/*v*) CuSO_4_·5H_2_O/8.0% (*v*/*v*) H_3_PO_4_ charring at 180 °C were excised, chloroform‐extracted, esterified, and analyzed via GC‒MS. TAG recovery efficiency (E) was calculated by comparing the peak areas of the standard (Sigma‒Aldrich) before and after TLC separation. The total TAG content (M_tag_) was calculated as M_tag_ = ∑Mn/E× (Mrn + 12 + 16 – 18 + 5/3)/Mrn, where n represents each fatty acid. The TAG content in the algal powder was calculated as M_tag_/2×M_lipid_.

G3P was converted to H_2_O_2_ via glycerol‐3‐phosphate oxidase and reacted with Amplex Red to produce resorufin, which was then quantified by spectrometry (OD_570_). Glycerol‐3‐phosphate oxidase and Amplex Red were provided within the Amplex Red Glycerol Assay Kit. Lysates of log‐phase cells were prepared and collected for protein quantification via the Bradford method. The lysates were heated at 95 °C for 5 min to inactivate glycerol kinase activity since G3P was stable at 95 °C for 20 min.^[^ ^51^ ^]^ The content of G3P was analyzed against a G3P standard curve (Table S4, Supporting Information) and normalized to the protein content.

Glycerol was quantified using the Amplex Red Glycerol Assay Kit. Cell extracts of log‐phase cells were prepared for protein quantification via the Bradford method. Then, catalase and glycerol‐3‐phosphate oxidase were added, and the mixture was incubated at 37 °C for 1 h to eliminate G3P interference.^[^ ^51^ ^]^ The glycerol content was assayed against a glycerol curve (Table S4, Supporting Information) and normalized to the protein concentration.

Cell observation of *Schizochytrium* ZW1 was conducted using an Olympus BX43 microscope at 1,000× magnification. To visualize lipid droplets, cells were stained with BODIPY 505/515 (100 µg mL^−1^; 20 µL per 1 mL of cell suspension) for 10 min in the dark, followed by washing. The stained lipid droplets were then observed under a Leica Stellaris8 confocal microscope at 630× magnification, using an excitation wavelength of 480 nm and an emission wavelength of 507 nm.

### Enzyme Assays

For cell extract preparation, the cells (30 OD_660_ units) were lysed with zirconium oxide beads (vortexing with ice‐cooling), and centrifuged (4 °C, 14 000 rpm, 1 h), and the supernatants were assayed for protein determination via the Bradford method. A glycerol‐3‐phosphate phosphatase assay was performed as described previously.^[^ ^52^ ^]^ Briefly, the reaction mixture (200 µL: 2.5 mm MgCl_2_, 2.9 mm G3P, Tris‐HCl pH 7.0, 100 µL extract) was incubated at 37 °C for 15 min. The released phosphate was quantified using a Total Phosphorus Content Assay Kit and normalized to the protein concentration. Monoacylglycerol lipase activity was assayed using a previously reported method.^[^ ^53^ ^]^ Briefly, the reaction mixture (1 mL: 240 µm arachidonoyl‐1‐thio‐glycerol, Tris‐HCl pH 7.0, 100 µL extract) was incubated at 37 °C for 5 min; then, 10 µL of 100 mm 5,5′‐dithiobis‐2‐nitrobenzoic acid was added, and the absorbance at 412 nm was measured. The monoacylglycerol lipase activity was normalized to the protein concentration. GPD1 activity was determined by monitoring the reduction of DHAP by NADH, which was measured as the change in absorbance at 340 nm as assayed using a spectrophotometer.^[^ ^54^ ^]^ The 250 µL reaction mixture contained 20 mm imidazole‐HCl, 1 mm dithioerythritol, 10 mm MgCl_2_, 11.57 mm DHAP, 0.11 mm NADH, and 10 µL of cell extract. The mixture was incubated at 37 °C for 5 min, after which the absorbance at 340 nm was measured. As a control, the absorbance of 0.11 mm NADH in a 250 µL reaction mixture with 10 µL of buffer was measured. The enzyme activity was normalized to the intracellular protein content. GPD2 activity was determined on the basis of the phenazine methosulfate (PMS)‐mediated reduction of 3‐(4,5‐dimethylthiazol‐2‐yl)‐2,5‐diphenyl tetrazolium bromide (MTT) to MTT‐formazan, which absorbs at 570 nm.^[^ ^55^ ^]^ The 1 mL reaction mixture contained 75 µm MTT, 600 µm PMS, 10 mm DL‐glycerol‐3‐phosphate, 0.2% (*v*/*v*) Triton X‐100, 10 µm flavin adenine dinucleotide, 1 mm flavin mononucleotide, and 0.2 mL of cell extract. The mixture was incubated at 37 °C for 20 min, after which the absorbance at 570 nm was measured. The enzyme activity was normalized to the intracellular protein content.

### ARTP and FACS‐Based Screening

ARTP mutagenesis of *Schizochytrium* was conducted as follows.^[^ ^48^ ^]^ For FACS‐based screening, cells (OD_660_ 0.5) were stained with 60 µL of 100 µg mL^−1^ 4,4‐difluoro‐1,3,5,7‐tetramethyl‐4‐bora‐3a,4a‐diaza‐s‐indacene and analyzed using a BD FACSDiscover instrument. The sorting parameters were as follows: 70 µm nozzle, FITC channel (480/510 nm), and 200–300 V voltage. The cells were gated (P1: viable cells; P2: debris exclusion; P3: singlets) before isolation of the top 1% of the FITC‐bright population (P4). The detailed mutagenesis and screening strategy was as follows: Using a single colony as an example, it was inoculated into fermentation medium until the peak of fatty acid accumulation (72 h), followed by FACS‐based screening. Two 24‐well plates were collected (each plate seeded with two starting clones) and cultivated for 72 h, after which OD values were measured. Three mutant strains with higher OD values were selected and transferred into shake flasks for rescreening, where the strain with the highest DCW was chosen. To increase throughput, ten single colonies were selected and conducted mutagenesis and screening in parallel following the same procedure. After the first round, TFA content and DCW of the ten resulting strains were measured and found that ≈6–7 strains showed both higher TFA content and higher DCW than the parental strain, demonstrating the effectiveness of the method. Although certain mutants exhibited inferior fermentation performance compared with the parental strain—likely due to the inherent randomness of mutagenesis—they were also continued to select and constituted the pool of ten colonies for the next round of mutagenesis and screening. The selection criteria were as follows: *i*) TFA—cells with fluorescence intensity higher than the parental strain and within the top ≈1% of the cell population during FACS; *ii*) Growth—strains with the highest OD and DCW. The total number of colonies screened was 2640 (with each well inoculated with two parental colonies), and the positive rate was ≈1.4–1.6%.

### Genome Resequencing

Genomic DNA from log‐phase *Schizochytrium* strains (ZW1 and ZW2) was sequenced (BGI DNBSEQ T7 platform, 150 bp paired‐end) after library preparation (350 bp inserts). Clean data (23 M reads) were aligned to the reference genome (*Schizochytrium* sp. 31), and average depths of 81.08 × and 78.75 × were achieved for ZW1 and ZW2, respectively. SNPs/indels were identified, and key SNP sites were validated by PCR amplification in both strains.

### Transcriptomic Analysis

Total RNA from triplicate cultures (strains ZW1/ZW2) was poly(A)‐enriched, fragmented, and sequenced (Illumina NovaSeq 6000 platform). Clean reads were de novo assembled (Trinity), quantified (RSEM FPKM), and analyzed to identify DEGs (DESeq2: |fold change| ≥ 2, *q* < 0.05). Functional annotation was performed via the NR/KEGG databases.

### LC‒MS Metabolomics

LC‒MS metabolomics analysis was performed according to previously described methods.^[^ ^50^ ^]^ Briefly, metabolites extracted from cells (MeOH/H_2_O extraction, freeze‒thaw cycles) were analyzed via an Agilent 1260 HPLC‐6410 MS (C18 column, ESI). The data were processed using Mass Hunter software (Tables S8 and S9, Supporting Information).

### qRT‒PCR Analysis

Total RNA was extracted using TRIzol reagent and the miRNeasy Mini Kit. cDNA synthesis was performed via a HiScript II Q RT SuperMix for qPCR (+gDNA wiper) kit. qRT‒PCR was performed on a LightCycler 480 instrument (Roche) using the ChamQ Universal SYBR qPCR Master Mix Kit. The relative expression levels of each gene were calculated using the 2^−ΔΔCt^ method.^[^ ^56^ ^]^

### Protein Structure Prediction, Molecular Docking, and Energy Calculations

3D protein structures were predicted using AlphaFold2, leveraging UniRef/PDB homology for conserved regions. Models were assessed via pLDDT confidence scores. AutoDock Vina was applied to dock substrates (glycerol‐3‐phosphate, arachidonoyl‐1‐thio‐glycerol, and phosphatidylserine) to parent and mutant proteins (G3PP, MAL, and PSD). Binding pockets were defined by coordinates after preprocessing (hydrogenation, charge assignment). The binding pocket centers and sizes were defined as follows: for ZW1‐G3PP at residue 147: center = (12.852, 4.101, 0.213), pocket size = 18.5; for ZW2‐G3PP at residue 147: center = (7.142, −3.350, −12.359), pocket size = 18.5; for ZW1‐MAL at residue 249: center = (5.200, −8.900, −6.000), pocket size = 24; for ZW2‐MAL at residue 249: center = (−0.200, −4.400, −9.300), pocket size = 24; for ZW1‐PSD at residue 58: center = (0.800, −7.200, 10.400), pocket size = 30; for ZW2‐PSD at residue 58: center = (−1.700, 5.500, −5.300), pocket size = 30. Energy evaluation was performed using the Autodock4 scoring function. The primary energies calculated included the binding energy and intermolecular interaction energy.

### Molecular Dynamics Simulations

MD simulations were conducted using the GROMACS software with the CHARMM force field to describe intermolecular interactions. The simulation workflow was as follows: *i*) Preprocessing: protein desolvation, ligand hydrogenation (Avogadro), and topology file generation via CgenFF; *ii*) system setup: solvated in TIP3P water, neutralized with ions; *iii*) energy minimization; *iv*) equilibration (NVT/NPT); and v) production run. The trajectories were visualized using PyMOL. The number of docking repetitions was set to 30, and the scoring criteria uses the default function of GROMACS software, which considers the steric interactions of all atom pairs, the hydrophobic effect between hydrophobic atom pairs, and the interaction of hydrogen bonding.

### Statistical Analysis

Prior to analysis, TAG, G3P, and glycerol values were normalized to the mean of the control group. Data are presented as mean ± standard deviation (SD). Based on previous literature,^[^ ^29^, ^57^, ^58^, ^59^ ^]^ the measured parameters—including fatty acid levels, enzyme activities, and intracellular metabolites—were assumed to follow a normal distribution. Homogeneity of variances was assessed using Levene's test in OriginPro 2021. For comparisons between two groups, a two‐tailed Student's *t*‐test was performed in Microsoft Excel. For comparisons among three or more groups, one‐way ANOVA followed by Tukey's multiple comparison test was conducted using the Paired Comparison Plot tool in OriginPro 2021. Sample sizes were at least n = 3 per group, and differences were considered statistically significant at *p* < 0.05. * *p* < 0.05, ** *p* < 0.01.

## Conflict of Interest

The authors declare no conflict of interest.

## Author Contributions

F.W. and W.J. contributed equally to this work. F.W. conducted investigation, methodology, formal analysis, data curation, writing (original draft, review, editing), supervision, and funding acquisition. W.J. conducted investigation, methodology, formal analysis, and data curation. T.W. performed methodology, formal analysis, and data curation. J.J. carried out methodology, formal analysis, data curation, and supervision. K.C. performed methodology, formal analysis, and data curation. J.L. conducted formal analysis, data curation, writing (review, editing), and supervision. L.C. carried out formal analysis, data curation, writing (review, editing), and supervision. W.Z. conducted formal analysis, data curation, writing (review, editing), supervision, and funding acquisition.

## Supporting information



Supporting Information

Supplemental Data File

Supplemental Video 1

Supplemental Video 2

## Data Availability

The data that support the findings of this study are available from the corresponding author upon reasonable request. The accession codes (SRR32995179, SRR32997095, SRR33014728, SRR33018047) for the genome resequencing data and RNA‐seq data reported in this paper are available at the Sequence Read Archive at PRJNA1247005, PRJNA1247006, PRJNA1247007, PRJNA1247008. This paper does not report original code.
