# Supplementary material for: Suppressing Glycerol‐3‐phosphate Phosphatase and Enhancing Glycerol‐3‐Phosphate Shuttle Flux Crucial for High‐Efficiency Fatty Acid Production in the Fast‐Growing Oleaginous Schizochytrium
Source: Adv Sci (Weinh). 2025 Sep 24;12(45):e10021. doi: 10.1002/advs.202510021 (PMC12677618; doi:10.1002/advs.202510021)
Supplement: Supplementary file 1 — Supporting Information [file ADVS-12-e10021-s001.docx]

**Suppressing Glycerol-3-phosphate Phosphatase and Enhancing Glycerol-3-phosphate Shuttle Flux Crucial for High-Efficiency Fatty Acid Production in the Fast-growing Oleaginous *Schizochytrium***

*Fangzhong Wang^a,b^, Weijia Jin^a^, Tiantian Wang, Junkai Ji, Kun Cao, Jianqiang Li, Lei Chen^b^, Weiwen Zhang^b^*

^a^ These authors contributed equally to the paper.

Correspondence to:

^b^ [fangzhong.wang@tju.edu.cn](mailto:fangzhong.wang@tju.edu.cn); [Lchen@tju.edu.cn](mailto:Lchen@tju.edu.cn); [wwzhang8@tju.edu.cn](mailto:wwzhang8@tju.edu.cn)


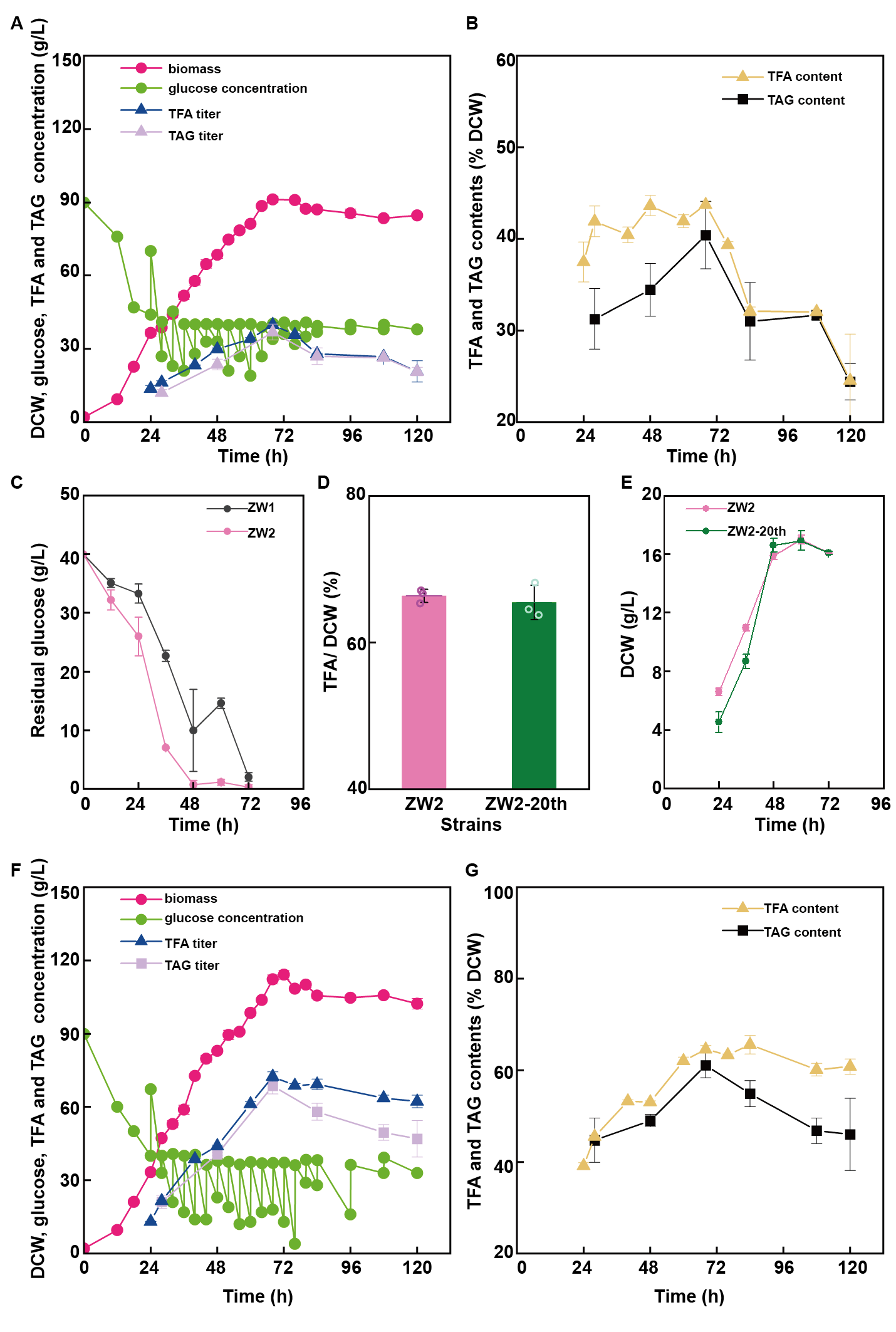
**Figure S1 *Schizochytrium* sp. strain ZW1 vs. ZW2 fermentation performance.** A) 5-L fermentation profiles of ZW1. Growth curve (pink), residual sugar titer (lime green), TFA titer (dark blue), and triacylglycerol titer (lavender). B) TFA (yellow) and TAG (black) content trends of ZW1. C) Glucose consumption: ZW1 (dark grey) vs. ZW2 (light rose). D-E) TFA (D) and Growth curves (E): ZW2 (light rose) vs. ZW2 (20th generation, green). F) 5-L fermentation profiles of ZW2. Growth curve (pink), residual glucose titer (lime green), TFA titer (dark blue), and triacylglycerol titer (lavender). G) TFA (yellow) and TAG (black) content trends of ZW2. Error bars represent mean ± SD. For panels A, B, F, G, data represent n = 3 technical replicates. For panels C–E, statistical analysis was performed using a two-tailed Student’s t-test in Microsoft Excel, with n = 3 biologically independent samples. Statistical significance was defined as *p* < 0.05. * *p* < 0.05, ** *p* < 0.01.

**
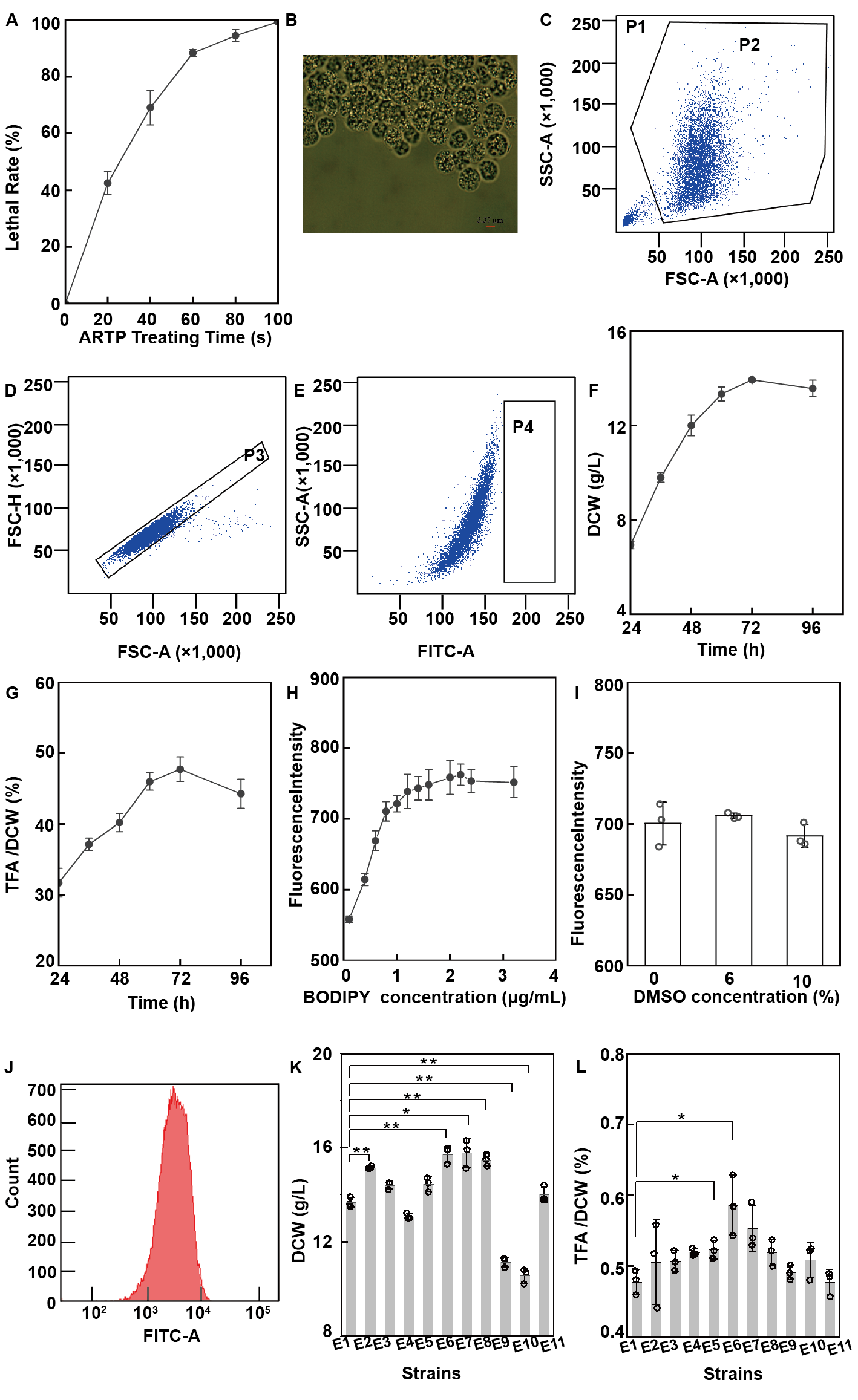
**

**Figure S2. Optimization of ARTP-FACS mutagenesis and screening parameters.**

A) ARTP lethality curve for ZW1; B) Microscopic images; C-E) Sorting strategy: P1: total cells; P2: debris exclusion; P3: single-cell gating; P4: top 1% fluorescence; F) Growth curve of ZW1; G) TFA accumulation profile of ZW1; H-I) Optimization of BODIPY staining (H) and DMSO concentration (I); J) Flow cytometry fluorescence analysis of BODIPY-stained ZW1; K-L) biomass content (K) and TFA content (L) of the final rounds of screened strains; E1: ZW1; E2-E11: mutant strains. Error bars represent mean ± SD. Data represent n = 3 biologically independent samples. Statistical analysis was performed using a two-tailed Student’s t-test in Microsoft Excel, with n = 3 biologically independent samples. Statistical significance was defined as *p* < 0.05. * *p* < 0.05, ** *p* < 0.01.

**
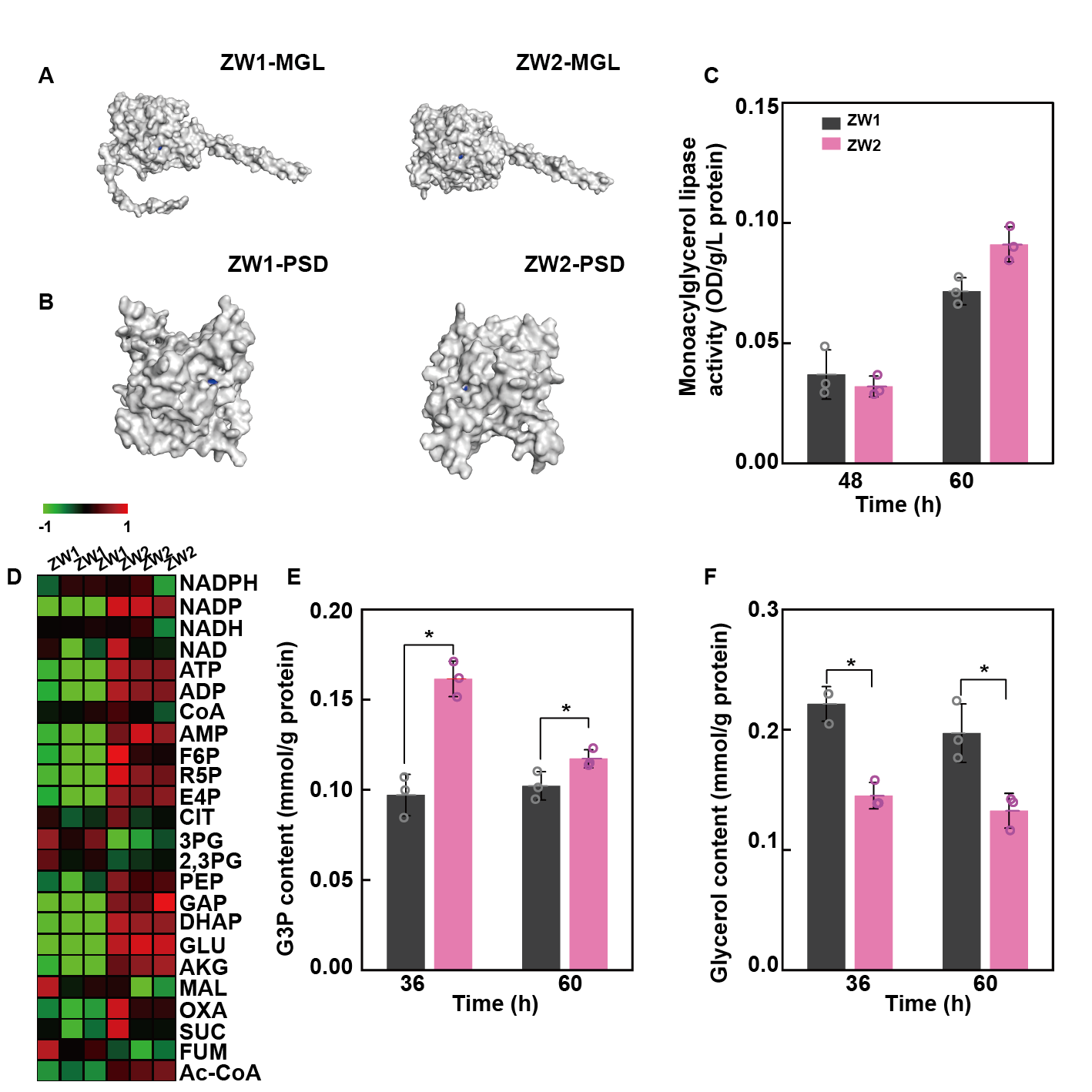
**

**Figure S3. Metabolomics, Protein structure prediction and enzyme activity analysis.** A) Predicted MAL structures: ZW1-MAL (overall confidence: 88.10; site 249: 98.07). ZW2-MAL (overall confidence: 96.12; site 249: 98.51). B) Predicted PSD structures, ZW1 (overall confidence: 84.09; site 249: 95.00), ZW2 (overall confidence: 83.81; site 249: 92.48). C) MAL activity comparison. ZW1: dark grey; ZW2: light rose. D) Comparative metabolomics analysis of ZW1 and ZW2 at 36 h. E-F) G3P (E) and glycerol (F) contents in ZW1 (dark grey) vs. ZW2 (light rose). Error bars represent mean ± SD. Data represent n = 3 biologically independent samples. Statistical analysis was performed using a two-tailed Student’s t-test in Microsoft Excel, with n = 3 biologically independent samples. Statistical significance was defined as *p* < 0.05. * *p* < 0.05.

**
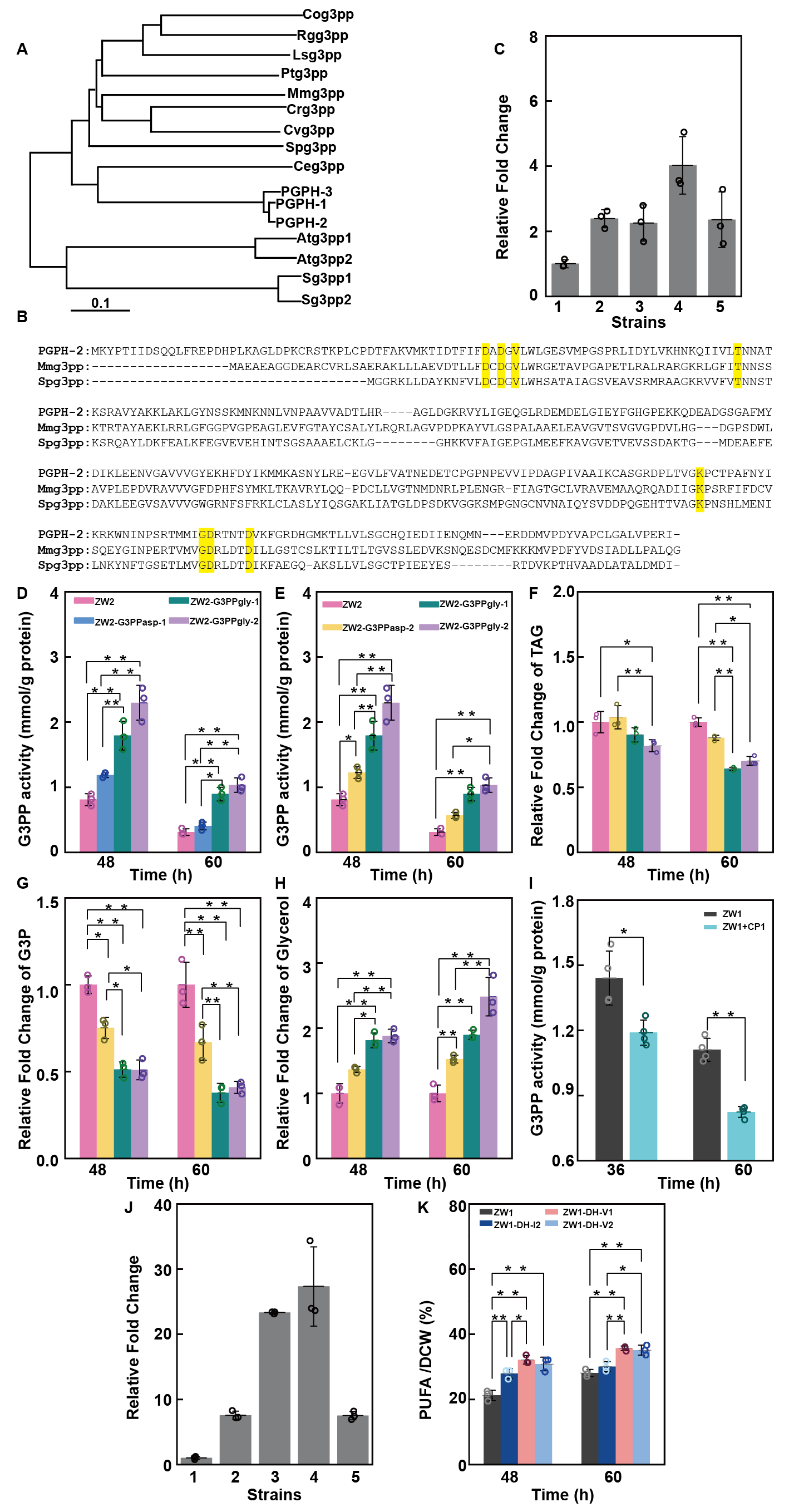
**

**Figure S4. Mutations in G3PP and ORFC sites increase TFA and PUFA production in *Schizochytrium* sp. strain ZW1.** A) Phylogenetic tree of G3PP proteins. Cog3pp: *C*. *oleaginosus* IBC0246 G3PP protein; Rgg3pp: *R*. *graminis* WP1 G3PP protein; Lsg3pp: *L*. *starkeyi* NRRL Y-11557 G3PP protein; Ptg3pp: *P*. *tricornutum* G3PP protein; Crg3pp: *C*. *reinhardtii* G3PP protein; Cvg3pp: *C*. *variabilis* G3PP protein; PGPH-1: *C.elegans* G3PP protein 1; PGPH-2: *C. elegans* G3PP protein 2; PGPH-3: *C. elegans* G3PP protein 3; Ceg3pp: *C. elegans* G3PP protein; Mmg3pp: *M. musculus* G3PP protein; Spg3pp: *Schizochytrium* G3PP protein; Atg3pp1: *A. thaliana* G3PP protein 1; Atg3pp2: *A. thaliana* G3PP protein 2; Sg3pp1: *S. cerevisiae* G3PP protein 1; Sg3pp2: *S. cerevisiae* G3PP protein 2. B) Multispecies G3PP alignment (PGPH-2, *C. elegans*; Mmg3pp, *M. musculus*; Spg3pp, *Schizochytrium*); the conserved active sites are highlighted. C) qRT‒PCR verification of gene expression. 1: ZW2; 2: ZW2-G3PP_gly_-1; 3: ZW2-G3PP_gly_-2; 4: ZW2-G3PP_asp_-1; 5: ZW2-G3PP_asp_-2. D-E) G3PP activity, ZW2: light rose; G3PP_asp_: blue (E)/yellow (F); G3PP_gly_: green/purple. F-H) Intracellular metabolite content analysis: TAG (F), G3P (G), and glycerol (H) contents across strains; ZW2: light rose; G3PP_asp_: yellow; G3PP_gly_: green/purple. I) G3PP activity in *Schizochytium* ZW1 without (dark grey) and with (cyan blue) the CP1 inhibitor. J) qRT‒PCR confirmation. 1: ZW1; 2: ZW1-DH-I1; 3: ZW1-DH-I2; 4: ZW1-DH-V1; 5: ZW1-DH-V2. K) PUFA content, ZW1: dark grey; ZW1-DH-I1: dark blue; ZW1-DH-V1: lavender; ZW1-DH-V2: light blue. Error bars represent mean ± SD. For panels D-H, K, statistical analysis was followed by one-way ANOVA with Tukey’s multiple comparison test using the Paired Comparison Plot tool in OriginPro 2021 (n = 3 biologically independent samples, * *p* < 0.05, ** *p* < 0.01), values in F-H were first normalized to the mean of the control group, then carried out for statistical analysis. For panels I, statistical analysis was performed using a two-tailed Student’s t-test in Microsoft Excel, with n = 4 biologically independent samples. Statistical significance was defined as *p* < 0.05. * *p* < 0.05, ** *p* < 0.01.


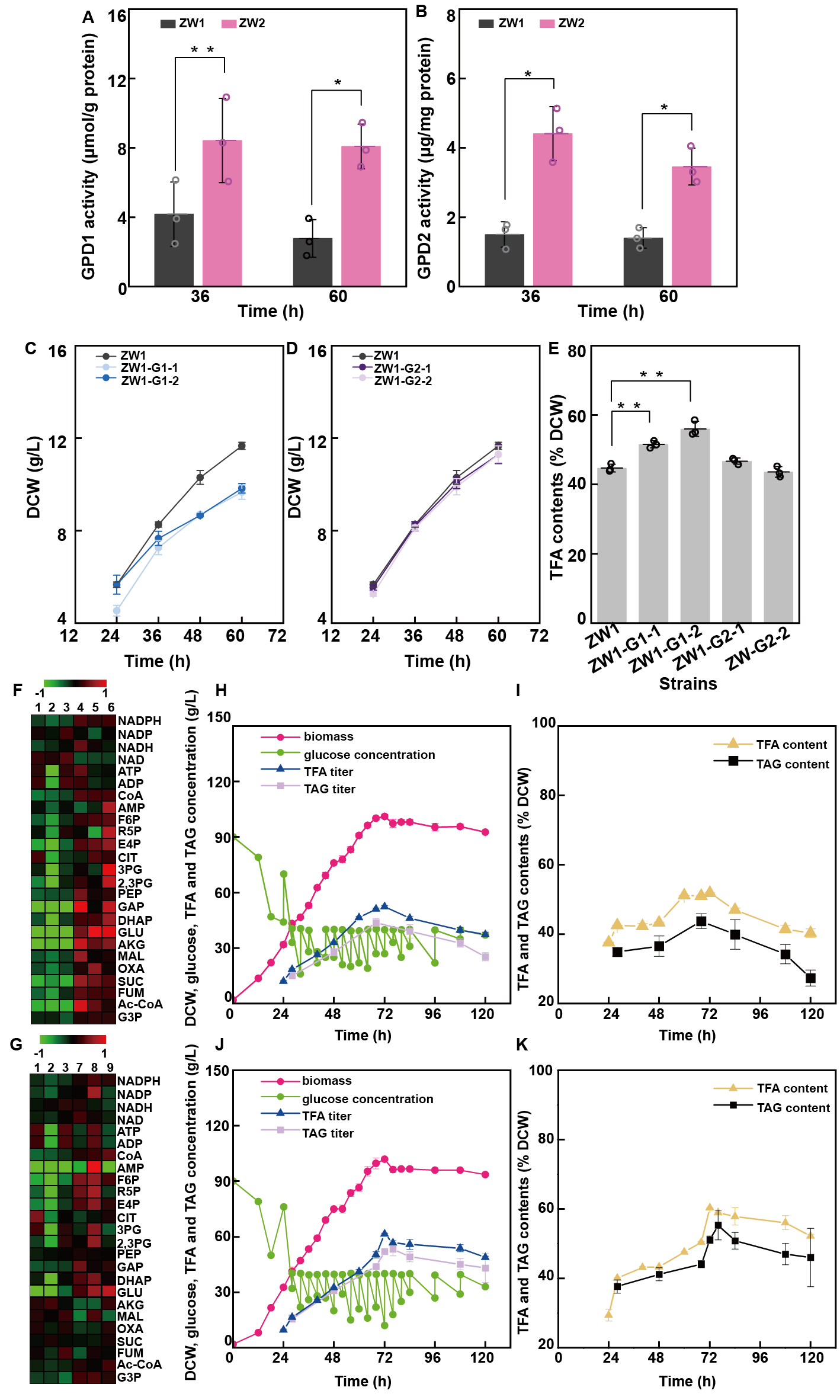


**Figure S5. Increased G3P cycle flux drives biomass and fatty acid production in *Schizochytrium* ZW2.** A-B) GPD1 (A) and GPD2 (B) enzyme activities: ZW1 (dark grey) vs. ZW2 (light rose). C-E) Growth curves (C-D) and TFA contents (E) of ZW1 and *gpd1* or *gpd2*-overexpressing strains. Dark grey: ZW1; Blue and light blue: ZW1-G1-1 and ZW1-G1-2. Purple and light purple: ZW1-G2-1 and ZW1-G2-2. F-G) Comparative metabolomics analysis of ZW1 and transformants overexpressing both *gpd 1* and *gpd 2*. 1, 2, 3: ZW1; 4, 5, 6: ZW1-G1G2-1; 7,8,9: ZW1-G1G2-2. H) *Schizochytrium* ZW1 G1G2-2 fermentation in a 5-L fermentation system. Growth curve (pink), residual glucose titer (lime green), TFA titer (dark blue), and triacylglycerol titer (lavender). J) TFA and TAG content curves of *Schizochytrium* ZW1 G1G2-2 in a 5-L fermentation system. TFA content (yellow) and TAG content (black). J) Fermentation of *Schizochytrium* ZW1 G1G2-2 with the CP1 inhibitor added at 56 hours. Growth curve (pink), residual glucose titer (lime green), TFA titer (dark blue), and triacylglycerol titer (lavender). K) TFA and TAG content curves of *Schizochytrium* ZW1 G1G2-2 with the CP1 inhibitor added at 56 hours. TFA content (yellow) and TAG content (black). Error bars represent mean ± SD. For panels A and B, statistical analysis was performed using a two-tailed Student’s t-test in Microsoft Excel, with n = 3 biologically independent samples. * *p* < 0.05, ** *p* < 0.01. For panels E, statistical analysis was followed by one-way ANOVA with Tukey’s multiple comparison test using the Paired Comparison Plot tool in OriginPro 2021 (n = 3 biologically independent samples. Statistical significance was defined as *p* < 0.05. * *p* < 0.05, ** *p* < 0.01). For panels H-K, data represent n = 3 technical replicates.


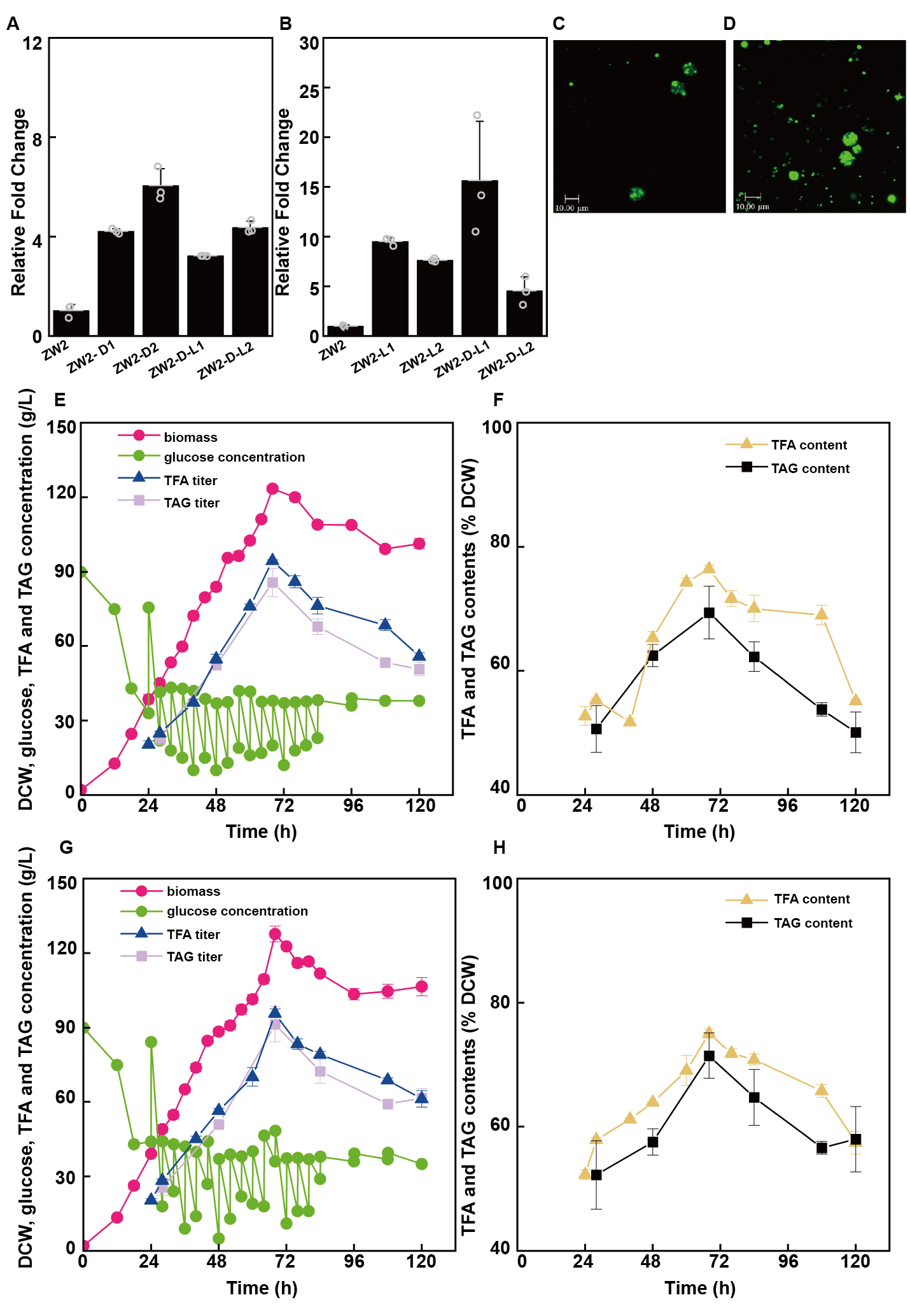


**Figure S6 Metabolic engineering of *Schizochytrium* sp. strain ZW2 to increase fatty acid production.** A-B) qRT‒PCR confirmation of *dgat* (A) and *lcas* (B) expression in transformants. C-D) Cell staining at 68 h of cultivation in a 5-L fermentation system: ZW1 (C) vs. ZW2-D-L-2 (D). E) Fermentation of ZW2-D-L-2. Growth curve (pink), residual glucose titer (lime green), TFA titer (dark blue), and triacylglycerol titer (lavender). G) TFA and TAG content curves of ZW2-D-L-2 in a 5-L fermentation system. TFA content (yellow) and TAG content (black). H) Fermentation of *Schizochytrium* ZW2-D-L-2. Growth curve (pink), residual glucose titer (lime green), TFA titer (dark blue), and triacylglycerol titer (lavender). I) TFA and TAG content curves of *Schizochytrium* ZW2-D-L-2 in a 5-L fermentation system. TFA content (yellow) and TAG content (black). Error bars represent mean ± SD. A and B, n = 3 biologically independent samples; E-H, n=3 technical replicates.


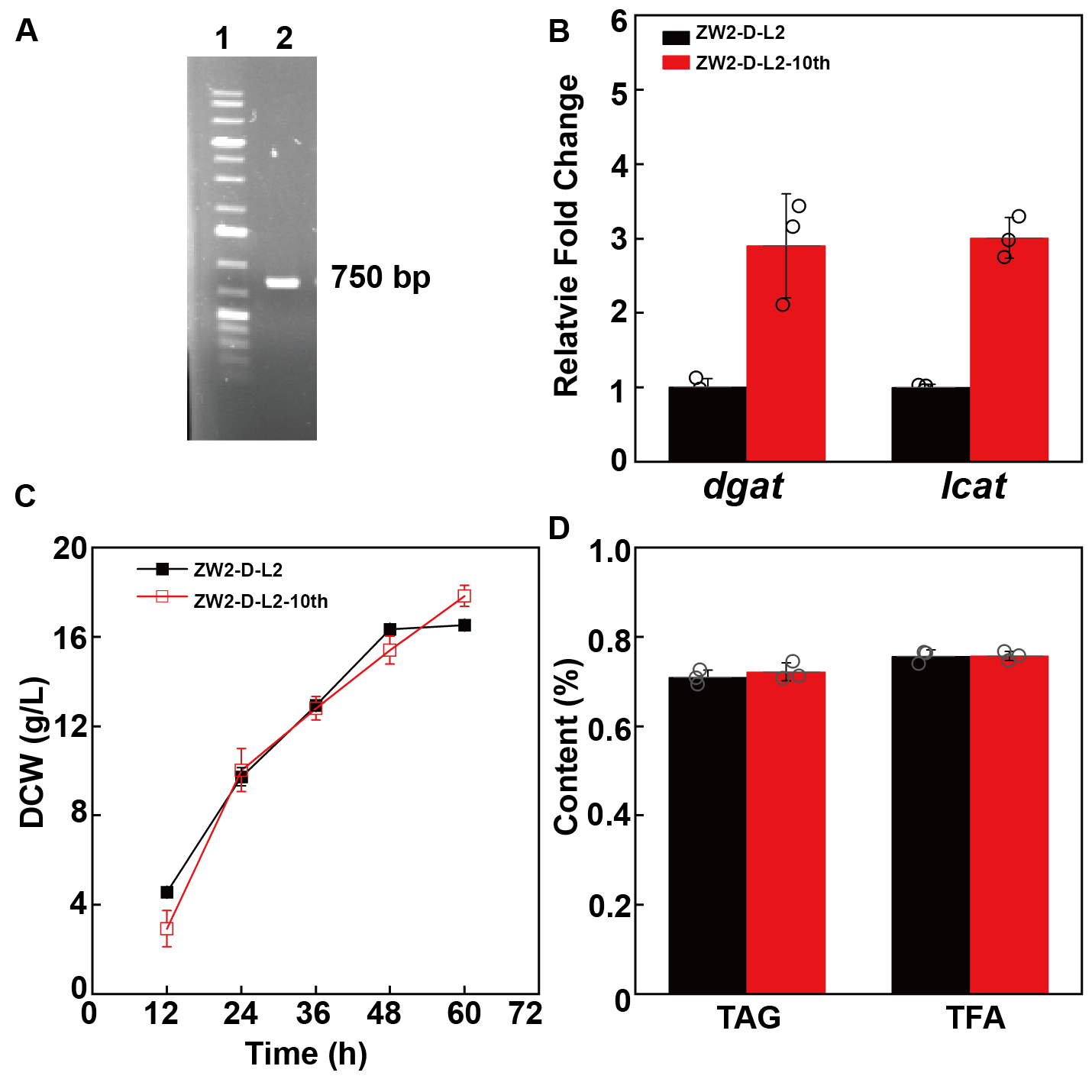


**Figure S7. Assessment of genetic and phenotypic stability in the ZW2-D-L-2 strain.** A) PCR amplification of the *g418* resistance gene (∼750 bp) after 10 successive passages under non-selective conditions. 1: DNA marker, 2: the *g418* resistance gene. B) Relative expression levels of target genes were measured via qRT-PCR. C) Growth curves of ZW2-D-L-2 and ZW2-D-L-2-10th. D) Comparison of TFA and TAG contents between ZW2-D-L-2 and ZW2-D-L-2-10th. Error bars represent mean ± SD. Statistical analysis was performed using a two-tailed Student’s t-test in Microsoft Excel, with n = 3 biologically independent samples. Statistical significance was defined as *p* < 0.05.

**Supplementary Table S1 Strains used in this study**

| Name | Parent strain | Relevant Manipulation |
| --- | --- | --- |
| *Schizochytrium* ZW2-G3PP_asp_-1 | *Schizochytrium* ZW2 | Overexpression of *g3pp*-*asp* |
| *Schizochytrium* ZW2-G3PP_asp_-2 | *Schizochytrium* ZW2 | Overexpression of *g3pp-asp* |
| *Schizochytrium* ZW2-G3PP_gly_-1 | *Schizochytrium* ZW2 | Overexpression of *g3ppase*-*gly* |
| *Schizochytrium* ZW2-G3PP_gly_-2 | *Schizochytrium* ZW2 | Overexpression of *g3ppase*-*gly* |
| *Schizochytrium* ZW1-DH-I1 | *Schizochytrium* ZW1 | Overexpression of *dh*-*iso* |
| *Schizochytrium* ZW1-DH-I2 | *Schizochytrium* ZW1 | Overexpression of *dh*-*iso* |
| *Schizochytrium* ZW1-DH-V1 | *Schizochytrium* ZW1 | Overexpression of *dh*-*val* |
| *Schizochytrium* ZW1-DH-V2 | *Schizochytrium* ZW1 | Overexpression of *dh*-*val* |
| *Schizochytrium* ZW1-G1-1 | *Schizochytrium* ZW1 | Overexpression of *gpd1* |
| *Schizochytrium* ZW1-G1-2 | *Schizochytrium* ZW1 | Overexpression of *gpd1* |
| *Schizochytrium* ZW1-G2-1 | *Schizochytrium* ZW1 | Overexpression of *gpd2* |
| *Schizochytrium* ZW1-G2-2 | *Schizochytrium* ZW1 | Overexpression of *gpd2* |
| *Schizochytrium* ZW1-G1G2-1 | *Schizochytrium* ZW1 | Overexpression of *gpd1* and *gpd2* |
| *Schizochytrium* ZW1-G1G2-2 | *Schizochytrium* ZW1 | Overexpression of *gpd1* and *gpd2* |
| *E. coli* BL21 (DE3)-DH-I | *E*. *coli* BL21 (DE3) | Ovexpression of pACYC184-hetI-SsorfA; pET22b-SsorfB；pRSFDuet-1-SsorfC-DHI |
| *E. coli* BL21 (DE3)-DH-V | *E*. *coli* BL21 (DE3) | Ovexpression of pACYC184-hetI-SsorfA; pET22b-SsorfB；pRSFDuet-1-SsorfC-DHV |
| *Schizochytrium* ZW2-D1 | *Schizochytrium* ZW2 | Overexpression of *dgat* |
| *Schizochytrium* ZW2-D2 | *Schizochytrium* ZW2 | Overexpression of *dgat* |
| *Schizochytrium* ZW2-L1 | *Schizochytrium* ZW2 | Overexpression of *lcat* |
| *Schizochytrium* ZW2-L2 | *Schizochytrium* ZW2 | Overexpression of *lcat* |
| *Schizochytrium* ZW2-D-L1 | *Schizochytrium* ZW2 | Overexpression of *dgat* and *lcat* |
| *Schizochytrium* ZW2-D-L2 | *Schizochytrium* ZW2 | Overexpression of *dgat* and *lcat* |
| *Schizochytrium* ZW1 | *Schizochytrium* ATCC20888 | Adaptive evolution |
| *Schizochytrium* M2 | *Schizochytrium* ZW1 | ARTP mutagenesis |
| *Schizochytrium* M3 | *Schizochytrium* ZW1 | ARTP mutagenesis |
| *Schizochytrium* M4 | *Schizochytrium* ZW1 | ARTP mutagenesis |
| *Schizochytrium* M5 | *Schizochytrium* ZW1 | ARTP mutagenesis |
| *Schizochytrium* M6 | *Schizochytrium* ZW1 | ARTP mutagenesis |
| *Schizochytrium* M7 | *Schizochytrium* ZW1 | ARTP mutagenesis |
| *Schizochytrium* M8 | *Schizochytrium* ZW1 | ARTP mutagenesis |
| *Schizochytrium* M9 | *Schizochytrium* ZW1 | ARTP mutagenesis |
| *Schizochytrium* M10 | *Schizochytrium* ZW1 | ARTP mutagenesis |
| *Schizochytrium* M11 | *Schizochytrium* ZW1 | ARTP mutagenesis |

**Supplementary Table S2 Plasmids constructed in this study**

| Supplementary Table S2 plasmids constructed in this study | |  |
| --- | --- | --- |
| Plasmid | Genotype | Source |
| pACYC184-*hetI*-*SsorfA* | *lacI*, *hetI*, *SsorfA*, Cm^r^ | This study |
| pACYC184-*hetI* | *lacI*, *hetI*, Cm^r^ | This study |
| pACYC184-*SsorfA* | *lacI*, *SsorfA*, Cm^r^ | This study |
| pET22b-*SsorfB* | *lacI*, *SsorfB*, Amp^r^ | This study |
| pRSFDuet-1-*SsorfC* | *lacI*, *SsorfC*, Km^r^ | This study |
| PUCGW-G418-G3PPA | *lacI*, *g418,* *g3ppa*, Amp^r^ | This study |
| PUCGW-G418-G3PPG | *lacI*, *g418*, *g3ppg*, Amp^r^ | This study |
| PUCGW-G418-DHI | *lacI*, *g418*, *DHI*, Amp^r^ | This study |
| PUCGW-G418-DHV | *lacI*, *g418*, *DHV*, Amp^r^ | This study |
| PUCGW-G418-GPD1 | *lacI*, *g418*, *gpd1*, Amp^r^ | This study |
| PUCGW-G418-GPD2 | *lacI*, *g418*, *gpd2*, Amp^r^ | This study |
| PUCGW-G418-GPD1-GPD2 | *lacI*, *g418*, *gpd1*, *gpd2*, Amp^r^ | This study |
| PUCGW-G418-DGAT | *lacI*, *g418,* *dgat*, Amp^r^ | This study |
| PUCGW-G418-LCAT | *lacI*, *g418*, *lcat*, Amp^r^ | This study |
| PUCGW-G418-DGAT-LCAT | *lacI*, *g418*, *dgat*, *lcat*, Amp^r^ | This study |

**Supplementary Table S3 Primers used in this study**

| Primers | sequence (5'-3') |
| --- | --- |
| gpd1-pug-F | TTTGCTGGAGACGCTGCAGCATcttagattagattgctatgctttctt |
| gpd1--pug-R | TCTCGCGCGCTCAATGCTGTGAgatcgttcaaacatttggcaat |
| gpd1-F | TCACAGCATTGAGCGCGCGAGAGG |
| gpd1-R | ATGCTGCAGCGTCTCCAGCAAATGA |
| gpd2-F | ATGGCGGCGCCGCTGGGC |
| gpd2-R | TCATCCGAACATGACGCCAGAAGAGGAAGAAGACTCGC |
| gpd2-pug-F | CTCTTCTGGCGTCATGTTCGGATGAtcaagaggatgtcagaatgccatt |
| gpd2-pug-R | GCCCAGCGGCGCCGCCATTCTTGCTGCGTGTGAAT |
| gpd1-gpd2-F | agaAGATCTCTCACCTCGTTCGTgatctagtaacatagatgacaccgcg |
| gpd1-gpd2-R | ATGACGCCGCCGGCAACGTTGTCAttttccctctttcttcctcta |
| gpd2-gpd1-F | TGACAACGTTGCCGGCGGCGTCA |
| gpd2-gpd1-R | ACGAACGAGGTGAGAGATCTtctca |
| lcat-F | agcatagcaatctaatctaagATGCCGAAACCTCTGGAAACAGCCC |
| lcat-R | cgtgacataactaattacatgaTCAGGCGTGAACCTCGGCGCGGTTG |
| pug-lcat-F | tcatgtaattagttatgtcacgcttacattcacgccc |
| pug-lcat-R | cttagattagattgctatgctttctttctaatgaacaagaagtaaaaa |
| dgat-F | aagcatagcaatctaatctaagATGCCGGAACTAGAAAAGTACTATG |
| dgat-R | agcgtgacataactaattacatgaTTAAACAAAGCGTAGCTTCCG |
| pug-dgat-F | tcatgtaattagttatgtcacgcttacattcacgccc |
| pug-dgat-R | cttagattagattgctatgctttctttctaatgaacaagaagtaaaaa |
| dgat-lcat-F | gatctagtaacatagatgacaccgcgcgcgataa |
| dgat-lcat-R | cgttcaaagccacatcactgggcacttc |
| lcat-dgat-F | tgatgtggctttgaacgGGCCTGCGCCTGCATCTGGTCTGGTCTCT |
| lcat-dgat-R | gcgcggtgtcatctatgttactagatcgcaaattaaagccttcgagcgt |
| g3pp-F | ATGGGCGGGCGCAAGCTTCTC |
| g3pp-R | CTAAATGTCCATGTCCAGAGCTGTTGCAAGGTCC |
| g3pp-pug-F | CTCTGGACATGGACATTTAGtcatgtaattagttatgtcacgcttacattca |
| g3pp-pug-R | AGAAGCTTGCGCCCGCCCATcttagattagattgctatgctttc |
| DH-F | agaaagcatagcaatctaatctaagATGGACCTCGCCGCCCGCGCCA |
| DH-R | aagcgtgacataactaattacatgaTTACTTGCCGTAGTCGCTGATGC |
| DH-pug-F | tcatgtaattagttatgtcacgcttacattc |
| DH-pug-R | cttagattagattgctatgctttctttctaatgaaca |
| pRSFDuent-F1 | CTACGAGCCCACCAACGCGCTCTAAGAATTCGAGCTCCGTCGACAAGCT |
| pRSFDuet-R1 | TGTTCGTCTTGACACGGAGCGCCATATGTATATCTCCTTCTTAAAGTT |
| OrfC-F | ATGGCGCTCCGTGTCAAGACGAACAAGAAGC |
| OrfC-R | TTAGAGCGCGTTGGTGGGCTCGTAGACAAAGG |
| pet22b-SsorfB-R | CGGCGCTCACATTCCGAGCGGCCATATGTATATCTCCTTCTTAAAGTTA |
| pet22b-SsorfB-F | ATACGTCCCGGCCGAGAAGCTGTAAGAATTCGAGCTCCGTCGACAAGCT |
| SsorfB-F | ATGGCCGCTCGGAATGTGAGCGC |
| SsorfB-R | TTACAGCTTCTCGGCCGGGACGTATGTGTCAT |
| HetI-F | ATGTTGCAGCATACTTGGCTACCAAAACCC |
| HetI-R | TCAATAATGCCAGAATTTTGGCTGCCAGCC |
| p15A-F | GCAGCCAAAATTCTGGCATTATTGACCTAGGTGAGCAAAGGATCCGAAT |
| p15A-R | TTGGTAGCCAAGTATGCTGCAACATATGTATATCTCCTTCTTAAAGTTA |
| SsORFA-F | ATGGCGGCCCGTCTGCAGGAGCAAAAGGG |
| SsORFA-R | TTAGAAGGCAAGGCTGTCCGTGGCG |
| p15A-F-1 | CACGGACAGCCTTGCCTTCTAAGGATCCGAATTCGAGCTCCGTCGACAA |
| p15A-R-1 | TTTGCTCCTGCAGACGGGCCGCCATATGTATATCTCCTTCTTAAAGTTA |
| SsorfA-R2 | AATTCGGATCCTTTGCTCATTAGAAGGCAAGGCTGTCCGTGGCGA |
| SsorfA-F2 | TGACCTAGGCGCGAAATTAATACGACTCACTATAGGGG |
| DHVI-QRT-F | CTTCGACGAGGACAACGACC |
| DHVI-QRT-R | GATGCGGTCGAGGCTAAAGT |
| G3PP-QRT-F | TCTTTGCTATCGGTGAGCCC |
| G3PP-QRT-R | AACTCTGCCTCATCCATGCC |
| G418-F | ATGGGGATTGAACAAGATGGATTGC |
| G418-R | TCAGAAGAACTCGTCAAGAAGGCG |
| dgat-QRT-F | GTGGTCTATCGTACGCCGTT |
| dgat-QRT-R | ACAGAGTGACCTTTGGCGAG |
| lcat-QRT-F | ATGGGCTACCTTGCCAACTC |
| lcat-QRT-R | TCACCACTGTGAAGCCATCC |
| Actin-QRT-F | AAGATCAAGGTCGTCGCTCC |
| Actin-QRT-R | AGACTCGTCGTACTCCTGCT |
| gpd1-QRT-F | AAACTTGGGGCGGTGAAAGA |
| gpd1-QRT-R | CTCATGGGCGCAAATGTAGC |
| gpd2-QRT-F | TTGACATTCGCGCCAACAAG |
| gpd2-QRT-R | GAAAGGTAGCCCGGAAGGAC |

**Supplementary Table S4 Standard curves used in this study**

| Products | Stardarded curves |
| --- | --- |
| Glycerol | Y=3538.2X-9.9711; X: OD, Y: μM |
| G3P | Y=2334.6X+26.499; X: OD, Y: μM |
| C14:0 | Y1=（1.26X+0.006991）× Y2; X: peak ratio；Y2: absoluate mass of starndard sample, mg；Y1: mg |
| C15:0 | Y1=（1.16686X+0.010949）× Y2; X: peak ratio；Y2: absoluate mass of starndard sample, mg；Y1: mg |
| C16:1 | Y1=（0.9986X+0.011385）× Y2; X: peak ratio；Y2: absoluate mass of starndard sample, mg；Y1: mg |
| C16:0 | Y1=（1.075X+0.023422）× Y2; X: peak ratio；Y2: absoluate mass of starndard sample, mg；Y1: mg |
| C17:0 | Y1=（0.99596X+0.017412）× Y2; X: peak ratio；Y2: absoluate mass of starndard sample, mg；Y1: mg |
| C18:1 | Y1=（0.92748X+0.027272）× Y2; X: peak ratio；Y2: absoluate mass of starndard sample, mg；Y1: mg |
| C18:0 | Y1=（0.7755X+0.009816）× Y2; X: peak ratio；Y2: absoluate mass of starndard sample, mg；Y1: mg |
| EPA | Y1=（1.72736X+0.016635）× Y2; X: peak ratio；Y2: absoluate mass of starndard sample, mg；Y1: mg |
| DPA | Y1=（1.9019X+0.045781）× Y2; X: peak ratio；Y2: absoluate mass of starndard sample, mg；Y1: mg |
| DHA | Y1=（1.475X+0.11954）× Y2; X: peak ratio；Y2: absoluate mass of starndard sample, mg；Y1: mg |

**Supplementary Table S5 Indels and SNPs identified through genome resequencing**

Table S5-1: Indels in coding sequences between ZW2 and ZW1

| Strain | Ref_ID | Type | Base | Pos_type | Annotation_type | strand | gene_id | ref_gene_product | Classification |
| --- | --- | --- | --- | --- | --- | --- | --- | --- | --- |
| ZW1 | scaffold330_8210 | I1 | T | Gene_inner | CDS | - | scaffold330.g3 | DNA-directed RNA polymerase I subunit RPA43 [Hondaea fermentalgiana] | Transcription |
| ZW2 | scaffold90_12183 | D1 | T | Gene_inner | CDS | - | scaffold90.g4 | calpain-like protease [Thraustotheca clavata] | Peptidases and inhibitors |

Table S5-2: Indels in intergenic regions between ZW2 and ZW1

| Strain | Position | Type | Base | Pos_type |
| --- | --- | --- | --- | --- |
| ZW2 | scaffold104_16925 | I2 | TT | intergenic |
|  | scaffold187_10666 | I1 | C | intergenic |
|  | scaffold215_6567 | D8 | GGGGGGGG | intergenic |
|  | scaffold232_33878 | I2 | TC | intergenic |
|  | scaffold401_22668 | I3 | CCC | intergenic |
|  | scaffold805_15006 | I3 | TCT | intergenic |
|  | scaffold867_11172 | D4 | TCCA | intergenic |
|  | scaffold92_14576 | D1 | T | intergenic |
| ZW1 | scaffold1933_4339 | I3 | TCT | intergenic |
|  | scaffold226_14121 | I1 | T | intergenic |
|  | scaffold521_18442 | I3 | CCC | intergenic |
|  | scaffold541_8136 | I1 | G | intergenic |
|  | scaffold72_9240 | I2 | CC | intergenic |

Table S5-3: SNPs in intergenic regions between ZW2 and ZW1

| Position | ref | ST7A | STA | ref_base<->sample_base | ref_gene_ID |
| --- | --- | --- | --- | --- | --- |
| scaffold1083_5994 | T | G | T | T<->G | intergenic |
| scaffold1096_10719 | G | G | T | G<->T | intergenic |
| scaffold1670_6203 | T | G | T | T<->G | intergenic |
| scaffold2156_2613 | G | G | T | G<->T | intergenic |
| scaffold2394_203 | G | G | T | G<->T | intergenic |
| scaffold276_9555 | T | G | T | T<->G | intergenic |
| scaffold387_9518 | G | G | T | G<->T | intergenic |
| scaffold404_300 | G | G | T | G<->T | intergenic |
| scaffold404_670 | T | G | T | T<->G | intergenic |
| scaffold490_23275 | G | G | T | G<->T | intergenic |
| scaffold512_16893 | T | G | T | T<->G | intergenic |
| scaffold516_12648 | T | G | T | T<->G | intergenic |
| scaffold634_19772 | G | G | T | G<->T | intergenic |
| scaffold681_483 | G | G | T | G<->T | intergenic |
| scaffold726_236 | G | G | T | G<->T | intergenic |
| scaffold726_563 | G | G | T | G<->T | intergenic |
| scaffold978_201 | G | G | T | G<->T | intergenic |
| scaffold1064_7171 | T | C | T | T<->C | intergenic |
| scaffold1084_44 | C | C | T | C<->T | intergenic |
| scaffold1116_8662 | T | C | T | T<->C | intergenic |
| scaffold1170_10686 | T | C | T | T<->C | intergenic |
| scaffold1240_9958 | C | C | T | C<->T | intergenic |
| scaffold1241_7213 | T | C | T | T<->C | intergenic |
| scaffold1259_3241 | T | C | T | T<->C | intergenic |
| scaffold144_28471 | T | C | T | T<->C | intergenic |
| scaffold148_43053 | C | C | T | C<->T | intergenic |
| scaffold1536_7465 | T | C | T | T<->C | intergenic |
| scaffold1603_1106 | C | C | T | C<->T | intergenic |
| scaffold1603_1148 | T | C | T | T<->C | intergenic |
| scaffold1603_33 | C | C | T | C<->T | intergenic |
| scaffold1603_977 | C | C | T | C<->T | intergenic |
| scaffold1629_4819 | C | C | T | C<->T | intergenic |
| scaffold1686_6282 | T | C | T | T<->C | intergenic |
| scaffold1810_5482 | T | C | T | T<->C | intergenic |
| scaffold1847_4985 | T | C | T | T<->C | intergenic |
| scaffold1849_4876 | T | C | T | T<->C | intergenic |
| scaffold1852_4993 | C | C | T | C<->T | intergenic |
| scaffold188_15110 | T | C | T | T<->C | intergenic |
| scaffold188_15147 | T | C | T | T<->C | intergenic |
| scaffold18_40900 | T | C | T | T<->C | intergenic |
| scaffold18_44559 | T | C | T | T<->C | intergenic |
| scaffold1933_4342 | C | C | T | C<->T | intergenic |
| scaffold2049_3319 | T | C | T | T<->C | intergenic |
| scaffold2049_3674 | T | C | T | T<->C | intergenic |
| scaffold2049_579 | C | C | T | C<->T | intergenic |
| scaffold2053_854 | T | C | T | T<->C | intergenic |
| scaffold2056_3658 | C | C | T | C<->T | intergenic |
| scaffold2139_2963 | T | C | T | T<->C | intergenic |
| scaffold2139_3056 | T | C | T | T<->C | intergenic |
| scaffold2139_3068 | T | C | T | T<->C | intergenic |
| scaffold2156_2739 | C | C | T | C<->T | intergenic |
| scaffold2193_239 | T | C | T | T<->C | intergenic |
| scaffold2319_1421 | T | C | T | T<->C | intergenic |
| scaffold2391_292 | T | C | T | T<->C | intergenic |
| scaffold2450_106 | C | C | T | C<->T | intergenic |
| scaffold26_38 | C | C | T | C<->T | intergenic |
| scaffold390_27630 | C | C | T | C<->T | intergenic |
| scaffold404_1415 | C | C | T | C<->T | intergenic |
| scaffold404_1445 | C | C | T | C<->T | intergenic |
| scaffold404_46 | T | C | T | T<->C | intergenic |
| scaffold404_550 | T | C | T | T<->C | intergenic |
| scaffold404_632 | C | C | T | C<->T | intergenic |
| scaffold417_7901 | T | C | T | T<->C | intergenic |
| scaffold463_39 | T | C | T | T<->C | intergenic |
| scaffold469_20557 | T | C | T | T<->C | intergenic |
| scaffold516_12677 | C | C | T | C<->T | intergenic |
| scaffold516_13253 | T | C | T | T<->C | intergenic |
| scaffold516_13269 | T | C | T | T<->C | intergenic |
| scaffold516_2958 | C | C | T | C<->T | intergenic |
| scaffold516_727 | T | C | T | T<->C | intergenic |
| scaffold518_17439 | T | C | T | T<->C | intergenic |
| scaffold527_261 | C | C | T | C<->T | intergenic |
| scaffold527_280 | C | C | T | C<->T | intergenic |
| scaffold527_326 | C | C | T | C<->T | intergenic |
| scaffold55_128 | C | C | T | C<->T | intergenic |
| scaffold64_5014 | T | C | T | T<->C | intergenic |
| scaffold726_291 | T | C | T | T<->C | intergenic |
| scaffold74_29284 | T | C | T | T<->C | intergenic |
| scaffold77_25657 | T | C | T | T<->C | intergenic |
| scaffold7_83918 | G | C | T | G<->CT | intergenic |
| scaffold809_4854 | T | C | T | T<->C | intergenic |
| scaffold809_5567 | C | C | T | C<->T | intergenic |
| scaffold839_3392 | T | C | T | T<->C | intergenic |
| scaffold863_6163 | C | C | T | C<->T | intergenic |
| scaffold915_11330 | C | C | T | C<->T | intergenic |
| scaffold992_295 | T | C | T | T<->C | intergenic |
| scaffold174_29802 | A | A | T | A<->T | intergenic |
| scaffold1847_5039 | A | A | T | A<->T | intergenic |
| scaffold2402_174 | A | A | T | A<->T | intergenic |
| scaffold404_826 | T | A | T | T<->A | intergenic |
| scaffold515_3181 | T | A | T | T<->A | intergenic |
| scaffold516_12796 | T | A | T | T<->A | intergenic |
| scaffold519_5305 | A | A | T | A<->T | intergenic |
| scaffold70_17377 | A | A | T | A<->T | intergenic |
| scaffold90_16302 | A | A | T | A<->T | intergenic |
| scaffold978_222 | A | A | T | A<->T | intergenic |
| scaffold978_280 | T | A | T | T<->A | intergenic |
| scaffold148_43026 | T | T | G | T<->G | intergenic |
| scaffold1603_1093 | G | T | G | G<->T | intergenic |
| scaffold1670_6146 | G | T | G | G<->T | intergenic |
| scaffold1678_6390 | G | T | G | G<->T | intergenic |
| scaffold187_38518 | T | T | G | T<->G | intergenic |
| scaffold188_14964 | G | T | G | G<->T | intergenic |
| scaffold2049_604 | T | T | G | T<->G | intergenic |
| scaffold2454_48 | G | T | G | G<->T | intergenic |
| scaffold279_6123 | T | T | G | T<->G | intergenic |
| scaffold404_344 | T | T | G | T<->G | intergenic |
| scaffold516_12658 | T | T | G | T<->G | intergenic |
| scaffold534_4449 | G | T | G | G<->T | intergenic |
| scaffold679_3538 | T | T | G | T<->G | intergenic |
| scaffold722_29 | G | T | G | G<->T | intergenic |
| scaffold792_16708 | G | T | G | G<->T | intergenic |
| scaffold806_95 | G | T | G | G<->T | intergenic |
| scaffold809_754 | G | T | G | G<->T | intergenic |
| scaffold814_26 | G | T | G | G<->T | intergenic |
| scaffold915_11355 | T | T | G | T<->G | intergenic |
| scaffold1137_148 | C | C | G | C<->G | intergenic |
| scaffold114_4284 | G | C | G | G<->C | intergenic |
| scaffold11_21246 | G | C | G | G<->C | intergenic |
| scaffold11_39042 | G | C | G | G<->C | intergenic |
| scaffold121_1701 | C | C | G | C<->G | intergenic |
| scaffold124_39670 | C | C | G | C<->G | intergenic |
| scaffold1288_8062 | G | C | G | G<->C | intergenic |
| scaffold1358_2254 | G | C | G | G<->C | intergenic |
| scaffold149_4557 | G | C | G | G<->C | intergenic |
| scaffold150_28552 | G | C | G | G<->C | intergenic |
| scaffold1541_6787 | C | C | G | C<->G | intergenic |
| scaffold155_4232 | C | C | G | C<->G | intergenic |
| scaffold1592_5622 | G | C | G | G<->C | intergenic |
| scaffold159_24550 | G | C | G | G<->C | intergenic |
| scaffold1659_2396 | C | C | G | C<->G | intergenic |
| scaffold1684_4834 | C | C | G | C<->G | intergenic |
| scaffold1804_5502 | C | C | G | C<->G | intergenic |
| scaffold1804_5510 | C | C | G | C<->G | intergenic |
| scaffold182_8423 | C | C | G | C<->G | intergenic |
| scaffold183_30825 | C | C | G | C<->G | intergenic |
| scaffold1847_4995 | C | C | G | C<->G | intergenic |
| scaffold1965_4519 | G | C | G | G<->C | intergenic |
| scaffold1_57572 | C | C | G | C<->G | intergenic |
| scaffold2179_2433 | A | C | G | A<->CG | intergenic |
| scaffold220_11916 | C | C | G | C<->G | intergenic |
| scaffold236_899 | C | C | G | C<->G | intergenic |
| scaffold241_12452 | C | C | G | C<->G | intergenic |
| scaffold258_27762 | C | C | G | C<->G | intergenic |
| scaffold261_37 | G | C | G | G<->C | intergenic |
| scaffold26_44495 | C | C | G | C<->G | intergenic |
| scaffold28_58120 | C | C | G | C<->G | intergenic |
| scaffold295_11705 | C | C | G | C<->G | intergenic |
| scaffold308_5869 | G | C | G | G<->C | intergenic |
| scaffold31_31344 | G | C | G | G<->C | intergenic |
| scaffold31_31371 | G | C | G | G<->C | intergenic |
| scaffold31_31395 | G | C | G | G<->C | intergenic |
| scaffold373_50 | C | C | G | C<->G | intergenic |
| scaffold401_20620 | C | C | G | C<->G | intergenic |
| scaffold404_26 | G | C | G | G<->C | intergenic |
| scaffold469_23807 | C | C | G | C<->G | intergenic |
| scaffold490_23647 | C | C | G | C<->G | intergenic |
| scaffold490_23984 | C | C | G | C<->G | intergenic |
| scaffold499_4148 | C | C | G | C<->G | intergenic |
| scaffold516_12741 | G | C | G | G<->C | intergenic |
| scaffold541_8149 | C | C | G | C<->G | intergenic |
| scaffold564_4849 | G | C | G | G<->C | intergenic |
| scaffold575_4689 | G | C | G | G<->C | intergenic |
| scaffold746_13088 | C | C | G | C<->G | intergenic |
| scaffold779_10499 | G | C | G | G<->C | intergenic |
| scaffold7_78057 | G | C | G | G<->C | intergenic |
| scaffold809_772 | G | C | G | G<->C | intergenic |
| scaffold80_23925 | G | C | G | G<->C | intergenic |
| scaffold81_13308 | C | C | G | C<->G | intergenic |
| scaffold926_4596 | G | C | G | G<->C | intergenic |
| scaffold930_5315 | C | C | G | C<->G | intergenic |
| scaffold105_29105 | G | A | G | G<->A | intergenic |
| scaffold1086_9669 | G | A | G | G<->A | intergenic |
| scaffold1087_4394 | G | A | G | G<->A | intergenic |
| scaffold1089_11793 | A | A | G | A<->G | intergenic |
| scaffold1167_6915 | G | A | G | G<->A | intergenic |
| scaffold1169_10965 | A | A | G | A<->G | intergenic |
| scaffold1176_776 | A | A | G | A<->G | intergenic |
| scaffold1187_595 | A | A | G | A<->G | intergenic |
| scaffold1229_6907 | G | A | G | G<->A | intergenic |
| scaffold1231_7358 | A | A | G | A<->G | intergenic |
| scaffold1232_10531 | G | A | G | G<->A | intergenic |
| scaffold123_18507 | A | A | G | A<->G | intergenic |
| scaffold1259_2795 | A | A | G | A<->G | intergenic |
| scaffold1272_6013 | G | A | G | G<->A | intergenic |
| scaffold127_15826 | G | A | G | G<->A | intergenic |
| scaffold128_105 | A | A | G | A<->G | intergenic |
| scaffold134_37744 | G | A | G | G<->A | intergenic |
| scaffold1428_29 | G | A | G | G<->A | intergenic |
| scaffold1429_3694 | A | A | G | A<->G | intergenic |
| scaffold148_18863 | A | A | G | A<->G | intergenic |
| scaffold148_43097 | A | A | G | A<->G | intergenic |
| scaffold148_43131 | A | A | G | A<->G | intergenic |
| scaffold1559_145 | A | A | G | A<->G | intergenic |
| scaffold1566_3838 | G | A | G | G<->A | intergenic |
| scaffold159_23848 | G | A | G | G<->A | intergenic |
| scaffold15_42992 | G | A | G | G<->A | intergenic |
| scaffold1603_1082 | G | A | G | G<->A | intergenic |
| scaffold1603_41 | A | A | G | A<->G | intergenic |
| scaffold1603_569 | G | A | G | G<->A | intergenic |
| scaffold1603_90 | A | A | G | A<->G | intergenic |
| scaffold1603_997 | G | A | G | G<->A | intergenic |
| scaffold1764_446 | G | A | G | G<->A | intergenic |
| scaffold1765_4543 | G | A | G | G<->A | intergenic |
| scaffold1789_5111 | G | A | G | G<->A | intergenic |
| scaffold182_32204 | G | A | G | G<->A | intergenic |
| scaffold1852_4868 | A | A | G | A<->G | intergenic |
| scaffold1885_4851 | G | A | G | G<->A | intergenic |
| scaffold1932_2732 | A | A | G | A<->G | intergenic |
| scaffold1984_555 | A | A | G | A<->G | intergenic |
| scaffold2104_1152 | A | A | G | A<->G | intergenic |
| scaffold2156_2361 | A | A | G | A<->G | intergenic |
| scaffold2156_2598 | A | A | G | A<->G | intergenic |
| scaffold2193_247 | G | A | G | G<->A | intergenic |
| scaffold21_11259 | G | A | G | G<->A | intergenic |
| scaffold230_580 | G | A | G | G<->A | intergenic |
| scaffold231_23565 | A | A | G | A<->G | intergenic |
| scaffold2391_210 | A | A | G | A<->G | intergenic |
| scaffold2391_43 | G | A | G | G<->A | intergenic |
| scaffold2402_209 | A | A | G | A<->G | intergenic |
| scaffold25_61954 | G | A | G | G<->A | intergenic |
| scaffold291_3124 | G | A | G | G<->A | intergenic |
| scaffold294_6183 | G | A | G | G<->A | intergenic |
| scaffold343_20413 | A | A | G | A<->G | intergenic |
| scaffold347_1039 | G | A | G | G<->A | intergenic |
| scaffold381_1811 | A | A | G | A<->G | intergenic |
| scaffold404_541 | G | A | G | G<->A | intergenic |
| scaffold404_74 | G | A | G | G<->A | intergenic |
| scaffold463_402 | A | A | G | A<->G | intergenic |
| scaffold470_39 | G | A | G | G<->A | intergenic |
| scaffold489_3681 | G | A | G | G<->A | intergenic |
| scaffold490_23348 | G | A | G | G<->A | intergenic |
| scaffold490_23638 | A | A | G | A<->G | intergenic |
| scaffold490_24162 | G | A | G | G<->A | intergenic |
| scaffold498_20310 | A | A | G | A<->G | intergenic |
| scaffold498_20317 | A | A | G | A<->G | intergenic |
| scaffold498_995 | A | A | G | A<->G | intergenic |
| scaffold516_12723 | G | A | G | G<->A | intergenic |
| scaffold516_12789 | G | A | G | G<->A | intergenic |
| scaffold516_13202 | G | A | G | G<->A | intergenic |
| scaffold516_218 | A | A | G | A<->G | intergenic |
| scaffold516_411 | G | A | G | G<->A | intergenic |
| scaffold520_18608 | G | A | G | G<->A | intergenic |
| scaffold527_270 | A | A | G | A<->G | intergenic |
| scaffold527_335 | A | A | G | A<->G | intergenic |
| scaffold57_1248 | A | A | G | A<->G | intergenic |
| scaffold598_2670 | G | A | G | G<->A | intergenic |
| scaffold5_107904 | A | A | G | A<->G | intergenic |
| scaffold615_2757 | G | A | G | G<->A | intergenic |
| scaffold615_495 | A | A | G | A<->G | intergenic |
| scaffold616_20282 | G | A | G | G<->A | intergenic |
| scaffold639_8260 | G | A | G | G<->A | intergenic |
| scaffold66_1490 | A | A | G | A<->G | intergenic |
| scaffold674_18989 | G | A | G | G<->A | intergenic |
| scaffold698_14084 | G | A | G | G<->A | intergenic |
| scaffold721_28 | G | A | G | G<->A | intergenic |
| scaffold75_46086 | G | A | G | G<->A | intergenic |
| scaffold787_1152 | G | A | G | G<->A | intergenic |
| scaffold794_274 | G | A | G | G<->A | intergenic |
| scaffold809_4791 | G | A | G | G<->A | intergenic |
| scaffold809_4804 | G | A | G | G<->A | intergenic |
| scaffold809_762 | G | A | G | G<->A | intergenic |
| scaffold80_39382 | G | A | G | G<->A | intergenic |
| scaffold816_16181 | A | A | G | A<->G | intergenic |
| scaffold837_7692 | G | A | G | G<->A | intergenic |
| scaffold85_7739 | G | A | G | G<->A | intergenic |
| scaffold89_45847 | A | A | G | A<->G | intergenic |
| scaffold940_270 | G | A | G | G<->A | intergenic |
| scaffold942_7656 | G | A | G | G<->A | intergenic |
| scaffold96_1070 | A | A | G | A<->G | intergenic |
| scaffold1340_9411 | C | G | C | C<->G | intergenic |
| scaffold1009_474 | T | T | C | T<->C | intergenic |
| scaffold100_32034 | T | T | C | T<->C | intergenic |
| scaffold1017_1496 | T | T | C | T<->C | intergenic |
| scaffold1095_7420 | C | T | C | C<->T | intergenic |
| scaffold1169_10932 | T | T | C | T<->C | intergenic |
| scaffold1190_6983 | C | T | C | C<->T | intergenic |
| scaffold1233_10097 | C | T | C | C<->T | intergenic |
| scaffold1245_5778 | T | T | C | T<->C | intergenic |
| scaffold126_37977 | C | T | C | C<->T | intergenic |
| scaffold12_29747 | T | T | C | T<->C | intergenic |
| scaffold1309_18 | C | T | C | C<->T | intergenic |
| scaffold1340_8861 | C | T | C | C<->T | intergenic |
| scaffold135_4159 | T | T | C | T<->C | intergenic |
| scaffold137_5527 | C | T | C | C<->T | intergenic |
| scaffold1426_8380 | T | T | C | T<->C | intergenic |
| scaffold1480_1169 | C | T | C | C<->T | intergenic |
| scaffold1508_3277 | C | T | C | C<->T | intergenic |
| scaffold1580_7148 | T | T | C | T<->C | intergenic |
| scaffold1603_1004 | C | T | C | C<->T | intergenic |
| scaffold1603_47 | T | T | C | T<->C | intergenic |
| scaffold1603_913 | C | T | C | C<->T | intergenic |
| scaffold1603_962 | C | T | C | C<->T | intergenic |
| scaffold1629_4688 | T | T | C | T<->C | intergenic |
| scaffold1684_5757 | T | T | C | T<->C | intergenic |
| scaffold1689_410 | T | T | C | T<->C | intergenic |
| scaffold1838_5083 | T | T | C | T<->C | intergenic |
| scaffold1847_5132 | C | T | C | C<->T | intergenic |
| scaffold1849_4494 | C | T | C | C<->T | intergenic |
| scaffold1852_3026 | C | T | C | C<->T | intergenic |
| scaffold188_14942 | C | T | C | C<->T | intergenic |
| scaffold1984_109 | C | T | C | C<->T | intergenic |
| scaffold2024_332 | C | T | C | C<->T | intergenic |
| scaffold203_12027 | C | T | C | C<->T | intergenic |
| scaffold2053_1369 | T | T | C | T<->C | intergenic |
| scaffold2056_3677 | T | T | C | T<->C | intergenic |
| scaffold206_23253 | C | T | C | C<->T | intergenic |
| scaffold207_14308 | T | T | C | T<->C | intergenic |
| scaffold2116_121 | C | T | C | C<->T | intergenic |
| scaffold2156_2802 | T | T | C | T<->C | intergenic |
| scaffold2156_2835 | C | T | C | C<->T | intergenic |
| scaffold2193_226 | C | T | C | C<->T | intergenic |
| scaffold2312_1589 | T | T | C | T<->C | intergenic |
| scaffold2319_1414 | C | T | C | C<->T | intergenic |
| scaffold2319_1713 | C | T | C | C<->T | intergenic |
| scaffold2391_344 | T | T | C | T<->C | intergenic |
| scaffold2391_77 | C | T | C | C<->T | intergenic |
| scaffold2402_178 | C | T | C | C<->T | intergenic |
| scaffold2403_74 | T | T | C | T<->C | intergenic |
| scaffold2403_90 | T | T | C | T<->C | intergenic |
| scaffold243_11747 | C | T | C | C<->T | intergenic |
| scaffold255_16586 | C | T | C | C<->T | intergenic |
| scaffold25_61961 | C | T | C | C<->T | intergenic |
| scaffold25_63483 | C | T | C | C<->T | intergenic |
| scaffold264_30737 | C | T | C | C<->T | intergenic |
| scaffold273_9930 | C | T | C | C<->T | intergenic |
| scaffold300_15727 | C | T | C | C<->T | intergenic |
| scaffold32_12432 | C | T | C | C<->T | intergenic |
| scaffold338_8833 | C | T | C | C<->T | intergenic |
| scaffold34_45417 | C | T | C | C<->T | intergenic |
| scaffold34_62478 | C | T | C | C<->T | intergenic |
| scaffold370_10089 | C | T | C | C<->T | intergenic |
| scaffold375_27509 | C | T | C | C<->T | intergenic |
| scaffold384_13663 | C | T | C | C<->T | intergenic |
| scaffold404_1396 | C | T | C | C<->T | intergenic |
| scaffold404_1457 | T | T | C | T<->C | intergenic |
| scaffold404_408 | T | T | C | T<->C | intergenic |
| scaffold463_337 | T | T | C | T<->C | intergenic |
| scaffold477_6488 | T | T | C | T<->C | intergenic |
| scaffold480_20929 | C | T | C | C<->T | intergenic |
| scaffold490_23315 | C | T | C | C<->T | intergenic |
| scaffold495_23606 | T | T | C | T<->C | intergenic |
| scaffold516_12729 | C | T | C | C<->T | intergenic |
| scaffold516_12989 | C | T | C | C<->T | intergenic |
| scaffold516_380 | C | T | C | C<->T | intergenic |
| scaffold537_22264 | T | T | C | T<->C | intergenic |
| scaffold538_3196 | C | T | C | C<->T | intergenic |
| scaffold573_18375 | C | T | C | C<->T | intergenic |
| scaffold590_756 | T | T | C | T<->C | intergenic |
| scaffold630_6401 | C | T | C | C<->T | intergenic |
| scaffold662_15191 | T | T | C | T<->C | intergenic |
| scaffold705_17189 | C | T | C | C<->T | intergenic |
| scaffold761_7316 | C | T | C | C<->T | intergenic |
| scaffold779_1756 | C | T | C | C<->T | intergenic |
| scaffold780_291 | T | T | C | T<->C | intergenic |
| scaffold793_413 | T | T | C | T<->C | intergenic |
| scaffold806_2722 | T | T | C | T<->C | intergenic |
| scaffold809_4130 | C | T | C | C<->T | intergenic |
| scaffold844_5506 | T | T | C | T<->C | intergenic |
| scaffold910_14217 | T | T | C | T<->C | intergenic |
| scaffold915_11222 | T | T | C | T<->C | intergenic |
| scaffold915_11259 | T | T | C | T<->C | intergenic |
| scaffold953_7713 | C | T | C | C<->T | intergenic |
| scaffold992_267 | C | T | C | C<->T | intergenic |
| scaffold993_12536 | C | T | C | C<->T | intergenic |
| scaffold9_38851 | C | T | C | C<->T | intergenic |
| scaffold1041_2057 | G | G | C | G<->C | intergenic |
| scaffold108_46810 | G | G | C | G<->C | intergenic |
| scaffold1136_618 | G | G | C | G<->C | intergenic |
| scaffold1140_11025 | G | G | C | G<->C | intergenic |
| scaffold1164_11171 | G | G | C | G<->C | intergenic |
| scaffold1238_6386 | C | G | C | C<->G | intergenic |
| scaffold126_24653 | G | G | C | G<->C | intergenic |
| scaffold1407_5143 | C | G | C | C<->G | intergenic |
| scaffold1499_3890 | G | G | C | G<->C | intergenic |
| scaffold149_17398 | C | G | C | C<->G | intergenic |
| scaffold1523_6269 | C | G | C | C<->G | intergenic |
| scaffold1524_256 | C | G | C | C<->G | intergenic |
| scaffold1603_905 | C | G | C | C<->G | intergenic |
| scaffold162_36721 | C | G | C | C<->G | intergenic |
| scaffold181_33990 | C | G | C | C<->G | intergenic |
| scaffold1836_766 | C | G | C | C<->G | intergenic |
| scaffold185_8326 | G | G | C | G<->C | intergenic |
| scaffold188_15177 | C | G | C | C<->G | intergenic |
| scaffold1932_1823 | C | G | C | C<->G | intergenic |
| scaffold2049_1894 | G | G | C | G<->C | intergenic |
| scaffold2056_3665 | G | G | C | G<->C | intergenic |
| scaffold2312_1580 | G | G | C | G<->C | intergenic |
| scaffold2319_1466 | C | G | C | C<->G | intergenic |
| scaffold24_29198 | C | G | C | C<->G | intergenic |
| scaffold309_31178 | G | G | C | G<->C | intergenic |
| scaffold316_30963 | C | G | C | C<->G | intergenic |
| scaffold337_22486 | C | G | C | C<->G | intergenic |
| scaffold36_37327 | C | G | C | C<->G | intergenic |
| scaffold400_15708 | G | G | C | G<->C | intergenic |
| scaffold404_371 | G | G | C | G<->C | intergenic |
| scaffold404_64 | C | G | C | C<->G | intergenic |
| scaffold404_643 | G | G | C | G<->C | intergenic |
| scaffold404_755 | C | G | C | C<->G | intergenic |
| scaffold432_555 | C | G | C | C<->G | intergenic |
| scaffold43_779 | G | G | C | G<->C | intergenic |
| scaffold455_24647 | G | G | C | G<->C | intergenic |
| scaffold47_60387 | G | G | C | G<->C | intergenic |
| scaffold516_12608 | G | G | C | G<->C | intergenic |
| scaffold516_2050 | C | G | C | C<->G | intergenic |
| scaffold51_12531 | G | G | C | G<->C | intergenic |
| scaffold545_11529 | C | G | C | C<->G | intergenic |
| scaffold60_56595 | C | G | C | C<->G | intergenic |
| scaffold610_9903 | G | G | C | G<->C | intergenic |
| scaffold621_10392 | G | G | C | G<->C | intergenic |
| scaffold622_16943 | C | G | C | C<->G | intergenic |
| scaffold629_12659 | C | G | C | C<->G | intergenic |
| scaffold64_5053 | C | G | C | C<->G | intergenic |
| scaffold675_8372 | G | G | C | G<->C | intergenic |
| scaffold686_11847 | G | G | C | G<->C | intergenic |
| scaffold736_16346 | G | G | C | G<->C | intergenic |
| scaffold78_52255 | C | G | C | C<->G | intergenic |
| scaffold817_8550 | C | G | C | C<->G | intergenic |
| scaffold82_24940 | G | G | C | G<->C | intergenic |
| scaffold851_11066 | C | G | C | C<->G | intergenic |
| scaffold884_12801 | C | G | C | C<->G | intergenic |
| scaffold90_16896 | G | G | C | G<->C | intergenic |
| scaffold915_11247 | G | G | C | G<->C | intergenic |
| scaffold941_3706 | G | G | C | G<->C | intergenic |
| scaffold1940_4079 | T | C | C | T<->C | intergenic |
| scaffold1034_10288 | A | A | C | A<->C | intergenic |
| scaffold11_65660 | A | A | C | A<->C | intergenic |
| scaffold1202_10784 | C | A | C | C<->A | intergenic |
| scaffold127_28994 | A | A | C | A<->C | intergenic |
| scaffold133_31144 | A | A | C | A<->C | intergenic |
| scaffold1509_7658 | C | A | C | C<->A | intergenic |
| scaffold174_11131 | A | A | C | A<->C | intergenic |
| scaffold1852_3186 | A | A | C | A<->C | intergenic |
| scaffold1874_4491 | A | A | C | A<->C | intergenic |
| scaffold1984_224 | A | A | C | A<->C | intergenic |
| scaffold200_99 | A | A | C | A<->C | intergenic |
| scaffold2053_1415 | A | A | C | A<->C | intergenic |
| scaffold30_54226 | G | A | C | G<->AC | intergenic |
| scaffold376_15866 | A | A | C | A<->C | intergenic |
| scaffold48_594 | A | A | C | A<->C | intergenic |
| scaffold490_23284 | A | A | C | A<->C | intergenic |
| scaffold516_2094 | C | A | C | C<->A | intergenic |
| scaffold60_51835 | C | A | C | C<->A | intergenic |
| scaffold616_20380 | C | A | C | C<->A | intergenic |
| scaffold631_18710 | A | A | C | A<->C | intergenic |
| scaffold700_13330 | C | A | C | C<->A | intergenic |
| scaffold915_11237 | A | A | C | A<->C | intergenic |
| scaffold929_12255 | A | A | C | A<->C | intergenic |
| scaffold1084_7893 | A | T | A | A<->T | intergenic |
| scaffold1309_64 | T | T | A | T<->A | intergenic |
| scaffold1603_1011 | A | T | A | A<->T | intergenic |
| scaffold1603_1120 | T | T | A | T<->A | intergenic |
| scaffold1626_161 | T | T | A | T<->A | intergenic |
| scaffold172_48 | A | T | A | A<->T | intergenic |
| scaffold1765_4553 | A | T | A | A<->T | intergenic |
| scaffold2049_3762 | A | T | A | A<->T | intergenic |
| scaffold402_7952 | A | T | A | A<->T | intergenic |
| scaffold451_13916 | C | T | A | C<->TA | intergenic |
| scaffold490_23616 | T | T | A | T<->A | intergenic |
| scaffold490_23910 | T | T | A | T<->A | intergenic |
| scaffold516_12777 | T | T | A | T<->A | intergenic |
| scaffold558_1572 | T | T | A | T<->A | intergenic |
| scaffold61_50026 | A | T | A | A<->T | intergenic |
| scaffold809_787 | T | T | A | T<->A | intergenic |
| scaffold829_15591 | T | T | A | T<->A | intergenic |
| scaffold1003_9593 | A | G | A | A<->G | intergenic |
| scaffold1084_7869 | A | G | A | A<->G | intergenic |
| scaffold1187_499 | G | G | A | G<->A | intergenic |
| scaffold1309_42 | G | G | A | G<->A | intergenic |
| scaffold130_5802 | A | G | A | A<->G | intergenic |
| scaffold1449_466 | A | G | A | A<->G | intergenic |
| scaffold1469_5027 | A | G | A | A<->G | intergenic |
| scaffold159_14068 | A | G | A | A<->G | intergenic |
| scaffold1603_209 | G | G | A | G<->A | intergenic |
| scaffold1603_530 | A | G | A | A<->G | intergenic |
| scaffold1603_82 | G | G | A | G<->A | intergenic |
| scaffold1670_6427 | G | G | A | G<->A | intergenic |
| scaffold1670_6456 | A | G | A | A<->G | intergenic |
| scaffold1793_5107 | G | G | A | G<->A | intergenic |
| scaffold1847_5052 | A | G | A | A<->G | intergenic |
| scaffold1847_5110 | G | G | A | G<->A | intergenic |
| scaffold1847_5227 | A | G | A | A<->G | intergenic |
| scaffold1849_5167 | G | G | A | G<->A | intergenic |
| scaffold188_14958 | G | G | A | G<->A | intergenic |
| scaffold2049_3396 | A | G | A | A<->G | intergenic |
| scaffold2049_3686 | A | G | A | A<->G | intergenic |
| scaffold2049_3740 | G | G | A | G<->A | intergenic |
| scaffold2053_1006 | A | G | A | A<->G | intergenic |
| scaffold2053_1036 | A | G | A | A<->G | intergenic |
| scaffold2053_1349 | G | G | A | G<->A | intergenic |
| scaffold2053_749 | A | G | A | A<->G | intergenic |
| scaffold207_37037 | A | G | A | A<->G | intergenic |
| scaffold2104_1162 | G | G | A | G<->A | intergenic |
| scaffold2136_3158 | G | G | A | G<->A | intergenic |
| scaffold2139_2960 | G | G | A | G<->A | intergenic |
| scaffold2156_2505 | G | G | A | G<->A | intergenic |
| scaffold2156_3036 | G | G | A | G<->A | intergenic |
| scaffold21_52647 | A | G | A | A<->G | intergenic |
| scaffold2319_1530 | G | G | A | G<->A | intergenic |
| scaffold286_13383 | A | G | A | A<->G | intergenic |
| scaffold36_20246 | A | G | A | A<->G | intergenic |
| scaffold397_12552 | A | G | A | A<->G | intergenic |
| scaffold397_47 | A | G | A | A<->G | intergenic |
| scaffold404_1347 | G | G | A | G<->A | intergenic |
| scaffold404_6591 | A | G | A | A<->G | intergenic |
| scaffold439_24461 | A | G | A | A<->G | intergenic |
| scaffold463_449 | G | G | A | G<->A | intergenic |
| scaffold490_23354 | A | G | A | A<->G | intergenic |
| scaffold490_23925 | G | G | A | G<->A | intergenic |
| scaffold498_20776 | G | G | A | G<->A | intergenic |
| scaffold507_19024 | A | G | A | A<->G | intergenic |
| scaffold516_12631 | A | G | A | A<->G | intergenic |
| scaffold516_13222 | G | G | A | G<->A | intergenic |
| scaffold516_2056 | A | G | A | A<->G | intergenic |
| scaffold516_2065 | A | G | A | A<->G | intergenic |
| scaffold516_34 | A | G | A | A<->G | intergenic |
| scaffold516_735 | A | G | A | A<->G | intergenic |
| scaffold659_1107 | A | G | A | A<->G | intergenic |
| scaffold675_16557 | A | G | A | A<->G | intergenic |
| scaffold685_18530 | G | G | A | G<->A | intergenic |
| scaffold6_87878 | A | G | A | A<->G | intergenic |
| scaffold721_129 | G | G | A | G<->A | intergenic |
| scaffold726_227 | G | G | A | G<->A | intergenic |
| scaffold726_408 | G | G | A | G<->A | intergenic |
| scaffold746_7182 | A | G | A | A<->G | intergenic |
| scaffold785_19 | A | G | A | A<->G | intergenic |
| scaffold809_4818 | A | G | A | A<->G | intergenic |
| scaffold809_5601 | A | G | A | A<->G | intergenic |
| scaffold95_29107 | A | G | A | A<->G | intergenic |
| scaffold978_175 | G | G | A | G<->A | intergenic |
| scaffold978_181 | G | G | A | G<->A | intergenic |
| scaffold1084_1115 | A | C | A | A<->C | intergenic |
| scaffold1187_737 | C | C | A | C<->A | intergenic |
| scaffold1192_10264 | A | C | A | A<->C | intergenic |
| scaffold119_32530 | A | C | A | A<->C | intergenic |
| scaffold1259_4703 | A | C | A | A<->C | intergenic |
| scaffold155_4314 | C | C | A | C<->A | intergenic |
| scaffold1603_1051 | A | C | A | A<->C | intergenic |
| scaffold1603_651 | A | C | A | A<->C | intergenic |
| scaffold1629_4714 | C | C | A | C<->A | intergenic |
| scaffold175_40802 | C | C | A | C<->A | intergenic |
| scaffold1847_4922 | A | C | A | A<->C | intergenic |
| scaffold2049_3761 | C | C | A | C<->A | intergenic |
| scaffold2139_2972 | A | C | A | A<->C | intergenic |
| scaffold2319_1558 | A | C | A | A<->C | intergenic |
| scaffold2319_1706 | A | C | A | A<->C | intergenic |
| scaffold25_63501 | A | C | A | A<->C | intergenic |
| scaffold274_32039 | A | C | A | A<->C | intergenic |
| scaffold409_22339 | A | C | A | A<->C | intergenic |
| scaffold63_6085 | T | C | A | T<->CA | intergenic |
| scaffold693_7570 | A | C | A | A<->C | intergenic |
| scaffold707_2306 | C | C | A | C<->A | intergenic |
| scaffold717_1156 | A | C | A | A<->C | intergenic |
| scaffold72_2908 | A | C | A | A<->C | intergenic |
| scaffold940_2289 | A | C | A | A<->C | intergenic |
| scaffold940_3617 | A | C | A | A<->C | intergenic |
| scaffold949_4745 | C | C | A | C<->A | intergenic |
| scaffold977_1391 | C | C | A | C<->A | intergenic |
| scaffold98_7999 | A | C | A | A<->C | intergenic |

Table S5-4: SNPs in coding sequences between ZW2 and ZW1

| Reference genome | ref | ZW2 | ZW1 | ref<->sample | proPos | ref<->sample | ref<->sample | mutate_type | ref_gene_ID | ref_gene_product | Classification |
| --- | --- | --- | --- | --- | --- | --- | --- | --- | --- | --- | --- |
| scaffold307_22404 | T | T | C | T<->C | 506 | GAT<->GGT | D<->G | nonsyn | scaffold307.t7.cds1 | Acyl-CoA dehydrogenase family member 11 [Hondaea fermentalgiana] | Lipid metabolism |
| scaffold478_3182 | C | T | C | C<->T | 280 | CGC<->CAC | R<->H | nonsyn | scaffold478.t2.cds1 | Phosphatidylinositol/phosphatidylcholine transfer protein SFH3 | Lipid metabolism |
| scaffold351_3089 | G | G | C | G<->C | 5 | GCG<->GGG | A<->G | nonsyn | scaffold351.t2.cds1 | Predicted esterase | Lipid metabolism |
| scaffold1624_5616 | T | C | T | T<->C | 273 | ATT<->GTT | I<->V | nonsyn | scaffold1624.t2.cds1 | polyunsaturated fatty acid synthase subunit C | Lipid metabolism |
| scaffold18_62175 | A | A | G | A<->G | 249 | TCG<->CCG | S<->P | nonsyn | scaffold18.t23.cds1 | Monoacylglycerol lipase ABHD12 | Lipid metabolism |
| scaffold483_2583 | A | A | G | A<->G | 147 | GAT<->GGT | D<->G | nonsyn | scaffold483.t2.cds1 | Glycerol-3-phosphate phosphatase | Lipid metabolism |
| scaffold602_16331 | A | A | G | A<->G | 58 | GAT<->GGT | D<->G | nonsyn | scaffold602.t8.cds1 | Phosphatidylserine decarboxylase | Lipid metabolism |
| scaffold880_9396 | A | A | G | A<->G | 165 | TCA<->CCA | S<->P | nonsyn | scaffold880.t4.cds2 | Phospholipase A1 | Lipid metabolism |
| scaffold1061_11008 | T | T | G | T<->G | 640 | GAT<->GAG | D<->E | nonsyn | scaffold1061.t2.cds1 | Nuclear pore complex protein Nup93 [Hondaea fermentalgiana] | Translation |
| scaffold119_180 | C | T | C | C<->T | 12 | GCT<->GTT | A<->V | nonsyn | scaffold119.t1.cds1 | 40S ribosomal protein S3a [Hondaea fermentalgiana] | Translation |
| scaffold14_63982 | G | G | C | G<->C | 7 | GCG<->GGG | A<->G | nonsyn | scaffold14.t19.cds1 | Elongation factor G, mitochondrial [Hondaea fermentalgiana] | Translation |
| scaffold479_16877 | G | G | C | G<->C | 19 | GCC<->GGC | A<->G | nonsyn | scaffold479.t5.cds1 | Histidine--tRNA ligase, cytoplasmic [Hondaea fermentalgiana] | Translation |
| scaffold562_11165 | C | C | G | C<->G | 208 | CGC<->CCC | R<->P | nonsyn | scaffold562.t4.cds1 | Phenylalanine--tRNA ligase beta subunit [Hondaea fermentalgiana] | Translation |
| scaffold149_26664 | G | A | G | G<->A | 143 | CAC<->TAC | H<->Y | nonsyn | scaffold149.t9.cds1 | Importin subunit alpha [Hondaea fermentalgiana] | Translation |
| scaffold1051_11553 | T | C | T | T<->C | 170 | GAT<->GGT | D<->G | nonsyn | scaffold1051.t3.cds1 | Nucleolar protein 14 [Hondaea fermentalgiana] | Translation |
| scaffold4_70793 | C | T | C | C<->T | 167 | CAT<->TAT | H<->Y | nonsyn | scaffold4.t23.cds1 | Histidine triad nucleotide-binding protein 1 [Hondaea fermentalgiana] | Translation |
| scaffold126_38476 | C | G | C | C<->G | 5 | CGG<->GGG | R<->G | nonsyn | scaffold126.t13.cds1 | Histone-lysine N-methyltransferase ATXR3 [Hondaea fermentalgiana] | Translation |
| scaffold184_37170 | T | T | C | T<->C | 671 | GAC<->GGC | D<->G | nonsyn | scaffold184.t11.cds1 | YTH domain-containing family protein 2 [Hondaea fermentalgiana] | Transcription |
| scaffold1932_1696 | T | T | C | T<->C | 154 | TTG<->TCG | L<->S | nonsyn | scaffold1932.t1.cds2 | Myelin regulatory factor [Hondaea fermentalgiana] | Transcription |
| scaffold1932_1977 | T | T | C | T<->C | 2 | TTG<->TCG | L<->S | nonsyn | scaffold1932.t1.cds3 | Myelin regulatory factor [Hondaea fermentalgiana] | Transcription |
| scaffold1932_2033 | T | T | C | T<->C | 21 | TTC<->CTC | F<->L | nonsyn | scaffold1932.t1.cds3 | Myelin regulatory factor [Hondaea fermentalgiana] | Transcription |
| scaffold286_14696 | T | T | A | T<->A | 35 | ACC<->TCC | T<->S | nonsyn | scaffold286.t5.cds1 | Transcription factor MYB44 [Hondaea fermentalgiana] | Transccription |
| scaffold8_3947 | T | T | C | T<->C | 97 | GAT<->GGT | D<->G | nonsyn | scaffold8.t2.cds1 | ATP-dependent RNA helicase dbp4 [Hondaea fermentalgiana] | Transcription |
| scaffold1932_1291 | A | G | A | A<->G | 19 | CAA<->CGA | Q<->R | nonsyn | scaffold1932.t1.cds2 | Myelin regulatory factor [Hondaea fermentalgiana] | Transcription |
| scaffold1932_1660 | A | G | A | A<->G | 142 | GAG<->GGG | E<->G | nonsyn | scaffold1932.t1.cds2 | Myelin regulatory factor [Hondaea fermentalgiana] | Transcription |
| scaffold1932_2042 | G | G | C | G<->C | 24 | GCT<->CCT | A<->P | nonsyn | scaffold1932.t1.cds3 | Myelin regulatory factor [Hondaea fermentalgiana] | Transcription |
| scaffold1932_257 | G | G | A | G<->A | 86 | AGC<->AAC | S<->N | nonsyn | scaffold1932.t1.cds1 | Myelin regulatory factor [Hondaea fermentalgiana] | Transcription |
| scaffold1932_2702 | G | G | C | G<->C | 244 | GCT<->CCT | A<->P | nonsyn | scaffold1932.t1.cds3 | Myelin regulatory factor [Hondaea fermentalgiana] | Transcription |
| scaffold277_20022 | G | G | C | G<->C | 10 | GCG<->GGG | A<->G | nonsyn | scaffold277.t6.cds1 | Cleavage and polyadenylation specificity factor subunit 3 [Hondaea fermentalgiana] | Transcription |
| scaffold168_38810 | A | C | A | A<->C | 209 | ATG<->CTG | M<->L | nonsyn | scaffold168.t13.cds1 | Transcription initiation factor TFIID subunit 6 [Hondaea fermentalgiana] | Transcription |
| scaffold1932_1687 | A | A | G | A<->G | 151 | CAA<->CGA | Q<->R | nonsyn | scaffold1932.t1.cds2 | Myelin regulatory factor [Hondaea fermentalgiana] | Transcription |
| scaffold1932_2712 | A | A | G | A<->G | 247 | AAC<->AGC | N<->S | nonsyn | scaffold1932.t1.cds3 | Myelin regulatory factor [Hondaea fermentalgiana] | Transcription |
| scaffold1286_8667 | C | T | C | C<->T | 364 | TGG<->TGA | W<->X | nonsense | scaffold1286.t2.cds2 | Disintegrin and metalloproteinase domain-containing protein B [Hondaea fermentalgiana] | Signal transduction |
| scaffold140_30369 | T | T | C | T<->C | 93 | ATT<->ACT | I<->T | nonsyn | scaffold140.t8.cds7 | Nucleotide-binding oligomerization domain-containing protein 1 [Hondaea fermentalgiana] | Signal transduction |
| scaffold1800_5211 | T | T | C | T<->C | 21 | ACG<->GCG | T<->A | nonsyn | scaffold1800.t3.cds1 | Adenylate kinase [Hondaea fermentalgiana] | Signal transduction |
| scaffold27_38422 | C | T | C | C<->T | 133 | CGG<->CAG | R<->Q | nonsyn | scaffold27.t1.cds1 | Hepatocyte growth factor receptor [Hondaea fermentalgiana] | Signal transduction |
| scaffold48_8175 | C | T | C | C<->T | 519 | ACC<->ATC | T<->I | nonsyn | scaffold48.t2.cds1 | Cilia- and flagella-associated protein 221 [Hondaea fermentalgiana] | Signal transduction |
| scaffold566_7486 | C | T | C | C<->T | 1988 | GAT<->AAT | D<->N | nonsyn | scaffold566.t4.cds1 | Cilia- and flagella-associated protein 46 [Hondaea fermentalgiana] | Signal transduction |
| scaffold607_2375 | A | C | A | A<->C | 65 | CAG<->CCG | Q<->P | nonsyn | scaffold607.t1.cds1 | Cyclic AMP-responsive element-binding protein 1 [Hondaea fermentalgiana] | Signal transduction |
| scaffold1131_167 | A | A | G | A<->G | 47 | GAG<->GGG | E<->G | nonsyn | scaffold1131.t1.cds1 | Phosphatidylinositol 4-phosphate 5-kinase 1 [Hondaea fermentalgiana] | Signal transduction |
| scaffold1322_6859 | G | A | G | G<->A | 808 | GGG<->GAG | G<->E | nonsyn | scaffold1322.t1.cds1 | Regulator of G-protein signaling 13 [Hondaea fermentalgiana] | Signal transduction |
| scaffold133_25905 | G | A | G | G<->A | 2673 | TGG<->TGA | W<->X | nonsense | scaffold133.t5.cds2 | Serine/threonine-protein kinase mTOR [Hondaea fermentalgiana] | Signal transduction |
| scaffold1657_1475 | A | A | G | A<->G | 413 | GAA<->GGA | E<->G | nonsyn | scaffold1657.t1.cds1 | Disintegrin and metalloproteinase domain-containing protein B [Hondaea fermentalgiana] | Signal transduction |
| scaffold2399_1063 | G | G | C | G<->C | 57 | CAG<->GAG | Q<->E | nonsyn | scaffold2399.t1.cds1 | Calpain-like protein [Hondaea fermentalgiana] | Protein folding, sorting and degradation |
| scaffold2399_1096 | G | G | A | G<->A | 46 | CAC<->TAC | H<->Y | nonsyn | scaffold2399.t1.cds1 | Calpain-like protein [Hondaea fermentalgiana] | Protein folding, sorting and degradation |
| scaffold2399_1108 | G | G | A | G<->A | 42 | CAA<->TAA | Q<->X | nonsense | scaffold2399.t1.cds1 | Calpain-like protein [Hondaea fermentalgiana] | Protein folding, sorting and degradation |
| scaffold2399_772 | G | G | A | G<->A | 154 | CAA<->TAA | Q<->X | nonsense | scaffold2399.t1.cds1 | Calpain-like protein [Hondaea fermentalgiana] | Protein folding, sorting and degradation |
| scaffold909_156 | C | G | C | C<->G | 39 | GCC<->GGC | A<->G | nonsyn | scaffold909.t1.cds1 | Zinc metalloproteinase-disintegrin-like [Hondaea fermentalgiana] | Protein folding, sorting and degradation |
| scaffold2399_1033 | C | C | A | C<->A | 67 | GAT<->TAT | D<->Y | nonsyn | scaffold2399.t1.cds1 | Calpain-like protein [Hondaea fermentalgiana] | Protein folding, sorting and degradation |
| scaffold2399_741 | C | C | T | C<->T | 164 | GGG<->GAG | G<->E | nonsyn | scaffold2399.t1.cds1 | Calpain-like protein [Hondaea fermentalgiana] | Protein folding, sorting and degradation |
| scaffold826_1065 | C | C | G | C<->G | 34 | GCG<->GGG | A<->G | nonsyn | scaffold826.t1.cds1 | ATP-dependent Clp protease ATP-binding subunit ClpX [Hondaea fermentalgiana] | Protein folding, sorting and degradation |
| scaffold561_4752 | A | A | T | A<->T | 377 | GAC<->GTC | D<->V | nonsyn | scaffold561.t2.cds1 | Signal recognition particle subunit SRP68 [Hondaea fermentalgiana] | Protein folding, sorting and degradation |
| scaffold796_4019 | A | A | C | A<->C | 344 | ATG<->AGG | M<->R | nonsyn | scaffold796.t1.cds1 | E3 SUMO-protein ligase PIAS2 [Hondaea fermentalgiana] | Protein folding, sorting and degradation |
| scaffold1825_3668 | T | C | T | T<->C | 42 | ATC<->GTC | I<->V | nonsyn | scaffold1825.t2.cds1 | 26S proteasome regulatory subunit 6A-like [Hondaea fermentalgiana] | Protein folding, sorting and degradation |
| scaffold1464_6883 | C | T | C | C<->T | 51 | CCT<->TCT | P<->S | nonsyn | scaffold1464.t4.cds2 | Kinesin light chain [Hondaea fermentalgiana] | Cytoskeleton proteins |
| scaffold1464_7072 | C | T | C | C<->T | 114 | CGT<->TGT | R<->C | nonsyn | scaffold1464.t4.cds2 | Kinesin light chain [Hondaea fermentalgiana] | Cytoskeleton proteins |
| scaffold2104_1093 | C | T | C | C<->T | 19 | TGC<->TAC | C<->Y | nonsyn | scaffold2104.t1.cds2 | Kinesin light chain 1 [Hondaea fermentalgiana] | Cytoskeleton proteins |
| scaffold2319_990 | T | T | G | T<->G | 90 | GAA<->GAC | E<->D | nonsyn | scaffold2319.t1.cds1 | Kinesin light chain 3 [Hondaea fermentalgiana] | Cytoskeleton proteins |
| scaffold2394_618 | G | T | G | G<->T | 101 | GCT<->TCT | A<->S | nonsyn | scaffold2394.t1.cds1 | Kinesin light chain 3 [Hondaea fermentalgiana] | Cytoskeleton proteins |
| scaffold54_39262 | C | T | C | C<->T | 645 | CAC<->TAC | H<->Y | nonsyn | scaffold54.t7.cds2 | Dynein heavy chain 6, axonemal [Hondaea fermentalgiana] | Cytoskeleton proteins |
| scaffold694_17181 | G | T | G | G<->T | 53 | GTT<->TTT | V<->F | nonsyn | scaffold694.t4.cds1 | Kinesin light chain 3 [Hondaea fermentalgiana] | Cytoskeleton proteins |
| scaffold721_970 | T | T | C | T<->C | 94 | AAA<->GAA | K<->E | nonsyn | scaffold721.t2.cds1 | Kinesin light chain 1 [Hondaea fermentalgiana] | Cytoskeleton proteins |
| scaffold1464_6851 | T | G | T | T<->G | 40 | TTT<->TGT | F<->C | nonsyn | scaffold1464.t4.cds2 | Kinesin light chain [Hondaea fermentalgiana] | Cytoskeleton proteins |
| scaffold1464_6913 | C | G | C | C<->G | 61 | CCA<->GCA | P<->A | nonsyn | scaffold1464.t4.cds2 | Kinesin light chain [Hondaea fermentalgiana] | Cytoskeleton proteins |
| scaffold2307_699 | A | G | A | A<->G | 137 | GTT<->GCT | V<->A | nonsyn | scaffold2307.t1.cds1 | Kinesin light chain 3 [Hondaea fermentalgiana] | Cytoskeleton proteins |
| scaffold2319_246 | T | G | T | T<->G | 338 | GAA<->GAC | E<->D | nonsyn | scaffold2319.t1.cds1 | Kinesin light chain 3 [Hondaea fermentalgiana] | Cytoskeleton proteins |
| scaffold2450_755 | G | G | A | G<->A | 187 | GGG<->AGG | G<->R | nonsyn | scaffold2450.t1.cds1 | Kinesin light chain 3 [Hondaea fermentalgiana] | Cytoskeleton proteins |
| scaffold2450_816 | G | G | T | G<->T | 207 | CGT<->CTT | R<->L | nonsyn | scaffold2450.t1.cds1 | Kinesin light chain 3 [Hondaea fermentalgiana] | Cytoskeleton proteins |
| scaffold2450_966 | G | G | A | G<->A | 257 | GGC<->GAC | G<->D | nonsyn | scaffold2450.t1.cds1 | Kinesin light chain 3 [Hondaea fermentalgiana] | Cytoskeleton proteins |
| scaffold694_17124 | C | G | C | C<->G | 34 | CAA<->GAA | Q<->E | nonsyn | scaffold694.t4.cds1 | Kinesin light chain 3 [Hondaea fermentalgiana] | Cytoskeleton proteins |
| scaffold694_17343 | A | G | A | A<->G | 107 | AAC<->GAC | N<->D | nonsyn | scaffold694.t4.cds1 | Kinesin light chain 3 [Hondaea fermentalgiana] | Cytoskeleton proteins |
| scaffold91_44636 | C | G | C | C<->G | 58 | GTG<->CTG | V<->L | nonsyn | scaffold91.t15.cds2 | Coronin-like protein [Hondaea fermentalgiana] | Cytoskeleton proteins |
| scaffold1321_3904 | C | C | T | C<->T | 52 | ACA<->ATA | T<->I | nonsyn | scaffold1321.t1.cds3 | Kinesin light chain 1 [Hondaea fermentalgiana] | Cytoskeleton proteins |
| scaffold1464_6826 | T | C | T | T<->C | 32 | TGG<->CGG | W<->R | nonsyn | scaffold1464.t4.cds2 | Kinesin light chain [Hondaea fermentalgiana] | Cytoskeleton proteins |
| scaffold1464_6865 | A | C | A | A<->C | 45 | AAA<->CAA | K<->Q | nonsyn | scaffold1464.t4.cds2 | Kinesin light chain [Hondaea fermentalgiana] | Cytoskeleton proteins |
| scaffold1464_7112 | A | C | A | A<->C | 127 | CAA<->CCA | Q<->P | nonsyn | scaffold1464.t4.cds2 | Kinesin light chain [Hondaea fermentalgiana] | Cytoskeleton proteins |
| scaffold2319_623 | C | C | T | C<->T | 213 | GGA<->AGA | G<->R | nonsyn | scaffold2319.t1.cds1 | Kinesin light chain 3 [Hondaea fermentalgiana] | Cytoskeleton proteins |
| scaffold2394_543 | G | C | G | G<->C | 76 | GCG<->CCG | A<->P | nonsyn | scaffold2394.t1.cds1 | Kinesin light chain 3 [Hondaea fermentalgiana] | Cytoskeleton proteins |
| scaffold2450_828 | C | C | A | C<->A | 211 | TCC<->TAC | S<->Y | nonsyn | scaffold2450.t1.cds1 | Kinesin light chain 3 [Hondaea fermentalgiana] | Cytoskeleton proteins |
| scaffold44_17649 | A | C | A | A<->C | 4229 | GAT<->GAG | D<->E | nonsyn | scaffold44.t7.cds1 | Dynein heavy chain 1, axonemal [Hondaea fermentalgiana] | Cytoskeleton proteins |
| scaffold721_584 | C | C | T | C<->T | 101 | GGA<->AGA | G<->R | nonsyn | scaffold721.t1.cds1 | Kinesin light chain 3 [Hondaea fermentalgiana] | Cytoskeleton proteins |
| scaffold1464_6796 | C | A | C | C<->A | 22 | CTA<->ATA | L<->I | nonsyn | scaffold1464.t4.cds2 | Kinesin light chain [Hondaea fermentalgiana] | Cytoskeleton proteins |
| scaffold1464_7151 | A | A | G | A<->G | 140 | TAC<->TGC | Y<->C | nonsyn | scaffold1464.t4.cds2 | Kinesin light chain [Hondaea fermentalgiana] | Cytoskeleton proteins |
| scaffold1765_4936 | A | A | T | A<->T | 125 | ATT<->TTT | I<->F | nonsyn | scaffold1765.t2.cds1 | Kinesin light chain 3 [Hondaea fermentalgiana] | Cytoskeleton proteins |
| scaffold186_34007 | G | A | G | G<->A | 2246 | ATG<->ATA | M<->I | nonsyn | scaffold186.t7.cds1 | Myosin-6 [Hondaea fermentalgiana] | Cytoskeleton proteins |
| scaffold2104_1069 | G | A | G | G<->A | 27 | CCT<->CTT | P<->L | nonsyn | scaffold2104.t1.cds2 | Kinesin light chain 1 [Hondaea fermentalgiana] | Cytoskeleton proteins |
| scaffold2307_927 | T | A | T | T<->A | 61 | TAC<->TTC | Y<->F | nonsyn | scaffold2307.t1.cds1 | Kinesin light chain 3 [Hondaea fermentalgiana] | Cytoskeleton proteins |
| scaffold2307_937 | T | A | T | T<->A | 58 | AGT<->TGT | S<->C | nonsyn | scaffold2307.t1.cds1 | Kinesin light chain 3 [Hondaea fermentalgiana] | Cytoskeleton proteins |
| scaffold2319_865 | G | A | G | G<->A | 132 | GCC<->GTC | A<->V | nonsyn | scaffold2319.t1.cds1 | Kinesin light chain 3 [Hondaea fermentalgiana] | Cytoskeleton proteins |
| scaffold2394_524 | C | A | C | C<->A | 69 | GAC<->GAA | D<->E | nonsyn | scaffold2394.t1.cds1 | Kinesin light chain 3 [Hondaea fermentalgiana] | Cytoskeleton proteins |
| scaffold2450_795 | T | A | T | T<->A | 200 | GTG<->GAG | V<->E | nonsyn | scaffold2450.t1.cds1 | Kinesin light chain 3 [Hondaea fermentalgiana] | Cytoskeleton proteins |
| scaffold2450_818 | G | A | G | G<->A | 208 | GAG<->AAG | E<->K | nonsyn | scaffold2450.t1.cds1 | Kinesin light chain 3 [Hondaea fermentalgiana] | Cytoskeleton proteins |
| scaffold349_10619 | A | A | G | A<->G | 2496 | GAT<->GGT | D<->G | nonsyn | scaffold349.t2.cds1 | Dynein heavy chain 10, axonemal [Hondaea fermentalgiana] | Cytoskeleton proteins |
| scaffold349_8761 | G | A | G | G<->A | 1877 | GGG<->AGG | G<->R | nonsyn | scaffold349.t2.cds1 | Dynein heavy chain 10, axonemal [Hondaea fermentalgiana] | Cytoskeleton proteins |
| scaffold694_17286 | A | A | G | A<->G | 88 | AAA<->GAA | K<->E | nonsyn | scaffold694.t4.cds1 | Kinesin light chain 3 [Hondaea fermentalgiana] | Cytoskeleton proteins |
| scaffold721_964 | A | A | C | A<->C | 96 | TTG<->GTG | L<->V | nonsyn | scaffold721.t2.cds1 | Kinesin light chain 1 [Hondaea fermentalgiana] | Cytoskeleton proteins |
| scaffold162_17974 | A | T | A | A<->T | 1749 | ACC<->TCC | T<->S | nonsyn | scaffold162.t6.cds1 | Myosin-6 [Hondaea fermentalgiana] | Cytoskeleton proteins |
| scaffold1510_4124 | C | T | C | C<->T | 861 | GCA<->ACA | A<->T | nonsyn | scaffold1510.t2.cds1 | UDP-N-acetylglucosamine--peptide N-acetylglucosaminyltransferase 110 kDa subunit [Hondaea ferment | Glycan biosynthesis and metabolism |
| scaffold2043_2471 | C | T | C | C<->T | 428 | GTG<->ATG | V<->M | nonsyn | scaffold2043.t1.cds1 | Alpha-glucosidase [Hondaea fermentalgiana] | Glycan biosynthesis and metabolism |
| scaffold384_13165 | T | T | C | T<->C | 1187 | TTA<->TCA | L<->S | nonsyn | scaffold384.t1.cds2 | GREB1-like protein [Hondaea fermentalgiana] | Glycan biosynthesis and metabolism |
| scaffold384_14820 | C | T | C | C<->T | 60 | CAG<->TAG | Q<->X | nonsense | scaffold384.t1.cds3 | GREB1-like protein [Hondaea fermentalgiana] | Glycan biosynthesis and metabolism |
| scaffold384_14854 | C | T | C | C<->T | 71 | ACG<->ATG | T<->M | nonsyn | scaffold384.t1.cds3 | GREB1-like protein [Hondaea fermentalgiana] | Glycan biosynthesis and metabolism |
| scaffold396_1756 | G | T | G | G<->T | 559 | GAC<->TAC | D<->Y | nonsyn | scaffold396.t1.cds1 | Beta-hexosaminidase subunit beta [Hondaea fermentalgiana] | Glycan biosynthesis and metabolism |
| scaffold396_2755 | C | T | C | C<->T | 892 | CTT<->TTT | L<->F | nonsyn | scaffold396.t1.cds1 | Beta-hexosaminidase subunit beta [Hondaea fermentalgiana] | Glycan biosynthesis and metabolism |
| scaffold396_2773 | G | T | G | G<->T | 898 | GAC<->TAC | D<->Y | nonsyn | scaffold396.t1.cds1 | Beta-hexosaminidase subunit beta [Hondaea fermentalgiana] | Glycan biosynthesis and metabolism |
| scaffold384_13194 | G | G | A | G<->A | 1197 | GTT<->ATT | V<->I | nonsyn | scaffold384.t1.cds2 | GREB1-like protein [Hondaea fermentalgiana] | Glycan biosynthesis and metabolism |
| scaffold396_1078 | T | G | T | T<->G | 333 | TAC<->GAC | Y<->D | nonsyn | scaffold396.t1.cds1 | Beta-hexosaminidase subunit beta [Hondaea fermentalgiana] | Glycan biosynthesis and metabolism |
| scaffold396_1645 | A | G | A | A<->G | 522 | ACC<->GCC | T<->A | nonsyn | scaffold396.t1.cds1 | Beta-hexosaminidase subunit beta [Hondaea fermentalgiana] | Glycan biosynthesis and metabolism |
| scaffold396_1901 | G | G | A | G<->A | 607 | CGG<->CAG | R<->Q | nonsyn | scaffold396.t1.cds1 | Beta-hexosaminidase subunit beta [Hondaea fermentalgiana] | Glycan biosynthesis and metabolism |
| scaffold396_545 | G | G | A | G<->A | 155 | CGG<->CAG | R<->Q | nonsyn | scaffold396.t1.cds1 | Beta-hexosaminidase subunit beta [Hondaea fermentalgiana] | Glycan biosynthesis and metabolism |
| scaffold158_10739 | T | C | T | T<->C | 1328 | ATC<->GTC | I<->V | nonsyn | scaffold158.t3.cds2 | Sushi, von Willebrand factor type A, EGF and pentraxin domain-containing protein 1 | Glycan biosynthesis and metabolism |
| scaffold364_5366 | A | C | A | A<->C | 538 | GAT<->GAG | D<->E | nonsyn | scaffold364.t3.cds1 | Alpha-N-acetylglucosaminidase [Hondaea fermentalgiana] | Glycan biosynthesis and metabolism |
| scaffold175_36506 | G | A | G | G<->A | 560 | ACC<->ATC | T<->I | nonsyn | scaffold175.t12.cds1 | D-inositol-3-phosphate glycosyltransferase [Hondaea fermentalgiana] | Glycan biosynthesis and metabolism |
| scaffold22_44623 | A | A | G | A<->G | 188 | GAT<->GGT | D<->G | nonsyn | scaffold22.t15.cds1 | GREB1-like protein [Hondaea fermentalgiana] | Glycan biosynthesis and metabolism |
| scaffold384_13153 | A | A | T | A<->T | 1183 | GAT<->GTT | D<->V | nonsyn | scaffold384.t1.cds2 | GREB1-like protein [Hondaea fermentalgiana] | Glycan biosynthesis and metabolism |
| scaffold396_1405 | G | A | G | G<->A | 442 | GAT<->AAT | D<->N | nonsyn | scaffold396.t1.cds1 | Beta-hexosaminidase subunit beta [Hondaea fermentalgiana] | Glycan biosynthesis and metabolism |
| scaffold396_1804 | G | C | G | G<->C | 575 | GCA<->CCA | A<->P | nonsyn | scaffold396.t1.cds1 | Beta-hexosaminidase subunit beta [Hondaea fermentalgiana] | Glycan biosynthesis and metabolism |
| scaffold2130_2323 | T | T | C | T<->C | 96 | GAT<->GGT | D<->G | nonsyn | scaffold2130.t1.cds1 | ATP-binding cassette sub-family D member 1 [Hondaea fermentalgiana] | Transport |
| scaffold562_12857 | T | T | C | T<->C | 72 | ATC<->ACC | I<->T | nonsyn | scaffold562.t5.cds1 | Sideroflexin-5 [Hondaea fermentalgiana] | Transport |
| scaffold617_2991 | C | T | C | C<->T | 35 | CGC<->CAC | R<->H | nonsyn | scaffold617.t1.cds1 | Vacuolar cation/proton exchanger 3 [Hondaea fermentalgiana] | Transport |
| scaffold451_17926 | A | G | A | A<->G | 1055 | GAT<->GGT | D<->G | nonsyn | scaffold451.t6.cds1 | Ectopic P granules protein 5-like [Hondaea fermentalgiana] | Transport |
| scaffold591_18313 | A | G | A | A<->G | 493 | TCC<->CCC | S<->P | nonsyn | scaffold591.t7.cds1 | Trafficking protein particle complex subunit 10 [Hondaea fermentalgiana] | Transport |
| scaffold59_6997 | T | G | C | T<->GC | 427 | GAT<->GAG\|GAC | D<->E\|D | nonsyn\|syn | scaffold59.t2.cds2 | Potassium voltage-gated channel subfamily H member 6 [Hondaea fermentalgiana] | Transport |
| scaffold1052_5807 | C | C | G | C<->G | 35 | CGG<->GGG | R<->G | nonsyn | scaffold1052.t3.cds3 | Mitochondrial 2-oxodicarboxylate carrier 2 [Hondaea fermentalgiana] | Transport |
| scaffold1105_2280 | A | C | A | A<->C | 161 | ATG<->AGG | M<->R | nonsyn | scaffold1105.t1.cds2 | Potassium voltage-gated channel subfamily H member 6 [Hondaea fermentalgiana] | Transport |
| scaffold812_14583 | C | C | G | C<->G | 499 | GCC<->CCC | A<->P | nonsyn | scaffold812.t4.cds1 | Brefeldin A-inhibited guanine nucleotide-exchange protein 1 [Hondaea fermentalgiana] | Transport |
| scaffold1918_2715 | G | A | G | G<->A | 465 | CAG<->TAG | Q<->X | nonsense | scaffold1918.t2.cds2 | ABC transporter ATP-binding protein [Hondaea fermentalgiana] | Transport |
| scaffold249_14062 | A | A | T | A<->T | 1534 | GAT<->GTT | D<->V | nonsyn | scaffold249.t2.cds1 | Exocyst complex component SEC5A [Hondaea fermentalgiana] | Transport |
| scaffold336_20354 | A | A | T | A<->T | 188 | ATC<->AAC | I<->N | nonsyn | scaffold336.t9.cds1 | Aquaporin-3 [Hondaea fermentalgiana] | Transport |
| scaffold482_3750 | A | A | T | A<->T | 1023 | GTC<->GAC | V<->D | nonsyn | scaffold482.t2.cds1 | ABC transporter ATP-binding protein [Hondaea fermentalgiana] | Transport |
| scaffold63_30509 | G | A | G | G<->A | 241 | CAA<->TAA | Q<->X | nonsense | scaffold63.t15.cds1 | Sodium channel protein type 11 subunit alpha [Hondaea fermentalgiana] | Transport |
| scaffold965_9414 | C | T | C | C<->T | 3052 | CCG<->TCG | P<->S | nonsyn | scaffold965.t1.cds1 | Neuroblastoma-amplified sequence [Hondaea fermentalgiana] | Transport |
| scaffold965_9451 | G | A | G | G<->A | 3064 | CGG<->CAG | R<->Q | nonsyn | scaffold965.t1.cds1 | Neuroblastoma-amplified sequence [Hondaea fermentalgiana] | Transport |
| scaffold1710_3576 | C | T | C | C<->T | 261 | CCA<->CTA | P<->L | nonsyn | scaffold1710.t2.cds3 | Cysteine desulfurase, mitochondrial [Hondaea fermentalgiana] | Biosynthesis of cofactors |
| scaffold267_291 | C | C | T | C<->T | 6 | GCG<->GTG | A<->V | nonsyn | scaffold267.t1.cds1 | 2-methoxy-6-polyprenyl-1,4-benzoquinol methylase | Biosynthesis of cofactors |
| scaffold475_355 | G | T | G | G<->T | 84 | GGA<->TGA | G<->X | nonsense | scaffold475.t1.cds1 | NAD-dependent deacetylase sir2E [Hondaea fermentalgiana] | Nicotinate and nicotinamide metabolism |
| scaffold1751_1518 | C | C | T | C<->T | 14 | TCA<->TTA | S<->L | nonsyn | scaffold1751.t1.cds1 | Sterile alpha and TIR motif-containing protein tir-1 [Hondaea fermentalgiana] | Nicotinate and nicotinamide metabolism |
| scaffold1751_1674 | A | G | A | A<->G | 66 | CAC<->CGC | H<->R | nonsyn | scaffold1751.t1.cds1 | Sterile alpha and TIR motif-containing protein tir-1 [Hondaea fermentalgiana] | Nicotinate and nicotinamide metabolism |
| scaffold974_4386 | C | T | C | C<->T | 80 | GTG<->ATG | V<->M | nonsyn | scaffold974.t3.cds1 | E3 ubiquitin-protein ligase TRIM71 [Hondaea fermentalgiana] | Ubiquitin system |
| scaffold1115_250 | G | G | C | G<->C | 13 | GCC<->CCC | A<->P | nonsyn | scaffold1115.t1.cds1 | E3 ubiquitin-protein ligase BRE1 [Hondaea fermentalgiana] | Ubiquitin system |
| scaffold1250_8955 | C | T | C | C<->T | 243 | CGA<->CAA | R<->Q | nonsyn | scaffold1250.t5.cds1 | Ankyrin repeat domain-containing protein 1 [Hondaea fermentalgiana] | Ubiquitin system |
| scaffold2053_1084 | T | G | T | T<->G | 21 | GAA<->GAC | E<->D | nonsyn | scaffold2053.t1.cds2 | Leucine-rich repeat-containing protein 1 [Hondaea fermentalgiana] | Ubiquitin system |
| scaffold2053_985 | C | G | C | C<->G | 5 | GAA<->CAA | E<->Q | nonsyn | scaffold2053.t1.cds3 | Leucine-rich repeat-containing protein 1 [Hondaea fermentalgiana] | Ubiquitin system |
| scaffold1874_805 | C | G | C | C<->G | 40 | TCC<->TGC | S<->C | nonsyn | scaffold1874.t1.cds1 | Leucine-rich repeat receptor-like protein kinase PEPR1 [Hondaea fermentalgiana] | Ubiquitin system |
| scaffold2411_857 | G | G | C | G<->C | 104 | GCC<->GGC | A<->G | nonsyn | scaffold2411.t1.cds1 | Leucine-rich repeat serine/threonine-protein kinase 1 [Hondaea fermentalgiana] | Ubiquitin system |
| scaffold2053_781 | T | C | T | T<->C | 21 | GAC<->GGC | D<->G | nonsyn | scaffold2053.t1.cds4 | Leucine-rich repeat-containing protein 1 [Hondaea fermentalgiana] | Ubiquitin system |
| scaffold2053_790 | T | C | T | T<->C | 18 | GAC<->GGC | D<->G | nonsyn | scaffold2053.t1.cds4 | Leucine-rich repeat-containing protein 1 [Hondaea fermentalgiana] | Ubiquitin system |
| scaffold822_4930 | G | G | T | G<->T | 729 | GCG<->TCG | A<->S | nonsyn | scaffold822.t1.cds1 | Oxidation resistance protein 1 [Hondaea fermentalgiana] | Oxidation and reduction |
| scaffold228_2799 | C | T | C | C<->T | 231 | ATG<->ATA | M<->I | nonsyn | scaffold228.t2.cds1 | Short chain dehydrogenase family protein [Hondaea fermentalgiana] | Oxidation and reduction |
| scaffold20_73540 | A | G | T | A<->GT | 247 | TCC<->CCC\|ACC | S<->P\|T | nonsyn\|nonsyn | scaffold20.t25.cds1 | Fructose-2,6-bisphosphatase TIGAR [Hondaea fermentalgiana] | Cabohydrate metabolism |
| scaffold1258_2745 | A | A | C | A<->C | 266 | GAT<->GAG | D<->E | nonsyn | scaffold1258.t1.cds2 | Pyruvate, phosphate dikinase [Hondaea fermentalgiana] | Cabohydrate metabolism |
| scaffold151_23428 | C | T | C | C<->T | 133 | GTC<->ATC | V<->I | nonsyn | scaffold151.t6.cds1 | Hypothetical Protein FCC1311_050702 [Hondaea fermentalgiana] | Hypothetical protein |
| scaffold184_35844 | T | T | C | T<->C | 683 | TCC<->CCC | S<->P | nonsyn | scaffold184.t10.cds2 | Hypothetical Protein FCC1311_087122 [Hondaea fermentalgiana] | Hypothetical protein |
| scaffold1964_1646 | C | T | C | C<->T | 214 | ATG<->ATA | M<->I | nonsyn | scaffold1964.t1.cds2 | Hypothetical Protein FCC1311_053442 [Hondaea fermentalgiana] | Hypothetical protein |
| scaffold198_18889 | T | T | G | T<->G | 102 | ATC<->CTC | I<->L | nonsyn | scaffold198.t6.cds1 | Hypothetical Protein FCC1311_031142 [Hondaea fermentalgiana] | Hypothetical protein |
| scaffold1_7753 | T | T | A | T<->A | 112 | GTG<->GAG | V<->E | nonsyn | scaffold1.t2.cds2 | hypothetical protein [Flavobacteriaceae bacterium] | Hypothetical protein |
| scaffold2001_2554 | C | T | C | C<->T | 798 | CGT<->TGT | R<->C | nonsyn | scaffold2001.t1.cds2 | Hypothetical Protein FCC1311_111502 [Hondaea fermentalgiana] | Hypothetical protein |
| scaffold2232_2103 | C | T | C | C<->T | 161 | CCG<->CTG | P<->L | nonsyn | scaffold2232.t1.cds3 | Hypothetical Protein FCC1311_113942 [Hondaea fermentalgiana] | Hypothetical protein |
| scaffold293_9406 | C | T | C | C<->T | 45 | GTA<->ATA | V<->I | nonsyn | scaffold293.t5.cds2 | Hypothetical Protein FCC1311_074832 [Hondaea fermentalgiana] | Hypothetical protein |
| scaffold324_15504 | C | T | C | C<->T | 2438 | GCA<->ACA | A<->T | nonsyn | scaffold324.t5.cds1 | Hypothetical Protein FCC1311_047152 [Hondaea fermentalgiana] | Hypothetical protein |
| scaffold331_9481 | C | T | C | C<->T | 137 | GGC<->AGC | G<->S | nonsyn | scaffold331.t4.cds1 | Hypothetical Protein FCC1311_094012 [Hondaea fermentalgiana] | Hypothetical protein |
| scaffold341_15406 | T | T | C | T<->C | 195 | ACG<->GCG | T<->A | nonsyn | scaffold341.t6.cds1 | Hypothetical Protein FCC1311_066552 [Hondaea fermentalgiana] | Hypothetical protein |
| scaffold341_15448 | T | T | G | T<->G | 181 | AAG<->CAG | K<->Q | nonsyn | scaffold341.t6.cds1 | Hypothetical Protein FCC1311_066552 [Hondaea fermentalgiana] | Hypothetical protein |
| scaffold429_16540 | T | T | G | T<->G | 66 | ATC<->CTC | I<->L | nonsyn | scaffold429.t5.cds1 | Hypothetical Protein FCC1311_029742 [Hondaea fermentalgiana] | Hypothetical protein |
| scaffold470_669 | A | T | A | A<->T | 205 | AAG<->ATG | K<->M | nonsyn | scaffold470.t1.cds1 | Hypothetical Protein FCC1311_113272 [Hondaea fermentalgiana] | Hypothetical protein |
| scaffold516_14422 | T | T | C | T<->C | 1274 | CAC<->CGC | H<->R | nonsyn | scaffold516.t1.cds1 | WD repeat-containing protein wdr-5.1 [Hondaea fermentalgiana] | Hypothetical protein |
| scaffold58_56521 | T | T | C | T<->C | 101 | ACG<->GCG | T<->A | nonsyn | scaffold58.t19.cds1 | Hypothetical Protein FCC1311_062132 [Hondaea fermentalgiana] | Hypothetical protein |
| scaffold758_12986 | T | T | G | T<->G | 501 | TTC<->GTC | F<->V | nonsyn | scaffold758.t5.cds3 | hypothetical protein | Hypothetical protein |
| scaffold863_6521 | T | T | C | T<->C | 18 | GAC<->GGC | D<->G | nonsyn | scaffold863.t2.cds3 | hypothetical protein DB42_EU00010 [Neochlamydia sp. EPS4] | Hypothetical protein |
| scaffold863_6563 | T | T | A | T<->A | 4 | AAG<->ATG | K<->M | nonsyn | scaffold863.t2.cds3 | hypothetical protein DB42_EU00010 [Neochlamydia sp. EPS4] | Hypothetical protein |
| scaffold88_28151 | A | T | A | A<->T | 1020 | CTG<->CAG | L<->Q | nonsyn | scaffold88.t10.cds1 | Hypothetical Protein FCC1311_055362 [Hondaea fermentalgiana] | Hypothetical protein |
| scaffold1021_8718 | A | G | A | A<->G | 140 | GAT<->GGT | D<->G | nonsyn | scaffold1021.t3.cds1 | Hypothetical Protein FCC1311_091452 [Hondaea fermentalgiana] | Hypothetical protein |
| scaffold1117_1541 | A | G | A | A<->G | 305 | CAC<->CGC | H<->R | nonsyn | scaffold1117.t1.cds2 | Protein kinase, putative [Hondaea fermentalgiana] | Hypothetical protein |
| scaffold1117_1736 | A | G | G | A<->G | 370 | CAA<->CGA | Q<->R | nonsyn | scaffold1117.t1.cds2 | Protein kinase, putative [Hondaea fermentalgiana] | Hypothetical protein |
| scaffold1118_2318 | A | G | A | A<->G | 496 | ATG<->GTG | M<->V | nonsyn | scaffold1118.t1.cds1 | WW domain-binding protein 4 [Hondaea fermentalgiana] | Hypothetical protein |
| scaffold150_703 | A | G | A | A<->G | 17 | ATT<->GTT | I<->V | nonsyn | scaffold150.t1.cds1 | Hypothetical Protein FCC1311_104332 [Hondaea fermentalgiana] | Hypothetical protein |
| scaffold150_793 | A | G | A | A<->G | 47 | AAG<->GAG | K<->E | nonsyn | scaffold150.t1.cds1 | Hypothetical Protein FCC1311_104332 [Hondaea fermentalgiana] | Hypothetical protein |
| scaffold2134_2259 | C | G | C | C<->G | 197 | AGT<->ACT | S<->T | nonsyn | scaffold2134.t1.cds1 | Hypothetical Protein FCC1311_113102 [Hondaea fermentalgiana] | Hypothetical protein |
| scaffold2312_400 | A | G | A | A<->G | 117 | GAA<->GGA | E<->G | nonsyn | scaffold2312.t1.cds1 | Hypothetical Protein FCC1311_111472 [Hondaea fermentalgiana] | Hypothetical protein |
| scaffold2312_414 | A | G | A | A<->G | 122 | ATC<->GTC | I<->V | nonsyn | scaffold2312.t1.cds1 | Hypothetical Protein FCC1311_111472 [Hondaea fermentalgiana] | Hypothetical protein |
| scaffold325_23335 | A | G | A | A<->G | 1 | ATG<->GTG | M<->V | start_mutation | scaffold325.t13.cds1 | Hypothetical Protein FCC1311_106632 [Hondaea fermentalgiana] | Hypothetical protein |
| scaffold35_17419 | G | G | A | G<->A | 64 | GTC<->ATC | V<->I | nonsyn | scaffold35.t4.cds1 | Hypothetical Protein FCC1311_028662 [Hondaea fermentalgiana] | Hypothetical protein |
| scaffold467_392 | G | G | A | G<->A | 88 | CGC<->CAC | R<->H | nonsyn | scaffold467.t1.cds1 | hypothetical protein | Hypothetical protein |
| scaffold944_2770 | G | G | A | G<->A | 157 | GTA<->ATA | V<->I | nonsyn | scaffold944.t2.cds1 | Hypothetical Protein FCC1311_052782 [Hondaea fermentalgiana] | Hypothetical protein |
| scaffold1117_1543 | C | C | T | C<->T | 306 | CGT<->TGT | R<->C | nonsyn | scaffold1117.t1.cds2 | Hypothetical Protein FCC1311_066552 [Hondaea fermentalgiana] | Hypothetical protein |
| scaffold1169_2053 | C | C | G | C<->G | 416 | CGG<->GGG | R<->G | nonsyn | scaffold1169.t1.cds1 | Hypothetical Protein FCC1311_054022 [Hondaea fermentalgiana] | Hypothetical protein |
| scaffold1529_5003 | T | C | T | T<->C | 75 | GAC<->GGC | D<->G | nonsyn | scaffold1529.t3.cds1 | hypothetical protein | Hypothetical protein |
| scaffold1620_4601 | T | C | T | T<->C | 32 | AAC<->GAC | N<->D | nonsyn | scaffold1620.t2.cds1 | hypothetical protein | Hypothetical protein |
| scaffold1620_4609 | T | C | T | T<->C | 29 | AAC<->AGC | N<->S | nonsyn | scaffold1620.t2.cds1 | hypothetical protein | Hypothetical protein |
| scaffold17_231 | C | C | A | C<->A | 53 | ACG<->AAG | T<->K | nonsyn | scaffold17.t1.cds1 | hypothetical protein | Hypothetical protein |
| scaffold206_4518 | T | C | T | T<->C | 334 | ATC<->ACC | I<->T | nonsyn | scaffold206.t3.cds1 | Hypothetical Protein FCC1311_046202 [Hondaea fermentalgiana] | Hypothetical protein |
| scaffold2125_298 | C | C | T | C<->T | 289 | CGG<->CAG | R<->Q | nonsyn | scaffold2125.t1.cds1 | Hypothetical Protein FCC1311_113272 [Hondaea fermentalgiana] | Hypothetical protein |
| scaffold242_13643 | A | C | A | A<->C | 124 | ATC<->CTC | I<->L | nonsyn | scaffold242.t6.cds1 | hypothetical protein | Hypothetical protein |
| scaffold286_318 | C | C | A | C<->A | 26 | GCG<->GAG | A<->E | nonsyn | scaffold286.t1.cds1 | Transmembrane protein DDB_G0269096 [Hondaea fermentalgiana] | Hypothetical protein |
| scaffold515_20639 | G | C | G | G<->C | 48 | GTC<->CTC | V<->L | nonsyn | scaffold515.t3.cds1 | Hypothetical Protein FCC1311_100102 [Hondaea fermentalgiana] | Hypothetical protein |
| scaffold58_55076 | T | C | T | T<->C | 127 | CAG<->CGG | Q<->R | nonsyn | scaffold58.t19.cds2 | Hypothetical Protein FCC1311_062132 [Hondaea fermentalgiana] | Hypothetical protein |
| scaffold58_55082 | T | C | T | T<->C | 125 | CAG<->CGG | Q<->R | nonsyn | scaffold58.t19.cds2 | Hypothetical Protein FCC1311_062132 [Hondaea fermentalgiana] | Hypothetical protein |
| scaffold1254_1025 | A | A | C | A<->C | 266 | ATC<->CTC | I<->L | nonsyn | scaffold1254.t1.cds1 | Hypothetical Protein FCC1311_097472 [Hondaea fermentalgiana] | Hypothetical protein |
| scaffold1430_2561 | A | A | G | A<->G | 836 | ATC<->GTC | I<->V | nonsyn | scaffold1430.t1.cds1 | Protein kinase, putative [Hondaea fermentalgiana] | Hypothetical protein |
| scaffold17_197 | A | A | C | A<->C | 42 | ACG<->CCG | T<->P | nonsyn | scaffold17.t1.cds1 | Hypothetical Protein FCC1311_074202 [Hondaea fermentalgiana] | Hypothetical protein |
| scaffold203_22002 | T | A | T | T<->A | 454 | TCT<->ACT | S<->T | nonsyn | scaffold203.t6.cds1 | Hypothetical Protein FCC1311_082682 [Hondaea fermentalgiana] | Hypothetical protein |
| scaffold2049_402 | A | A | G | A<->G | 84 | ATG<->GTG | M<->V | nonsyn | scaffold2049.t1.cds1 | Hypothetical Protein FCC1311_104322 [Hondaea fermentalgiana] | Hypothetical protein |
| scaffold2139_2499 | A | A | T | A<->T | 130 | ACA<->TCA | T<->S | nonsyn | scaffold2139.t1.cds2 | Hypothetical Protein FCC1311_114162 [Hondaea fermentalgiana] | Hypothetical protein |
| scaffold2139_2776 | C | A | C | C<->A | 222 | GCT<->GAT | A<->D | nonsyn | scaffold2139.t1.cds2 | Hypothetical Protein FCC1311_114162 [Hondaea fermentalgiana] | Hypothetical protein |
| scaffold2139_2932 | A | A | G | A<->G | 274 | GAT<->GGT | D<->G | nonsyn | scaffold2139.t1.cds2 | Hypothetical Protein FCC1311_114162 [Hondaea fermentalgiana] | Hypothetical protein |
| scaffold213_8803 | G | A | G | G<->A | 220 | CCA<->CTA | P<->L | nonsyn | scaffold213.t4.cds1 | Hypothetical Protein FCC1311_081792 [Hondaea fermentalgiana] | Hypothetical protein |
| scaffold2312_270 | A | A | G | A<->G | 74 | ATC<->GTC | I<->V | nonsyn | scaffold2312.t1.cds1 | Hypothetical Protein FCC1311_111472 [Hondaea fermentalgiana] | Hypothetical protein |
| scaffold1212_3058 | T | T | C | T<->C | 448 | GAC<->GGC | D<->G | nonsyn | scaffold1212.t2.cds1 | Nucleolar complex protein 4-like [Hondaea fermentalgiana] | Hypothetical protein |
| scaffold196_31811 | C | T | C | C<->T | 217 | GCT<->ACT | A<->T | nonsyn | scaffold196.t10.cds1 | Metabotropic glutamate receptor-like protein E [Hondaea fermentalgiana] | Hypothetical protein |
| scaffold84_32147 | A | A | G | A<->G | 8 | ACG<->GCG | T<->A | nonsyn | scaffold84.t10.cds1 | RAC family serine/threonine-protein kinase-like [Hondaea fermentalgiana] | Hypothetical protein |
| scaffold256_16460 | A | A | G | A<->G | 49 | ATG<->GTG | M<->V | nonsyn | scaffold256.t4.cds2 | Hypothetical Protein FCC1311_103972 [Hondaea fermentalgiana] | Hypothetical protein |
| scaffold341_15553 | T | A | T | T<->A | 146 | AAC<->TAC | N<->Y | nonsyn | scaffold341.t6.cds1 | Hypothetical Protein FCC1311_066552 [Hondaea fermentalgiana] | Hypothetical protein |
| scaffold38_1116 | G | A | G | G<->A | 96 | GAC<->AAC | D<->N | nonsyn | scaffold38.t1.cds1 | Hypothetical Protein FCC1311_025702 [Hondaea fermentalgiana] | Hypothetical protein |
| scaffold1049_11579 | C | C | G | C<->G | 147 | GCC<->CCC | A<->P | nonsyn | scaffold1049.t4.cds1 | Hypothetical Protein FCC1311_113272 [Hondaea fermentalgiana] | Hypothetical protein |
| scaffold659_1319 | C | A | T | C<->AT | 60 | ACC<->AAC\|ATC | T<->N\|I | nonsyn\|nonsyn | scaffold659.t1.cds2 | Hypothetical Protein FCC1311_113272 [Hondaea fermentalgiana] | Hypothetical protein |
| scaffold75_20902 | G | A | G | G<->A | 6 | CCG<->TCG | P<->S | nonsyn | scaffold75.t7.cds1 | Protein kinase, putative [Hondaea fermentalgiana] | Hypothetical protein |
| scaffold772_11624 | T | A | T | T<->A | 711 | GTC<->GAC | V<->D | nonsyn | scaffold772.t4.cds1 | Protein kinase, putative [Hondaea fermentalgiana] | Hypothetical protein |
| scaffold838_1690 | A | A | C | A<->C | 461 | CAT<->CCT | H<->P | nonsyn | scaffold838.t1.cds1 | Protein phosphatase, putative [Hondaea fermentalgiana] | Hypothetical protein |
| scaffold944_2708 | T | A | T | T<->A | 136 | ATG<->AAG | M<->K | nonsyn | scaffold944.t2.cds1 | Hypothetical Protein FCC1311_052782 [Hondaea fermentalgiana] | Hypothetical protein |
| scaffold944_2749 | A | A | G | A<->G | 150 | AAG<->GAG | K<->E | nonsyn | scaffold944.t2.cds1 | Hypothetical Protein FCC1311_052782 [Hondaea fermentalgiana] | Hypothetical protein |
| scaffold319_23912 | A | A | G | A<->G | 782 | GAT<->GGT | D<->G | nonsyn | scaffold319.t7.cds1 | Coiled-coil domain-containing protein 180 [Hondaea fermentalgiana] | Hypothetical protein |
| scaffold27_41182 | G | C | G | G<->C | 405 | GCC<->CCC | A<->P | nonsyn | scaffold27.t2.cds1 | Hypothetical Protein FCC1311_013962 [Hondaea fermentalgiana] | Hypothetical protein |
| scaffold1922_2474 | T | C | T | T<->C | 27 | CAG<->CGG | Q<->R | nonsyn | scaffold1922.t2.cds2 | helix-turn-helix domain-containing protein [Hyphomicrobiales bacterium] | Hypothetical protein |
| scaffold476_272 | A | A | G | A<->G | 84 | AAG<->GAG | K<->E | nonsyn | scaffold476.t1.cds1 | Methionine synthase [Hondaea fermentalgiana] | Cobalamin transport and metabolism |
| scaffold493_8876 | A | A | G | A<->G | 935 | ATC<->GTC | I<->V | nonsyn | scaffold493.t2.cds1 | Carbamoyl-phosphate synthase small chain [Hondaea fermentalgiana] | Amino acid metablism |
| scaffold1091_238 | A | A | G | A<->G | 35 | GAG<->GGG | E<->G | nonsyn | scaffold1091.t1.cds1 | N-alpha-acetyltransferase 15, NatA auxiliary subunit [Hondaea fermentalgiana] | Chromosome and associated proteins |
| scaffold1371_2379 | A | C | A | A<->C | 370 | AAC<->CAC | N<->H | nonsyn | scaffold1371.t1.cds2 | Phosphatidylinositol 4-kinase gamma 2 [Hondaea fermentalgiana] | Regulation of actin cytoskeleton |
| scaffold2099_1241 | A | A | T | A<->T | 670 | GTC<->GAC | V<->D | nonsyn | scaffold2099.t1.cds1 | Phytochrome [Hondaea fermentalgiana] | Circadian rhythm |
| scaffold108_35329 | A | A | G | A<->G | 1051 | AAT<->AGT | N<->S | nonsyn | scaffold108.t16.cds1 | Fanconi anemia group D2 protein-like [Hondaea fermentalgiana] | Fanconi anemia pathway |
| scaffold145_18276 | G | A | G | G<->A | 57 | CGA<->CAA | R<->Q | nonsyn | scaffold145.t7.cds1 | Cell division control protein 45-like [Hondaea fermentalgiana] | DNA replication |
| scaffold195_22811 | C | T | C | C<->T | 604 | GCC<->GTC | A<->V | nonsyn | scaffold195.t7.cds1 | beta-carotene synthase | Carotenoid biosynthesis |
| scaffold680_2148 | C | T | C | C<->T | 111 | GGT<->GAT | G<->D | nonsyn | scaffold680.t2.cds1 | MORN repeat-containing protein 4 [Hondaea fermentalgiana] | Cilium and associated proteins |
| scaffold756_1486 | A | T | A | A<->T | 401 | GAG<->GTG | E<->V | nonsyn | scaffold756.t1.cds1 | TBC1 domain family member 1 [Hondaea fermentalgiana] | Thyroid hormone signaling pathway |
| scaffold320_6626 | C | C | G | C<->G | 284 | CGC<->GGC | R<->G | nonsyn | scaffold320.t2.cds1 | Nucleosome assembly protein 1;1 [Hondaea fermentalgiana] | Chromosome and associated proteins |
| scaffold1536_6901 | C | T | C | C<->T | 1136 | ACC<->ACT | T<->T | syn | scaffold1536.t1.cds1 | polyunsaturated fatty acid synthase subunit A [Schizochytrium sp. ATCC 20888] | Lipid metabolism |
| scaffold1536_7408 | T | C | T | T<->C | 1 | GCT<->GCC | A<->A | start_mutation | scaffold1536.t1.cds2 | polyunsaturated fatty acid synthase subunit A [Schizochytrium sp. ATCC 20888] | Lipid metabolism |
| scaffold1536_7372 | T | C | T | T<->C | 1293 | ACT<->ACC | T<->T | syn | scaffold1536.t1.cds1 | polyunsaturated fatty acid synthase subunit A [Schizochytrium sp. ATCC 20888] | Lipid metabolism |
| scaffold341_15341 | G | C | G | G<->C | 216 | CGC<->CGG | R<->R | syn | scaffold341.t6.cds1 | Gut esterase 1 [Hondaea fermentalgiana] | Lipid metabolism |
| scaffold2307_683 | C | T | C | C<->T | 142 | GCG<->GCA | A<->A | syn | scaffold2307.t1.cds1 | Kinesin light chain 3 [Hondaea fermentalgiana] | Cytoskeleton proteins |
| scaffold2307_944 | C | T | C | C<->T | 55 | CAG<->CAA | Q<->Q | syn | scaffold2307.t1.cds1 | Kinesin light chain 3 [Hondaea fermentalgiana] | Cytoskeleton proteins |
| scaffold2394_659 | G | T | G | G<->T | 114 | GTG<->GTT | V<->V | syn | scaffold2394.t1.cds1 | Kinesin light chain 3 [Hondaea fermentalgiana] | Cytoskeleton proteins |
| scaffold2402_492 | C | T | C | C<->T | 91 | CTC<->CTT | L<->L | syn | scaffold2402.t1.cds1 | Kinesin light chain 3 [Hondaea fermentalgiana] | Cytoskeleton proteins |
| scaffold2450_646 | T | T | C | T<->C | 150 | CGT<->CGC | R<->R | syn | scaffold2450.t1.cds1 | Kinesin light chain 3 [Hondaea fermentalgiana] | Cytoskeleton proteins |
| scaffold54_37534 | C | T | C | C<->T | 69 | CTG<->TTG | L<->L | syn | scaffold54.t7.cds2 | Dynein heavy chain 6, axonemal [Hondaea fermentalgiana] | Cytoskeleton proteins |
| scaffold697_9330 | C | T | C | C<->T | 56 | TCG<->TCA | S<->S | syn | scaffold697.t4.cds1 | Kinesin light chain 3 [Hondaea fermentalgiana] | Cytoskeleton proteins |
| scaffold2450_769 | G | G | A | G<->A | 191 | CAG<->CAA | Q<->Q | syn | scaffold2450.t1.cds1 | Kinesin light chain 3 [Hondaea fermentalgiana] | Cytoskeleton proteins |
| scaffold2450_787 | T | G | T | T<->G | 197 | GGT<->GGG | G<->G | syn | scaffold2450.t1.cds1 | Kinesin light chain 3 [Hondaea fermentalgiana] | Cytoskeleton proteins |
| scaffold2450_826 | T | G | T | T<->G | 210 | CCT<->CCG | P<->P | syn | scaffold2450.t1.cds1 | Kinesin light chain 3 [Hondaea fermentalgiana] | Cytoskeleton proteins |
| scaffold385_14635 | A | G | A | A<->G | 221 | GAT<->GAC | D<->D | syn | scaffold385.t5.cds1 | Kinesin, putative [Hondaea fermentalgiana] | Cytoskeleton proteins |
| scaffold2402_705 | T | C | G | T<->CG | 162 | ACT<->ACC\|ACG | T<->T\|T | syn\|syn | scaffold2402.t1.cds1 | Kinesin light chain 3 [Hondaea fermentalgiana] | Cytoskeleton proteins |
| scaffold2402_780 | T | C | T | T<->C | 187 | GAT<->GAC | D<->D | syn | scaffold2402.t1.cds1 | Kinesin light chain 3 [Hondaea fermentalgiana] | Cytoskeleton proteins |
| scaffold694_17903 | A | C | A | A<->C | 293 | GCA<->GCC | A<->A | syn | scaffold694.t4.cds1 | Kinesin light chain 3 [Hondaea fermentalgiana] | Cytoskeleton proteins |
| scaffold1778_2104 | A | A | G | A<->G | 88 | GTT<->GTC | V<->V | syn | scaffold1778.t1.cds1 | Kinesin light chain 3 [Hondaea fermentalgiana] | Cytoskeleton proteins |
| scaffold2319_978 | G | A | G | G<->A | 94 | GAC<->GAT | D<->D | syn | scaffold2319.t1.cds1 | Kinesin light chain 3 [Hondaea fermentalgiana] | Cytoskeleton proteins |
| scaffold2391_407 | G | A | T | G<->AT | 16 | ACG<->ACA\|ACT | T<->T\|T | syn\|syn | scaffold2391.t1.cds1 | Kinesin light chain 3 [Hondaea fermentalgiana] | Cytoskeleton proteins |
| scaffold2450_805 | A | A | G | A<->G | 203 | GAA<->GAG | E<->E | syn | scaffold2450.t1.cds1 | Kinesin light chain 3 [Hondaea fermentalgiana] | Cytoskeleton proteins |
| scaffold694_17144 | G | A | G | G<->A | 40 | CCG<->CCA | P<->P | syn | scaffold694.t4.cds1 | Kinesin light chain 3 [Hondaea fermentalgiana] | Cytoskeleton proteins |
| scaffold1765_5115 | T | C | T | T<->C | 184 | CAT<->CAC | H<->H | syn | scaffold1765.t2.cds1 | Kinesin light chain 3 [Hondaea fermentalgiana] | Cytoskeleton proteins |
| scaffold1765_5091 | C | T | C | C<->T | 176 | GAC<->GAT | D<->D | syn | scaffold1765.t2.cds1 | Kinesin light chain 3 [Hondaea fermentalgiana] | Cytoskeleton proteins |
| scaffold224_3720 | T | T | C | T<->C | 915 | CAT<->CAC | H<->H | syn | scaffold224.t1.cds1 | Alpha-glucosidase [Hondaea fermentalgiana] | Glycan biosynthesis and metabolism |
| scaffold384_13112 | T | T | C | T<->C | 1169 | ATT<->ATC | I<->I | syn | scaffold384.t1.cds2 | GREB1-like protein [Hondaea fermentalgiana] | Glycan biosynthesis and metabolism |
| scaffold384_13130 | T | T | C | T<->C | 1175 | GTT<->GTC | V<->V | syn | scaffold384.t1.cds2 | GREB1-like protein [Hondaea fermentalgiana] | Glycan biosynthesis and metabolism |
| scaffold384_13214 | T | T | C | T<->C | 1203 | CAT<->CAC | H<->H | syn | scaffold384.t1.cds2 | GREB1-like protein [Hondaea fermentalgiana] | Glycan biosynthesis and metabolism |
| scaffold384_13235 | T | T | C | T<->C | 1210 | CGT<->CGC | R<->R | syn | scaffold384.t1.cds2 | GREB1-like protein [Hondaea fermentalgiana] | Glycan biosynthesis and metabolism |
| scaffold396_1881 | T | T | C | T<->C | 600 | ATT<->ATC | I<->I | syn | scaffold396.t1.cds1 | Beta-hexosaminidase subunit beta [Hondaea fermentalgiana] | Glycan biosynthesis and metabolism |
| scaffold396_1929 | T | T | C | T<->C | 616 | CAT<->CAC | H<->H | syn | scaffold396.t1.cds1 | Beta-hexosaminidase subunit beta [Hondaea fermentalgiana] | Glycan biosynthesis and metabolism |
| scaffold384_14828 | A | G | A | A<->G | 62 | TCA<->TCG | S<->S | syn | scaffold384.t1.cds3 | GREB1-like protein [Hondaea fermentalgiana] | Glycan biosynthesis and metabolism |
| scaffold396_1920 | G | G | A | G<->A | 613 | ACG<->ACA | T<->T | syn | scaffold396.t1.cds1 | Beta-hexosaminidase subunit beta [Hondaea fermentalgiana] | Glycan biosynthesis and metabolism |
| scaffold396_1909 | C | C | T | C<->T | 610 | CTG<->TTG | L<->L | syn | scaffold396.t1.cds1 | Beta-hexosaminidase subunit beta [Hondaea fermentalgiana] | Glycan biosynthesis and metabolism |
| scaffold396_2481 | T | C | T | T<->C | 800 | AAT<->AAC | N<->N | syn | scaffold396.t1.cds1 | Beta-hexosaminidase subunit beta [Hondaea fermentalgiana] | Glycan biosynthesis and metabolism |
| scaffold396_2976 | C | C | T | C<->T | 965 | GCC<->GCT | A<->A | syn | scaffold396.t1.cds1 | Beta-hexosaminidase subunit beta [Hondaea fermentalgiana] | Glycan biosynthesis and metabolism |
| scaffold396_553 | C | C | T | C<->T | 158 | CTG<->TTG | L<->L | syn | scaffold396.t1.cds1 | Beta-hexosaminidase subunit beta [Hondaea fermentalgiana] | Glycan biosynthesis and metabolism |
| scaffold384_15506 | G | A | G | G<->A | 288 | ACG<->ACA | T<->T | syn | scaffold384.t1.cds3 | GREB1-like protein [Hondaea fermentalgiana] | Glycan biosynthesis and metabolism |
| scaffold396_2676 | G | A | G | G<->A | 865 | GTG<->GTA | V<->V | syn | scaffold396.t1.cds1 | Beta-hexosaminidase subunit beta [Hondaea fermentalgiana] | Glycan biosynthesis and metabolism |
| scaffold396_2688 | G | A | G | G<->A | 869 | GCG<->GCA | A<->A | syn | scaffold396.t1.cds1 | Beta-hexosaminidase subunit beta [Hondaea fermentalgiana] | Glycan biosynthesis and metabolism |
| scaffold560_5038 | T | T | C | T<->C | 11 | GCT<->GCC | A<->A | syn | scaffold560.t3.cds1 | Lysine-specific demethylase 8 [Hondaea fermentalgiana] | Transcription |
| scaffold1932_1502 | G | G | A | G<->A | 89 | TCG<->TCA | S<->S | syn | scaffold1932.t1.cds2 | Myelin regulatory factor [Hondaea fermentalgiana] | Transcription |
| scaffold1932_312 | T | G | T | T<->G | 104 | GTT<->GTG | V<->V | syn | scaffold1932.t1.cds1 | Myelin regulatory factor [Hondaea fermentalgiana] | Transcription |
| scaffold1932_4261 | A | G | A | A<->G | 251 | TCA<->TCG | S<->S | syn | scaffold1932.t1.cds4 | Myelin regulatory factor [Hondaea fermentalgiana] | Transcription |
| scaffold1932_2488 | T | C | T | T<->C | 172 | GGT<->GGC | G<->G | syn | scaffold1932.t1.cds3 | Myelin regulatory factor [Hondaea fermentalgiana] | Transcription |
| scaffold1932_3706 | C | C | T | C<->T | 66 | CTC<->CTT | L<->L | syn | scaffold1932.t1.cds4 | Myelin regulatory factor [Hondaea fermentalgiana] | Transcription |
| scaffold1932_4114 | C | C | T | C<->T | 202 | GTC<->GTT | V<->V | syn | scaffold1932.t1.cds4 | Myelin regulatory factor [Hondaea fermentalgiana] | Transcription |
| scaffold1932_4153 | T | C | T | T<->C | 215 | GTT<->GTC | V<->V | syn | scaffold1932.t1.cds4 | Myelin regulatory factor [Hondaea fermentalgiana] | Transcription |
| scaffold1932_63 | G | C | G | G<->C | 21 | GGG<->GGC | G<->G | syn | scaffold1932.t1.cds1 | Myelin regulatory factor [Hondaea fermentalgiana] | Transcription |
| scaffold1932_2002 | A | A | T | A<->T | 10 | GCA<->GCT | A<->A | syn | scaffold1932.t1.cds3 | Myelin regulatory factor [Hondaea fermentalgiana] | Transcription |
| scaffold1932_2431 | A | A | G | A<->G | 153 | ACA<->ACG | T<->T | syn | scaffold1932.t1.cds3 | Myelin regulatory factor [Hondaea fermentalgiana] | Transcription |
| scaffold1932_336 | G | A | G | G<->A | 112 | ACG<->ACA | T<->T | syn | scaffold1932.t1.cds1 | Myelin regulatory factor [Hondaea fermentalgiana] | Transcription |
| scaffold1932_105 | T | T | G | T<->G | 35 | GCT<->GCG | A<->A | syn | scaffold1932.t1.cds1 | Myelin regulatory factor [Hondaea fermentalgiana] | Transcription |
| scaffold1932_3940 | T | T | G | T<->G | 144 | GCT<->GCG | A<->A | syn | scaffold1932.t1.cds4 | Myelin regulatory factor [Hondaea fermentalgiana] | Transcription |
| scaffold564_19755 | C | T | C | C<->T | 296 | ACG<->ACA | T<->T | syn | scaffold564.t7.cds1 | HemK methyltransferase family member 1 [Hondaea fermentalgiana] | Translation |
| scaffold935_6420 | C | T | C | C<->T | 8 | ACG<->ACA | T<->T | syn | scaffold935.t2.cds1 | Ribosome-recycling factor [Hondaea fermentalgiana] | Translation |
| scaffold428_21383 | G | A | G | G<->A | 40 | GTG<->GTA | V<->V | syn | scaffold428.t10.cds1 | Elongation factor 2 [Hondaea fermentalgiana] | Translation |
| scaffold70_37823 | C | T | C | C<->T | 378 | CTC<->CTT | L<->L | syn | scaffold70.t13.cds1 | Leucine-rich repeat-containing protein 1 [Hondaea fermentalgiana] | Ubiquitin system |
| scaffold2053_831 | A | G | A | A<->G | 4 | CTT<->CTC | L<->L | syn | scaffold2053.t1.cds4 | Leucine-rich repeat-containing protein 1 [Hondaea fermentalgiana] | Ubiquitin system |
| scaffold299_19800 | T | G | T | T<->G | 698 | GCA<->GCC | A<->A | syn | scaffold299.t5.cds1 | Ankyrin repeat domain-containing protein 1 [Hondaea fermentalgiana] | Ubiquitin system |
| scaffold197_9970 | C | T | C | C<->T | 99 | GCC<->GCT | A<->A | syn | scaffold197.t4.cds2 | Ubiquitin carboxyl-terminal hydrolase 2 [Hondaea fermentalgiana] | Ubiquitin system |
| scaffold725_5823 | T | T | A | T<->A | 52 | GGT<->GGA | G<->G | syn | scaffold725.t3.cds1 | WASH complex subunit 4 [Hondaea fermentalgiana] | Transport and catabolism |
| scaffold145_38010 | A | G | A | A<->G | 89 | GAT<->GAC | D<->D | syn | scaffold145.t17.cds3 | Tubulin beta chain [Hondaea fermentalgiana] | Transport and catabolism |
| scaffold145_38016 | G | A | G | G<->A | 87 | GAC<->GAT | D<->D | syn | scaffold145.t17.cds3 | Tubulin beta chain [Hondaea fermentalgiana] | Transport and catabolism |
| scaffold345_21362 | C | G | C | C<->G | 27 | GGC<->GGG | G<->G | syn | scaffold345.t5.cds1 | Mitochondrial import inner membrane translocase subunit Tim23 [Hondaea fermentalgiana] | Mitochondrial biogenesis |
| scaffold472_8035 | G | G | C | G<->C | 655 | CCG<->CCC | P<->P | syn | scaffold472.t2.cds1 | Cyclin-dependent kinase C-1 [Hondaea fermentalgiana] | ATP-dependent chromatin remodeling |
| scaffold674_2913 | A | G | A | A<->G | 839 | TTA<->TTG | L<->L | syn | scaffold674.t1.cds2 | Helicase swr1 [Hondaea fermentalgiana] | ATP-dependent chromatin remodeling |
| scaffold106_26666 | G | C | G | G<->C | 301 | CCG<->CCC | P<->P | syn | scaffold106.t10.cds1 | Delta-1-pyrroline-5-carboxylate synthase [Hondaea fermentalgiana] | Amino acid metabolism |
| scaffold163_9593 | C | C | G | C<->G | 682 | GTG<->GTC | V<->V | syn | scaffold163.t4.cds1 | Trafficking protein particle complex subunit 11 [Hondaea fermentalgiana] | Membrane trafficking |
| scaffold2282_1177 | T | C | T | T<->C | 126 | GGT<->GGC | G<->G | syn | scaffold2282.t1.cds1 | Leucine-rich repeat serine/threonine-protein kinase 2 [Hondaea fermentalgiana] | Membrane trafficking |
| scaffold463_15377 | C | C | G | C<->G | 645 | GGC<->GGG | G<->G | syn | scaffold463.t5.cds1 | Brefeldin A-inhibited guanine nucleotide-exchange protein 1 [Hondaea fermentalgiana] | Membrane trafficking |
| scaffold760_6913 | T | C | T | T<->C | 909 | GAT<->GAC | D<->D | syn | scaffold760.t2.cds1 | Vacuolar protein sorting-associated protein 53-like [Hondaea fermentalgiana] | Membrane trafficking |
| scaffold2411_922 | A | A | G | A<->G | 82 | CTT<->CTC | L<->L | syn | scaffold2411.t1.cds1 | Leucine-rich repeat serine/threonine-protein kinase 1 [Hondaea fermentalgiana] | Membrane trafficking |
| scaffold903_7856 | G | C | G | G<->C | 21 | GGC<->GGG | G<->G | syn | scaffold903.t3.cds1 | Kinetochore protein Spc25 [Hondaea fermentalgiana] | Chromosome and associated proteins |
| scaffold877_8854 | T | C | T | T<->C | 160 | GGA<->GGG | G<->G | syn | scaffold877.t4.cds1 | Chromatin modification-related protein eaf3 [Hondaea fermentalgiana] | Chromosome and associated proteins |
| scaffold119_13491 | G | A | G | G<->A | 409 | ATC<->ATT | I<->I | syn | scaffold119.t6.cds1 | RCC1 and BTB domain-containing protein 2 [Hondaea fermentalgiana] | Chromosome and associated proteins |
| scaffold1694_4300 | T | C | T | T<->C | 609 | GAT<->GAC | D<->D | syn | scaffold1694.t2.cds1 | DNA replication licensing factor MCM5 [Hondaea fermentalgiana] | Replication and repair |
| scaffold2399_181 | A | A | G | A<->G | 351 | TTG<->CTG | L<->L | syn | scaffold2399.t1.cds1 | Calpain-like protein [Hondaea fermentalgiana] | Protein folding, sorting and degradation |
| scaffold143_25440 | T | T | A | T<->A | 167 | GGT<->GGA | G<->G | syn | scaffold143.t6.cds2 | Methionine aminopeptidase 2 [Hondaea fermentalgiana] | Protein folding, sorting and degradation |
| scaffold175_31383 | C | T | C | C<->T | 323 | AAG<->AAA | K<->K | syn | scaffold175.t11.cds1 | Lon protease-like, mitochondrial [Hondaea fermentalgiana] | Protein folding, sorting and degradation |
| scaffold1751_1636 | C | T | C | C<->T | 53 | CTC<->CTT | L<->L | syn | scaffold1751.t1.cds1 | Sterile alpha and TIR motif-containing protein tir-1 [Hondaea fermentalgiana] | Nicotinate and nicotinamide metabolism |
| scaffold476_280 | T | T | C | T<->C | 86 | TGT<->TGC | C<->C | syn | scaffold476.t1.cds1 | Methionine synthase [Hondaea fermentalgiana] | Cobalamin transport and metabolism |
| scaffold48_6685 | C | T | C | C<->T | 22 | ATC<->ATT | I<->I | syn | scaffold48.t2.cds1 | Cilia- and flagella-associated protein 221 [Hondaea fermentalgiana] | Signal transduction |
| scaffold61_6422 | C | T | C | C<->T | 397 | GAG<->GAA | E<->E | syn | scaffold61.t3.cds1 | Adenylyltransferase and sulfurtransferase MOCS3 [Hondaea fermentalgiana] | Folding, sorting and degradation |
| scaffold67_31924 | C | T | C | C<->T | 34 | GGG<->GGA | G<->G | syn | scaffold67.t14.cds1 | 5-hydroxyisourate hydrolase [Hondaea fermentalgiana] | Nucleotide metabolism |
| scaffold31_47832 | A | A | G | A<->G | 9 | GAT<->GAC | D<->D | syn | scaffold31.t15.cds1 | Zinc metalloproteinase/disintegrin [Hondaea fermentalgiana] | Peptidases and inhibitors |
| scaffold643_8237 | G | A | G | G<->A | 161 | GAG<->GAA | E<->E | syn | scaffold643.t1.cds1 | Cytochrome c peroxidase, mitochondrial [Hondaea fermentalgiana] | Oxidoreductases |
| scaffold942_10312 | G | A | G | G<->A | 1105 | GCC<->GCT | A<->A | syn | scaffold942.t3.cds1 | Myosin-1 [Hondaea fermentalgiana] | Motor proteins |
| scaffold2312_449 | G | A | G | G<->A | 133 | ACG<->ACA | T<->T | syn | scaffold2312.t1.cds1 | Hypothetical Protein FCC1311_113272 [Hondaea fermentalgiana] | Hypothetical protein |
| scaffold1_7670 | G | G | T | G<->T | 84 | GTG<->GTT | V<->V | syn | scaffold1.t2.cds2 | hypothetical protein [Flavobacteriaceae bacterium] | Hypothetical protein |
| scaffold429_18607 | C | T | C | C<->T | 415 | CCC<->CCT | P<->P | syn | scaffold429.t6.cds1 | Hypothetical Protein FCC1311_029752 [Hondaea fermentalgiana] | Hypothetical protein |
| scaffold470_556 | T | T | C | T<->C | 167 | ATT<->ATC | I<->I | syn | scaffold470.t1.cds1 | Hypothetical Protein FCC1311_113272 [Hondaea fermentalgiana] | Hypothetical protein |
| scaffold547_13494 | T | T | C | T<->C | 205 | GAT<->GAC | D<->D | syn | scaffold547.t6.cds1 | Hypothetical Protein FCC1311_024042 [Hondaea fermentalgiana] | Hypothetical protein |
| scaffold58_54919 | C | T | C | C<->T | 179 | CGG<->CGA | R<->R | syn | scaffold58.t19.cds2 | Hypothetical Protein FCC1311_062132 [Hondaea fermentalgiana] | Hypothetical protein |
| scaffold94_46209 | T | T | C | T<->C | 1123 | GAT<->GAC | D<->D | syn | scaffold94.t7.cds1 | Mic1 domain-containing protein DDB_G0286707 [Hondaea fermentalgiana] | Hypothetical protein |
| scaffold973_805 | T | T | C | T<->C | 5 | GAT<->GAC | D<->D | syn | scaffold973.t1.cds1 | Hypothetical Protein FCC1311_002102 [Hondaea fermentalgiana] | Hypothetical protein |
| scaffold984_6338 | T | T | C | T<->C | 585 | AGA<->AGG | R<->R | syn | scaffold984.t3.cds2 | Hypothetical Protein FCC1311_105742 [Hondaea fermentalgiana] | Hypothetical protein |
| scaffold1073_10091 | A | G | A | A<->G | 354 | CGA<->CGG | R<->R | syn | scaffold1073.t2.cds2 | WD repeat-containing protein 1 [Hondaea fermentalgiana] | Hypothetical protein |
| scaffold150_843 | A | G | A | A<->G | 63 | GAA<->GAG | E<->E | syn | scaffold150.t1.cds1 | Hypothetical Protein FCC1311_104332 [Hondaea fermentalgiana] | Hypothetical protein |
| scaffold1922_2451 | A | G | A | A<->G | 35 | TTA<->CTA | L<->L | syn | scaffold1922.t2.cds2 | helix-turn-helix domain-containing protein [Hyphomicrobiales bacterium] | Hypothetical protein |
| scaffold2125_201 | G | G | A | G<->A | 321 | TCC<->TCT | S<->S | syn | scaffold2125.t1.cds1 | Hypothetical Protein FCC1311_113272 [Hondaea fermentalgiana] | Hypothetical protein |
| scaffold2134_2252 | G | G | A | G<->A | 199 | GGC<->GGT | G<->G | syn | scaffold2134.t1.cds1 | Hypothetical Protein FCC1311_113102 [Hondaea fermentalgiana] | Hypothetical protein |
| scaffold2134_2315 | G | G | A | G<->A | 178 | GCC<->GCT | A<->A | syn | scaffold2134.t1.cds1 | Hypothetical Protein FCC1311_113102 [Hondaea fermentalgiana] | Hypothetical protein |
| scaffold2134_2324 | A | G | A | A<->G | 175 | ACT<->ACC | T<->T | syn | scaffold2134.t1.cds1 | Hypothetical Protein FCC1311_113102 [Hondaea fermentalgiana] | Hypothetical protein |
| scaffold2134_2657 | A | G | A | A<->G | 64 | ACT<->ACC | T<->T | syn | scaffold2134.t1.cds1 | Hypothetical Protein FCC1311_113102 [Hondaea fermentalgiana] | Hypothetical protein |
| scaffold254_9134 | A | G | A | A<->G | 217 | TAT<->TAC | Y<->Y | syn | scaffold254.t4.cds1 | Hypothetical Protein FCC1311_078722 [Hondaea fermentalgiana] | Hypothetical protein |
| scaffold341_15377 | A | G | A | A<->G | 204 | GGT<->GGC | G<->G | syn | scaffold341.t6.cds1 | Hypothetical Protein FCC1311_066552 [Hondaea fermentalgiana] | Hypothetical protein |
| scaffold470_544 | A | G | A | A<->G | 163 | AAA<->AAG | K<->K | syn | scaffold470.t1.cds1 | Hypothetical Protein FCC1311_113272 [Hondaea fermentalgiana] | Hypothetical protein |
| scaffold58_56555 | A | G | A | A<->G | 89 | CCT<->CCC | P<->P | syn | scaffold58.t19.cds1 | Hypothetical Protein FCC1311_062132 [Hondaea fermentalgiana] | Hypothetical protein |
| scaffold58_56561 | A | G | A | A<->G | 87 | AGT<->AGC | S<->S | syn | scaffold58.t19.cds1 | Hypothetical Protein FCC1311_062132 [Hondaea fermentalgiana] | Hypothetical protein |
| scaffold64_51942 | A | G | A | A<->G | 12 | CGA<->CGG | R<->R | syn | scaffold64.t14.cds1 | Hypothetical Protein FCC1311_092382 [Hondaea fermentalgiana] | Hypothetical protein |
| scaffold806_1180 | A | G | A | A<->G | 170 | CTT<->CTC | L<->L | syn | scaffold806.t1.cds1 | Hypothetical Protein FCC1311_110092 [Hondaea fermentalgiana] | Hypothetical protein |
| scaffold1620_4506 | T | C | T | T<->C | 63 | GCA<->GCG | A<->A | syn | scaffold1620.t2.cds1 | Hypothetical Protein FCC1311_113162 [Hondaea fermentalgiana] | Hypothetical protein |
| scaffold17_280 | C | C | A | C<->A | 69 | GCC<->GCA | A<->A | syn | scaffold17.t1.cds1 | hypothetical protein | Hypothetical protein |
| scaffold2049_176 | T | C | T | T<->C | 8 | TGT<->TGC | C<->C | syn | scaffold2049.t1.cds1 | Hypothetical Protein FCC1311_104322 [Hondaea fermentalgiana] | Hypothetical protein |
| scaffold2312_380 | T | C | T | T<->C | 110 | GAT<->GAC | D<->D | syn | scaffold2312.t1.cds1 | Hypothetical Protein FCC1311_111472 [Hondaea fermentalgiana] | Hypothetical protein |
| scaffold319_1945 | C | C | T | C<->T | 505 | GTC<->GTT | V<->V | syn | scaffold319.t1.cds1 | Hypothetical Protein FCC1311_034842 [Hondaea fermentalgiana] | Hypothetical protein |
| scaffold454_18459 | T | C | T | T<->C | 319 | GAT<->GAC | D<->D | syn | scaffold454.t6.cds1 | Hypothetical Protein FCC1311_076022 [Hondaea fermentalgiana] | Hypothetical protein |
| scaffold470_820 | C | C | T | C<->T | 255 | GAC<->GAT | D<->D | syn | scaffold470.t1.cds1 | Hypothetical Protein FCC1311_076022 [Hondaea fermentalgiana] | Hypothetical protein |
| scaffold516_14433 | C | C | T | C<->T | 1270 | GAG<->GAA | E<->E | syn | scaffold516.t1.cds1 | WD repeat-containing protein wdr-5.1 [Hondaea fermentalgiana] | Hypothetical protein |
| scaffold1071_325 | C | T | C | C<->T | 99 | CGC<->CGT | R<->R | syn | scaffold1071.t1.cds1 | Disease resistance protein RPP5 [Hondaea fermentalgiana] | Hypothetical protein |
| scaffold1480_7507 | C | T | C | C<->T | 24 | CCG<->CCA | P<->P | syn | scaffold1480.t3.cds1 | Ankyrin repeat and protein kinase domain-containing protein 1 [Hondaea fermentalgiana] | Hypothetical protein |
| scaffold636_8287 | T | C | T | T<->C | 337 | GAT<->GAC | D<->D | syn | scaffold636.t4.cds1 | Hypothetical Protein FCC1311_047472 [Hondaea fermentalgiana] | Hypothetical protein |
| scaffold14_67785 | G | A | G | G<->A | 224 | TTG<->TTA | L<->L | syn | scaffold14.t20.cds2 | Hypothetical Protein FCC1311_055092 [Hondaea fermentalgiana] | Hypothetical protein |
| scaffold164_19954 | G | A | G | G<->A | 2646 | CAC<->CAT | H<->H | syn | scaffold164.t6.cds2 | Hypothetical Protein FCC1311_012962 [Hondaea fermentalgiana] | Hypothetical protein |
| scaffold2125_273 | A | A | T | A<->T | 297 | CCT<->CCA | P<->P | syn | scaffold2125.t1.cds1 | Hypothetical Protein FCC1311_113272 [Hondaea fermentalgiana] | Hypothetical protein |
| scaffold2134_2093 | A | A | G | A<->G | 252 | GCT<->GCC | A<->A | syn | scaffold2134.t1.cds1 | Hypothetical Protein FCC1311_113102 [Hondaea fermentalgiana] | Hypothetical protein |
| scaffold516_14448 | A | A | G | A<->G | 1265 | AAT<->AAC | N<->N | syn | scaffold516.t1.cds1 | WD repeat-containing protein wdr-5.1 [Hondaea fermentalgiana] | Hypothetical protein |
| scaffold58_56483 | A | A | G | A<->G | 113 | ACT<->ACC | T<->T | syn | scaffold58.t19.cds1 | Hypothetical Protein FCC1311_062132 [Hondaea fermentalgiana] | Hypothetical protein |
| scaffold74_747 | G | A | G | G<->A | 248 | GCG<->GCA | A<->A | syn | scaffold74.t1.cds1 | Hypothetical Protein FCC1311_005815 [Hondaea fermentalgiana] | Hypothetical protein |
| scaffold794_6418 | G | A | G | G<->A | 407 | GAG<->GAA | E<->E | syn | scaffold794.t3.cds1 | Hypothetical Protein FCC1311_066842 [Hondaea fermentalgiana] | Hypothetical protein |
| scaffold341_15560 | G | A | G | G<->A | 143 | ATC<->ATT | I<->I | syn | scaffold341.t6.cds1 | Hypothetical Protein FCC1311_066552 [Hondaea fermentalgiana] | Hypothetical protein |
| scaffold1425_6040 | C | T | C | C<->T | 156 | TCG<->TCA | S<->S | syn | scaffold1425.t3.cds1 | Hypothetical Protein FCC1311_051992 [Hondaea fermentalgiana] | Hypothetical protein |
| scaffold150_786 | C | T | C | C<->T | 44 | CAC<->CAT | H<->H | syn | scaffold150.t1.cds1 | Hypothetical Protein FCC1311_104332 [Hondaea fermentalgiana] | Hypothetical protein |
| scaffold17_214 | T | T | C | T<->C | 47 | ACT<->ACC | T<->T | syn | scaffold17.t1.cds1 | hypothetical protein | Hypothetical protein |
| scaffold1905_636 | T | T | C | T<->C | 110 | GAT<->GAC | D<->D | syn | scaffold1905.t1.cds1 | Hypothetical Protein FCC1311_103402 [Hondaea fermentalgiana] | Hypothetical protein |
| scaffold1_7688 | T | T | C | T<->C | 90 | ATT<->ATC | I<->I | syn | scaffold1.t2.cds2 | hypothetical protein [Flavobacteriaceae bacterium] | Hypothetical protein |
| scaffold1_7703 | T | T | G | T<->G | 95 | GCT<->GCG | A<->A | syn | scaffold1.t2.cds2 | hypothetical protein [Flavobacteriaceae bacterium] | Hypothetical protein |
| scaffold1_7715 | T | T | C | T<->C | 99 | GAT<->GAC | D<->D | syn | scaffold1.t2.cds2 | hypothetical protein [Flavobacteriaceae bacterium] | Hypothetical protein |

**Supplementary Table S6 Hydrogen bonds and salt bridges formed before and after mutation at G3PP position 147**

Table S6-1: Hydrogen bonds formed before and after mutation at G3PP position 147

| Strains | Index | Residue | AA | Distance H-A | Distance D-A | Donor Angle | Protein donor ? | Side chain | Donor Atom | Acceptor Atom |
| --- | --- | --- | --- | --- | --- | --- | --- | --- | --- | --- |
| ZW2 | 1 | 99 | Gly | 1.95 | 2.96 | 174.39 | √ | × | 942[Nam] | 7[O3] |
|  | 2 | 99 | Gly | 2.11 | 2.78 | 124.36 | × | × | 7[O3] | 945[O2] |
|  | 3 | 116 | Glu | 2.10 | 2.61 | 110.96 | × | √ | 6[O3] | 1094[O-] |
|  | 4 | 117 | Val | 2.07 | 3.00 | 152.21 | √ | × | 1096[Nam] | 5[O2] |
|  | 5 | 147 | Asp | 2.06 | 2.97 | 154.87 | × | √ | 9[O3] | 1362[Oco2] |
|  | 6 | 147 | Asp | 2.07 | 2.87 | 137.68 | × | √ | 8[O3] | 1362[Oco2] |
|  | 7 | 149 | Asn | 1.96 | 2.91 | 157.48 | √ | √ | 1389[Nam] | 9[O3] |
|  | 8 | 149 | Asn | 3.24 | 3.91 | 125.41 | √ | × | 1383[Nam] | 9[O3] |
|  | 9 | 154 | Lys | 1.89 | 2.52 | 117.26 | √ | √ | 1451[N3+] | 8[O3] |
| ZW1 | 1 | 97 | Ala | 1.77 | 2.72 | 162.50 | × | × | 7[O3] | 930[O2] |
|  | 2 | 99 | Gly | 2.26 | 3.16 | 147.84 | √ | × | 942[Nam] | 5[O2] |
|  | 3 | 99 | Gly | 1.92 | 2.73 | 139.82 | × | × | 6[O3] | 945[O2] |
|  | 4 | 99 | Gly | 2.09 | 2.58 | 109.15 | × | × | 8[O3] | 945[O2] |
|  | 5 | 99 | Gly | 2.18 | 3.08 | 153.53 | × | × | 9[O3] | 945[O2] |
|  | 6 | 154 | Lys | 2.49 | 2.98 | 109.41 | √ | √ | 14471[N3+] | 9[O3] |

Table S6-2: Salt bridges formed before and after mutation at G3PP position 147

| Strains | Index | Residue | AA | Distance | Protein positive? | Ligand Group | Ligand Atoms |
| --- | --- | --- | --- | --- | --- | --- | --- |
| ZW2 | 1 | 154 | Lys | 3.35 | √ | Phosphate | 10, 10, 4, 5, 6, 7 |
| ZW1 | 1 | 154 | Lys | 3.4 | √ | Phosphate | 10, 10, 4, 5, 6, 7 |

**Supplementary Table S7 DEGs between *Schizochytrium* ZW1 and ZW2 (fold change ≥ 2, fold change ≤ 0.5, q value < 0.05).**

| Gene ID | log2(E6/10151) | Qvalue(10151-vs-E6) | Annotation | Classification |
| --- | --- | --- | --- | --- |
| Unigene941_All | 2.441538426 | 2.60833E-13 | delta3,5-delta2,4-dienoyl-CoA isomerase | lipid metabolism |
| Unigene3124_All | 2.135735445 | 1.33439E-12 | sterol carrier protein 2 | lipid metabolism |
| CL535.Contig3_All | 2.067550356 | 9.12975E-06 | phosphatidate phosphatase | lipid metabolism |
| Unigene4076_All | 1.71022506 | 3.62098E-25 | acetyl-CoA carboxylase | lipid metabolism |
| CL262.Contig10_All | 1.604557181 | 3.3376E-14 | polyunsaturated fatty acid synthase subunit A | lipid metabolism |
| Unigene5127_All | 1.468155152 | 3.67429E-38 | serine palmitoyltransferase | lipid metabolism |
| Unigene7029_All | 1.338967659 | 2.88692E-25 | glycerol kinase | lipid metabolism |
| Unigene5193_All | 1.316886937 | 3.92656E-07 | glycerol-3-phosphate dehydrogenase | lipid metabolism |
| Unigene3753_All | 1.315432423 | 4.74381E-09 | fatty acid synthase subunit alpha, fungi type | lipid metabolism |
| Unigene2374_All | 1.247833398 | 0.000168596 | glycerol kinase | lipid metabolism |
| Unigene3817_All | 1.241582443 | 7.8383E-11 | acyl-CoA dehydrogenase | lipid metabolism |
| CL612.Contig1_All | 1.201312573 | 5.30472E-08 | acetyl-CoA C-acetyltransferase | lipid metabolism |
| CL262.Contig8_All | 1.099633185 | 8.00621E-59 | polyunsaturated fatty acid synthase subunit A | lipid metabolism |
| CL900.Contig2_All | 1.068592759 | 0.005089329 | lysophosphatidate acyltransferase | lipid metabolism |
| CL731.Contig1_All | 1.026907009 | 1.85612E-06 | glycerol-3-phosphate dehydrogenase | lipid metabolism |
| Unigene4276_All | 2.199352137 | 2.07738E-15 | glycine cleavage system H protein | carbohydrate metabolism |
| CL51.Contig2_All | 1.892838045 | 1.88674E-35 | pyruvate dehydrogenase E1 component alpha subunit | carbohydrate metabolism |
| Unigene9552_All | 1.781222828 | 1.78593E-93 | carbonyl reductase 1 | carbohydrate metabolism |
| Unigene2638_All | 1.666315908 | 1.98951E-22 | D-xylose reductase | carbohydrate metabolism |
| Unigene2522_All | 1.629679073 | 9.24918E-29 | phosphoglycerate kinase | carbohydrate metabolism |
| Unigene10732_All | 1.556561003 | 0.005122594 | cytochrome P450 | carbohydrate metabolism |
| Unigene9706_All | 1.497209757 | 3.91271E-30 | alpha-galactosidase | carbohydrate metabolism |
| CL363.Contig1_All | 1.22346826 | 8.60761E-37 | D-xylose reductase | carbohydrate metabolism |
| Unigene380_All | 1.222052032 | 1.08852E-05 | NADPH:quinone reductase | carbohydrate metabolism |
| Unigene1459_All | 1.128936878 | 0.003706035 | glutaredoxin 3 | carbohydrate metabolism |
| Unigene4117_All | 1.126720789 | 0.009119452 | NADH dehydrogenase | carbohydrate metabolism |
| Unigene6389_All | 1.113199784 | 2.82271E-30 | epsilon-lactone hydrolase | carbohydrate metabolism |
| CL363.Contig2_All | 1.103331544 | 3.13723E-43 | D-xylose reductase | carbohydrate metabolism |
| CL363.Contig3_All | 1.091999706 | 2.26745E-14 | L-glyceraldehyde reductase | carbohydrate metabolism |
| Unigene6391_All | 1.089715171 | 1.23593E-53 | ubiquinol oxidase | carbohydrate metabolism |
| Unigene8546_All | 1.073994781 | 2.62911E-05 | 6-methylsalicylic acid synthase | carbohydrate metabolism |
| Unigene10756_All | 1.057611354 | 0.001240229 | catalase | carbohydrate metabolism |
| Unigene10763_All | 1.043468575 | 1.26656E-23 | choline-sulfatase | carbohydrate metabolism |
| Unigene4894_All | 1.036273635 | 0.001522276 | ferric-chelate reductase | carbohydrate metabolism |
| Unigene7011_All | 1.01696624 | 8.69119E-06 | carnitine O-acetyltransferase | carbohydrate metabolism |
| Unigene7393_All | 1.00902558 | 3.23496E-06 | fumarate hydratase | carbohydrate metabolism |
| CL994.Contig1_All | 2.43623269 | 2.60617E-26 | 1-phosphatidylinositol-4-phosphate 5-kinase | signal transduction |
| Unigene2432_All | 2.185910396 | 6.51869E-12 | mitogen-activated protein kinase kinase kinase | signal transduction |
| Unigene10556_All | 2.106552978 | 3.00615E-56 | serum/glucocorticoid-regulated kinase 2 | signal transduction |
| Unigene5350_All | 1.954237736 | 0.000431689 | serine/threonine protein kinase KIN1/ | signal transduction |
| CL627.Contig1_All | 1.937005711 | 2.99489E-07 | TBC1 domain family member 2 | signal transduction |
| CL994.Contig2_All | 1.925531238 | 1.58272E-10 | 1-phosphatidylinositol-4-phosphate 5-kinase | signal transduction |
| CL1051.Contig14_All | 1.423594676 | 1.50494E-14 | serine/threonine-protein kinase CHEK2 | signal transduction |
| CL1051.Contig11_All | 1.156016977 | 2.3282E-19 | ser/thr/tyr protein kinase RAD53 | signal transduction |
| Unigene2059_All | 1.100967257 | 0.001273933 | 5'-AMP-activated protein kinase, regulatory gamma subunit | signal transduction |
| Unigene4519_All | 1.060366141 | 1.51777E-06 | mitogen-activated protein kinase 1/3 | signal transduction |
| Unigene9087_All | 1.050538637 | 2.64875E-10 | ser/thr/tyr protein kinase RAD53 | signal transduction |
| Unigene6257_All | 1.005621515 | 0.00553916 | protein phosphatase PTC1 | signal transduction |
| Unigene9335_All | 4.414586723 | 1.42659E-25 | solute carrier family 35 | Transport and catabolism |
| Unigene6200_All | 1.749044142 | 6.43468E-07 | vacuolar protein-sorting-associated protein 4 | Transport and catabolism |
| Unigene7556_All | 1.345484553 | 0.000855037 | trafficking protein particle complex subunit 3 | Transport and catabolism |
| CL822.Contig2_All | 1.199038821 | 2.24418E-09 | vacuolar protein sorting-associated protein 41 | Transport and catabolism |
| Unigene4109_All | 1.182899953 | 0.009639745 | vacuolar protein sorting-associated protein 1 | Transport and catabolism |
| CL539.Contig2_All | 20.95738521 | 9.47722E-07 | P-type Ca2+ transporter type 2A | Membrane transport |
| CL448.Contig3_All | 5.066639438 | 0.031128843 | high-affinity iron transporter | Membrane transport |
| CL981.Contig1_All | 2.723819642 | 3.13247E-06 | magnesium transporter | Membrane transport |
| Unigene696_All | 2.711464902 | 7.4293E-159 | proton-dependent oligopeptide transporter, POT family | Membrane transport |
| Unigene7174_All | 2.427797065 | 5.28046E-91 | ATP-binding cassette, subfamily B (MDR/TAP), member 1 | Membrane transport |
| CL81.Contig4_All | 2.089224485 | 1.11037E-30 | MFS transporter, SP family, sugar:H+ symporter | Membrane transport |
| CL81.Contig3_All | 2.036581966 | 5.13405E-34 | MFS transporter, SP family, sugar:H+ symporter | Membrane transport |
| Unigene10673_All | 1.653815619 | 0.005690059 | aquaglyceroporin related protein, other eukaryote | Membrane transport |
| Unigene6048_All | 1.439278718 | 3.4501E-11 | proton-dependent oligopeptide transporter, POT family | Membrane transport |
| CL81.Contig7_All | 1.428870644 | 7.68716E-50 | MFS transporter, SP family, sugar:H+ symporter | Membrane transport |
| Unigene7169_All | 1.294152026 | 1.15078E-68 | MFS transporter, SP family, sugar:H+ symporte | Membrane transport |
| Unigene6360_All | 1.252619841 | 1.40771E-23 | ATP-binding cassette, subfamily B (MDR/TAP), member 7 | Membrane transport |
| Unigene4571_All | 1.233420735 | 5.25164E-07 | ammonium transporter, Amt family | Membrane transport |
| Unigene5660_All | 1.065158208 | 2.14285E-42 | aquaglyceroporin related protein | Membrane transport |
| CL81.Contig9_All | 1.043794356 | 1.30987E-51 | MFS transporter, SP family, sugar:H+ symporter | Membrane transport |
| Unigene2352_All | 2.789979227 | 1.63566E-06 | F-box and WD-40 domain protein CDC4 | Cell growth and death |
| Unigene2287_All | 2.488715184 | 1.44291E-62 | F-box and WD-40 domain protein CDC4 | Cell growth and death |
| Unigene2298_All | 2.448306179 | 5.2077E-104 | F-box and WD-40 domain protein CDC4 | Cell growth and death |
| Unigene34_All | 2.233518057 | 0.030116228 | NLR family CARD domain-containing protein 3 | Cell growth and death |
| Unigene2650_All | 1.884372022 | 1.31561E-39 | kinesin family member C1 | Cell growth and death |
| Unigene6309_All | 1.782401643 | 4.83011E-15 | erine/threonine-protein phosphatase 2A regulatory subunit A | Cell growth and death |
| Unigene2019_All | 1.728846329 | 1.86169E-13 | F-box and WD-40 domain protein CDC4 | Cell growth and death |
| Unigene2345_All | 1.484234875 | 2.01868E-11 | F-box and WD-40 domain protein CDC4 | Cell growth and death |
| Unigene364_All | 1.321419506 | 5.21807E-24 | myosin heavy chain 9/10/11/14 | Cell growth and death |
| Unigene7848_All | 1.286180103 | 2.9684E-16 | mitochondrial division protein 1 | Cell growth and death |
| Unigene8067_All | 1.268737047 | 3.11827E-05 | CDK inhibitor PHO81 | Cell growth and death |
| Unigene7954_All | 1.226823846 | 3.93959E-31 | actin, other eukaryote; | Cell growth and death |
| Unigene872_All | 1.209534921 | 0.0191786 | ADP-ribosylation factor-like protein 2 | Cell growth and death |
| Unigene8321_All | 1.203382322 | 5.01871E-12 | mitotic spindle assembly checkpoint protein MAD2 | Cell growth and death |
| CL979.Contig2_All | 1.149805485 | 6.55413E-47 | kinesin family member 18/19 | Cell growth and death |
| Unigene2302_All | 1.118988634 | 0.04478945 | F-box and WD-40 domain protein CDC4 | Cell growth and death |
| Unigene2282_All | 1.116731228 | 1.09272E-06 | F-box and WD-40 domain protein CDC4 | Cell growth and death |
| Unigene8288_All | 1.083540514 | 0.031993075 | cell division cycle 14 | Cell growth and death |
| Unigene1528_All | 1.03170211 | 4.40519E-20 | anaphase-promoting complex subunit 3 | Cell growth and death |
| Unigene2370_All | 1.014985411 | 0.001724867 | F-box and WD-40 domain protein CDC4 | Cell growth and death |
| Unigene8438_All | 1.004875522 | 1.10461E-12 | Xaa-Pro dipeptidase | Cell growth and death |
| Unigene8687_All | 2.422924998 | 1.104E-153 | apoptosis-inducing factor 2 | aging |
| Unigene6472_All | 2.110121723 | 3.06575E-12 | apoptosis-inducing factor 2 | aging |
| Unigene3526_All | 1.229541542 | 2.13059E-16 | protein O-GlcNAc transferase [EC:2.4.1.255] | Glycan biosynthesis and metabolism |
| Unigene8074_All | 1.221471321 | 1.35746E-06 | dihydrofolate synthase | Metabolism of cofactors and vitamins |
| Unigene2978_All | 1.095026968 | 0.008988842 | NAD+ kinase | Metabolism of cofactors and vitamins |
| Unigene2649_All | 1.020338961 | 0.011993765 | tartrate-resistant acid phosphatase type 5 | Metabolism of cofactors and vitamins |
| CL851.Contig1_All | 4.758316606 | 3.9064E-103 | prephenate dehydratase | amino acid metabolism |
| CL851.Contig2_All | 2.269406166 | 5.12E-113 | threonine synthase | amino acid metabolism |
| Unigene4682_All | 1.569605949 | 1.50486E-20 | trypsin | amino acid metabolism |
| Unigene1494_All | 1.497585751 | 0.000688224 | lysosomal Pro-X carboxypeptidase | amino acid metabolism |
| Unigene3948_All | 1.248318508 | 0.00032535 | 3-hydroxy acid dehydrogenase / malonic semialdehyde reductase | amino acid metabolism |
| Unigene10760_All | 1.179612208 | 3.92547E-15 | argininosuccinate synthase | amino acid metabolism |
| CL32.Contig7_All | 1.149556442 | 3.54188E-24 | cerevisin | amino acid metabolism |
| Unigene589_All | 1.149453359 | 0.000759829 | trypsin [EC:3.4.21.4] | amino acid metabolism |
| Unigene2305_All | 1.102538135 | 4.27187E-07 | sentrin-specific protease 1 | amino acid metabolism |
| Unigene10369_All | 1.056707316 | 1.76108E-24 | sentrin-specific protease 7 | amino acid metabolism |
| Unigene2330_All | 1.04398201 | 1.31407E-10 | sentrin-specific protease 1 | amino acid metabolism |
| CL32.Contig4_All | 1.041767075 | 8.11159E-24 | cerevisin | amino acid metabolism |
| Unigene10123_All | 1.86386832 | 3.77639E-06 | DNA ligase 1 | Replication and repair |
| CL753.Contig3_All | 1.279541254 | 0.031389435 | chromatin modification-related protein YNG2 | transcription |
| Unigene10711_All | 1.231962833 | 1.06127E-13 | U1 small nuclear ribonucleoprotein 70kDa | Transcription |
| Unigene4943_All | 1.187050269 | 0.007447869 | Prp8 binding protein | Transcription |
| Unigene10232_All | 1.162124009 | 0.031308421 | structural maintenance of chromosomes protein 6 | transcription |
| Unigene9131_All | 1.135172526 | 1.1031E-33 | Myb-like DNA-binding protein BAS1 | transcription |
| Unigene5742_All | 1.133134113 | 3.92757E-30 | sister chromatid cohesion protein PDS5 | transcription |
| CL1046.Contig1_All | 1.088955103 | 0.001450522 | nuclear transcription factor Y, gamma | transcription |
| Unigene11988_All | 1.057468978 | 1.18843E-19 | histone H3 | transcription |
| CL716.Contig2_All | 1.117695142 | 3.74263E-45 | molecular chaperone HtpG | Folding, sorting and degradation |
| CL130.Contig2_All | 1.114828529 | 4.66534E-08 | CCR4-NOT transcription complex subunit 6 | Folding, sorting and degradation |
| Unigene9533_All | 1.098322269 | 2.59228E-08 | ubiquitin-conjugating enzyme E2 | Folding, sorting and degradation |
| CL189.Contig3_All | 1.090387583 | 0.000973434 | DnaJ homolog subfamily B member 12 | Folding, sorting and degradation |
| CL801.Contig2_All | 2.486497213 | 2.34953E-36 | large subunit ribosomal protein L7e | Translation |
| CL779.Contig1_All | 2.053951604 | 0.012584038 | large subunit ribosomal protein L11 | Translation |
| Unigene2368_All | 1.499404326 | 1.53714E-39 | U3 small nucleolar RNA-associated protein 13 | Translation |
| Unigene8066_All | 1.115486054 | 0.00012731 | protein-lysine N-methyltransferase EEF2KMT | translation |
| Unigene2643_All | 1.115094452 | 3.6027E-13 | protein-lysine N-methyltransferase EEF2KMT | translation |
| CL889.Contig2_All | 1.093385554 | 0.006270618 | NADH dehydrogenase (ubiquinone) Fe-S protein 6 | Energy metabolism |
| Unigene6402_All | 1.073945967 | 3.80856E-36 | carbonic anhydrase | Energy metabolism |
| Unigene5811_All | 2.505038641 | 2.35103E-29 | UMP-CMP kinase | Nucleotide metabolism |
| CL954.Contig1_All | 1.704070627 | 2.1297E-31 | 3',5'-cyclic-nucleotide phosphodiesterase | Nucleotide metabolism |
| Unigene3375_All | 1.131938147 | 1.07351E-10 | adenylylsulfate kinase | Nucleotide metabolism |
| Unigene4115_All | 1.095940423 | 2.5453E-25 | ribonucleoside-diphosphate reductase subunit M2 | Nucleotide metabolism |
| Unigene11005_All | 7.549098196 | 2.55478E-08 | hypothetical protein | hypothetical protein |
| CL282.Contig3_All | 7.331845389 | 9.85067E-08 | hypothetical protein | hypothetical protein |
| CL617.Contig1_All | 7.168493272 | 3.46668E-07 | hypothetical protein | hypothetical protein |
| Unigene11006_All | 6.768630085 | 6.25831E-07 | hypothetical protein | hypothetical protein |
| CL133.Contig5_All | 6.37028306 | 2.31204E-05 | hypothetical protein | hypothetical protein |
| Unigene8737_All | 5.613651216 | 1.88069E-11 | hypothetical protein | hypothetical protein |
| Unigene8754_All | 5.583834554 | 0.000956518 | hypothetical protein | hypothetical protein |
| CL149.Contig1_All | 5.27011765 | 0.000230927 | hypothetical protein | hypothetical protein |
| Unigene5469_All | 5.190655478 | 0.003884677 | hypothetical protein | hypothetical protein |
| Unigene2096_All | 5.094551387 | 5.88448E-07 | hypothetical protein | hypothetical protein |
| Unigene5407_All | 5.054122961 | 0.00633015 | hypothetical protein | hypothetical protein |
| CL458.Contig2_All | 5.045743144 | 1.63562E-59 | hypothetical protein | hypothetical protein |
| CL973.Contig2_All | 4.980687958 | 0.008247776 | hypothetical protein | hypothetical protein |
| Unigene2599_All | 4.640504911 | 2.05386E-13 | hypothetical protein | hypothetical protein |
| CL261.Contig1_All | 4.629164569 | 3.56499E-31 | hypothetical protein | hypothetical protein |
| CL78.Contig8_All | 4.628429491 | 0.030977586 | hypothetical protein | hypothetical protein |
| Unigene452_All | 4.494350615 | 8.8612E-20 | hypothetical protein | hypothetical protein |
| Unigene6512_All | 4.480490029 | 6.88469E-08 | hypothetical protein | hypothetical protein |
| Unigene6479_All | 4.194723834 | 2.63588E-10 | hypothetical protein | hypothetical protein |
| Unigene4421_All | 3.785632887 | 0.01819421 | hypothetical protein | hypothetical protein |
| CL65.Contig1_All | 3.624922203 | 2.56138E-84 | hypothetical protein | hypothetical protein |
| Unigene7296_All | 3.539789163 | 2.4509E-136 | hypothetical protein | hypothetical protein |
| Unigene7655_All | 3.397854303 | 0.002532182 | hypothetical protein | hypothetical protein |
| Unigene973_All | 3.374496333 | 0.011634099 | hypothetical protein | hypothetical protein |
| Unigene8749_All | 3.333586434 | 5.10357E-08 | hypothetical protein | hypothetical protein |
| Unigene9811_All | 3.299509791 | 2.3521E-259 | hypothetical protein | hypothetical protein |
| CL65.Contig4_All | 3.281575931 | 1.85245E-76 | hypothetical protein | hypothetical protein |
| Unigene5949_All | 3.25443788 | 2.36974E-08 | hypothetical protein | hypothetical protein |
| CL65.Contig6_All | 3.212008983 | 4.7717E-249 | hypothetical protein | hypothetical protein |
| Unigene10848_All | 3.181376474 | 2.8444E-259 | hypothetical protein | hypothetical protein |
| Unigene6716_All | 3.181340042 | 9.21633E-75 | hypothetical protein | hypothetical protein |
| Unigene2311_All | 3.160465727 | 2.36849E-05 | hypothetical protein | hypothetical protein |
| Unigene4889_All | 3.147803847 | 1.43013E-10 | hypothetical protein | hypothetical protein |
| Unigene4267_All | 3.129295447 | 2.61568E-17 | hypothetical protein | hypothetical protein |
| Unigene2127_All | 3.061804539 | 2.7725E-08 | hypothetical protein | hypothetical protein |
| Unigene9195_All | 2.980533148 | 6.0499E-68 | hypothetical protein | hypothetical protein |
| Unigene8329_All | 2.952805337 | 0.000144018 | hypothetical protein | hypothetical protein |
| Unigene8181_All | 2.934449322 | 1.47394E-27 | hypothetical protein | hypothetical protein |
| CL862.Contig1_All | 2.87062488 | 0.009271728 | hypothetical protein | hypothetical protein |
| Unigene8820_All | 2.866181763 | 0.019041582 | hypothetical protein | hypothetical protein |
| Unigene4138_All | 2.811568753 | 4.63795E-07 | hypothetical protein | hypothetical protein |
| Unigene11015_All | 2.795326589 | 0.002972706 | hypothetical protein | hypothetical protein |
| Unigene9885_All | 2.78933923 | 0.000100214 | hypothetical protein | hypothetical protein |
| Unigene11221_All | 2.78519519 | 0.037060724 | hypothetical protein | hypothetical protein |
| Unigene7577_All | 2.768624244 | 1.05563E-07 | hypothetical protein | hypothetical protein |
| Unigene6098_All | 2.768180585 | 1.92112E-48 | hypothetical protein | hypothetical protein |
| Unigene9959_All | 2.737743135 | 0.004651274 | hypothetical protein | hypothetical protein |
| Unigene1221_All | 2.720119491 | 3.46268E-88 | hypothetical protein | hypothetical protein |
| Unigene1320_All | 2.70844686 | 9.75832E-16 | hypothetical protein | hypothetical protein |
| CL206.Contig5_All | 2.686577267 | 2.41582E-08 | hypothetical protein | hypothetical protein |
| Unigene8624_All | 2.629867315 | 3.73066E-12 | hypothetical protein | hypothetical protein |
| Unigene11291_All | 2.581657681 | 1.20028E-33 | hypothetical protein | hypothetical protein |
| CL413.Contig2_All | 2.562041987 | 1.19328E-05 | hypothetical protein | hypothetical protein |
| Unigene963_All | 2.546028611 | 3.41003E-07 | hypothetical protein | hypothetical protein |
| CL206.Contig3_All | 2.543315151 | 3.52928E-11 | hypothetical protein | hypothetical protein |
| CL458.Contig1_All | 2.542698804 | 0.001133405 | hypothetical protein | hypothetical protein |
| CL133.Contig3_All | 2.513618297 | 2.625E-58 | hypothetical protein | hypothetical protein |
| CL633.Contig1_All | 2.494316676 | 4.39186E-51 | hypothetical protein | hypothetical protein |
| Unigene6567_All | 2.456014303 | 0.027738932 | hypothetical protein | hypothetical protein |
| Unigene7191_All | 2.44388818 | 6.16614E-11 | hypothetical protein | hypothetical protein |
| CL257.Contig4_All | 2.401600056 | 1.8476E-19 | hypothetical protein | hypothetical protein |
| Unigene9567_All | 2.376763503 | 6.00153E-61 | hypothetical protein | hypothetical protein |
| CL315.Contig3_All | 2.354727256 | 2.68857E-07 | hypothetical protein | hypothetical protein |
| Unigene9252_All | 2.346680349 | 3.73355E-20 | hypothetical protein | hypothetical protein |
| Unigene4251_All | 2.334547473 | 0.002526603 | hypothetical protein | hypothetical protein |
| Unigene6107_All | 2.318729655 | 1.39403E-15 | hypothetical protein | hypothetical protein |
| CL946.Contig2_All | 2.30812253 | 2.28795E-20 | hypothetical protein | hypothetical protein |
| CL544.Contig2_All | 2.307147136 | 2.29337E-14 | hypothetical protein | hypothetical protein |
| Unigene11008_All | 2.286972391 | 0.00263643 | hypothetical protein | hypothetical protein |
| CL413.Contig1_All | 2.272874916 | 9.36935E-14 | hypothetical protein | hypothetical protein |
| CL982.Contig1_All | 2.269702596 | 2.10705E-24 | hypothetical protein | hypothetical protein |
| Unigene2344_All | 2.264942974 | 1.72737E-31 | hypothetical protein | hypothetical protein |
| Unigene9884_All | 2.264120669 | 9.70306E-05 | hypothetical protein | hypothetical protein |
| CL346.Contig2_All | 2.189320526 | 6.1796E-08 | hypothetical protein | hypothetical protein |
| Unigene10956_All | 2.186541249 | 1.68873E-06 | hypothetical protein | hypothetical protein |
| Unigene3138_All | 2.176309187 | 0.001993482 | hypothetical protein | hypothetical protein |
| Unigene4988_All | 2.175485035 | 8.1871E-193 | hypothetical protein | hypothetical protein |
| CL282.Contig5_All | 2.17510962 | 0.006509192 | hypothetical protein | hypothetical protein |
| Unigene2283_All | 2.145524948 | 0.00752327 | hypothetical protein | hypothetical protein |
| Unigene8383_All | 2.145054176 | 1.96667E-26 | hypothetical protein | hypothetical protein |
| Unigene11031_All | 2.138599061 | 0.001694673 | hypothetical protein | hypothetical protein |
| Unigene10807_All | 2.132735525 | 3.77092E-22 | hypothetical protein | hypothetical protein |
| Unigene7658_All | 2.125907634 | 6.02805E-08 | hypothetical protein | hypothetical protein |
| Unigene2112_All | 2.113361289 | 0.001024994 | hypothetical protein | hypothetical protein |
| Unigene9283_All | 2.106837608 | 2.62026E-10 | hypothetical protein | hypothetical protein |
| CL206.Contig2_All | 2.101958452 | 4.14505E-06 | hypothetical protein | hypothetical protein |
| CL633.Contig2_All | 2.092849738 | 3.21471E-69 | hypothetical protein | hypothetical protein |
| Unigene512_All | 2.080087278 | 2.31457E-28 | hypothetical protein | hypothetical protein |
| Unigene10272_All | 2.078668344 | 1.7444E-140 | hypothetical protein | hypothetical protein |
| CL903.Contig2_All | 2.075261912 | 5.58585E-12 | hypothetical protein | hypothetical protein |
| Unigene8725_All | 2.071287985 | 0.000169359 | hypothetical protein | hypothetical protein |
| Unigene4716_All | 2.068009786 | 2.10751E-06 | hypothetical protein | hypothetical protein |
| Unigene1728_All | 2.054464938 | 7.14221E-08 | hypothetical protein | hypothetical protein |
| CL573.Contig1_All | 2.04207499 | 5.29909E-19 | hypothetical protein | hypothetical protein |
| Unigene2150_All | 2.027950567 | 0.021107859 | hypothetical protein | hypothetical protein |
| CL544.Contig1_All | 2.025537656 | 2.40837E-16 | hypothetical protein | hypothetical protein |
| Unigene4795_All | 2.021717298 | 5.54853E-06 | hypothetical protein | hypothetical protein |
| CL133.Contig4_All | 1.996109425 | 6.88469E-08 | hypothetical protein | hypothetical protein |
| CL661.Contig2_All | 1.984925501 | 0.000848835 | hypothetical protein | hypothetical protein |
| Unigene9509_All | 1.977410009 | 1.21221E-47 | hypothetical protein | hypothetical protein |
| Unigene9317_All | 1.975856878 | 9.13968E-15 | hypothetical protein | hypothetical protein |
| CL575.Contig1_All | 1.965188298 | 2.78461E-09 | hypothetical protein | hypothetical protein |
| Unigene8698_All | 1.963392436 | 7.19508E-06 | hypothetical protein | hypothetical protein |
| Unigene4302_All | 1.962354905 | 0.022135933 | hypothetical protein | hypothetical protein |
| Unigene1669_All | 1.957247695 | 1.50444E-13 | hypothetical protein | hypothetical protein |
| Unigene1754_All | 1.95139048 | 4.04724E-06 | hypothetical protein | hypothetical protein |
| Unigene818_All | 1.944617211 | 1.88302E-20 | hypothetical protein | hypothetical protein |
| Unigene998_All | 1.943400905 | 0.010374274 | hypothetical protein | hypothetical protein |
| Unigene2121_All | 1.938436772 | 0.001591917 | hypothetical protein | hypothetical protein |
| CL983.Contig2_All | 1.926333505 | 4.2067E-99 | hypothetical protein | hypothetical protein |
| Unigene935_All | 1.922405648 | 0.001614103 | hypothetical protein | hypothetical protein |
| Unigene5425_All | 1.904404036 | 0.041941159 | hypothetical protein | hypothetical protein |
| Unigene9847_All | 1.903174607 | 0.000122606 | hypothetical protein | hypothetical protein |
| Unigene954_All | 1.898580747 | 0.022223635 | hypothetical protein | hypothetical protein |
| CL974.Contig1_All | 1.896553818 | 4.574E-07 | hypothetical protein | hypothetical protein |
| Unigene2358_All | 1.895775306 | 8.79617E-19 | hypothetical protein | hypothetical protein |
| Unigene5405_All | 1.872019483 | 0.000937056 | hypothetical protein | hypothetical protein |
| Unigene7109_All | 1.857338375 | 6.75738E-32 | hypothetical protein | hypothetical protein |
| Unigene6893_All | 1.85312556 | 1.73661E-33 | hypothetical protein | hypothetical protein |
| Unigene2862_All | 1.842765541 | 1.63328E-08 | hypothetical protein | hypothetical protein |
| Unigene7822_All | 1.827682171 | 3.83394E-20 | hypothetical protein | hypothetical protein |
| Unigene2073_All | 1.825204877 | 0.000572029 | hypothetical protein | hypothetical protein |
| Unigene1570_All | 1.82306135 | 1.04442E-16 | hypothetical protein | hypothetical protein |
| CL982.Contig2_All | 1.812334809 | 4.29633E-21 | hypothetical protein | hypothetical protein |
| Unigene2336_All | 1.809895665 | 4.24494E-29 | hypothetical protein | hypothetical protein |
| CL527.Contig5_All | 1.806296053 | 2.12449E-32 | hypothetical protein | hypothetical protein |
| Unigene4774_All | 1.805942799 | 0.000197241 | hypothetical protein | hypothetical protein |
| Unigene2102_All | 1.79860432 | 0.009104509 | hypothetical protein | hypothetical protein |
| Unigene3549_All | 1.794323795 | 1.11328E-12 | hypothetical protein | hypothetical protein |
| Unigene6695_All | 1.787393131 | 6.60897E-28 | hypothetical protein | hypothetical protein |
| CL570.Contig1_All | 1.775283256 | 2.89664E-43 | hypothetical protein | hypothetical protein |
| Unigene944_All | 1.774979176 | 0.000244469 | hypothetical protein | hypothetical protein |
| Unigene456_All | 1.768218314 | 6.17263E-10 | hypothetical protein | hypothetical protein |
| CL951.Contig1_All | 1.763184147 | 5.08676E-07 | hypothetical protein | hypothetical protein |
| Unigene5959_All | 1.753154702 | 1.08032E-11 | hypothetical protein | hypothetical protein |
| Unigene600_All | 1.744638084 | 1.07036E-12 | hypothetical protein | hypothetical protein |
| Unigene4485_All | 1.742275558 | 1.26504E-07 | hypothetical protein | hypothetical protein |
| CL1001.Contig1_All | 1.742133822 | 0.002159637 | hypothetical protein | hypothetical protein |
| Unigene7653_All | 1.740651316 | 0.038771708 | hypothetical protein | hypothetical protein |
| Unigene4473_All | 1.738191962 | 2.66773E-09 | hypothetical protein | hypothetical protein |
| CL960.Contig2_All | 1.731277387 | 0.015002637 | hypothetical protein | hypothetical protein |
| CL955.Contig3_All | 1.727026267 | 7.19881E-27 | hypothetical protein | hypothetical protein |
| Unigene3130_All | 1.707096206 | 0.049418454 | hypothetical protein | hypothetical protein |
| CL983.Contig1_All | 1.704135433 | 1.82325E-48 | hypothetical protein | hypothetical protein |
| Unigene8444_All | 1.702392262 | 5.32756E-11 | hypothetical protein | hypothetical protein |
| Unigene6049_All | 1.696553953 | 5.3522E-20 | hypothetical protein | hypothetical protein |
| Unigene5422_All | 1.686307481 | 0.001272767 | hypothetical protein | hypothetical protein |
| CL719.Contig2_All | 1.685556115 | 9.83956E-09 | hypothetical protein | hypothetical protein |
| CL56.Contig1_All | 1.684270744 | 6.93367E-23 | hypothetical protein | hypothetical protein |
| CL747.Contig3_All | 1.68347853 | 6.59149E-24 | hypothetical protein | hypothetical protein |
| Unigene4841_All | 1.677046514 | 3.99096E-07 | hypothetical protein | hypothetical protein |
| CL56.Contig2_All | 1.670602201 | 6.85884E-11 | hypothetical protein | hypothetical protein |
| Unigene928_All | 1.669737353 | 0.000441156 | hypothetical protein | hypothetical protein |
| Unigene6222_All | 1.669412317 | 1.80097E-26 | hypothetical protein | hypothetical protein |
| CL22.Contig1_All | 1.666126959 | 1.01672E-08 | hypothetical protein | hypothetical protein |
| CL387.Contig1_All | 1.663170885 | 7.09395E-10 | hypothetical protein | hypothetical protein |
| Unigene3303_All | 1.662897033 | 5.57367E-40 | hypothetical protein | hypothetical protein |
| Unigene7622_All | 1.656545121 | 0.000217894 | hypothetical protein | hypothetical protein |
| Unigene6715_All | 1.654250387 | 0.020937269 | hypothetical protein | hypothetical protein |
| Unigene8764_All | 1.653957019 | 0.035223093 | hypothetical protein | hypothetical protein |
| CL17.Contig2_All | 1.644297674 | 9.3712E-27 | hypothetical protein | hypothetical protein |
| Unigene7609_All | 1.643767331 | 0.007744925 | hypothetical protein | hypothetical protein |
| Unigene1829_All | 1.640913832 | 1.9062E-13 | hypothetical protein | hypothetical protein |
| Unigene8742_All | 1.635747444 | 0.001903154 | hypothetical protein | hypothetical protein |
| Unigene4453_All | 1.625512673 | 2.73577E-31 | hypothetical protein | hypothetical protein |
| Unigene5369_All | 1.624701609 | 0.007997118 | hypothetical protein | hypothetical protein |
| Unigene5388_All | 1.609691998 | 0.013509574 | hypothetical protein | hypothetical protein |
| Unigene7885_All | 1.606191447 | 6.3942E-05 | hypothetical protein | hypothetical protein |
| CL96.Contig1_All | 1.601389954 | 0.001719693 | hypothetical protein | hypothetical protein |
| Unigene3036_All | 1.579893921 | 9.7475E-27 | hypothetical protein | hypothetical protein |
| Unigene10845_All | 1.57579917 | 8.46841E-05 | hypothetical protein | hypothetical protein |
| Unigene9289_All | 1.570712233 | 2.47043E-09 | hypothetical protein | hypothetical protein |
| Unigene7694_All | 1.567569445 | 0.027864915 | hypothetical protein | hypothetical protein |
| CL40.Contig2_All | 1.564910593 | 1.0545E-08 | hypothetical protein | hypothetical protein |
| Unigene6021_All | 1.560157263 | 3.41423E-07 | hypothetical protein | hypothetical protein |
| CL346.Contig5_All | 1.5578764 | 1.26683E-07 | hypothetical protein | hypothetical protein |
| Unigene5058_All | 1.557416134 | 0.009153278 | hypothetical protein | hypothetical protein |
| Unigene4445_All | 1.556625688 | 1.41955E-21 | hypothetical protein | hypothetical protein |
| Unigene9209_All | 1.549693867 | 2.79709E-17 | hypothetical protein | hypothetical protein |
| Unigene2145_All | 1.543259341 | 0.009460483 | hypothetical protein | hypothetical protein |
| Unigene142_All | 1.542110518 | 1.29896E-28 | hypothetical protein | hypothetical protein |
| Unigene6485_All | 1.539126822 | 0.001202596 | hypothetical protein | hypothetical protein |
| Unigene1991_All | 1.533976919 | 9.6626E-75 | hypothetical protein | hypothetical protein |
| CL395.Contig2_All | 1.528231425 | 0.013235056 | hypothetical protein | hypothetical protein |
| Unigene11990_All | 1.52753589 | 0.001719693 | hypothetical protein | hypothetical protein |
| Unigene8646_All | 1.523538872 | 1.20174E-26 | hypothetical protein | hypothetical protein |
| Unigene6704_All | 1.520592675 | 3.00846E-10 | hypothetical protein | hypothetical protein |
| CL346.Contig4_All | 1.520214164 | 2.23793E-24 | hypothetical protein | hypothetical protein |
| Unigene8232_All | 1.519554293 | 4.51108E-17 | hypothetical protein | hypothetical protein |
| Unigene3451_All | 1.513819171 | 4.11302E-96 | hypothetical protein | hypothetical protein |
| Unigene3299_All | 1.511174923 | 9.87799E-17 | hypothetical protein | hypothetical protein |
| CL213.Contig1_All | 1.510519728 | 9.21871E-27 | hypothetical protein | hypothetical protein |
| CL282.Contig8_All | 1.508549689 | 1.84756E-23 | hypothetical protein | hypothetical protein |
| Unigene8750_All | 1.505421325 | 0.041655363 | hypothetical protein | hypothetical protein |
| Unigene8636_All | 1.489558965 | 3.56499E-31 | hypothetical protein | hypothetical protein |
| CL1042.Contig1_All | 1.487828939 | 1.46054E-08 | hypothetical protein | hypothetical protein |
| Unigene4257_All | 1.471969359 | 0.010951489 | hypothetical protein | hypothetical protein |
| Unigene3319_All | 1.468572401 | 1.90774E-17 | hypothetical protein | hypothetical protein |
| CL758.Contig2_All | 1.466944188 | 6.78214E-11 | hypothetical protein | hypothetical protein |
| CL315.Contig4_All | 1.463965752 | 2.31953E-12 | hypothetical protein | hypothetical protein |
| CL85.Contig1_All | 1.459537824 | 0.000414555 | hypothetical protein | hypothetical protein |
| Unigene2703_All | 1.459196231 | 0.002051315 | hypothetical protein | hypothetical protein |
| Unigene10994_All | 1.458889449 | 0.035025507 | hypothetical protein | hypothetical protein |
| Unigene7241_All | 1.455173791 | 1.75922E-15 | hypothetical protein | hypothetical protein |
| CL573.Contig2_All | 1.454723821 | 8.44228E-25 | hypothetical protein | hypothetical protein |
| Unigene10293_All | 1.45075059 | 7.79857E-05 | hypothetical protein | hypothetical protein |
| Unigene9488_All | 1.447163047 | 0.000964451 | hypothetical protein | hypothetical protein |
| Unigene4062_All | 1.437929306 | 0.023461976 | hypothetical protein | hypothetical protein |
| Unigene1975_All | 1.434214558 | 2.52812E-21 | hypothetical protein | hypothetical protein |
| Unigene850_All | 1.434089885 | 0.000159235 | hypothetical protein | hypothetical protein |
| Unigene1216_All | 1.432393863 | 2.09733E-28 | hypothetical protein | hypothetical protein |
| Unigene3326_All | 1.428536068 | 1.65474E-37 | hypothetical protein | hypothetical protein |
| Unigene2509_All | 1.427081228 | 0.003882486 | hypothetical protein | hypothetical protein |
| Unigene5108_All | 1.423618224 | 0.01021324 | hypothetical protein | hypothetical protein |
| Unigene10522_All | 1.41780166 | 0.001541785 | hypothetical protein | hypothetical protein |
| Unigene3873_All | 1.416015598 | 4.51249E-05 | hypothetical protein | hypothetical protein |
| CL345.Contig2_All | 1.410539154 | 1.5138E-36 | hypothetical protein | hypothetical protein |
| CL22.Contig10_All | 1.410222248 | 4.30247E-08 | hypothetical protein | hypothetical protein |
| CL955.Contig1_All | 1.408911043 | 2.92148E-07 | hypothetical protein | hypothetical protein |
| Unigene339_All | 1.407000528 | 2.81237E-08 | hypothetical protein | hypothetical protein |
| Unigene3316_All | 1.406151446 | 5.67573E-05 | hypothetical protein | hypothetical protein |
| Unigene1614_All | 1.401899662 | 1.73246E-28 | hypothetical protein | hypothetical protein |
| CL677.Contig2_All | 1.401688848 | 6.05802E-09 | hypothetical protein | hypothetical protein |
| Unigene9394_All | 1.395997478 | 1.41395E-10 | hypothetical protein | hypothetical protein |
| CL1023.Contig1_All | 1.39484413 | 2.52987E-30 | hypothetical protein | hypothetical protein |
| Unigene10782_All | 1.394493087 | 9.56096E-08 | hypothetical protein | hypothetical protein |
| Unigene402_All | 1.392953493 | 0.007614981 | hypothetical protein | hypothetical protein |
| Unigene2108_All | 1.382594487 | 0.005603738 | hypothetical protein | hypothetical protein |
| Unigene942_All | 1.38193746 | 2.64523E-05 | hypothetical protein | hypothetical protein |
| Unigene10643_All | 1.379823398 | 3.34344E-05 | hypothetical protein | hypothetical protein |
| Unigene6706_All | 1.379691581 | 1.39416E-10 | hypothetical protein | hypothetical protein |
| Unigene1756_All | 1.370666836 | 0.005062521 | hypothetical protein | hypothetical protein |
| Unigene227_All | 1.370523995 | 5.34525E-24 | hypothetical protein | hypothetical protein |
| CL276.Contig2_All | 1.369636349 | 1.29601E-26 | hypothetical protein | hypothetical protein |
| Unigene2399_All | 1.369611195 | 8.53257E-07 | hypothetical protein | hypothetical protein |
| Unigene967_All | 1.365934782 | 0.017351908 | hypothetical protein | hypothetical protein |
| CL200.Contig1_All | 1.362638398 | 0.0182005 | hypothetical protein | hypothetical protein |
| Unigene4033_All | 1.362444311 | 2.08184E-60 | hypothetical protein | hypothetical protein |
| CL917.Contig2_All | 1.359912684 | 0.008922029 | hypothetical protein | hypothetical protein |
| Unigene3135_All | 1.359303051 | 0.030660766 | hypothetical protein | hypothetical protein |
| Unigene3952_All | 1.359213875 | 0.000116611 | hypothetical protein | hypothetical protein |
| Unigene957_All | 1.354043493 | 0.000444135 | hypothetical protein | hypothetical protein |
| Unigene3653_All | 1.351444233 | 1.84369E-05 | hypothetical protein | hypothetical protein |
| Unigene6705_All | 1.349022607 | 0.013069787 | hypothetical protein | hypothetical protein |
| Unigene3335_All | 1.348428486 | 3.69249E-07 | hypothetical protein | hypothetical protein |
| Unigene7371_All | 1.343628335 | 4.16239E-23 | hypothetical protein | hypothetical protein |
| Unigene1603_All | 1.331670251 | 2.33036E-19 | hypothetical protein | hypothetical protein |
| Unigene7155_All | 1.331543661 | 0.007782105 | hypothetical protein | hypothetical protein |
| Unigene1820_All | 1.329778141 | 0.006977768 | hypothetical protein | hypothetical protein |
| Unigene9841_All | 1.327174119 | 0.000685552 | hypothetical protein | hypothetical protein |
| Unigene1818_All | 1.327133695 | 0.001201466 | hypothetical protein | hypothetical protein |
| Unigene4233_All | 1.323763974 | 0.010973157 | hypothetical protein | hypothetical protein |
| Unigene615_All | 1.32169358 | 4.94728E-05 | hypothetical protein | hypothetical protein |
| Unigene6131_All | 1.321217242 | 1.01892E-06 | hypothetical protein | hypothetical protein |
| Unigene6326_All | 1.317342504 | 6.40437E-09 | hypothetical protein | hypothetical protein |
| Unigene7215_All | 1.31682343 | 0.001028708 | hypothetical protein | hypothetical protein |
| Unigene2573_All | 1.314260336 | 2.81698E-06 | hypothetical protein | hypothetical protein |
| Unigene7589_All | 1.313270176 | 2.05787E-07 | hypothetical protein | hypothetical protein |
| CL109.Contig5_All | 1.312725165 | 0.031381112 | hypothetical protein | hypothetical protein |
| CL524.Contig1_All | 1.310894402 | 2.95938E-05 | hypothetical protein | hypothetical protein |
| Unigene3321_All | 1.306122286 | 3.77452E-06 | hypothetical protein | hypothetical protein |
| Unigene7534_All | 1.306067724 | 2.89696E-11 | hypothetical protein | hypothetical protein |
| Unigene5258_All | 1.305202857 | 0.015661341 | hypothetical protein | hypothetical protein |
| CL653.Contig1_All | 1.304407296 | 6.65914E-23 | hypothetical protein | hypothetical protein |
| CL642.Contig3_All | 1.304186837 | 2.54127E-05 | hypothetical protein | hypothetical protein |
| CL65.Contig3_All | 1.302103779 | 1.09617E-16 | hypothetical protein | hypothetical protein |
| Unigene4241_All | 1.29981127 | 2.53788E-39 | hypothetical protein | hypothetical protein |
| Unigene1191_All | 1.299636021 | 1.8128E-07 | hypothetical protein | hypothetical protein |
| Unigene12216_All | 1.293946703 | 1.0536E-49 | hypothetical protein | hypothetical protein |
| CL107.Contig1_All | 1.289581526 | 2.83112E-26 | hypothetical protein | hypothetical protein |
| CL420.Contig3_All | 1.289394577 | 8.80382E-12 | hypothetical protein | hypothetical protein |
| Unigene3136_All | 1.286876536 | 0.026926174 | hypothetical protein | hypothetical protein |
| Unigene2655_All | 1.285419586 | 0.00015932 | hypothetical protein | hypothetical protein |
| CL527.Contig2_All | 1.284742308 | 4.97696E-06 | hypothetical protein | hypothetical protein |
| Unigene1159_All | 1.282192676 | 0.000672281 | hypothetical protein | hypothetical protein |
| Unigene1573_All | 1.281954179 | 0.009805102 | hypothetical protein | hypothetical protein |
| Unigene6525_All | 1.278755726 | 0.006125815 | hypothetical protein | hypothetical protein |
| Unigene10902_All | 1.275285916 | 0.024409174 | hypothetical protein | hypothetical protein |
| CL790.Contig3_All | 1.271867578 | 8.04647E-06 | hypothetical protein | hypothetical protein |
| Unigene6519_All | 1.270130583 | 0.029539981 | hypothetical protein | hypothetical protein |
| Unigene5557_All | 1.269280898 | 0.000308028 | hypothetical protein | hypothetical protein |
| Unigene10311_All | 1.267313614 | 3.287E-10 | hypothetical protein | hypothetical protein |
| Unigene1876_All | 1.266987316 | 2.811E-38 | hypothetical protein | hypothetical protein |
| Unigene2723_All | 1.254469886 | 1.23422E-12 | hypothetical protein | hypothetical protein |
| Unigene3315_All | 1.247412752 | 1.56437E-23 | hypothetical protein | hypothetical protein |
| Unigene4232_All | 1.24458227 | 0.047396719 | hypothetical protein | hypothetical protein |
| Unigene1831_All | 1.239830682 | 8.13372E-12 | hypothetical protein | hypothetical protein |
| CL351.Contig2_All | 1.23758806 | 6.322E-38 | hypothetical protein | hypothetical protein |
| Unigene5355_All | 1.235955171 | 0.009739797 | hypothetical protein | hypothetical protein |
| CL186.Contig1_All | 1.233279626 | 3.19559E-05 | hypothetical protein | hypothetical protein |
| CL313.Contig2_All | 1.233014646 | 0.013753332 | hypothetical protein | hypothetical protein |
| Unigene1_All | 1.227822506 | 1.22106E-10 | hypothetical protein | hypothetical protein |
| Unigene7541_All | 1.227251339 | 0.015547701 | hypothetical protein | hypothetical protein |
| Unigene3007_All | 1.226533203 | 1.28155E-07 | hypothetical protein | hypothetical protein |
| Unigene3302_All | 1.218758621 | 1.11777E-13 | hypothetical protein | hypothetical protein |
| Unigene1247_All | 1.21670202 | 1.53738E-13 | hypothetical protein | hypothetical protein |
| Unigene3573_All | 1.213474816 | 0.004251566 | hypothetical protein | hypothetical protein |
| CL141.Contig6_All | 1.213018716 | 3.33973E-07 | hypothetical protein | hypothetical protein |
| Unigene9224_All | 1.210975802 | 3.56541E-10 | hypothetical protein | hypothetical protein |
| Unigene9082_All | 1.20571621 | 1.31727E-50 | hypothetical protein | hypothetical protein |
| Unigene8730_All | 1.204413711 | 0.028433951 | hypothetical protein | hypothetical protein |
| CL575.Contig2_All | 1.204143382 | 0.004156977 | hypothetical protein | hypothetical protein |
| Unigene8328_All | 1.200508663 | 0.013001034 | hypothetical protein | hypothetical protein |
| CL183.Contig3_All | 1.200263464 | 3.92656E-07 | hypothetical protein | hypothetical protein |
| Unigene9233_All | 1.19940556 | 2.21522E-10 | hypothetical protein | hypothetical protein |
| CL677.Contig1_All | 1.197453846 | 3.47773E-68 | hypothetical protein | hypothetical protein |
| Unigene5280_All | 1.196427751 | 0.012327452 | hypothetical protein | hypothetical protein |
| Unigene332_All | 1.193653872 | 1.00594E-12 | hypothetical protein | hypothetical protein |
| CL22.Contig19_All | 1.191531995 | 3.51208E-11 | hypothetical protein | hypothetical protein |
| Unigene179_All | 1.190968344 | 1.02993E-06 | hypothetical protein | hypothetical protein |
| Unigene3312_All | 1.190159011 | 1.01595E-19 | hypothetical protein | hypothetical protein |
| CL119.Contig1_All | 1.18431196 | 4.21671E-05 | hypothetical protein | hypothetical protein |
| Unigene8158_All | 1.183870699 | 7.02924E-13 | hypothetical protein | hypothetical protein |
| Unigene4905_All | 1.182535794 | 3.38152E-08 | hypothetical protein | hypothetical protein |
| Unigene9255_All | 1.171288424 | 4.22877E-08 | hypothetical protein | hypothetical protein |
| Unigene1174_All | 1.170249586 | 1.8989E-07 | hypothetical protein | hypothetical protein |
| Unigene10879_All | 1.1688377 | 1.32972E-20 | hypothetical protein | hypothetical protein |
| Unigene582_All | 1.165765325 | 0.015982149 | hypothetical protein | hypothetical protein |
| CL22.Contig5_All | 1.16319721 | 1.22353E-09 | hypothetical protein | hypothetical protein |
| Unigene8823_All | 1.161545191 | 1.33077E-34 | hypothetical protein | hypothetical protein |
| Unigene8459_All | 1.159421257 | 7.68599E-08 | hypothetical protein | hypothetical protein |
| Unigene8364_All | 1.15623537 | 3.9736E-11 | hypothetical protein | hypothetical protein |
| Unigene5701_All | 1.153372452 | 0.000270997 | hypothetical protein | hypothetical protein |
| Unigene3970_All | 1.142023018 | 0.000230976 | hypothetical protein | hypothetical protein |
| CL213.Contig2_All | 1.13881343 | 1.2841E-05 | hypothetical protein | hypothetical protein |
| CL299.Contig3_All | 1.138283738 | 0.021693224 | hypothetical protein | hypothetical protein |
| Unigene430_All | 1.137206445 | 1.36202E-23 | hypothetical protein | hypothetical protein |
| CL346.Contig3_All | 1.136425678 | 2.64682E-28 | hypothetical protein | hypothetical protein |
| CL1051.Contig5_All | 1.134549739 | 2.99846E-06 | hypothetical protein | hypothetical protein |
| Unigene1588_All | 1.134134341 | 5.7824E-14 | hypothetical protein | hypothetical protein |
| CL443.Contig3_All | 1.133177682 | 1.82325E-05 | hypothetical protein | hypothetical protein |
| CL297.Contig2_All | 1.131592231 | 1.30858E-15 | hypothetical protein | hypothetical protein |
| Unigene3986_All | 1.130349159 | 0.010947098 | hypothetical protein | hypothetical protein |
| Unigene2169_All | 1.12565828 | 5.4866E-06 | hypothetical protein | hypothetical protein |
| Unigene7145_All | 1.122852108 | 1.07502E-15 | hypothetical protein | hypothetical protein |
| Unigene3330_All | 1.122192049 | 4.33222E-28 | hypothetical protein | hypothetical protein |
| Unigene9336_All | 1.121241212 | 0.007358372 | hypothetical protein | hypothetical protein |
| Unigene8947_All | 1.121118499 | 0.003050406 | hypothetical protein | hypothetical protein |
| Unigene7625_All | 1.120563973 | 0.034275699 | hypothetical protein | hypothetical protein |
| Unigene3824_All | 1.119597737 | 0.010149965 | hypothetical protein | hypothetical protein |
| CL624.Contig1_All | 1.119397986 | 1.79076E-05 | hypothetical protein | hypothetical protein |
| Unigene2410_All | 1.117364257 | 1.3161E-05 | hypothetical protein | hypothetical protein |
| Unigene5937_All | 1.117310843 | 3.22887E-07 | hypothetical protein | hypothetical protein |
| Unigene4089_All | 1.115266998 | 3.66816E-25 | hypothetical protein | hypothetical protein |
| Unigene5982_All | 1.113486338 | 0.004809449 | hypothetical protein | hypothetical protein |
| Unigene6154_All | 1.11153308 | 0.018660497 | hypothetical protein | hypothetical protein |
| Unigene3695_All | 1.111334428 | 0.001209038 | hypothetical protein | hypothetical protein |
| CL732.Contig1_All | 1.109246217 | 7.25588E-07 | hypothetical protein | hypothetical protein |
| Unigene508_All | 1.109183167 | 0.002117105 | hypothetical protein | hypothetical protein |
| Unigene5114_All | 1.107597633 | 0.011390801 | hypothetical protein | hypothetical protein |
| Unigene4942_All | 1.106604136 | 2.34852E-05 | hypothetical protein | hypothetical protein |
| CL527.Contig1_All | 1.105854816 | 0.013139849 | hypothetical protein | hypothetical protein |
| CL798.Contig1_All | 1.105853809 | 1.53407E-05 | hypothetical protein | hypothetical protein |
| CL853.Contig2_All | 1.104100529 | 3.77866E-05 | hypothetical protein | hypothetical protein |
| Unigene2291_All | 1.10324944 | 0.00053617 | hypothetical protein | hypothetical protein |
| CL955.Contig2_All | 1.098638303 | 0.000220094 | hypothetical protein | hypothetical protein |
| CL527.Contig4_All | 1.093379986 | 1.29743E-08 | hypothetical protein | hypothetical protein |
| Unigene1683_All | 1.09232225 | 0.008425889 | hypothetical protein | hypothetical protein |
| Unigene1323_All | 1.091386019 | 9.97051E-05 | hypothetical protein | hypothetical protein |
| Unigene4016_All | 1.090019932 | 2.05478E-14 | hypothetical protein | hypothetical protein |
| Unigene9829_All | 1.086957995 | 0.003098955 | hypothetical protein | hypothetical protein |
| Unigene385_All | 1.086081293 | 8.31889E-12 | hypothetical protein | hypothetical protein |
| Unigene2281_All | 1.082503923 | 3.37554E-15 | hypothetical protein | hypothetical protein |
| Unigene8902_All | 1.081391934 | 3.19647E-51 | hypothetical protein | hypothetical protein |
| CL109.Contig6_All | 1.07920963 | 0.020433002 | hypothetical protein | hypothetical protein |
| Unigene7570_All | 1.078762551 | 4.88933E-29 | hypothetical protein | hypothetical protein |
| CL752.Contig2_All | 1.078732197 | 0.007450637 | hypothetical protein | hypothetical protein |
| Unigene5738_All | 1.078351145 | 0.000125712 | hypothetical protein | hypothetical protein |
| Unigene4775_All | 1.078075126 | 0.006608937 | hypothetical protein | hypothetical protein |
| CL417.Contig1_All | 1.077205228 | 0.003065606 | hypothetical protein | hypothetical protein |
| Unigene5796_All | 1.07402418 | 1.43045E-06 | hypothetical protein | hypothetical protein |
| Unigene5957_All | 1.071040061 | 0.000986449 | hypothetical protein | hypothetical protein |
| Unigene1858_All | 1.070811985 | 0.027031662 | hypothetical protein | hypothetical protein |
| Unigene10249_All | 1.070367853 | 0.001880596 | hypothetical protein | hypothetical protein |
| Unigene8017_All | 1.069091468 | 1.21393E-14 | hypothetical protein | hypothetical protein |
| CL119.Contig2_All | 1.068204414 | 1.20132E-43 | hypothetical protein | hypothetical protein |
| Unigene10160_All | 1.066312208 | 4.6698E-13 | hypothetical protein | hypothetical protein |
| Unigene2623_All | 1.064592997 | 5.43733E-20 | hypothetical protein | hypothetical protein |
| CL257.Contig3_All | 1.064374863 | 7.26691E-11 | hypothetical protein | hypothetical protein |
| CL704.Contig3_All | 1.060095363 | 6.40808E-23 | hypothetical protein | hypothetical protein |
| Unigene7256_All | 1.058333595 | 1.08754E-13 | hypothetical protein | hypothetical protein |
| Unigene10987_All | 1.056916926 | 0.001960222 | hypothetical protein | hypothetical protein |
| CL474.Contig4_All | 1.056787417 | 1.43871E-29 | hypothetical protein | hypothetical protein |
| Unigene1555_All | 1.056730126 | 4.00833E-10 | hypothetical protein | hypothetical protein |
| CL176.Contig4_All | 1.056209543 | 1.01741E-31 | hypothetical protein | hypothetical protein |
| Unigene2439_All | 1.056004832 | 1.98045E-06 | hypothetical protein | hypothetical protein |
| Unigene1549_All | 1.055779849 | 0.002830309 | hypothetical protein | hypothetical protein |
| CL900.Contig1_All | 1.054678329 | 0.035285454 | hypothetical protein | hypothetical protein |
| CL478.Contig2_All | 1.053692518 | 4.56117E-05 | hypothetical protein | hypothetical protein |
| CL22.Contig13_All | 1.052054755 | 6.78726E-17 | hypothetical protein | hypothetical protein |
| Unigene9160_All | 1.051502157 | 0.008660637 | hypothetical protein | hypothetical protein |
| Unigene4092_All | 1.049418097 | 1.09996E-36 | hypothetical protein | hypothetical protein |
| CL284.Contig1_All | 1.049088456 | 0.003377726 | hypothetical protein | hypothetical protein |
| CL570.Contig2_All | 1.047330705 | 3.4649E-08 | hypothetical protein | hypothetical protein |
| Unigene45_All | 1.043345677 | 5.79018E-13 | hypothetical protein | hypothetical protein |
| CL686.Contig2_All | 1.042855814 | 0.030426595 | hypothetical protein | hypothetical protein |
| Unigene3346_All | 1.038446981 | 4.82292E-08 | hypothetical protein | hypothetical protein |
| Unigene3748_All | 1.035474975 | 2.6339E-18 | hypothetical protein | hypothetical protein |
| Unigene4105_All | 1.034284375 | 0.01383781 | hypothetical protein | hypothetical protein |
| Unigene2526_All | 1.029410531 | 3.37957E-08 | hypothetical protein | hypothetical protein |
| Unigene1661_All | 1.026571989 | 0.00158421 | hypothetical protein | hypothetical protein |
| CL688.Contig1_All | 1.022986363 | 1.14425E-16 | hypothetical protein | hypothetical protein |
| Unigene10649_All | 1.022250784 | 3.69815E-16 | hypothetical protein | hypothetical protein |
| Unigene9443_All | 1.019755021 | 0.033630614 | hypothetical protein | hypothetical protein |
| Unigene2540_All | 1.017664305 | 0.014579211 | hypothetical protein | hypothetical protein |
| Unigene6259_All | 1.015501841 | 1.18214E-10 | hypothetical protein | hypothetical protein |
| CL17.Contig1_All | 1.015335503 | 7.35648E-07 | hypothetical protein | hypothetical protein |
| CL153.Contig2_All | 1.014557546 | 0.030977586 | hypothetical protein | hypothetical protein |
| Unigene3347_All | 1.014117646 | 3.66748E-13 | hypothetical protein | hypothetical protein |
| Unigene4644_All | 1.013674955 | 1.22931E-06 | hypothetical protein | hypothetical protein |
| Unigene7463_All | 1.0130223 | 0.038251156 | hypothetical protein | hypothetical protein |
| Unigene2049_All | 1.011647162 | 1.34346E-12 | hypothetical protein | hypothetical protein |
| Unigene3802_All | 1.011622099 | 5.60846E-14 | hypothetical protein | hypothetical protein |
| Unigene2482_All | 1.011176895 | 0.003891499 | hypothetical protein | hypothetical protein |
| Unigene6451_All | 1.008640738 | 1.05881E-15 | hypothetical protein | hypothetical protein |
| CL706.Contig3_All | 1.000986864 | 0.007784979 | hypothetical protein | hypothetical protein |
| CL745.Contig2_All | 1.000366169 | 3.41879E-47 | hypothetical protein | hypothetical protein |
| Unigene4169_All | -1.023048274 | 6.22939E-20 | enoyl-CoA hydratase | lipid metabolism |
| Unigene4728_All | -1.027814563 | 1.97057E-07 | phosphatidylinositol transfer protein | lipid metabolism |
| Unigene3874_All | -1.032892919 | 1.17574E-11 | phytanoyl-CoA hydroxylase | lipid metabolism |
| Unigene1875_All | -1.085765556 | 1.85204E-15 | acyl-CoA dehydrogenase | lipid metabolism |
| Unigene1393_All | -1.118206061 | 6.52247E-17 | sn1-specific diacylglycerol lipase | lipid metabolism |
| Unigene9831_All | -1.165361997 | 0.00337042 | sphingolipid 8-(E)-desaturase | lipid metabolism |
| Unigene3739_All | -1.536451026 | 3.29542E-39 | short-chain 2-methylacyl-CoA dehydrogenase | lipid metabolism |
| Unigene151_All | -1.550808845 | 2.94616E-17 | sn1-specific diacylglycerol lipase | lipid metabolism |
| Unigene4825_All | -1.620131639 | 4.77098E-09 | 2-acylglycerol O-acyltransferase 2 | lipid metabolism |
| CL271.Contig1_All | -1.997426815 | 3.9992E-74 | long-chain acyl-CoA synthetase | lipid metabolism |
| Unigene9763_All | -1.01778644 | 9.87514E-31 | all-trans-retinol dehydrogenase (NAD+) | carbohydrate metabolism |
| Unigene8600_All | -1.029638494 | 2.35632E-05 | succinate dehydrogenase (ubiquinone) membrane anchor subunit | carbohydrate metabolism |
| Unigene4907_All | -1.071005295 | 0.01900978 | protein SDA1 | carbohydrate metabolism |
| Unigene817_All | -1.085141653 | 7.13382E-29 | alpha-methylacyl-CoA racemase | Transport and catabolism |
| CL561.Contig2_All | -1.086981172 | 0.010510135 | NADH:quinone reductase (non-electrogenic) | carbohydrate metabolism |
| Unigene2970_All | -1.090115798 | 6.52247E-17 | oxalate---CoA ligase | carbohydrate metabolism |
| Unigene5967_All | -1.127649496 | 0.026148469 | succinate dehydrogenase | carbohydrate metabolism |
| Unigene5568_All | -1.133899736 | 3.04179E-10 | peroxisomal enoyl-CoA hydratase 2 | carbohydrate metabolism |
| Unigene1877_All | -1.19336558 | 5.67102E-61 | aldehyde dehydrogenase (NAD+) | carbohydrate metabolism |
| Unigene250_All | -1.194450901 | 3.11387E-14 | salicylate hydroxylase | carbohydrate metabolism |
| Unigene123_All | -1.209439801 | 2.79978E-28 | 2-hydroxyacyl-CoA lyase | Transport and catabolism |
| CL8.Contig1_All | -1.229947471 | 1.3625E-26 | carnitine O-acetyltransferase | Transport and catabolism |
| Unigene2514_All | -1.283807713 | 8.04381E-19 | pyruvate, water dikinase | carbohydrate metabolism |
| CL957.Contig1_All | -1.288260194 | 5.43154E-05 | retinol dehydrogenase 12 | carbohydrate metabolism |
| CL836.Contig1_All | -1.342479917 | 1.87632E-83 | oxalate---CoA ligase | carbohydrate metabolism |
| Unigene10776_All | -1.367607065 | 1.21148E-35 | epsilon-lactone hydrolase | carbohydrate metabolism |
| Unigene7506_All | -1.38224653 | 1.75999E-25 | NADPH:quinone reductase | carbohydrate metabolism |
| CL628.Contig2_All | -1.485089276 | 1.48354E-32 | glutamine synthetase | carbohydrate metabolism |
| Unigene7545_All | -1.491037051 | 3.63314E-07 | phytanoyl-CoA hydroxylase | Transport and catabolism |
| Unigene8299_All | -1.510182061 | 4.30267E-08 | glutamine synthetase | carbohydrate metabolism |
| CL509.Contig1_All | -1.687714337 | 7.58241E-07 | retinol dehydrogenase 12 | carbohydrate metabolism |
| Unigene868_All | -1.756960523 | 5.013E-37 | inositol oxygenase | carbohydrate metabolism |
| Unigene2453_All | -1.818521219 | 3.52395E-63 | NADPH oxidase 2 | carbohydrate metabolism |
| Unigene1996_All | -1.936986551 | 1.93712E-12 | acid phosphatase type 7 | carbohydrate metabolism |
| Unigene2635_All | -2.650680543 | 2.7141E-110 | sorbose reductase | carbohydrate metabolism |
| CL803.Contig4_All | -2.755032724 | 0.001523373 | glucan endo-1,3-alpha-glucosidase | carbohydrate metabolism |
| Unigene8785_All | -3.243632954 | 1.20725E-18 | NADPH2 dehydrogenase | carbohydrate metabolism |
| CL694.Contig1_All | -1.046540186 | 8.33435E-70 | RHO1 GDP-GTP exchange protein 1/2 | signal transduction |
| Unigene328_All | -1.061238161 | 5.80847E-35 | Rab proteins geranylgeranyltransferase component A | signal transduction |
| Unigene10839_All | -1.069537857 | 1.0119E-51 | rabconnectin-3a | signal transduction |
| CL1020.Contig1_All | -1.072865027 | 9.61415E-09 | SNF1-activating kinase 1 | signal transduction |
| Unigene9296_All | -1.081164723 | 3.45686E-07 | tyrosine-protein phosphatase 2/3 | signal transduction |
| CL72.Contig3_All | -1.107235384 | 0.000493969 | SNF1-activating kinase 1 | signal transduction |
| CL310.Contig2_All | -1.233521725 | 1.27322E-11 | serine/threonine-protein kinase CHEK2 | signal transduction |
| CL310.Contig1_All | -1.385218316 | 8.0866E-76 | serine/threonine-protein kinase CHEK2 | signal transduction |
| CL947.Contig1_All | -1.458064063 | 0.00036488 | platelet-activating factor acetylhydrolase IB subunit alpha+ | signal transduction |
| Unigene2470_All | -1.618773111 | 2.36493E-10 | ser/thr/tyr protein kinase RAD53 | signal transduction |
| Unigene164_All | -1.695062317 | 8.32012E-49 | atypical dual specificity phosphatase | signal transduction |
| Unigene1706_All | -1.766717476 | 3.50472E-23 | atypical dual specificity phosphatase | signal transduction |
| CL482.Contig2_All | -2.323462425 | 1.0283E-222 | ELMO domain-containing protein | signal transduction |
| Unigene5478_All | -2.774663407 | 3.16561E-14 | Ras-related protein Rab-5C | signal transduction |
| Unigene9833_All | -3.579516644 | 8.59722E-58 | serum/glucocorticoid-regulated kinase 2 | signal transduction |
| Unigene10372_All | -1.006151222 | 2.77667E-18 | sorting nexin-3 | Transport and catabolism |
| Unigene8343_All | -1.207534894 | 2.86465E-06 | charged multivesicular body protein 4A/B | Transport and catabolism |
| Unigene8728_All | -1.284653034 | 0.010299528 | multidrug resistance protein, MATE family | Transport and catabolism |
| Unigene9929_All | -1.450604779 | 0.046144418 | solute carrier family 26 (sodium-independent sulfate anion transporter), member 11 | Transport and catabolism |
| Unigene753_All | -1.465460932 | 2.15018E-12 | amphiphysin | Transport and catabolism |
| Unigene7733_All | -1.493859363 | 0.005088697 | dynein cytoplasmic 1 intermediate chain | Transport and catabolism |
| Unigene3227_All | -1.834531688 | 0.024732139 | dynein cytoplasmic 1 intermediate chain | Transport and catabolism |
| CL182.Contig2_All | -1.024234369 | 1.14377E-27 | ammonium transporter | Membrane transport |
| Unigene1491_All | -1.053533522 | 1.55369E-08 | Ca2+:H+ antiporter | Membrane transport |
| Unigene10191_All | -1.075556352 | 6.20247E-05 | ATP_binding cassette | Membrane transport |
| CL812.Contig2_All | -1.176062647 | 5.80078E-05 | ATP_binding cassette | Membrane transport |
| Unigene2087_All | -1.204937129 | 0.000506165 | MFS transporter, UMF1 family | Membrane transport |
| CL182.Contig1_All | -1.208472223 | 2.98749E-29 | ammonium transporter | Membrane transport |
| Unigene5998_All | -1.259263868 | 3.6601E-13 | ATP_binding cassette | Membrane transport |
| Unigene9039_All | -1.290597289 | 1.18751E-24 | ATP_binding cassette | Membrane transport |
| Unigene3601_All | -1.297987577 | 5.9175E-14 | voltage-gated hydrogen channel 1 | Membrane transport |
| Unigene4515_All | -1.334894405 | 3.94932E-64 | ATP_binding cassette | Membrane transport |
| Unigene1388_All | -1.344138172 | 2.35447E-19 | ATP_binding cassette | Membrane transport |
| Unigene8164_All | -1.358957382 | 0.000147048 | coiled-coil domain-containing protein 130 | Membrane transport |
| Unigene2673_All | -1.374535166 | 1.19542E-25 | magnesium transporter | Membrane transport |
| CL397.Contig4_All | -1.605916605 | 8.85734E-72 | ATP_binding cassette | Membrane transport |
| CL491.Contig2_All | -1.665156688 | 3.9757E-114 | ATP_binding cassette | Membrane transport |
| Unigene827_All | -1.769726774 | 4.82861E-13 | ATP-binding cassette | Membrane transport |
| Unigene3149_All | -1.77630553 | 0.002057214 | voltage-dependent calcium channel | Membrane transport |
| Unigene1464_All | -1.854012265 | 1.5459E-45 | ATP_binding cassette | Membrane transport |
| Unigene4119_All | -1.923264748 | 4.54347E-71 | phosphate transporter | Membrane transport |
| Unigene4507_All | -1.972990038 | 9.67546E-67 | ATP_binding cassette | Membrane transport |
| CL448.Contig2_All | -2.861270569 | 5.29919E-13 | high-affinity iron transporter | Membrane transport |
| CL397.Contig1_All | -10.02862528 | 1.77951E-15 | ATP_binding cassette | Membrane transport |
| Unigene469_All | -1.030113626 | 1.41158E-16 | serine/threonine-protein phosphatase PPG1 | Cell growth and death |
| Unigene959_All | -1.196926137 | 0.015557498 | dynactin 1 | Cell growth and death |
| CL104.Contig1_All | -1.215282578 | 1.18613E-26 | cyclin-dependent kinase | Cell growth and death |
| CL180.Contig1_All | -1.876327999 | 1.13076E-05 | dynein light chain LC8-type | Cell growth and death |
| Unigene7727_All | -2.092821338 | 0.030336486 | cell growth-regulating nucleolar protein | Cell growth and death |
| Unigene12389_All | -12.3055373 | 1.15544E-23 | drebrin-like protein | Cell growth and death |
| Unigene11025_All | -6.280006845 | 1.88034E-25 | N-sulfoglucosamine sulfohydrolase | Glycan biosynthesis and metabolism |
| CL743.Contig1_All | -1.082673839 | 0.001976356 | bilirubin oxidase | Metabolism of cofactors and vitamins |
| CL126.Contig1_All | -1.181086642 | 8.64123E-05 | phosphopantothenoylcysteine decarboxylase | Metabolism of cofactors and vitamins |
| Unigene10706_All | -1.234106786 | 1.13031E-11 | pyridoxal 5'-phosphate synthase pdxT subunit | Metabolism of cofactors and vitamins |
| Unigene340_All | -1.058596064 | 4.31527E-24 | hercynylcysteine S-oxide lyase | amino acid metabolism |
| Unigene8116_All | -1.151322868 | 3.70059E-14 | benzoate 4-monooxygenase | amino acid metabolism |
| Unigene10318_All | -1.212382383 | 9.51288E-39 | pro-apoptotic serine protease NMA111 | amino acid metabolism |
| Unigene7226_All | -1.490755138 | 6.33654E-87 | protease IV [EC:3.4.21.-] | amino acid metabolism |
| CL762.Contig2_All | -1.491316732 | 2.22378E-57 | choline dehydrogenase | amino acid metabolism |
| Unigene652_All | -1.896994304 | 6.02142E-11 | 3-hydroxy acid dehydrogenase | amino acid metabolism |
| Unigene737_All | -2.481888361 | 6.1147E-123 | cytochrome P450 | amino acid metabolism |
| Unigene7823_All | -3.332821542 | 5.9957E-278 | cytochrome P450 | amino acid metabolism |
| Unigene3649_All | -1.045950262 | 3.27506E-08 | DNA repair protein RAD57 | Replication and repair |
| Unigene558_All | -1.066033 | 3.64066E-28 | DNA damage-inducible protein 1 | Replication and repair |
| Unigene6074_All | -1.080383539 | 1.56464E-06 | endonuclease V | Replication and repair |
| Unigene1365_All | -1.153771697 | 3.94931E-39 | methylated-DNA-[protein]-cysteine S-methyltransferase | Replication and repair |
| Unigene4266_All | -1.740747713 | 1.61311E-09 | exonuclease 1 | Replication and repair |
| Unigene5849_All | -1.008512771 | 2.05183E-09 | GPN-loop GTPase | transcription |
| CL856.Contig1_All | -1.035089501 | 5.09568E-22 | regulator of chromosome condensation | transcription |
| Unigene9762_All | -1.063680364 | 4.34945E-25 | pre-mRNA-splicing factor ATP-dependent RNA helicase DHX16 | Transcription |
| CL961.Contig1_All | -1.161618006 | 0.016790029 | [histone H3]-dimethyl-L-lysine9 demethylase | transcription |
| Unigene5082_All | -1.256688704 | 1.77116E-31 | plasminogen activator inhibitor 1 RNA-binding protein | transcription |
| Unigene289_All | -1.32084439 | 4.77989E-06 | Myb-like DNA-binding protein BAS1 | transcription |
| Unigene10085_All | -1.415383294 | 4.78221E-24 | protein AATF/BFR2 | transcription |
| CL721.Contig2_All | -1.454945192 | 2.21641E-07 | ATP-dependent DNA helicase | transcription |
| Unigene1901_All | -1.568646979 | 8.125E-46 | RNA 3'-terminal phosphate cyclase (ATP) | transcription |
| CL25.Contig1_All | -1.683454525 | 2.01473E-18 | small nuclear ribonucleoprotein F | Transcription |
| Unigene3163_All | -1.756288563 | 6.40241E-06 | DNA polymerase eta | transcription |
| Unigene3691_All | -2.03982511 | 1.19542E-25 | Myb-like DNA-binding protein | transcription |
| Unigene1165_All | -2.268873989 | 1.5021E-140 | Myb-like DNA-binding protein | transcription |
| Unigene1088_All | -3.004126617 | 0.04571958 | DNA polymerase eta | transcription |
| CL753.Contig4_All | -4.543699611 | 0.00030715 | chromatin modification-related protein YNG2 | transcription |
| CL113.Contig2_All | -1.08796837 | 5.75956E-56 | HSP20 family protein | Folding, sorting and degradation |
| Unigene3217_All | -1.248772506 | 0.036436121 | CCR4-NOT transcription complex subunit | Folding, sorting and degradation |
| Unigene951_All | -1.271815985 | 0.000984056 | exosome complex component RRP41 | Folding, sorting and degradation |
| Unigene302_All | -1.386241812 | 6.15043E-11 | exosome complex component RRP40 | Folding, sorting and degradation |
| Unigene7939_All | -1.494374565 | 1.7224E-102 | proteasome activator subunit 4 | Folding, sorting and degradation |
| Unigene3719_All | -1.622010122 | 4.60712E-18 | CCR4-NOT transcription complex subunit 6 | Folding, sorting and degradation |
| Unigene2515_All | -1.04675425 | 6.44107E-13 | elongation factor G | translation |
| Unigene213_All | -1.071613578 | 0.000821967 | L-threonylcarbamoyladenylate synthase | translation |
| Unigene5700_All | -1.091197794 | 5.26897E-13 | U3 small nucleolar RNA-associated protein 22 | Translation |
| Unigene5964_All | -1.092434564 | 2.01473E-18 | type I protein arginine methyltransferase | translation |
| Unigene1577_All | -1.107053048 | 2.89815E-10 | cleavage and polyadenylation specificity factor subunit 3 | Translation |
| Unigene10090_All | -1.108514079 | 1.64298E-33 | E3 ubiquitin-protein ligase UBR1 | translation |
| CL926.Contig2_All | -1.119018003 | 1.9062E-84 | tRNA-specific adenosine deaminase 3 | translation |
| Unigene9203_All | -1.144702841 | 2.4682E-10 | RNA 3'-terminal phosphate cyclase-like protein | Translation |
| CL670.Contig1_All | -1.160466293 | 4.77434E-22 | rRNA 2'-O-methyltransferase fibrillarin | Translation |
| Unigene3578_All | -1.169522584 | 4.99636E-11 | RNA-binding protein NOB1 | Translation |
| Unigene4624_All | -1.172953991 | 3.48073E-15 | rRNA biogenesis protein RRP5 | translation |
| Unigene5351_All | -1.233517821 | 1.6687E-75 | RIO kinase 1 | Translation |
| Unigene6420_All | -1.294539928 | 1.59883E-19 | U3 small nucleolar ribonucleoprotein protei | Translation |
| Unigene10651_All | -1.299957266 | 3.16979E-13 | peptidyl-prolyl isomerase F (cyclophilin D) [EC:5.2.1.8] | translation |
| CL406.Contig2_All | -1.306433908 | 2.03198E-07 | DDB1- and CUL4-associated factor 13 | translation |
| Unigene2782_All | -1.360271368 | 4.06761E-13 | U3 small nucleolar RNA-associated protein 24 | Translation |
| CL406.Contig1_All | -1.515591293 | 0.010295603 | DDB1- and CUL4-associated factor 13 | translation |
| CL337.Contig1_All | -1.768501616 | 0.031226294 | small subunit ribosomal protein S10 | Translation |
| Unigene4100_All | -1.87308974 | 3.29369E-07 | regulator of Ty1 transposition protein 103 | translation |
| CL927.Contig2_All | -1.956540398 | 1.79302E-89 | tRNA pseudouridine38-40 synthase | translation |
| Unigene9501_All | -2.267768608 | 2.04314E-40 | la-related protein 1 | translation |
| Unigene9235_All | -2.483988307 | 2.62157E-36 | ubiquitin carboxyl-terminal hydrolase 8 | translation |
| Unigene8814_All | -2.57803438 | 0.000995954 | E3 ubiquitin-protein ligase RNF115/126 | translation |
| CL422.Contig4_All | -3.053043477 | 0 | heat shock 70kDa protein 4 | translation |
| Unigene3460_All | -1.056064102 | 1.20329E-22 | sulfite reductase (NADPH) hemoprotein beta-component | Energy metabolism |
| CL561.Contig1_All | -1.077965698 | 1.09409E-48 | nitronate monooxygenase | Energy metabolism |
| CL730.Contig2_All | -1.080490614 | 5.86952E-45 | coiled-coil-helix-coiled-coil-helix domain-containing protein 2 | Energy metabolism |
| Unigene6020_All | -1.168490504 | 5.59796E-09 | cytochrome c oxidase subunit 1 | Energy metabolism |
| Unigene10558_All | -1.193498089 | 1.22437E-14 | mitochondrial chaperone BCS1 | Energy metabolism |
| Unigene9969_All | -2.976479605 | 0.008497639 | sulfite reductase (NADPH) flavoprotein alpha-component | Energy metabolism |
| Unigene8051_All | -1.000074036 | 8.0002E-10 | hypothetical protein | hypothetical protein |
| CL429.Contig1_All | -1.000323509 | 3.97993E-17 | hypothetical protein | hypothetical protein |
| CL157.Contig3_All | -1.004385269 | 0.001897233 | hypothetical protein | hypothetical protein |
| Unigene9151_All | -1.00887236 | 1.01439E-05 | hypothetical protein | hypothetical protein |
| Unigene9081_All | -1.010463298 | 4.12437E-14 | hypothetical protein | hypothetical protein |
| Unigene8137_All | -1.015365016 | 4.86794E-07 | hypothetical protein | hypothetical protein |
| CL547.Contig2_All | -1.018250606 | 2.03293E-07 | hypothetical protein | hypothetical protein |
| CL14.Contig2_All | -1.018368037 | 3.75461E-36 | hypothetical protein | hypothetical protein |
| Unigene10364_All | -1.022836409 | 1.07397E-05 | hypothetical protein | hypothetical protein |
| Unigene4992_All | -1.023952907 | 1.08977E-09 | hypothetical protein | hypothetical protein |
| Unigene5381_All | -1.024080414 | 0.049817518 | hypothetical protein | hypothetical protein |
| Unigene10396_All | -1.024926169 | 0.023515548 | hypothetical protein | hypothetical protein |
| Unigene10977_All | -1.025160699 | 0.040877808 | hypothetical protein | hypothetical protein |
| Unigene2007_All | -1.025305218 | 4.39386E-15 | hypothetical protein | hypothetical protein |
| Unigene2111_All | -1.030561914 | 0.041741579 | hypothetical protein | hypothetical protein |
| CL118.Contig2_All | -1.046318411 | 8.70157E-26 | hypothetical protein | hypothetical protein |
| Unigene581_All | -1.048780509 | 2.49078E-23 | hypothetical protein | hypothetical protein |
| Unigene10952_All | -1.049103281 | 0.020407776 | hypothetical protein | hypothetical protein |
| Unigene3678_All | -1.051191596 | 2.30556E-16 | hypothetical protein | hypothetical protein |
| Unigene206_All | -1.051359488 | 0.000335785 | hypothetical protein | hypothetical protein |
| Unigene1551_All | -1.053282919 | 0.015232661 | hypothetical protein | hypothetical protein |
| Unigene8208_All | -1.058152206 | 0.005301017 | hypothetical protein | hypothetical protein |
| CL1021.Contig2_All | -1.058548847 | 4.43084E-32 | hypothetical protein | hypothetical protein |
| Unigene7015_All | -1.061297685 | 2.86817E-07 | hypothetical protein | hypothetical protein |
| Unigene5438_All | -1.064137131 | 0.027433935 | hypothetical protein | hypothetical protein |
| Unigene5104_All | -1.069926319 | 0.016415591 | hypothetical protein | hypothetical protein |
| Unigene7948_All | -1.07155992 | 2.00309E-48 | hypothetical protein | hypothetical protein |
| Unigene4946_All | -1.073845348 | 0.002834644 | hypothetical protein | hypothetical protein |
| Unigene2630_All | -1.074122792 | 8.28725E-05 | hypothetical protein | hypothetical protein |
| Unigene9710_All | -1.077031687 | 1.2921E-67 | hypothetical protein | hypothetical protein |
| CL967.Contig1_All | -1.078742251 | 5.30641E-07 | hypothetical protein | hypothetical protein |
| Unigene6388_All | -1.089902417 | 5.10259E-16 | hypothetical protein | hypothetical protein |
| Unigene9574_All | -1.090456886 | 1.78819E-16 | hypothetical protein | hypothetical protein |
| Unigene8311_All | -1.091113893 | 2.99479E-15 | hypothetical protein | hypothetical protein |
| Unigene5674_All | -1.091545174 | 1.02004E-05 | hypothetical protein | hypothetical protein |
| CL921.Contig2_All | -1.095210375 | 0.004147323 | hypothetical protein | hypothetical protein |
| CL651.Contig1_All | -1.095644917 | 4.84147E-26 | hypothetical protein | hypothetical protein |
| CL78.Contig4_All | -1.097017135 | 1.78314E-59 | hypothetical protein | hypothetical protein |
| Unigene10892_All | -1.097427757 | 1.27116E-05 | hypothetical protein | hypothetical protein |
| Unigene2462_All | -1.101209072 | 2.21631E-30 | hypothetical protein | hypothetical protein |
| Unigene3587_All | -1.102599351 | 0.000372374 | hypothetical protein | hypothetical protein |
| Unigene1565_All | -1.103831517 | 4.0718E-05 | hypothetical protein | hypothetical protein |
| CL327.Contig2_All | -1.10597854 | 7.1752E-05 | hypothetical protein | hypothetical protein |
| Unigene6468_All | -1.108932818 | 3.81257E-23 | hypothetical protein | hypothetical protein |
| Unigene2535_All | -1.108993955 | 4.88111E-05 | hypothetical protein | hypothetical protein |
| CL481.Contig1_All | -1.113003122 | 0.000976212 | hypothetical protein | hypothetical protein |
| CL193.Contig1_All | -1.11307647 | 0.040269525 | hypothetical protein | hypothetical protein |
| Unigene964_All | -1.113549328 | 0.036334869 | hypothetical protein | hypothetical protein |
| Unigene6168_All | -1.114670637 | 3.80737E-06 | hypothetical protein | hypothetical protein |
| Unigene5991_All | -1.116123233 | 2.3011E-55 | hypothetical protein | hypothetical protein |
| Unigene9482_All | -1.118299507 | 0.00470853 | hypothetical protein | hypothetical protein |
| Unigene555_All | -1.122921018 | 2.16527E-09 | hypothetical protein | hypothetical protein |
| Unigene7469_All | -1.126748068 | 4.27583E-28 | hypothetical protein | hypothetical protein |
| Unigene7677_All | -1.127141531 | 0.019738282 | hypothetical protein | hypothetical protein |
| Unigene9263_All | -1.128858964 | 0.003690698 | hypothetical protein | hypothetical protein |
| Unigene8057_All | -1.12917715 | 0.003229111 | hypothetical protein | hypothetical protein |
| Unigene1390_All | -1.13484395 | 0.00071905 | hypothetical protein | hypothetical protein |
| Unigene6504_All | -1.139827838 | 0.01064621 | hypothetical protein | hypothetical protein |
| CL75.Contig2_All | -1.142186614 | 1.77418E-72 | hypothetical protein | hypothetical protein |
| Unigene8_All | -1.143804787 | 0.001132982 | hypothetical protein | hypothetical protein |
| CL52.Contig2_All | -1.14396164 | 2.91061E-06 | hypothetical protein | hypothetical protein |
| CL11.Contig2_All | -1.145286422 | 0.001800201 | hypothetical protein | hypothetical protein |
| Unigene7299_All | -1.146065968 | 3.57142E-36 | hypothetical protein | hypothetical protein |
| CL33.Contig3_All | -1.14717031 | 3.76774E-22 | hypothetical protein | hypothetical protein |
| CL967.Contig2_All | -1.149145643 | 1.64961E-68 | hypothetical protein | hypothetical protein |
| Unigene1760_All | -1.159951014 | 7.79837E-09 | hypothetical protein | hypothetical protein |
| Unigene8338_All | -1.163137237 | 2.43961E-21 | hypothetical protein | hypothetical protein |
| CL226.Contig2_All | -1.164763217 | 0.000121054 | hypothetical protein | hypothetical protein |
| Unigene962_All | -1.164917335 | 0.005274122 | hypothetical protein | hypothetical protein |
| CL327.Contig3_All | -1.165320447 | 3.9712E-39 | hypothetical protein | hypothetical protein |
| Unigene1765_All | -1.167147855 | 3.46627E-07 | hypothetical protein | hypothetical protein |
| Unigene636_All | -1.17309875 | 2.38375E-10 | hypothetical protein | hypothetical protein |
| Unigene4877_All | -1.173576214 | 1.02825E-21 | hypothetical protein | hypothetical protein |
| Unigene6125_All | -1.17897914 | 0.012599453 | hypothetical protein | hypothetical protein |
| CL1029.Contig2_All | -1.181097683 | 5.57888E-06 | hypothetical protein | hypothetical protein |
| Unigene10640_All | -1.182236799 | 0.001020994 | hypothetical protein | hypothetical protein |
| Unigene5065_All | -1.182554045 | 1.9843E-07 | hypothetical protein | hypothetical protein |
| Unigene7195_All | -1.18696658 | 1.35731E-05 | hypothetical protein | hypothetical protein |
| Unigene5063_All | -1.193788186 | 1.08847E-05 | hypothetical protein | hypothetical protein |
| Unigene2611_All | -1.196570156 | 0.000529538 | hypothetical protein | hypothetical protein |
| Unigene5477_All | -1.196839375 | 0.043332698 | hypothetical protein | hypothetical protein |
| Unigene1739_All | -1.196913057 | 0.045612226 | hypothetical protein | hypothetical protein |
| Unigene9382_All | -1.198067082 | 4.55406E-31 | hypothetical protein | hypothetical protein |
| Unigene4360_All | -1.205881022 | 0.047467888 | hypothetical protein | hypothetical protein |
| CL787.Contig2_All | -1.206141199 | 0.000735018 | hypothetical protein | hypothetical protein |
| CL327.Contig1_All | -1.207323025 | 4.18866E-07 | hypothetical protein | hypothetical protein |
| CL193.Contig3_All | -1.209312378 | 1.15015E-56 | hypothetical protein | hypothetical protein |
| CL820.Contig2_All | -1.209350098 | 0.014009482 | hypothetical protein | hypothetical protein |
| Unigene1812_All | -1.210035104 | 4.73584E-06 | hypothetical protein | hypothetical protein |
| Unigene8733_All | -1.214733893 | 0.001200422 | hypothetical protein | hypothetical protein |
| Unigene3708_All | -1.216139154 | 5.79357E-21 | hypothetical protein | hypothetical protein |
| Unigene3945_All | -1.219584792 | 0.03291283 | hypothetical protein | hypothetical protein |
| CL757.Contig2_All | -1.22328587 | 6.64005E-85 | hypothetical protein | hypothetical protein |
| CL74.Contig11_All | -1.2279027 | 2.44767E-29 | hypothetical protein | hypothetical protein |
| Unigene6495_All | -1.231044761 | 0.000653337 | hypothetical protein | hypothetical protein |
| Unigene7649_All | -1.234271508 | 0.009522194 | hypothetical protein | hypothetical protein |
| Unigene966_All | -1.237232697 | 0.034462109 | hypothetical protein | hypothetical protein |
| Unigene1684_All | -1.237339366 | 1.04584E-05 | hypothetical protein | hypothetical protein |
| Unigene10557_All | -1.238295842 | 4.66255E-14 | hypothetical protein | hypothetical protein |
| Unigene9880_All | -1.239953585 | 0.009102518 | hypothetical protein | hypothetical protein |
| CL589.Contig2_All | -1.246212704 | 0.009033358 | hypothetical protein | hypothetical protein |
| Unigene4686_All | -1.25079299 | 7.4242E-06 | hypothetical protein | hypothetical protein |
| Unigene4972_All | -1.251016082 | 0.006471119 | hypothetical protein | hypothetical protein |
| CL78.Contig9_All | -1.251317473 | 1.22534E-10 | hypothetical protein | hypothetical protein |
| Unigene2556_All | -1.251777368 | 4.68613E-09 | hypothetical protein | hypothetical protein |
| Unigene8746_All | -1.254306441 | 1.38395E-40 | hypothetical protein | hypothetical protein |
| Unigene10430_All | -1.254708737 | 1.16606E-07 | hypothetical protein | hypothetical protein |
| Unigene3813_All | -1.264218925 | 0.001297908 | hypothetical protein | hypothetical protein |
| Unigene7678_All | -1.264381053 | 0.016609961 | hypothetical protein | hypothetical protein |
| Unigene5955_All | -1.271086732 | 6.89133E-09 | hypothetical protein | hypothetical protein |
| Unigene3888_All | -1.272275823 | 2.27931E-06 | hypothetical protein | hypothetical protein |
| Unigene8149_All | -1.27561394 | 5.52393E-07 | hypothetical protein | hypothetical protein |
| Unigene461_All | -1.276410076 | 0.000506165 | hypothetical protein | hypothetical protein |
| Unigene7741_All | -1.277631667 | 0.049693652 | hypothetical protein | hypothetical protein |
| Unigene10075_All | -1.279813692 | 1.83221E-72 | hypothetical protein | hypothetical protein |
| Unigene6105_All | -1.279936202 | 1.03628E-16 | hypothetical protein | hypothetical protein |
| Unigene5810_All | -1.282190848 | 9.10218E-11 | hypothetical protein | hypothetical protein |
| Unigene8827_All | -1.289059797 | 0.019139272 | hypothetical protein | hypothetical protein |
| CL272.Contig2_All | -1.290425793 | 0.024943965 | hypothetical protein | hypothetical protein |
| CL118.Contig1_All | -1.29107114 | 1.52659E-11 | hypothetical protein | hypothetical protein |
| Unigene575_All | -1.29544695 | 7.41819E-06 | hypothetical protein | hypothetical protein |
| CL41.Contig2_All | -1.298499849 | 1.2341E-118 | hypothetical protein | hypothetical protein |
| CL310.Contig4_All | -1.298619911 | 8.39829E-67 | hypothetical protein | hypothetical protein |
| CL921.Contig1_All | -1.298681252 | 0.00119666 | hypothetical protein | hypothetical protein |
| Unigene8599_All | -1.312691224 | 7.24218E-06 | hypothetical protein | hypothetical protein |
| CL907.Contig1_All | -1.318730828 | 1.20196E-08 | hypothetical protein | hypothetical protein |
| Unigene934_All | -1.320997109 | 0.002988218 | hypothetical protein | hypothetical protein |
| Unigene7170_All | -1.323444739 | 0.027384344 | hypothetical protein | hypothetical protein |
| Unigene5320_All | -1.324055215 | 3.69898E-16 | hypothetical protein | hypothetical protein |
| CL193.Contig2_All | -1.330091651 | 8.72066E-05 | hypothetical protein | hypothetical protein |
| Unigene12556_All | -1.337184229 | 0.000376107 | hypothetical protein | hypothetical protein |
| Unigene6838_All | -1.33885683 | 2.44404E-75 | hypothetical protein | hypothetical protein |
| Unigene7216_All | -1.346771838 | 0.002784462 | hypothetical protein | hypothetical protein |
| CL108.Contig2_All | -1.346876365 | 3.61957E-25 | hypothetical protein | hypothetical protein |
| CL1016.Contig1_All | -1.353776413 | 1.48362E-07 | hypothetical protein | hypothetical protein |
| Unigene7612_All | -1.357545185 | 7.69164E-05 | hypothetical protein | hypothetical protein |
| Unigene1292_All | -1.360205916 | 2.50342E-09 | hypothetical protein | hypothetical protein |
| Unigene10980_All | -1.369272178 | 0.001874919 | hypothetical protein | hypothetical protein |
| CL74.Contig12_All | -1.373477188 | 4.56293E-46 | hypothetical protein | hypothetical protein |
| Unigene11097_All | -1.375629334 | 0.014945204 | hypothetical protein | hypothetical protein |
| Unigene7202_All | -1.384432608 | 5.44858E-23 | hypothetical protein | hypothetical protein |
| CL24.Contig3_All | -1.386337441 | 6.86926E-11 | hypothetical protein | hypothetical protein |
| Unigene3937_All | -1.386853253 | 3.8997E-10 | hypothetical protein | hypothetical protein |
| Unigene7674_All | -1.387143772 | 0.000377431 | hypothetical protein | hypothetical protein |
| Unigene4562_All | -1.388253384 | 5.86213E-25 | hypothetical protein | hypothetical protein |
| Unigene5390_All | -1.391583295 | 0.002385655 | hypothetical protein | hypothetical protein |
| CL842.Contig1_All | -1.39226364 | 0.00015421 | hypothetical protein | hypothetical protein |
| Unigene8451_All | -1.396307337 | 3.92835E-52 | hypothetical protein | hypothetical protein |
| Unigene10497_All | -1.40216661 | 1.11153E-06 | hypothetical protein | hypothetical protein |
| Unigene4272_All | -1.406900077 | 0.037404418 | hypothetical protein | hypothetical protein |
| Unigene2706_All | -1.409336408 | 1.97493E-18 | hypothetical protein | hypothetical protein |
| Unigene5728_All | -1.413197077 | 5.4792E-18 | hypothetical protein | hypothetical protein |
| Unigene4804_All | -1.419422851 | 9.38961E-10 | hypothetical protein | hypothetical protein |
| Unigene8352_All | -1.420391545 | 0.008418766 | hypothetical protein | hypothetical protein |
| CL74.Contig2_All | -1.421851458 | 4.66007E-18 | hypothetical protein | hypothetical protein |
| CL138.Contig1_All | -1.425479955 | 4.17892E-29 | hypothetical protein | hypothetical protein |
| Unigene9370_All | -1.427251712 | 3.01158E-05 | hypothetical protein | hypothetical protein |
| CL226.Contig3_All | -1.443867734 | 6.39162E-15 | hypothetical protein | hypothetical protein |
| Unigene7024_All | -1.448879568 | 4.06024E-08 | hypothetical protein | hypothetical protein |
| CL92.Contig2_All | -1.451739545 | 1.38084E-21 | hypothetical protein | hypothetical protein |
| Unigene5941_All | -1.4597155 | 2.13902E-73 | hypothetical protein | hypothetical protein |
| CL74.Contig4_All | -1.468663399 | 3.4006E-105 | hypothetical protein | hypothetical protein |
| Unigene4643_All | -1.472177598 | 2.36108E-41 | hypothetical protein | hypothetical protein |
| Unigene3354_All | -1.476361983 | 2.77976E-16 | hypothetical protein | hypothetical protein |
| Unigene8787_All | -1.477212763 | 0.017563631 | hypothetical protein | hypothetical protein |
| Unigene10988_All | -1.478023263 | 0.000707506 | hypothetical protein | hypothetical protein |
| CL481.Contig3_All | -1.479884219 | 1.52563E-42 | hypothetical protein | hypothetical protein |
| CL600.Contig4_All | -1.480481727 | 1.96159E-05 | hypothetical protein | hypothetical protein |
| CL113.Contig1_All | -1.481330329 | 3.9716E-80 | hypothetical protein | hypothetical protein |
| Unigene4849_All | -1.489305804 | 1.80097E-26 | hypothetical protein | hypothetical protein |
| Unigene4136_All | -1.491523704 | 3.6902E-23 | hypothetical protein | hypothetical protein |
| Unigene8529_All | -1.492437907 | 7.33706E-07 | hypothetical protein | hypothetical protein |
| Unigene5318_All | -1.495157733 | 6.68162E-31 | hypothetical protein | hypothetical protein |
| Unigene6533_All | -1.495284279 | 3.40214E-06 | hypothetical protein | hypothetical protein |
| Unigene2107_All | -1.498071995 | 0.000647673 | hypothetical protein | hypothetical protein |
| Unigene5301_All | -1.501334787 | 3.99394E-32 | hypothetical protein | hypothetical protein |
| Unigene5037_All | -1.508255924 | 2.79454E-10 | hypothetical protein | hypothetical protein |
| CL33.Contig2_All | -1.513096874 | 2.45549E-31 | hypothetical protein | hypothetical protein |
| CL52.Contig1_All | -1.515501522 | 1.35961E-11 | hypothetical protein | hypothetical protein |
| Unigene3686_All | -1.516007875 | 1.3158E-06 | hypothetical protein | hypothetical protein |
| CL181.Contig2_All | -1.516081439 | 7.39604E-73 | hypothetical protein | hypothetical protein |
| CL441.Contig1_All | -1.516630481 | 4.93203E-13 | hypothetical protein | hypothetical protein |
| Unigene103_All | -1.520027484 | 1.19849E-29 | hypothetical protein | hypothetical protein |
| CL787.Contig1_All | -1.523788588 | 4.59116E-18 | hypothetical protein | hypothetical protein |
| Unigene4484_All | -1.530673663 | 8.141E-11 | hypothetical protein | hypothetical protein |
| CL24.Contig1_All | -1.539304321 | 1.92486E-23 | hypothetical protein | hypothetical protein |
| Unigene8942_All | -1.547341919 | 4.044E-27 | hypothetical protein | hypothetical protein |
| CL331.Contig2_All | -1.547738737 | 1.37577E-24 | hypothetical protein | hypothetical protein |
| Unigene10512_All | -1.550786017 | 1.48435E-07 | hypothetical protein | hypothetical protein |
| Unigene8702_All | -1.550871381 | 0.000448984 | hypothetical protein | hypothetical protein |
| Unigene2594_All | -1.556486376 | 1.03578E-10 | hypothetical protein | hypothetical protein |
| Unigene309_All | -1.557142487 | 1.06263E-09 | hypothetical protein | hypothetical protein |
| Unigene3154_All | -1.557416946 | 0.024186317 | hypothetical protein | hypothetical protein |
| CL907.Contig2_All | -1.560303145 | 5.5096E-158 | hypothetical protein | hypothetical protein |
| Unigene670_All | -1.571442636 | 2.80046E-19 | hypothetical protein | hypothetical protein |
| Unigene11020_All | -1.574635266 | 0.049912336 | hypothetical protein | hypothetical protein |
| Unigene4976_All | -1.584096702 | 0.000829228 | hypothetical protein | hypothetical protein |
| Unigene1043_All | -1.58571671 | 0.017368551 | hypothetical protein | hypothetical protein |
| Unigene9246_All | -1.591896581 | 1.08279E-28 | hypothetical protein | hypothetical protein |
| CL310.Contig3_All | -1.592179497 | 8.00305E-44 | hypothetical protein | hypothetical protein |
| Unigene1476_All | -1.592258022 | 0.000166238 | hypothetical protein | hypothetical protein |
| CL74.Contig10_All | -1.593388316 | 2.6721E-158 | hypothetical protein | hypothetical protein |
| Unigene3179_All | -1.600186167 | 0.009364612 | hypothetical protein | hypothetical protein |
| Unigene8270_All | -1.600720591 | 0.000104678 | hypothetical protein | hypothetical protein |
| Unigene10692_All | -1.608470107 | 1.13658E-06 | hypothetical protein | hypothetical protein |
| Unigene4253_All | -1.617026388 | 0.030863928 | hypothetical protein | hypothetical protein |
| CL518.Contig1_All | -1.626230226 | 1.94863E-92 | hypothetical protein | hypothetical protein |
| CL526.Contig2_All | -1.626893594 | 5.51809E-06 | hypothetical protein | hypothetical protein |
| CL784.Contig1_All | -1.629514076 | 4.75994E-38 | hypothetical protein | hypothetical protein |
| Unigene914_All | -1.639388056 | 1.37979E-10 | hypothetical protein | hypothetical protein |
| Unigene2164_All | -1.644389649 | 0.017138307 | hypothetical protein | hypothetical protein |
| Unigene2559_All | -1.647674554 | 5.40368E-12 | hypothetical protein | hypothetical protein |
| Unigene1795_All | -1.658704974 | 8.02175E-97 | hypothetical protein | hypothetical protein |
| Unigene10773_All | -1.659033482 | 2.24886E-91 | hypothetical protein | hypothetical protein |
| CL92.Contig3_All | -1.696376852 | 8.36615E-85 | hypothetical protein | hypothetical protein |
| CL960.Contig1_All | -1.701497529 | 1.31594E-07 | hypothetical protein | hypothetical protein |
| CL558.Contig2_All | -1.70385382 | 8.5297E-40 | hypothetical protein | hypothetical protein |
| Unigene9066_All | -1.711016453 | 8.90818E-43 | hypothetical protein | hypothetical protein |
| Unigene5943_All | -1.716607725 | 2.7216E-10 | hypothetical protein | hypothetical protein |
| Unigene10604_All | -1.71993839 | 8.52771E-12 | hypothetical protein | hypothetical protein |
| Unigene9234_All | -1.74328856 | 1.14941E-58 | hypothetical protein | hypothetical protein |
| CL24.Contig6_All | -1.750482347 | 0.023318796 | hypothetical protein | hypothetical protein |
| Unigene7952_All | -1.751643609 | 5.80545E-19 | hypothetical protein | hypothetical protein |
| Unigene5319_All | -1.75499738 | 1.73682E-62 | hypothetical protein | hypothetical protein |
| CL833.Contig1_All | -1.768952934 | 4.5679E-118 | hypothetical protein | hypothetical protein |
| Unigene4326_All | -1.78295761 | 0.000106909 | hypothetical protein | hypothetical protein |
| Unigene1552_All | -1.785563179 | 7.69383E-26 | hypothetical protein | hypothetical protein |
| Unigene7732_All | -1.794770719 | 0.012414587 | hypothetical protein | hypothetical protein |
| CL237.Contig1_All | -1.797239949 | 0.005890165 | hypothetical protein | hypothetical protein |
| Unigene2606_All | -1.797677112 | 9.00676E-46 | hypothetical protein | hypothetical protein |
| CL74.Contig6_All | -1.799124686 | 1.1452E-86 | hypothetical protein | hypothetical protein |
| Unigene9416_All | -1.804886737 | 1.41025E-05 | hypothetical protein | hypothetical protein |
| Unigene6574_All | -1.814651941 | 0.001844964 | hypothetical protein | hypothetical protein |
| Unigene5371_All | -1.819570045 | 0.017283663 | hypothetical protein | hypothetical protein |
| Unigene6395_All | -1.825621843 | 2.61308E-20 | hypothetical protein | hypothetical protein |
| Unigene803_All | -1.829653395 | 2.1553E-109 | hypothetical protein | hypothetical protein |
| Unigene6193_All | -1.837987438 | 2.0215E-19 | hypothetical protein | hypothetical protein |
| Unigene2136_All | -1.857083121 | 0.037012968 | hypothetical protein | hypothetical protein |
| Unigene3119_All | -1.863523467 | 0.004408284 | hypothetical protein | hypothetical protein |
| CL318.Contig1_All | -1.867705279 | 0.004723923 | hypothetical protein | hypothetical protein |
| Unigene6042_All | -1.872234763 | 2.79842E-12 | hypothetical protein | hypothetical protein |
| CL64.Contig4_All | -1.88259412 | 1.42118E-35 | hypothetical protein | hypothetical protein |
| Unigene2589_All | -1.886751659 | 3.85232E-29 | hypothetical protein | hypothetical protein |
| Unigene2640_All | -1.901667969 | 9.9468E-11 | hypothetical protein | hypothetical protein |
| Unigene2219_All | -1.916336234 | 0.043289548 | hypothetical protein | hypothetical protein |
| Unigene7705_All | -1.918945722 | 0.027498107 | hypothetical protein | hypothetical protein |
| CL269.Contig4_All | -1.927603622 | 0.041941159 | hypothetical protein | hypothetical protein |
| CL289.Contig9_All | -1.930430582 | 4.73732E-11 | hypothetical protein | hypothetical protein |
| CL33.Contig1_All | -1.932623423 | 1.34319E-07 | hypothetical protein | hypothetical protein |
| Unigene2679_All | -1.935888138 | 8.38744E-24 | hypothetical protein | hypothetical protein |
| Unigene2609_All | -1.945129044 | 4.69481E-78 | hypothetical protein | hypothetical protein |
| CL897.Contig1_All | -1.956922918 | 0.000130229 | hypothetical protein | hypothetical protein |
| Unigene2668_All | -1.98360737 | 3.87448E-47 | hypothetical protein | hypothetical protein |
| Unigene8422_All | -1.994220338 | 3.64244E-72 | hypothetical protein | hypothetical protein |
| Unigene3818_All | -2.003776102 | 3.88563E-14 | hypothetical protein | hypothetical protein |
| Unigene9966_All | -2.01242312 | 0.006660598 | hypothetical protein | hypothetical protein |
| Unigene9741_All | -2.015582956 | 5.06479E-66 | hypothetical protein | hypothetical protein |
| Unigene8756_All | -2.038910977 | 0.000449126 | hypothetical protein | hypothetical protein |
| Unigene5008_All | -2.043500604 | 3.663E-270 | hypothetical protein | hypothetical protein |
| Unigene7735_All | -2.043930009 | 0.014954843 | hypothetical protein | hypothetical protein |
| Unigene9914_All | -2.048058925 | 4.77952E-05 | hypothetical protein | hypothetical protein |
| Unigene2800_All | -2.078768807 | 3.83908E-12 | hypothetical protein | hypothetical protein |
| Unigene4709_All | -2.084189053 | 1.35997E-28 | hypothetical protein | hypothetical protein |
| CL434.Contig2_All | -2.087325425 | 3.91668E-05 | hypothetical protein | hypothetical protein |
| CL203.Contig2_All | -2.093392808 | 0.00193692 | hypothetical protein | hypothetical protein |
| CL482.Contig1_All | -2.094731469 | 1.40587E-08 | hypothetical protein | hypothetical protein |
| Unigene644_All | -2.098500532 | 1.46946E-31 | hypothetical protein | hypothetical protein |
| Unigene3144_All | -2.114618577 | 9.74031E-05 | hypothetical protein | hypothetical protein |
| CL64.Contig1_All | -2.122663762 | 4.39687E-97 | hypothetical protein | hypothetical protein |
| Unigene5481_All | -2.128643919 | 1.24447E-07 | hypothetical protein | hypothetical protein |
| Unigene2141_All | -2.138491883 | 1.12605E-05 | hypothetical protein | hypothetical protein |
| Unigene7726_All | -2.154548642 | 0.00336474 | hypothetical protein | hypothetical protein |
| Unigene8822_All | -2.185759326 | 0.006242262 | hypothetical protein | hypothetical protein |
| Unigene10308_All | -2.202571508 | 1.58804E-30 | hypothetical protein | hypothetical protein |
| Unigene11054_All | -2.216297002 | 3.17637E-07 | hypothetical protein | hypothetical protein |
| Unigene4074_All | -2.237426652 | 2.14361E-16 | hypothetical protein | hypothetical protein |
| Unigene8870_All | -2.240902422 | 0.024695924 | hypothetical protein | hypothetical protein |
| CL331.Contig3_All | -2.247890133 | 2.15578E-06 | hypothetical protein | hypothetical protein |
| Unigene7699_All | -2.249607765 | 1.11459E-06 | hypothetical protein | hypothetical protein |
| Unigene8717_All | -2.281780406 | 5.36903E-13 | hypothetical protein | hypothetical protein |
| CL498.Contig1_All | -2.28501649 | 9.80818E-33 | hypothetical protein | hypothetical protein |
| Unigene6611_All | -2.308806054 | 0.002834644 | hypothetical protein | hypothetical protein |
| CL441.Contig4_All | -2.32819596 | 0.000176752 | hypothetical protein | hypothetical protein |
| Unigene8828_All | -2.3499167 | 0.047293026 | hypothetical protein | hypothetical protein |
| Unigene9369_All | -2.356721845 | 3.82006E-24 | hypothetical protein | hypothetical protein |
| Unigene9128_All | -2.360474676 | 1.06574E-26 | hypothetical protein | hypothetical protein |
| Unigene7745_All | -2.362643416 | 0.007338422 | hypothetical protein | hypothetical protein |
| Unigene5395_All | -2.362850153 | 0.008288696 | hypothetical protein | hypothetical protein |
| Unigene3153_All | -2.387598925 | 7.33049E-12 | hypothetical protein | hypothetical protein |
| Unigene4354_All | -2.391515199 | 2.97676E-07 | hypothetical protein | hypothetical protein |
| Unigene4898_All | -2.393219542 | 2.3665E-205 | hypothetical protein | hypothetical protein |
| Unigene5441_All | -2.397569156 | 0.000363398 | hypothetical protein | hypothetical protein |
| Unigene5527_All | -2.418119969 | 0.007571075 | hypothetical protein | hypothetical protein |
| Unigene953_All | -2.428341174 | 0.000295351 | hypothetical protein | hypothetical protein |
| Unigene5476_All | -2.443708936 | 0.003759026 | hypothetical protein | hypothetical protein |
| Unigene7722_All | -2.449641063 | 0.001103482 | hypothetical protein | hypothetical protein |
| Unigene2203_All | -2.469271803 | 0.000342864 | hypothetical protein | hypothetical protein |
| Unigene11068_All | -2.481098021 | 0.005672933 | hypothetical protein | hypothetical protein |
| Unigene3267_All | -2.482877748 | 0.023650337 | hypothetical protein | hypothetical protein |
| Unigene5475_All | -2.505473831 | 0.002572944 | hypothetical protein | hypothetical protein |
| Unigene8825_All | -2.548648764 | 3.68973E-07 | hypothetical protein | hypothetical protein |
| Unigene11142_All | -2.565229938 | 0.011223372 | hypothetical protein | hypothetical protein |
| CL784.Contig2_All | -2.570372354 | 7.03736E-40 | hypothetical protein | hypothetical protein |
| Unigene6614_All | -2.580499829 | 3.50383E-07 | hypothetical protein | hypothetical protein |
| CL878.Contig1_All | -2.587618583 | 0.007354656 | hypothetical protein | hypothetical protein |
| CL74.Contig9_All | -2.593073875 | 0.019484973 | hypothetical protein | hypothetical protein |
| Unigene10520_All | -2.609827211 | 2.03663E-55 | hypothetical protein | hypothetical protein |
| Unigene1575_All | -2.6115162 | 7.67264E-96 | hypothetical protein | hypothetical protein |
| Unigene11154_All | -2.612209703 | 0.032054906 | hypothetical protein | hypothetical protein |
| CL811.Contig2_All | -2.625108937 | 2.58781E-06 | hypothetical protein | hypothetical protein |
| Unigene12694_All | -2.709222596 | 0.023715237 | hypothetical protein | hypothetical protein |
| Unigene7664_All | -2.729277977 | 2.08733E-05 | hypothetical protein | hypothetical protein |
| CL518.Contig2_All | -2.780277602 | 1.34969E-05 | hypothetical protein | hypothetical protein |
| Unigene8757_All | -2.802648364 | 3.09964E-10 | hypothetical protein | hypothetical protein |
| Unigene4358_All | -2.828386118 | 0.000267843 | hypothetical protein | hypothetical protein |
| CL498.Contig2_All | -2.834360326 | 0.000191224 | hypothetical protein | hypothetical protein |
| Unigene2162_All | -2.853380624 | 0.000292807 | hypothetical protein | hypothetical protein |
| Unigene7767_All | -2.855419792 | 0.001948281 | hypothetical protein | hypothetical protein |
| CL441.Contig3_All | -2.885590791 | 1.62915E-35 | hypothetical protein | hypothetical protein |
| Unigene11049_All | -2.945820885 | 3.84221E-07 | hypothetical protein | hypothetical protein |
| Unigene7165_All | -2.974128064 | 2.75193E-42 | hypothetical protein | hypothetical protein |
| Unigene9926_All | -3.005979338 | 4.97388E-08 | hypothetical protein | hypothetical protein |
| Unigene2209_All | -3.00653641 | 1.38234E-06 | hypothetical protein | hypothetical protein |
| Unigene5444_All | -3.03386692 | 0.015119623 | hypothetical protein | hypothetical protein |
| Unigene9970_All | -3.051168761 | 0.005326988 | hypothetical protein | hypothetical protein |
| Unigene9513_All | -3.113262016 | 3.982E-191 | hypothetical protein | hypothetical protein |
| CL441.Contig2_All | -3.129685543 | 1.38942E-38 | hypothetical protein | hypothetical protein |
| Unigene7273_All | -3.160664343 | 7.93932E-45 | hypothetical protein | hypothetical protein |
| Unigene3160_All | -3.169865079 | 5.89874E-05 | hypothetical protein | hypothetical protein |
| Unigene2180_All | -3.289464935 | 0.008183942 | hypothetical protein | hypothetical protein |
| Unigene987_All | -3.375936672 | 1.23719E-05 | hypothetical protein | hypothetical protein |
| Unigene2198_All | -3.42355143 | 0.000684489 | hypothetical protein | hypothetical protein |
| Unigene8278_All | -3.631360362 | 2.7074E-104 | hypothetical protein | hypothetical protein |
| Unigene1057_All | -3.795868642 | 1.02345E-06 | hypothetical protein | hypothetical protein |
| Unigene12879_All | -4.111731806 | 0.040456952 | hypothetical protein | hypothetical protein |
| Unigene9903_All | -4.118779286 | 1.40576E-10 | hypothetical protein | hypothetical protein |
| Unigene12339_All | -4.546473839 | 0.010318903 | hypothetical protein | hypothetical protein |
| CL366.Contig1_All | -4.569417824 | 0.034279218 | hypothetical protein | hypothetical protein |
| Unigene12699_All | -4.570785449 | 0.037753742 | hypothetical protein | hypothetical protein |
| Unigene12766_All | -4.575202606 | 0.040877808 | hypothetical protein | hypothetical protein |
| Unigene9964_All | -4.674514965 | 1.58828E-13 | hypothetical protein | hypothetical protein |
| Unigene1004_All | -4.769938524 | 0.000407135 | hypothetical protein | hypothetical protein |
| Unigene8835_All | -4.911007327 | 0.000259656 | hypothetical protein | hypothetical protein |
| Unigene4331_All | -4.930196692 | 0.002786212 | hypothetical protein | hypothetical protein |
| Unigene7756_All | -5.15326647 | 0.007447869 | hypothetical protein | hypothetical protein |
| Unigene2249_All | -6.271613739 | 4.49246E-05 | hypothetical protein | hypothetical protein |
| Unigene7725_All | -6.509740336 | 1.25455E-05 | hypothetical protein | hypothetical protein |
| Unigene4369_All | -6.538758894 | 1.09931E-05 | hypothetical protein | hypothetical protein |
| CL175.Contig2_All | -6.678870981 | 4.13515E-89 | hypothetical protein | hypothetical protein |
| Unigene7775_All | -7.197674138 | 4.61523E-07 | hypothetical protein | hypothetical protein |
| CL175.Contig1_All | -7.807362348 | 2.34212E-71 | hypothetical protein | hypothetical protein |
| CL74.Contig7_All | -8.29754611 | 2.68135E-10 | hypothetical protein | hypothetical protein |

**Supplementary Table S8 Metabolomic data comparing *Schizochytrium* ZW1 and ZW2**

|  | ZW1 | ZW1 | ZW1 | ZW2 | ZW2 | ZW2 |
| --- | --- | --- | --- | --- | --- | --- |
| NADPH | 63 | 94 | 95 | 89 | 100 | 53 |
| NADP | 46 | 19 | 30 | 207 | 201 | 174 |
| NADH | 38 | 39 | 41 | 40 | 45 | 26 |
| NAD | 324 | 135 | 230 | 506 | 279 | 265 |
| ATP | 201 | 120 | 151 | 584 | 526 | 513 |
| ADP | 215 | 126 | 159 | 595 | 530 | 514 |
| CoA | 84 | 86 | 98 | 109 | 88 | 71 |
| AMP | 1187 | 626 | 770 | 2879 | 3767 | 3168 |
| F6P | 447 | 269 | 336 | 1738 | 843 | 788 |
| R5P | 119 | 73 | 82 | 411 | 324 | 300 |
| E4P | 180 | 128 | 144 | 456 | 420 | 440 |
| CIT | 1577 | 1087 | 1223 | 1947 | 1188 | 1339 |
| 3-PG | 729 | 523 | 664 | 247 | 300 | 382 |
| 1,3-PG | 346 | 246 | 287 | 205 | 227 | 249 |
| PEP | 105 | 77 | 115 | 211 | 173 | 180 |
| GAP | 33 | 27 | 35 | 159 | 148 | 258 |
| DHAP | 68 | 43 | 48 | 219 | 201 | 198 |
| Glu | 623 | 463 | 466 | 4238 | 4574 | 4418 |
| AKG | 83 | 65 | 72 | 190 | 213 | 227 |
| MAL | 1898 | 1037 | 1242 | 1230 | 536 | 737 |
| OXA | 729 | 602 | 678 | 1905 | 1205 | 1205 |
| SUC | 1281 | 738 | 979 | 2428 | 1285 | 1278 |
| FUM | 1419 | 835 | 995 | 676 | 487 | 606 |
| Ac-CoA | 2156 | 2422 | 2205 | 3994 | 4280 | 4543 |

**Supplementary Table S9 Metabolomic data comparing *Schizochytrium* ZW1 with ZW1 G1G2-1 and ZW1 G1G2-2**

|  | ZW1 | ZW1 | ZW1 | ZW1 G1G2-1 | ZW1 G1G2-1 | ZW1 G1G2-1 | ZW1 G1G2-2 | ZW1 G1G2-2 | ZW1 G1G2-2 |
| --- | --- | --- | --- | --- | --- | --- | --- | --- | --- |
| NADPH | 1370 | 1221 | 1329 | 1673 | 1930 | 1758 | 2043 | 1862 | 1952 |
| NADP | 2849 | 2534 | 3235 | 3354 | 5286 | 2772 | 2609 | 2073 | 2706 |
| NADH | 477 | 525 | 573 | 560 | 474 | 421 | 782 | 621 | 550 |
| NAD | 10576 | 10061 | 11577 | 13793 | 12052 | 10412 | 7338 | 7569 | 7700 |
| ATP | 4678 | 2039 | 4741 | 3188 | 4898 | 3031 | 5449 | 3831 | 3970 |
| ADP | 4690 | 2284 | 4926 | 3492 | 5109 | 3209 | 4734 | 3511 | 3729 |
| Coa | 753 | 787 | 881 | 1117 | 1188 | 1329 | 1336 | 1291 | 1299 |
| AMP | 48527 | 38653 | 48778 | 71638 | 450666 | 56347 | 38257 | 46093 | 84020 |
| F6P | 6652 | 5466 | 7506 | 15445 | 16597 | 13385 | 10025 | 10611 | 11163 |
| R5P | 1849 | 1250 | 2165 | 3293 | 3895 | 2064 | 1897 | 1218 | 3328 |
| E4P | 2369 | 1932 | 2916 | 4322 | 5124 | 3926 | 4804 | 5113 | 5854 |
| Citric | 23968 | 11517 | 16882 | 14201 | 16011 | 18270 | 17899 | 24529 | 22701 |
| 3-PG | 3186 | 1372 | 2958 | 2353 | 3871 | 2049 | 3629 | 2996 | 6745 |
| 23-PG | 1812 | 1256 | 2418 | 2125 | 3256 | 1948 | 3049 | 2657 | 4643 |
| PEP | 626 | 652 | 673 | 734 | 706 | 677 | 1129 | 866 | 934 |
| GAP | 648 | 664 | 694 | 1114 | 856 | 927 | 3018 | 1510 | 2687 |
| DHAP | 1239 | 530 | 1308 | 1694 | 1899 | 1416 | 1888 | 1880 | 2426 |
| Glu | 5671 | 5656 | 11468 | 18823 | 24035 | 26537 | 54810 | 83685 | 76430 |
| AKG | 4505 | 4910 | 3920 | 3126 | 3102 | 4679 | 16827 | 12497 | 13747 |
| Malate | 30253 | 27172 | 31852 | 18943 | 33610 | 20548 | 57830 | 38485 | 41153 |
| Oxa | 3005 | 2546 | 3023 | 2345 | 2444 | 3073 | 4070 | 5542 | 3885 |
| SUC | 37769 | 42924 | 37917 | 37421 | 36776 | 42302 | 100971 | 89445 | 82074 |
| FUM | 16468 | 13849 | 19349 | 12258 | 16800 | 15426 | 28137 | 26461 | 26393 |
| acety-coA | 1881 | 1776 | 1860 | 2273 | 2016 | 2473 | 5963 | 4244 | 3542 |
| G3P (mmol/g protein） | 0.0859 | 0.0872 | 0.0746 | 0.1237 | 0.1270 | 0.1143 | 0.1054 | 0.1094 | 0.1089 |

**Supplementary Note 1 Locations of mutation sites in genes related to lipid metabolism identified through genome resequencing.**

Acyl-CoA dehydrogenase family member 11

Hypothesized Function: Catalyzes the α, β-dehydrogenation of acyl-CoA to generate 2,3-dehydroacyl-CoA, representing the initial step in each cycle of fatty acid β-oxidation.

>ZW1-scaffold307.t7 EVMscaffold307.g7 scaffold307:22085-23920(-)

MERRVQDFQPGPRLGNTWREDVSLQRFAKAFLGESNDWDEVDQDLASFGAKCGAEYLKMADNAERFPPQLEQFNQWGQRVDRLHLAEGWRYFKRESAIEGLVSIPYEKRTPEARIHQVLKLMLFNPSGGMFGCPLAMTDGAALAIGGILAEQDALDARSSTRKLEEKTIAELRHAYKCLTTRDPDSFWTSGQWMTEKIGGSDLSRATQTFALERPSTTSTHALYGYKWFTSAVDSEMTMTLASLSDEASPDLDRERKPLSLFFLRTHKDPNHLSPEDAALAGSGDLPLNNIKVQRLKDKMATRQLPTAELILEGAHAHLLSKEGSGIKAVSPMLTVTRLHTALSSVGYCIRMRNLAIDFAHTRKAFGKAIVDKPLHLQSLYEYDMDVRGNTLFVLEVARLLGLLETGKASAHEDALLRATTSLVKVFCSRELVRLMSEGVELFGAVGVMNNHMSTIFRDASVMPIWEGTSNTLSLDLLRVAAREPEALEAMFKELRSMAPSSDAGGALRAEVDKVFGYIKANAANLEVFEWNARRLTMNLSRLYICALAARTAAKTGHKEDFAMYEYWLDRVTREYEPMTDPAVHDTYRSTLSSKMNGAMRDEDGNIRSVL

>ZW2-scaffold307.t7 EVMscaffold307.g7 scaffold307:22085-23920(-)

MERRVQDFQPGPRLGNTWREDVSLQRFAKAFLGESNDWDEVDQDLASFGAKCGAEYLKMADNAERFPPQLEQFNQWGQRVDRLHLAEGWRYFKRESAIEGLVSIPYEKRTPEARIHQVLKLMLFNPSGGMFGCPLAMTDGAALAIGGILAEQDALDARSSTRKLEEKTIAELRHAYKCLTTRDPDSFWTSGQWMTEKIGGSDLSRATQTFALERPSTTSTHALYGYKWFTSAVDSEMTMTLASLSDEASPDLDRERKPLSLFFLRTHKDPNHLSPEDAALAGSGDLPLNNIKVQRLKDKMATRQLPTAELILEGAHAHLLSKEGSGIKAVSPMLTVTRLHTALSSVGYCIRMRNLAIDFAHTRKAFGKAIVDKPLHLQSLYEYDMDVRGNTLFVLEVARLLGLLETGKASAHEDALLRATTSLVKVFCSRELVRLMSEGVELFGAVGVMNNHMSTIFRDASVMPIWEGTSNTLSLDLLRVAAREPEALEAMFKELRSMAPSSDAGDALRAEVDKVFGYIKANAANLEVFEWNARRLTMNLSRLYICALAARTAAKTGHKEDFAMYEYWLDRVTREYEPMTDPAVHDTYRSTLSSKMNGAMRDEDGNIRSVL


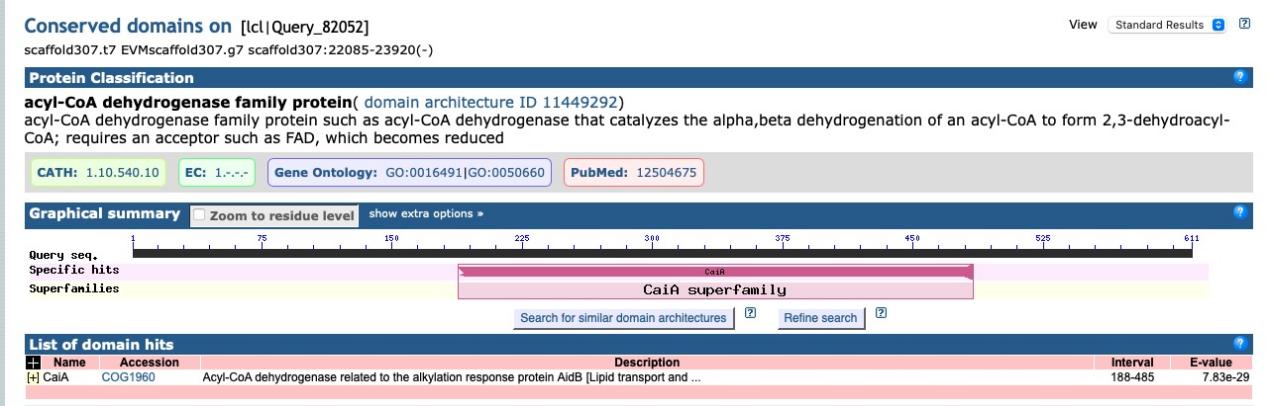


Phosphatidylinositol/phosphatidylcholine transfer protein SFH3

Hypothesized Function: Facilitates the transfer of phosphatidylinositol and phosphatidylcholine between cellular membranes.

>ZW1-scaffold478.t2 EVMscaffold478.g2 scaffold478:2596-4020(-)

MPRLSVRKSKDKLEAGANAAKAVWASGLAVSSTTVAAFEEGDEDGLKDLLDALDYTRADDPEATVPKRWVEYCGGDENKARRRWRAMAAYRESHAMDDMLRREQKEFFRIKKLYPQFWLGQDAQGNLVTLELLRDTREIVSLLRRSGISAERFARHQSFLTEYWIHQNLSRTGQMVRIIDVQDTPILAQSLGSVRQYFGATSIAMYHYPGLTAKIYFVNVSRAFRIIWSLMANFLDERTRESVHLLNSPLELPEDLMPDVFMGELSRDLEHVGIESTLSRFVRRINAQHASPQSVMTGGSGDDHEPAVLYDKDSFLAALPGSWTEISIESESLDPILRAAGVSYLSRTAFLMVSFSETITVDGAKVTVNAWAGPVSRMLSGNYDGTVEESDDEDDRVPDLEVRLDLVERPVFLVDSACTPCLRFTCKFPNGDVLTIHHGIHRFSKDKKVTNVKYKPRFGDSAPSCLLVMRKTSE

>ZW2-scaffold478.t2 EVMscaffold478.g2 scaffold478:2596-4020(-)

MPRLSVRKSKDKLEAGANAAKAVWASGLAVSSTTVAAFEEGDEDGLKDLLDALDYTRADDPEATVPKRWVEYCGGDENKARRRWRAMAAYRESHAMDDMLRREQKEFFRIKKLYPQFWLGQDAQGNLVTLELLRDTREIVSLLRRSGISAERFARHQSFLTEYWIHQNLSRTGQMVRIIDVQDTPILAQSLGSVRQYFGATSIAMYHYPGLTAKIYFVNVSRAFRIIWSLMANFLDERTRESVHLLNSPLELPEDLMPDVFMGELSRDLEHVGIESTLSHFVRRINAQHASPQSVMTGGSGDDHEPAVLYDKDSFLAALPGSWTEISIESESLDPILRAAGVSYLSRTAFLMVSFSETITVDGAKVTVNAWAGPVSRMLSGNYDGTVEESDDEDDRVPDLEVRLDLVERPVFLVDSACTPCLRFTCKFPNGDVLTIHHGIHRFSKDKKVTNVKYKPRFGDSAPSCLLVMRKTSE


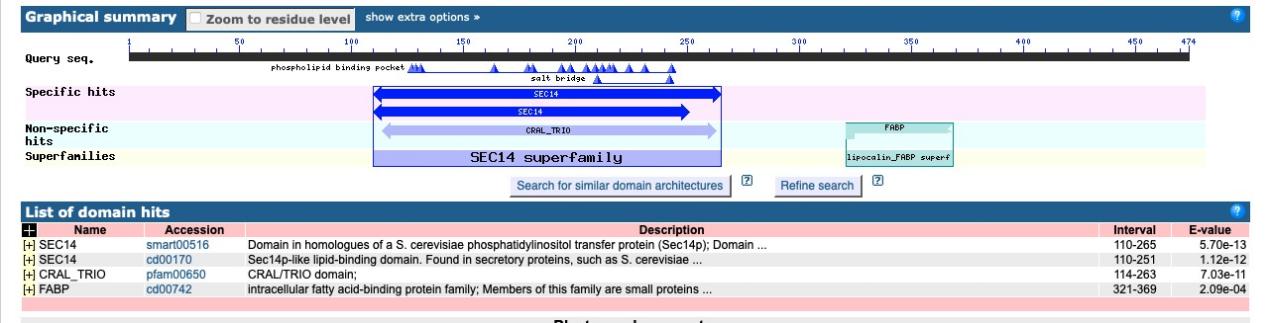


Predicted esterase

Hypothesized Function: Acts as a hydrolase, catalyzing the hydrolytic cleavage of esters in the presence of water to yield the corresponding acids and alcohols.

>ZW1-scaffold351.t2 EVMscaffold351.g2 scaffold351:2098-3102(-)

MEESGATAGAAATAAAAEEVRTLSVKELKALIARAGLGTEGCVEKADLIARAVEAHELLGQQQQEQERKQQVPKQEDASSSGSAGTCTTKAEKLGAYECLLVGHHDIEKLDVVVIVLHGFSAQNTDFVPLANEMARRMPSRRFMFVLPQAPVSAASMGAAAWWEIDVMRWLGAMQMGEAGVAKLIRDKPNGLDECRASMKEMVKAIEGRFLGDDVPLLIGGFSQGAMTAADLSWQLVGERRVHGVMMFSGAPIVVDEWHDKLTKAGKDHGLRVLMTHGRADNVLPFQASGWARDLLANSGVNVRYETHNGGHELGGPHILQAIQEFMEETMAHS

>ZW2-scaffold351.t2 EVMscaffold351.g2 scaffold351:2098-3102(-)

MEESAATAGAAATAAAAEEVRTLSVKELKALIARAGLGTEGCVEKADLIARAVEAHELLGQQQQEQERKQQVPKQEDASSSGSAGTCTTKAEKLGAYECLLVGHHDIEKLDVVVIVLHGFSAQNTDFVPLANEMARRMPSRRFMFVLPQAPVSAASMGAAAWWEIDVMRWLGAMQMGEAGVAKLIRDKPNGLDECRASMKEMVKAIEGRFLGDDVPLLIGGFSQGAMTAADLSWQLVGERRVHGVMMFSGAPIVVDEWHDKLTKAGKDHGLRVLMTHGRADNVLPFQASGWARDLLANSGVNVRYETHNGGHELGGPHILQAIQEFMEETMAHS


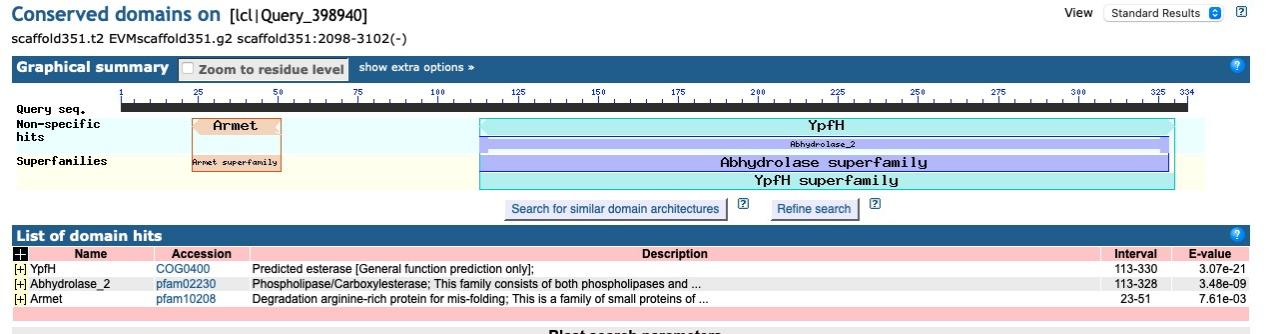


Polyunsaturated fatty acid synthase subunit C

Hypothesized Function: Participates in the biosynthesis of polyunsaturated fatty acids.

>ZW1-scaffold1624.t2 EVMscaffold1624.g2 scaffold1624:1966-6432(-)

MTKEELTSGKTEVFNYEELLEFAEGDIAKVFGPEFAVIDKYPRRVRLPAREYLLVTRVTLMDAEVNNYRVGARMVTEYDLPVNGELSEGGDCPWAVLVESGQCDLMLISYMGIDFQNQGDRVYRLLNTTLTFYGVAHEGETLEYDIRVTGFAKRLDGGISMFFFEYDCYVNGRLLIEMRDGCAGFFTNEELDAGKGVVFTRGDLAARAKIPKQDVSPYAVAPCLHKTKLNEKEMQTLVDKDWASVFGSKNGMPEINYKLCARKMLMIDRVTSIDHKGGVYGLGQLVGEKILERDHWYFPCHFVKDQVMAGSLVSDGCSQMLKMYMIWLGLHLTTGPFDFRPVNGHPNKVRCRGQISPHKGKLVYVMEIKEMGFDEDNDPYAIADVNIIDVDFEKGQDFSLDRISDYGKGDLNKKIVVDFKGIALKMQKRSTNKNPSKVQPVFANGAATVGPEASKASSGASASASAAPAKPAFSADVLAPKPVALPEHILKGDALAPKEMSWHPMARIPGNPTPSFAPSAYKPRNIAFTPFPGNPNDNDHTPGKMPLTWFNMAEFMAGKVSMCLGPEFAKFDDSNTSRSPAWDLALVTRAVSVSDLKHVNYRNIDLDPSKGTMVGEFDCPADAWFYKGACNDAHMPYSILMEIALQTSGVLTSVLKAPLTMEKDDILFRNLDANAEFVRADLDYRGKTIRNVTKCTGYSMLGEMGVHRFTFELYVDDVLFYKGSTSFGWFVPEVFAAQAGLDNGRKSEPWFIENKVPASQVSSFDVRPNGSGRTAIFANAPSGAQLNRRTDQGQYLDAVDIVSGSGKKSLGYAHGSKTVNPNDWFFSCHFWFDSVMPGSLGVESMFQLVEAIAAHEDLAGKHGIANPTFVHAPGKISWKYRGQLTPKSKKMDSEVHIVSVDAHDGVVDLVADGFLWADSLRVYSVSNIRVRIASGEAPAAASSAASVGSSASSVERTRSSPAVASGPAQTIDLKQLKTELLELDAPLYLSQDPTSGQLKKHTDVASGQATIVQPCTLGDLGDRSFMETYGVVAPLYTGAMAKGIASADLVIAAGKRKILGSFGAGGLPMHHVRAALEKIQAALPQGPYAVNLIHSPFDSNLEKGNVDLFLEKGVTVVEASAFMTLTPQVVRYRAAGLSRNADGSVNIRNRIIGKVSRTELAEMFIRPAPEHLLEKLIASGEITQEQAELARRVPVADDIAVEADSGGHTDNRPIHVILPLIINLRNRLHRECGYPAHLRVRVGAGGGVGCPQAAAAALTMGAAFIVTGTVNQVAKQSGTCDNVRKQLSQATYSDICMAPAADMFEEGVKLQVLKKGTMFPSRANKLYELFCKYDSFDSMPPAELERIEKRIFKRALQEVWEETKDFYINGLKNPEKIQRAEHDPKLKMSLCFRWYLGLASRWANMGAPDRVMDYQVWCGPAIGAFNDFIKGTYLDPAVSNEYPCVVQINLQILRGACYLRRLNALRNDPRIDLETEDAAFVYEPTNAL

>ZW2-scaffold1624.t2 EVMscaffold1624.g2 scaffold1624:1966-6432(-)

MTKEELTSGKTEVFNYEELLEFAEGDIAKVFGPEFAVIDKYPRRVRLPAREYLLVTRVTLMDAEVNNYRVGARMVTEYDLPVNGELSEGGDCPWAVLVESGQCDLMLISYMGIDFQNQGDRVYRLLNTTLTFYGVAHEGETLEYDIRVTGFAKRLDGGISMFFFEYDCYVNGRLLIEMRDGCAGFFTNEELDAGKGVVFTRGDLAARAKIPKQDVSPYAVAPCLHKTKLNEKEMQTLVDKDWASVFGSKNGMPEINYKLCARKMLMIDRVTSVDHKGGVYGLGQLVGEKILERDHWYFPCHFVKDQVMAGSLVSDGCSQMLKMYMIWLGLHLTTGPFDFRPVNGHPNKVRCRGQISPHKGKLVYVMEIKEMGFDEDNDPYAIADVNIIDVDFEKGQDFSLDRISDYGKGDLNKKIVVDFKGIALKMQKRSTNKNPSKVQPVFANGAATVGPEASKASSGASASASAAPAKPAFSADVLAPKPVALPEHILKGDALAPKEMSWHPMARIPGNPTPSFAPSAYKPRNIAFTPFPGNPNDNDHTPGKMPLTWFNMAEFMAGKVSMCLGPEFAKFDDSNTSRSPAWDLALVTRAVSVSDLKHVNYRNIDLDPSKGTMVGEFDCPADAWFYKGACNDAHMPYSILMEIALQTSGVLTSVLKAPLTMEKDDILFRNLDANAEFVRADLDYRGKTIRNVTKCTGYSMLGEMGVHRFTFELYVDDVLFYKGSTSFGWFVPEVFAAQAGLDNGRKSEPWFIENKVPASQVSSFDVRPNGSGRTAIFANAPSGAQLNRRTDQGQYLDAVDIVSGSGKKSLGYAHGSKTVNPNDWFFSCHFWFDSVMPGSLGVESMFQLVEAIAAHEDLAGKHGIANPTFVHAPGKISWKYRGQLTPKSKKMDSEVHIVSVDAHDGVVDLVADGFLWADSLRVYSVSNIRVRIASGEAPAAASSAASVGSSASSVERTRSSPAVASGPAQTIDLKQLKTELLELDAPLYLSQDPTSGQLKKHTDVASGQATIVQPCTLGDLGDRSFMETYGVVAPLYTGAMAKGIASADLVIAAGKRKILGSFGAGGLPMHHVRAALEKIQAALPQGPYAVNLIHSPFDSNLEKGNVDLFLEKGVTVVEASAFMTLTPQVVRYRAAGLSRNADGSVNIRNRIIGKVSRTELAEMFIRPAPEHLLEKLIASGEITQEQAELARRVPVADDIAVEADSGGHTDNRPIHVILPLIINLRNRLHRECGYPAHLRVRVGAGGGVGCPQAAAAALTMGAAFIVTGTVNQVAKQSGTCDNVRKQLSQATYSDICMAPAADMFEEGVKLQVLKKGTMFPSRANKLYELFCKYDSFDSMPPAELERIEKRIFKRALQEVWEETKDFYINGLKNPEKIQRAEHDPKLKMSLCFRWYLGLASRWANMGAPDRVMDYQVWCGPAIGAFNDFIKGTYLDPAVSNEYPCVVQINLQILRGACYLRRLNALRNDPRIDLETEDAAFVYEPTNAL


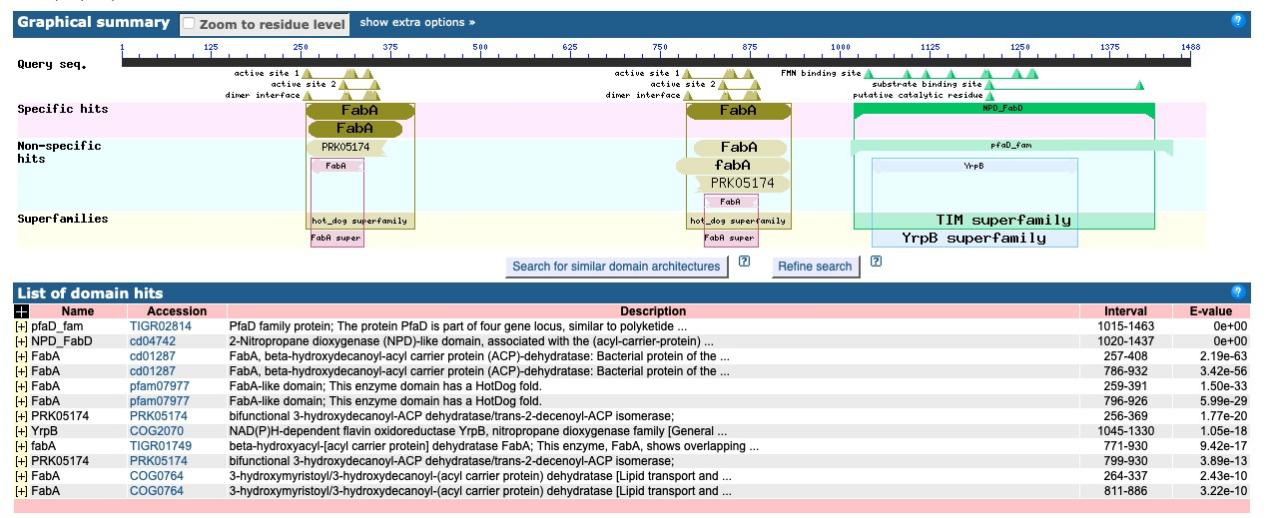


Glycerol-3-phosphate phosphatase

Hypothesized Function: Hydrolyzes glycerol-3-phosphate to produce glycerol.

>ZW1-scaffold483.t2 EVMscaffold483.g2 scaffold483:2144-3007(+)

MGGRKLLDAYKNFVLDCDGVLWHSATAIAGSVEAVSRMRAAGKRVVFVTNNSTKSRQAYLDKFEALKFEGVEVEHINTSGSAAAELCKLGGHKKVFAIGEPGLMEEFKAVGVETVEVSSDAKTGMDEAEFEDAKLEEGVSAVVVGWGRNFSFRKLCLASLYIQSGAKLIATGLDPSDKVGGKSMPGNGCNVNAIQYSVDDPQGEHTTVAGKPNSHLMENILNKYNFTGSETLMVGDRLDTDIKFAEGQAKSLLVLSGCTPIEEYESRTDVKPTHVAADLATALDMDI

>ZW2-scaffold483.t2 EVMscaffold483.g2 scaffold483:2144-3007(+)

MGGRKLLDAYKNFVLDCDGVLWHSATAIAGSVEAVSRMRAAGKRVVFVTNNSTKSRQAYLDKFEALKFEGVEVEHINTSGSAAAELCKLGGHKKVFAIGEPGLMEEFKAVGVETVEVSSDAKTGMDEAEFEDAKLEEGVSAVVVGWDRNFSFRKLCLASLYIQSGAKLIATGLDPSDKVGGKSMPGNGCNVNAIQYSVDDPQGEHTTVAGKPNSHLMENILNKYNFTGSETLMVGDRLDTDIKFAEGQAKSLLVLSGCTPIEEYESRTDVKPTHVAADLATALDMDI


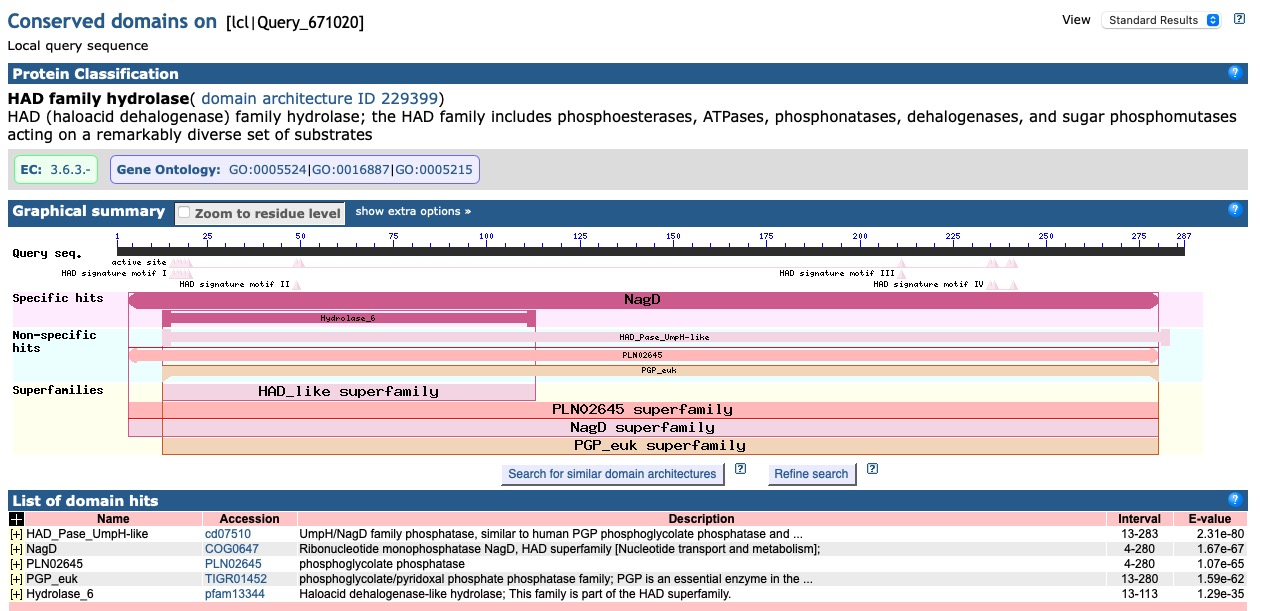


Monoacylglycerol lipase ABHD12

Hypothesized Function: Hydrolyzes glycerol monoesters of long-chain fatty acids, converting monoacylglycerols into free fatty acids and glycerol.

>ZW1-scaffold18.t23 EVMscaffold18.g23 scaffold18:61843-62919(-)

MTATTRLGVLAAATAFAVHTEKVQVETLVSAVQMTLWLMLGLLSFLIVKQNAILYIPHPDPSLSRAPDVTPLAFGDMPYEDVEIVTEDGVRIHSWLILQSAQDPAANRACNTLIYFHGNAGNVANRLPFYASLHEELKVNILAVDYRGFGSSQGEPSEPGLKEDARAVARFAADCKHIDPDRVFIFGRSLGGAVAIDLLASADCGVNVRGLVVENTFLSLAEMAMVVYPFLKPLRPFLRPPFLRNEWRPRDCIARIPCPILFISGGQDELIPPEQMRQLFRLSRRVPGTQFRLVPNGGHNDTPLHGGKEYTATIGNFFEDILSDRFVQEHHAGARRDTGASVDSILSEPDDFHHHIFS

>ZW2-scaffold18.t23 EVMscaffold18.g23 scaffold18:61843-62919(-)

MTATTRLGVLAAATAFAVHTEKVQVETLVSAVQMTLWLMLGLLSFLIVKQNAILYIPHPDPSLSRAPDVTPLAFGDMPYEDVEIVTEDGVRIHSWLILQSAQDPAANRACNTLIYFHGNAGNVANRLPFYASLHEELKVNILAVDYRGFGSSQGEPSEPGLKEDARAVARFAADCKHIDPDRVFIFGRSLGGAVAIDLLASADCGVNVRGLVVENTFLSLAEMAMVVYPFLKPLRPFLRPPFLRNEWRSRDCIARIPCPILFISGGQDELIPPEQMRQLFRLSRRVPGTQFRLVPNGGHNDTPLHGGKEYTATIGNFFEDILSDRFVQEHHAGARRDTGASVDSILSEPDDFHHHIFS


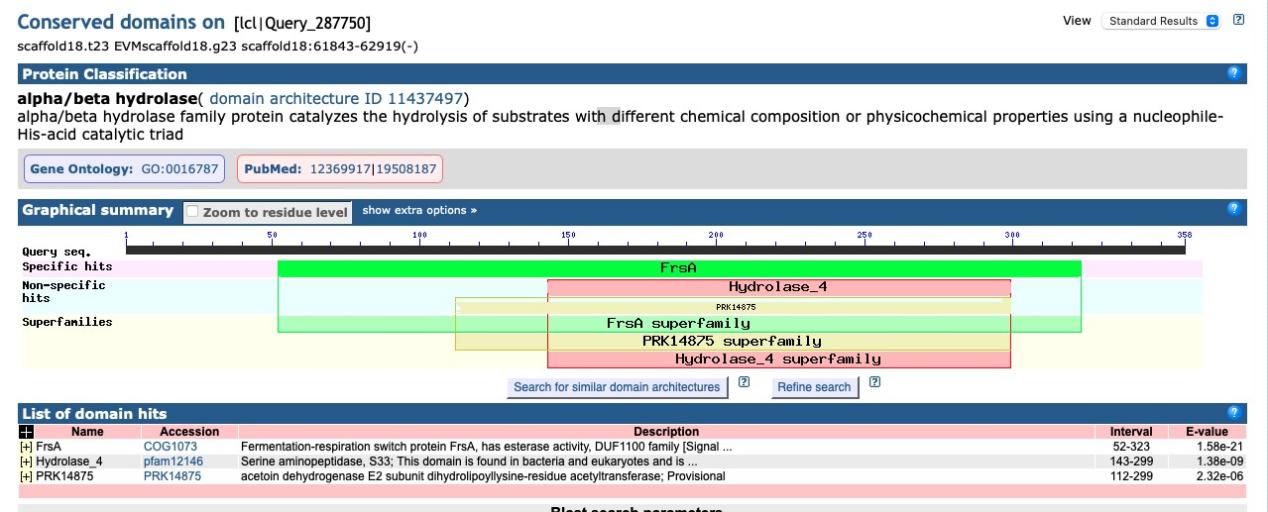


Phosphatidylserine decarboxylase

Hypothesized Function: Catalyzes the formation of phosphatidylethanolamine through the decarboxylation of phosphatidylserine.

>ZW1-scaffold602.t8 EVMscaffold602.g8 scaffold602:16159-17079(+)

MREIVYRGWAFMYSCRQHEMRDPYTSYPSLSAYFRRPLKNGVRRVDADSPGAVVCPVGAKVLHVGSTDLAKGVQAGGVRLEQVKGVSYPMEDFVGRFPSGLDTDSPAPGRTLQTVVLYLAPGDYHHFHSPVDWTIDQRRHFPGRLLPVRPWAARRVEDLYCRNERVVLNGTWEGGFFSFGAVGALNVGSIDICFDEDVLTNKESRFPYQRYTKAVSERNYAEPIDAVRGQPVGSFDFG**S**TIVLVFEASKEFTFNVTPGQTVRVGERLGTDKPRERQRPLTNCPPWPGDSSNTLTLQRLRALREEDE

> ZW2-scaffold602.t8 EVMscaffold602.g8 scaffold602:16159-17079(+)

MREIVYRGWAFMYSCRQHEMRDPYTSYPSLSAYFRRPLKNGVRRVDADSPGAVVCPVDAKVLHVGSTDLAKGVQAGGVRLEQVKGVSYPMEDFVGRFPSGLDTDSPAPGRTLQTVVLYLAPGDYHHFHSPVDWTIDQRRHFPGRLLPVRPWAARRVEDLYCRNERVVLNGTWEGGFFSFGAVGALNVGSIDICFDEDVLTNKESRFPYQRYTKAVSERNYAEPIDAVRGQPVGSFDFG**S**TIVLVFEASKEFTFNVTPGQTVRVGERLGTDKPRERQRPLTNCPPWPGDSSNTLTLQRLRALREEDE


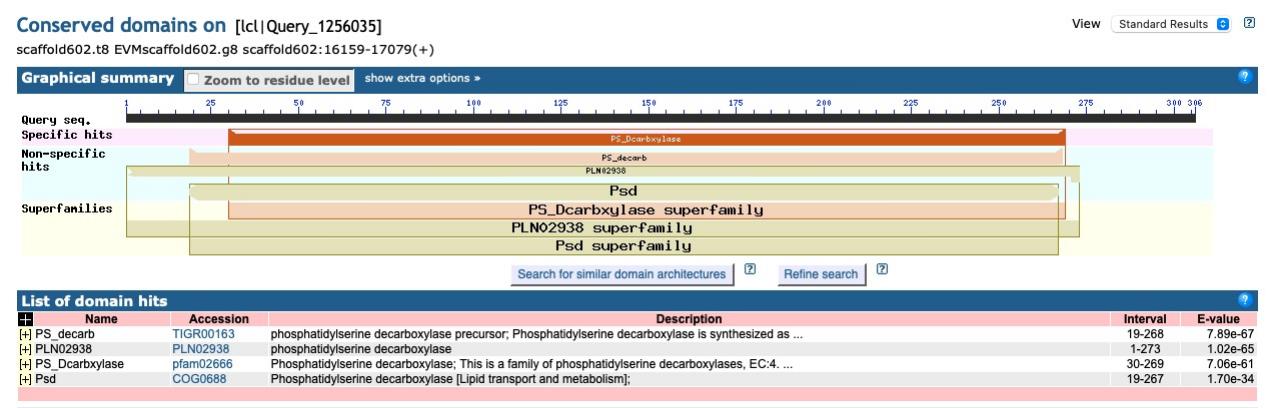


Phospholipase A1

Hypothesized Function: Specifically catalyzes the cleavage of phospholipids at the sn-1 position, releasing a fatty acid and generating a lysophospholipid.

>ZW1-scaffold880.t4 EVMscaffold880.g4 scaffold880:8647-15312(-)

MAAATSTSTSTSTSTSPAHQEAAGESGVTDKHAVAEKATPPQENGAREESPKIAQGAAAETLSAPTEPRSQEDRHEHEAAEHDEANDPGDKDDTDITQTVTVARTVSPEKRPEETLQQLQDKDMGKEDEDTTIQSQDVVEDKEDDTDVEGSLDEEENDSKSETGPLVEIPEDGADAKSETGSVVEVDIAPDHVSDTGENALGGEHPLYASLASLVNDRMAEASMNLHAMSGAKLLRLLLYCLIGNLIAVVITSISVAWSNASSEKMPGAPTLWLVLFWLVIRVLQSIWMTAVAVFFTYIVIVELEAQTFHAVDQDKLRVRKTSQVVVLLCTWCAWLLPSLNPGVTIVDLYAFVLAYRDWSFETYETFWPRQRYCVASDALFVASFFYGTIVLFKWIRIEAVKRDNWLRISLQHDGNSLDMQAVTDSLLDDHSRRHDHVRTVKGVVPRMAALYVIARLAVGEVFNFSMGFVPGTAWISVVRYCATEVPPGTPSPCTTYDDNVLTALIIVFSVWELILVAWIFVEAGRARNILSLFYLLQGRANLVSFHVLRAVFVLQMFFLYIVALVSEIGLAPVYGFYHGLTFSGVTYGDGPVSIVILAWITALVYMCLPCNWILTPRYWRKTRDTGYHYMKSYNEFALGVLGDTYIVDHLDVLAEDKEQTEFSSTDMGPADVATADAINHCLEHRASHFFSFDIAALLWNFAHTMYLTGTARFAQSDPDVLLLVEDDQFSLVAQISEESVNVYCAVFASSEMVVVAFRGNGARIPNSLASTRTPRMTEPLPAPGLGEDEEQVNVPVDAKHMSASSSAQSFGRLARMNSSILARPNSGATTSGGRNFCTWTRHQLGHVFSQLADPPLVRTDLSERYSVVASRILAVVQQVSEQKARPVFTTGHGVGGAYASLCALDLARKREEVGFSHVAVYSFGAPRFGNEAFKEKYAEMVPNHFDIANVSDSLHTQRATTLPWDPPQLARLGTLVLVDSLGSLVLRPDWLETYIMRVSKRKKFNGHEPLAYTTALMQWAHRMYGTSYRPLWTSSLHEVKLGLGSGTSAADVDKMVSNGTDPREDAPSDALEGWQTSVVRAYLQTSQVGLGPYFQEACIRIRVKRITGLHADTSTCIISCGPQGISSSLWEVGKTKPARLQHRSTLAGLRLTQSAQSPESPADMEAKSSKANLFGLGVDAAALAALPTRFADFSESDPMYFGVMQKLRPCAMLIIAGVDMAHGNFGSVCLPVTELFPIPNDKPKSVSFELTGRARRENAGDLKVHIEVDNVGDIPLGALSSSQHVGASELVVPADRRRTTATNLARYHRLAPGSGTEAIGAPPVLGVPGLGPLGSRPSALPRTASGEFVGTPGGLQLSQPMAYFYLKRNFNANSQRVVLRVGVMRATTRTLLLATHDGTPAKEIYAIVRLGKEAGRSRGAKTIHTPQWDELFTFGKLETLTGFEELKVEFYATAQDTAEEMRKLEAYGEEHLPPPMPGRSLSTASMDESRSMSGSHRRTGAEGHTRSIESMTGLLGNLGFGNSSASSINSSSGSSGSIPVGSSGHLHGGVRAGVSSVYGTAGSHFPDWNEVTPWDASGAAVAGGGLGTRLSTLSNSSSSLLQQSGPSLAAAAAALRTEPAPDDRLLGSVELPLSAIITKYQDTMPLTATEKQPLGARLKTPIMQGSKPRSMDWKDIGGSPRGRGSGSGGNFGALVGPASSVGGANNSSGAHSAGQSASGSSPGSSGLHPTSPAVANANNGSSGVNSAGGSPGPGQPGTGPASLNSGSFGSGGPGNRRRANMVRQASGSLRTIMNLATNPIGANFRMSRSNSAAGQSPAVTESAAALHRASTIDIPSGALLPESHGIISQAVPEEWKTKAANDYDERAFVVRVRVRRADQLEVQLQGSSPRPESPMTPGSLDSTASIDYPCVDSYCEVSLKDPLNGFEIAQSRRTAIMRDSVAPVWDEFFQFGKERASPVRGNEILSLEVHHWEMNGKGFSFGEVRLPMRKLQDALNMKTPFTEPLEKYDAQGNLLPRSGPSAELSFTISVTEFVNRDSLKSVSEAAAKLEFSKSPML

>ZW2-scaffold880.t4 EVMscaffold880.g4 scaffold880:8647-15312(-)

MAAATSTSTSTSTSTSPAHQEAAGESGVTDKHAVAEKATPPQENGAREESPKIAQGAAAETLSAPTEPRSQEDRHEHEAAEHDEANDPGDKDDTDITQTVTVARTVSPEKRPEETLQQLQDKDMGKEDEDTTIQSQDVVEDKEDDTDVEGSLDEEENDSKSETGSLVEIPEDGADAKSETGSVVEVDIAPDHVSDTGENALGGEHPLYASLASLVNDRMAEASMNLHAMSGAKLLRLLLYCLIGNLIAVVITSISVAWSNASSEKMPGAPTLWLVLFWLVIRVLQSIWMTAVAVFFTYIVIVELEAQTFHAVDQDKLRVRKTSQVVVLLCTWCAWLLPSLNPGVTIVDLYAFVLAYRDWSFETYETFWPRQRYCVASDALFVASFFYGTIVLFKWIRIEAVKRDNWLRISLQHDGNSLDMQAVTDSLLDDHSRRHDHVRTVKGVVPRMAALYVIARLAVGEVFNFSMGFVPGTAWISVVRYCATEVPPGTPSPCTTYDDNVLTALIIVFSVWELILVAWIFVEAGRARNILSLFYLLQGRANLVSFHVLRAVFVLQMFFLYIVALVSEIGLAPVYGFYHGLTFSGVTYGDGPVSIVILAWITALVYMCLPCNWILTPRYWRKTRDTGYHYMKSYNEFALGVLGDTYIVDHLDVLAEDKEQTEFSSTDMGPADVATADAINHCLEHRASHFFSFDIAALLWNFAHTMYLTGTARFAQSDPDVLLLVEDDQFSLVAQISEESVNVYCAVFASSEMVVVAFRGNGARIPNSLASTRTPRMTEPLPAPGLGEDEEQVNVPVDAKHMSASSSAQSFGRLARMNSSILARPNSGATTSGGRNFCTWTRHQLGHVFSQLADPPLVRTDLSERYSVVASRILAVVQQVSEQKARPVFTTGHGVGGAYASLCALDLARKREEVGFSHVAVYSFGAPRFGNEAFKEKYAEMVPNHFDIANVSDSLHTQRATTLPWDPPQLARLGTLVLVDSLGSLVLRPDWLETYIMRVSKRKKFNGHEPLAYTTALMQWAHRMYGTSYRPLWTSSLHEVKLGLGSGTSAADVDKMVSNGTDPREDAPSDALEGWQTSVVRAYLQTSQVGLGPYFQEACIRIRVKRITGLHADTSTCIISCGPQGISSSLWEVGKTKPARLQHRSTLAGLRLTQSAQSPESPADMEAKSSKANLFGLGVDAAALAALPTRFADFSESDPMYFGVMQKLRPCAMLIIAGVDMAHGNFGSVCLPVTELFPIPNDKPKSVSFELTGRARRENAGDLKVHIEVDNVGDIPLGALSSSQHVGASELVVPADRRRTTATNLARYHRLAPGSGTEAIGAPPVLGVPGLGPLGSRPSALPRTASGEFVGTPGGLQLSQPMAYFYLKRNFNANSQRVVLRVGVMRATTRTLLLATHDGTPAKEIYAIVRLGKEAGRSRGAKTIHTPQWDELFTFGKLETLTGFEELKVEFYATAQDTAEEMRKLEAYGEEHLPPPMPGRSLSTASMDESRSMSGSHRRTGAEGHTRSIESMTGLLGNLGFGNSSASSINSSSGSSGSIPVGSSGHLHGGVRAGVSSVYGTAGSHFPDWNEVTPWDASGAAVAGGGLGTRLSTLSNSSSSLLQQSGPSLAAAAAALRTEPAPDDRLLGSVELPLSAIITKYQDTMPLTATEKQPLGARLKTPIMQGSKPRSMDWKDIGGSPRGRGSGSGGNFGALVGPASSVGGANNSSGAHSAGQSASGSSPGSSGLHPTSPAVANANNGSSGVNSAGGSPGPGQPGTGPASLNSGSFGSGGPGNRRRANMVRQASGSLRTIMNLATNPIGANFRMSRSNSAAGQSPAVTESAAALHRASTIDIPSGALLPESHGIISQAVPEEWKTKAANDYDERAFVVRVRVRRADQLEVQLQGSSPRPESPMTPGSLDSTASIDYPCVDSYCEVSLKDPLNGFEIAQSRRTAIMRDSVAPVWDEFFQFGKERASPVRGNEILSLEVHHWEMNGKGFSFGEVRLPMRKLQDALNMKTPFTEPLEKYDAQGNLLPRSGPSAELSFTISVTEFVNRDSLKSVSEAAAKLEFSKSPML


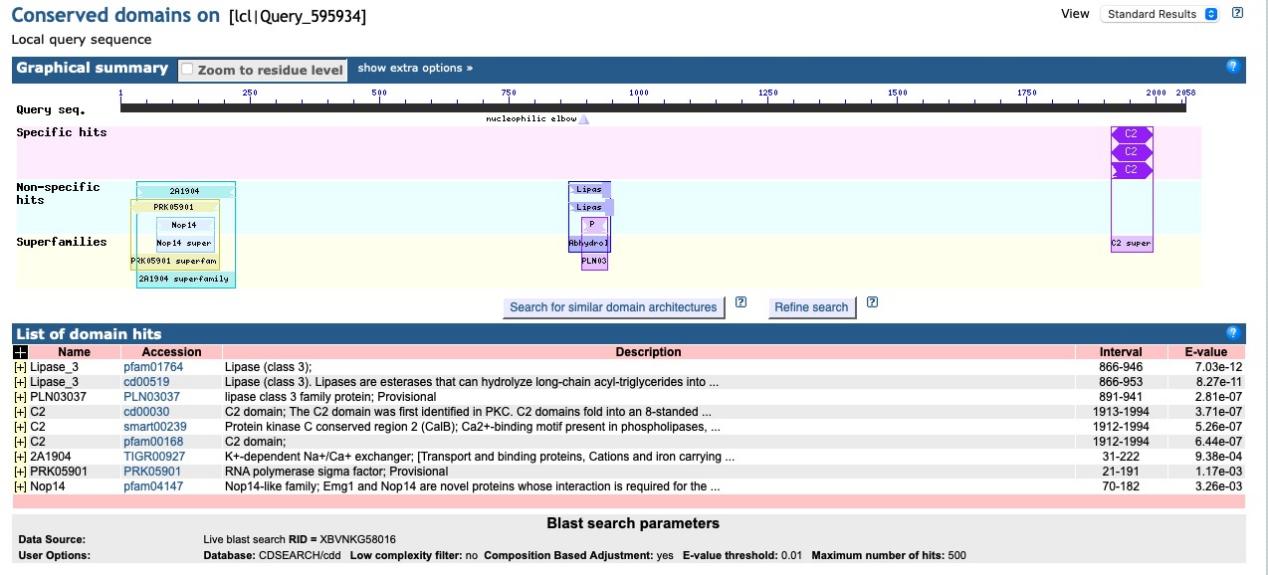


**Supplementary Note 2 A comprehensive techno-economic analysis**

To assess the practical applicability of this study, we conducted a comprehensive techno-economic analysis based on literatures^1^. The economic model assumes an annual production capacity of 10,000 tons of microbial oils with a fermentation volume of 250 m³, incorporating the following parameters: fixed capital investment (FCI) of $70.2 million, annual labor cost (C_ol_) of $0.5 million, annual utility costs (C_UT_) for steam and electricity of $7.1 million, and raw material cost (C_RM_) for glucose at $400 per ton. The cost of manufacture (COM) was calculated using the established equation: COM = 0.28FCI + 2.73C_ol_ + 1.23(C_RM_ + C_UT_). Our analysis incorporated two assumptions: (1) consistent fermentation performance of the engineered strain during scale-up, and (2) adoption of the reported lipid quantification methods using fatty acid methyl esters (FAME) and triacylglycerols (TAG) as standardized in the literature ^2, 3^. Comparative analysis revealed that our engineered strain demonstrates significant cost advantages: while *Yarrowia lipolytica* requires approximately $77.05 million to produce 10,000 tons of FAME, our strain achieves equivalent production at $68.74 million, representing a 10.79% cost reduction. Similarly, compared to *Rhodococcus opacus* PD630's TAG production cost of $90.04 million for 10,000 tons, our strain reduces costs to $74.78 million, achieving a remarkable 16.95% reduction.

**Supplementary Video 1 Molecular dynamics simulation of the G3P trajectory on the ZW1 G3PP protein.**

**Supplementary Video 2 Molecular dynamics simulation of the G3P trajectory on the ZW2 G3PP protein.**

Reference

1. A. A. Koutinas, A. Chatzifragkou, N. Kopsahelis, S. Papanikolaou, I. K. Koobos, Design and techno-economic evaluation of microbial oil production as a renewable resource for biodiesel and oleochemical production. *Fuel* 2014, 116, 566-577.

2. K. J. Qiao, T. M. Wasylenko, K. Zhou, P. Xu, G. Stephanopoulos, Lipid production in *Yarrowia lipolytica* is maximized by engineering cytosolic redox metabolism*. Nat*. *Biotechnol*. 2017, 35, 173-177.

3. H. M. Kim, T. U. Chae, S. Y. Choi, W. J. Kim, S. Y. Lee, Engineering of an oleaginous bacterium for the production of fatty acids and fuels. *Nat*. *Chem*. *Biol*. 2019, 15, 721-729.
